# Diversity-oriented synthetic strategy for developing a chemical modulator of protein*–*protein interaction

**DOI:** 10.1038/ncomms13196

**Published:** 2016-10-24

**Authors:** Jonghoon Kim, Jinjoo Jung, Jaeyoung Koo, Wansang Cho, Won Seok Lee, Chanwoo Kim, Wonwoo Park, Seung Bum Park

**Affiliations:** 1 Department of Biophysics and Chemical Biology, CRI Center for Chemical Proteomics, Seoul National University, Seoul 151-747, Korea; 2 Department of Chemistry, Seoul National University, Seoul 151-747, Korea

## Abstract

Diversity-oriented synthesis (DOS) can provide a collection of diverse and complex drug-like small molecules, which is critical in the development of new chemical probes for biological research of undruggable targets. However, the design and synthesis of small-molecule libraries with improved biological relevance as well as maximized molecular diversity represent a key challenge. Herein, we employ functional group-pairing strategy for the DOS of a chemical library containing privileged substructures, pyrimidodiazepine or pyrimidine moieties, as chemical navigators towards unexplored bioactive chemical space. To validate the utility of this DOS library, we identify a new small-molecule inhibitor of leucyl-tRNA synthetase–RagD protein–protein interaction, which regulates the amino acid-dependent activation of mechanistic target of rapamycin complex 1 signalling pathway. This work highlights that privileged substructure-based DOS strategy can be a powerful research tool for the construction of drug-like compounds to address challenging biological targets.

## Abstract (web summary)

Diversity-oriented synthesis is useful for generating complex molecular structures occupying diverse molecular space. Here the authors report a strategy to access libraries of privileged heterocyclic structures, and furthermore identify an inhibitor of LRS–RagD protein–protein interaction.

The molecular diversity and complexity in a screening collection of drug-like small molecules is a paramount breakthrough in the discovery of novel small-molecule modulators for currently ‘undruggable' targets, including protein–protein interactions (PPIs) and protein–nucleic acid interactions^1^,^2^,^3^. Towards this end, a strategy termed diversity-oriented synthesis (DOS) was devised, which seeks to populate the vast area of new chemical space made up of diverse and three-dimensional (3D) complex drug-like compounds^4^,^5^,^6^. Although DOS has emerged as an indispensable tool to promote the unbiased screening of compounds and their interactions with diverse biological targets, one of the key challenges in this field is the identification of appropriate chemical structures that will exhibit improved biological relevance and high molecular diversity. To address this issue, synthetic community has been developing many DOS-based approaches for the generation of compound libraries embodying core scaffolds of natural products or its mimetics^7^,^8^,^9^,^10^,^11^. Natural products have inherent bioactivity and high bioavailability; thus, the natural product-inspired DOS libraries with biological relevance could be of great value for the identification of bioactive compounds^12^,^13^,^14^.

With the goal of targeting unexplored biologically relevant chemical space, we postulated that privileged structures could also serve as ‘chemical navigators' and therefore reported a privileged substructure-based DOS (pDOS) strategy, which targets the synthesis of diverse polyheterocyclic skeletons containing privileged substructures through complexity-generating reactions in order to maximize the unbiased coverage of bioactive space^15^,^16^,^17^. By incorporating privileged substructures into a rigid core skeleton, we envisioned that the resulting compounds would exhibit enhanced interactions with various biomacromolecules including proteins and DNA/RNA. Consequently, we demonstrated the importance of pDOS strategy through the discovery of new bioactive small molecules that interact with a wide range of biological targets^18^,^19^.

As a continuation of our previous work, we identified pyrimidine as a new privileged substructure that could be used to navigate through bioactive chemical space. The pyrimidine moiety is commonly present in various bioactive small molecules, and it plays a critical role as a nucleoside analogue in various kinase inhibitors or adenosine receptor modulators due to its hydrogen bonding ability (Fig. 1a)^20^,^21^,^22^. Therefore, many synthetic efforts towards pyrimidine-containing species have been focused on aromatic monocyclic or bicyclic skeletons, which limits the structural diversity of the pyrimidine-containing core skeletons. In addition, the 3D structural complexity of the core skeletons becomes important because planar frameworks less frequently comprise FDA (Food and Drug Administration) -approved chemical entities, especially in regard to ‘undruggable' targets^23^,^24^,^25^.

To expand the molecular diversity beyond monocyclic and bicyclic pyrimidine skeletons, we develop a new pDOS strategy towards the divergent synthesis of natural product-like polyheterocycles containing pyrimidodiazepine or pyrimidine. Diazepine is also often found in complex natural products that exhibit a wide range of biological activities, and is known to be a prominent privileged structure that can improve the bioavailability and bioactivity of compounds^26^,^27^. In addition, seven-membered rings that are fused to aromatic rings generally have higher conformational flexibility and a greater number of reactive sites than six- or five-membered fused ring systems, as confirmed by the direct comparison of pyrimidine-embedded tricyclic 6/6/6 and 6/7/6 systems by overlaying the energy-minimized conformers aligned along the pyrimidine substructure (Fig. 1b). Thus, pyrimidodiazepine can serve as a versatile intermediate to access highly diverse and complex polyheterocycles through the incorporation of additional ring systems, which forms the basis of a new pyrimidodiazepine-based pDOS pathway. To establish the pDOS pathway, we first design and synthesize highly functionalized pyrimidodiazepine intermediates **1** containing five reactive sites (A–E). In our pDOS strategy, intermediates **1** can be transformed into nine distinct pyrimidodiazepine- or pyrimidine-containing scaffolds (**I**–**IX**) via pairing different functional groups at each reactive site (Fig. 1c). Then, we conduct ELISA-based high-throughput screening (HTS) of leucyl-tRNA synthetase (LRS)–Ras-related GTP-binding protein D (RagD) interaction^28^ to validate whether our distinct scaffolds can target unexplored biologically relevant chemical space. This screening exercise lead to the identification of an effective hit compound, **21f**, which regulates the amino acid-dependent activation of mechanistic target of rapamycin complex 1 (mTORC1) activity via specific inhibition of LRS–RagD interaction. **21f** can serve as a research tool with a novel mode of action for specific modulation of mTORC1 signalling pathway. Collectively, we confirm that pDOS is an appropriate strategy to target challenging biological targets.

## Results

### Pairing strategies for scaffolds I–III

**Figure 2:**
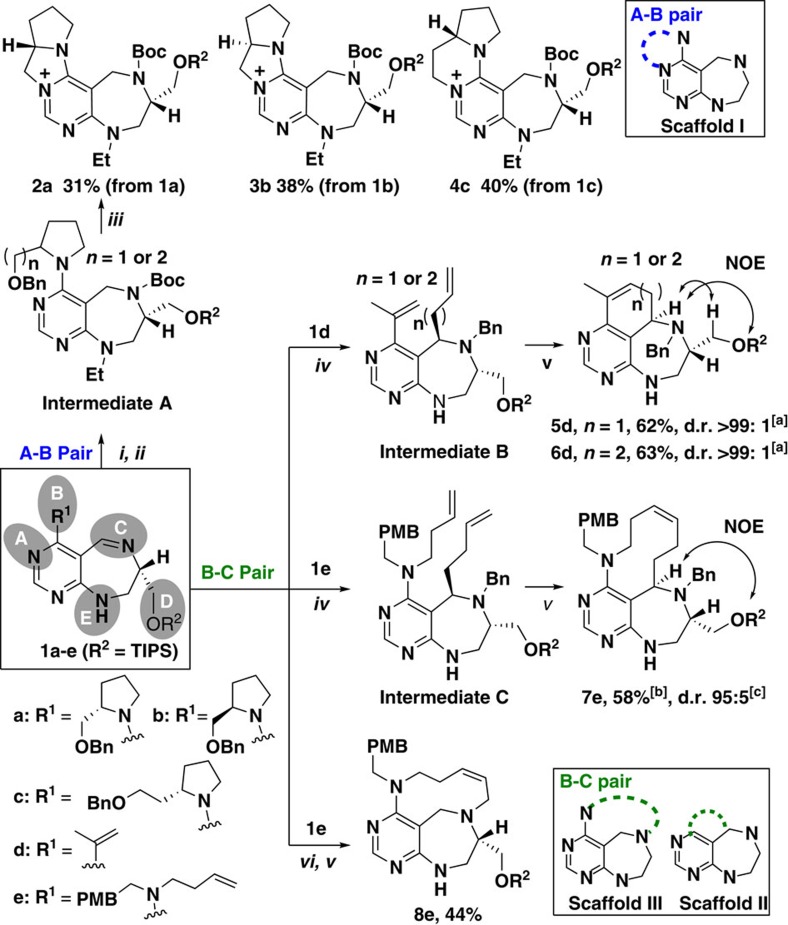
A–B and B–C paring pathways for synthesis of scaffolds I–III. Reagents and conditions: (**i**) NaBH_4_, MeOH, 0 °C→r.t. then Boc_2_O, TEA, DCM, 0 °C→r.t.; (**ii**) EtI, NaH, DMF, 0 °C→r.t.; (**iii**) Pd(OH)_2_/C, H_2_, MeOH, then MsCl, TEA, DCM, 0 °C→r.t.; (**iv**) BnBr, ACN, 80 °C, then RMgBr, THF, −78 °C→r.t.; (**v**) Grubbs' second-generation catalyst (20 mol%), toluene, reflux; and (**vi**) NaBH_4_, MeOH, 0 °C→r.t., then allyl bromide, TEA, DMF, 0 °C→r.t. TIPS, triisopropylsilyl; TEA, triethylamine; MsCl, methanesulfonyl chloride; BnBr, benzyl bromide; ACN, acetonitrile. ^[a]^Determined by LC-MS analysis of crude reaction mixture and by ^1^H NMR analysis of samples purified by short silica-gel column. ^[b]^Yield of the isolated major diastereomer. ^[c]^Determined by LC-MS analysis of crude reaction mixture. ^1^H NMR, proton nuclear magnetic resonance. LC-MS, liquid chromatography-mass spectrometry.

For the synthesis of scaffolds **I**–**III** through A–B or B–C pairs, suitable functional groups at the B position were imperative as they participated in the pairing reaction (Fig. 2). First, substrates **1a**–**e** were readily synthesized through a series of transformations (Supplementary Figs 1–3). For the synthesis of scaffold **I**, substrates **1a**–**c** were treated with sodium borohydride (NaBH_4_) to afford secondary amines; the resulting secondary amines were protected with Boc and aniline was *N*-alkylated, which allowed for the formation of intermediates **A**. Scaffold **I** was efficiently generated via A–B pairing through an intramolecular substitution reaction, when intermediates **A** were subjected to debenzylation and the resultant alcohols were subsequently activated with methanesulfonyl chloride (MsCl). Consequently, we obtained stereochemically enriched tetracycles **2a**, **3b** and **4c** (scaffold **I**), containing a pyrimidinium moiety, in moderate yields.

Next, we employed ring-closing metathesis (RCM) for the synthesis of scaffolds **II** and **III** via B–C pairing; in this case, a vinyl group was introduced at the C-reactive site of the key substrate. Accordingly, **1d** and **1e** were subjected to *N-*alkylation with benzyl bromide to afford iminium ions. Diastereoselective nucleophilic addition of allyl or homoallyl Grignard reagents to the resultant iminium ions generated intermediates **B** and **C**; RCM with Grubbs' second-generation catalyst provided **5d**, **6d** and **7e** (scaffold **II**), fused with six-, seven- and 10-membered rings, with good to excellent diastereoselectivity (Supplementary Figs 7–9 and 34–35). Notably, this diastereoselectivity without chiral auxiliaries or catalysts could be rationalized by the preferential approach of Grignard reagents towards the less hindered *re*-face of the imine in **1d** and **1e**. The stereochemistry of each product was confirmed by nuclear Overhauser effect (NOE) spectroscopy (Supplementary Figs 37,39 and 41). To generate scaffold **III**, **1e** was treated with NaBH_4_. Allylation of the resulting secondary amine and subsequent RCM with Grubbs' second-generation catalyst yielded **8e** (scaffold **III**), fused with 10-membered rings, in 44% overall yield.

### Pairing strategies for scaffolds IV–IX

**Figure 3:**
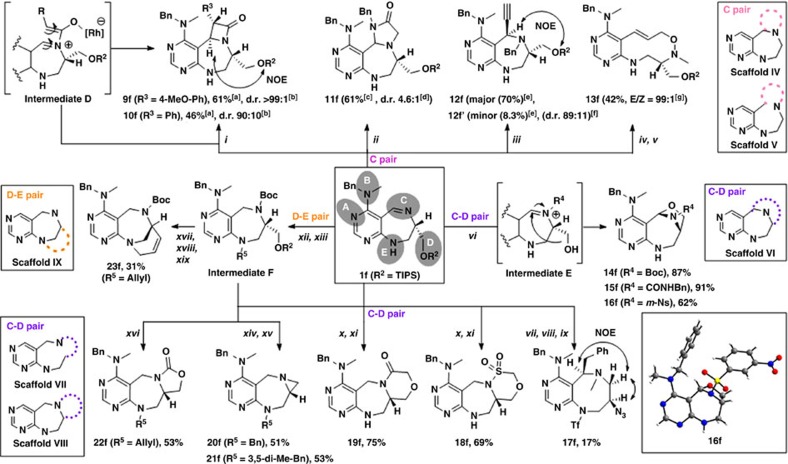
C, C–D and D–E paring pathways for synthesis of scaffolds IV–VII. Reagents and conditions: (**i**) Rh(PPh_3_)_3_Cl (10 mol%), 4-picoline *N*-oxide, 4-ethynylanisole or phenylacetylene, ACN, μ-wave, 90 °C, 35 min; (**ii**) *N*-benzyl-2-chloroacetamide, NaBr, DMF, μ-wave, 110 °C, 30 min, then DBU, DMF; (**iii**) BnBr, ACN, 80 °C, then ethynylmagnesium bromide, THF, −78 °C→r.t.; (**iv**) MeI, ACN, 40 °C, then vinylmagnesium bromide, THF, −78 °C→r.t.; (**v**) *m-*CPBA, DCM, then 3-chlorobenzoic acid; (**vi**) HF/pyridine/THF, then electrophiles (Boc_2_O, benzyl isocyanate or *m*-NsCl), DCM; (**vii**) MeI, ACN, 40 °C, then benzylmagnesium bromide, THF, −78 °C→r.t.; (**viii**) TBAF, THF; (**ix**) Tf_2_O, toluene, −40 °C, then NaN_3_, −78 °C→r.t.; (**x**) NaBH_4_, MeOH, 0 °C →r.t., then chloromethane sulfonyl chloride or chloroacetic anhydride, TEA, DCM, 0 °C→r.t.; (**xi**) TBAF, THF, then Cs_2_CO_3_, DMF, 90 °C; (**xii**) NaBH_4_, MeOH, 0 °C→r.t., then Boc_2_O, TEA, DCM, 0 °C→r.t.; (**xiii**) BnBr, 3,5-dimethylbenzyl bromide or allyl bromide, NaH, DMF, 0 °C→r.t.; (**xiv**) TBAF, THF, then TFA, DCM; (**xv**) PS-PPh_3_, DEAD, THF; (**xvi**) TBAF, THF, then NaH, THF, 0 °C→r.t.; (**xvii**) TBAF, THF, then Dess–Martin periodinane, DCM; (**xviii**) Ph_3_P^+^CH_3_Br^−^, MeLi, THF, 0 °C→r.t.; (**xix**) Grubbs' second-generation catalyst (20 mol%), toluene, reflux. DBU, 1,8-diazabicyclo[5.4.0]undec-7-ene; *m-*CPBA, 3-chloroperbenzoic acid; *m*-NsCl, 3-nitrobenzenesulfonyl chloride; TBAF, tetra-*n*-butylammonium fluoride; Tf_2_O, trifluoromethanesulfonic anhydride; PS-PPh_3_, polystyrene triphenylphosphine; and DEAD, diethyl azodicarboxylate. ^[a]^Yield of isolated major diastereomer. ^[b]^Determined by LC-MS analysis of crude reaction mixture. ^[c]^Yield of the mixture of diastereomers. ^[d]^Determined by ^1^H NMR spectroscopy. ^[e]^Yield of isolated diastereomer. ^[f]^Determined from purified yield of each diastereomer. ^[g]^Determined by LC-MS analysis of crude reaction mixture after treatment of 3-chlorobenzoic acid. ^1^H NMR, proton nuclear magnetic resonance; LC-MS, liquid chromatography-mass spectrometry.

As shown in Fig. 3, the reactive site B did not participate in the pairing reactions for the synthesis of scaffolds **IV**–**IX**. Therefore, we synthesized **1f** as a key substrate containing an *N*-benzyl-*N*-methyl amine moiety at the B position (Supplementary Fig. 2). For the preparation of scaffolds **IV** and **V**, the imine moiety of **1f** was the major synthetic functionality. Unlike in other pairing pathways, the imine moiety acts as both a nucleophile and electrophile concurrently, and thus, other cyclic structures could be introduced at the C position. First, we employed the rhodium-catalyzed oxygenative [2+2] cycloaddition of the alkyne and imine to generate a β-lactam ring^29^. The imine moiety of **1f** reacted with rhodium-complexed ketene species generated by the catalytic oxidation of the metal vinylidene complex to form zwitterionic intermediate **D**, which could be subjected to a conrotatory electrocyclic ring-closure reaction. After reaction condition screening (data not shown), *trans* isomers **9f** and **10f** (scaffold **IV**) were obtained in 61% and 46% yields, respectively, with good to excellent diastereoselectivity (Supplementary Figs 10 and 11). We performed NOE analysis and gradient-selected correlation spectroscopy (COSY) experiment to confirm the relative stereochemistry of **9f** and **10f** (Supplementary Figs 44 and 46). To construct another ring system, **1f** was reacted with *N*-benzyl-2-chloroacetamide to afford the iminium ion; intramolecular nucleophilic attack of the amide on the iminium ion provided imidazolidinone-containing **11f** (scaffold **IV**) as a diastereomeric mixture (d.r=6:1) in 61% overall yield. Moreover, without the incorporation of an additional ring structure, we obtained stereochemically enriched and highly functionalized pyrimidodiazepine **12f** (major, scaffold **IV**) and **12f**'(minor, scaffold **IV**) in 70% and 8.3% yields, respectively, via the reaction of **1f** with benzyl bromide, followed by *re*-face selective nucleophilic addition of an ethynyl Grignard reagent on the iminium ion. To generate scaffold **V**, **1f** was subjected to *N-*alkylation with methyl iodide, followed by nucleophilic addition of vinylmagnesium bromide to the resultant iminium ion afforded **SI**-**7** (Supplementary Fig. 4). Oxidation of **SI**–**7** with 3-chloroperbenzoic acid (*m*-CPBA) and conversion of seven-membered ring into 10-membered ring via *in situ* [2,3]-sigmatropic ring expansion of the resultant *N*-oxide provided **13f** (scaffold **V**) with excellent *(E)*-selectivity (Supplementary Figs 4 and 12)^30^.

For the synthesis of scaffolds **VI**–**VIII**, the C–D pairing pathways were investigated. Deprotection of the triisopropylsilyl (TIPS) group from **1f** provided an alcohol product, that was readily converted to the desired bridged oxazolidines **14f**, **15f** and **16f** (scaffold **VI**) in good to excellent yields upon treatment with various *N*-modifying agents such as Boc anhydride (Boc_2_O), benzyl isocyanate and 3-nitrobenzene sulfonyl chloride (*m*-NsCl) through iminium formation and subsequent intramolecular nucleophilic addition of the hydroxyl moiety^31^. The structure of bridged oxazolidine **16f** was confirmed by X-ray crystallographic analysis (Supplementary Fig. 5 and Supplementary Tables 1–5, CCDC number 1500586). To generate scaffold **VII**, **1f** was subjected to *N-*alkylation with methyl iodide, followed by nucleophilic addition of benzylmagnesium bromide to the resultant iminium ion and subsequent removal of the TIPS group afforded **SI**-**8** (Supplementary Fig. 6). Then, the hydroxyl moiety of **SI**-**8** was activated with trifluoromethanesulfonic anhydride to provide an aziridium intermediate and the subsequent nucleophilic ring expansion with azide anion afforded **17f** (scaffold **VII**) containing an eight-membered diazocane ring. In fact, benzodiazocine is known as an important pharmacophore, but the synthesis of diazocane has not been extensively studied due to the unfavourable transannular strain in medium-sized rings^32^. The stereochemistry of **17f** was confirmed by NOE spectroscopy (Supplementary Fig. 60). For the preparation of scaffold **VIII**, **1f** was treated with NaBH_4_ to afford a secondary amine, which underwent sulfonamide or amide formation upon treatment with chloromethane sulfonyl chloride or chloroacetic anhydride. The subsequent removal of the TIPS group and intramolecular nucleophilic attack of the resultant alcohols with electrophiles afforded **18f** or **19f** (scaffold **VIII**), fused with six-membered sultam or lactam rings. Furthermore, to generate **20f** and **21f** (scaffold **VIII**) containing an aziridine ring, **1f** was treated with NaBH_4_, and the resulting secondary amine was protected with Boc and aniline was *N*-alkylated to provide intermediates **F** (R^5^=Benzyl or 3,5-dimethylbenzyl). The removal of the TIPS and Boc group of intermediate **F**, followed by subsequent cyclization, produced **20f** or **21f** (scaffold **VIII**) in 51 and 53% overall yields, respectively. Finally, oxazolidinone-containing **22f** (scaffold **VIII**) was obtained through the TIPS deprotection of intermediate **F** (R^5^=allyl) and subsequent cyclization. For the synthesis of scaffold **IX** via D–E pairing, the removal of the TIPS group of intermediate **F** (R^5^=allyl) followed by Dess–Martin periodinane oxidation provided an aldehyde. The resulting unstable aldehyde was immediately subjected to Wittig olefination, followed by RCM with Grubbs' second-generation catalyst to afford **23f** (scaffold **IX**) containing bridge-head [4,3,1] ring systems.

### Cheminformatics analysis

**Figure 4:**
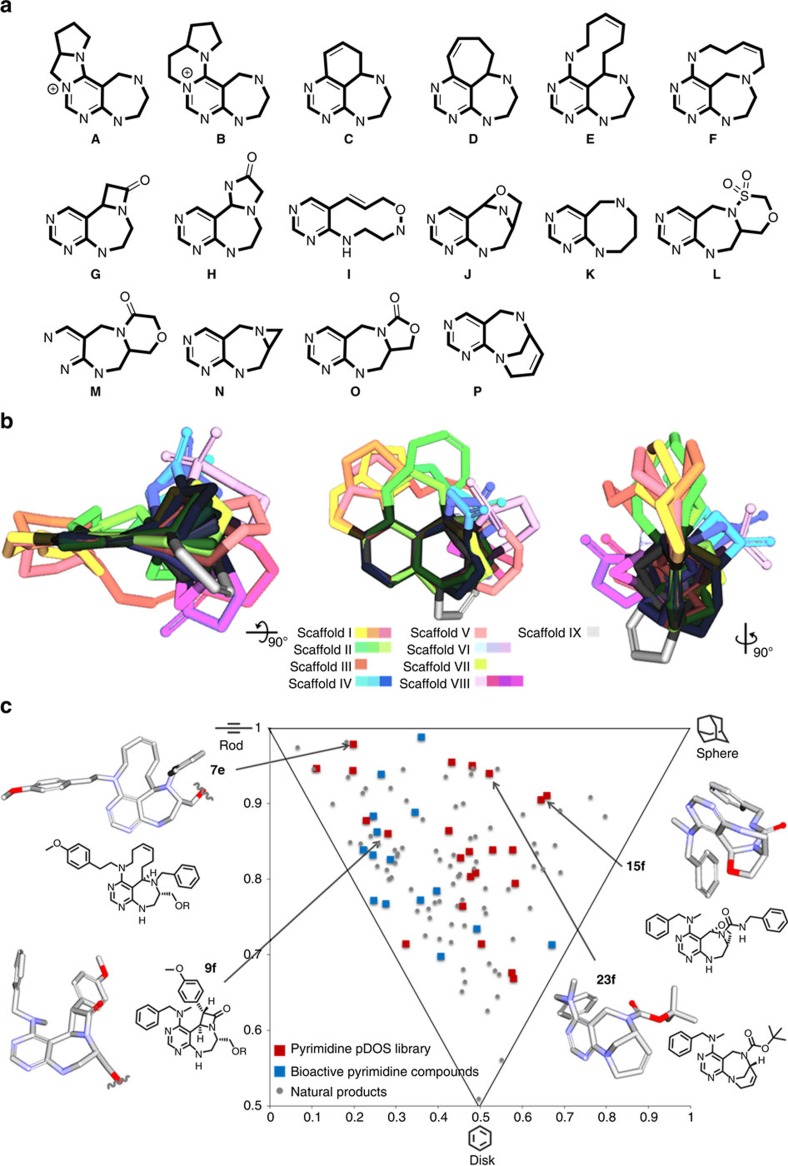
Chemoinformatic analysis for structural diversity and 3D complexity of pDOS library. (**a**) Core skeletons of 16 natural product-like pyrimidodiazepine- or pyrimidine-containing polyheterocycles. (**b**) Overlay of energy-minimized conformers of 16 core skeletons aligned by the pyrimidine substructure. (**c**) PMI plot. The 3D molecular shape of pDOS library (red squares) was quantitatively compared with that of 15 FDA-approved drugs embedded with pyrimidine moiety (blue squares) and 71 bioactive natural products (grey dots).

Through this pDOS strategy, we synthesized 16 distinct pyrimidodiazepine- or pyrimidine-containing polyheterocycles with high 3D complexity, skeletal diversity and biological relevancy (Fig. 4a). Notably, unique architectures of bioactive natural products such as medium-sized rings^33^ (**E**, **F**, **I** and **K**), bridge-head bicyclic structures^34^ (**J** and **P**) and β-lactam rings^35^,^36^ (**G**) were successfully incorporated into the pyrimidine-containing core skeletons. When we overlaid the energy-minimized conformers of each scaffold in 3D space by aligning the pyrimidine substructure, we clearly demonstrated the skeletal diversity and structural complexity of the resulting polyheterocycles (Fig. 4b). To quantitatively analyse the molecular diversity of this pDOS pathway, we performed principal moment of inertia (PMI) analysis to compare the degree of shape diversity of the representative polyheterocycles (Supplementary Fig. 14) of this study with the collection of 15 FDA-approved drugs containing pyrimidine moiety^37^ (Supplementary Fig. 15) and 71 bioactive natural products (Supplementary Table 6)^38^. The reference set of drugs containing pyrimidine moiety were narrowly dispersed in the region of rod-and disc-like shapes. On the other hand, our compounds were widely dispersed in the PMI plot, similar to natural products, and possessed more spherical characteristics than the FDA-approved drug set, which indicates the excellent shape diversity of newly synthesized core skeletons in this study (Fig. 4c and Supplementary Fig. 13).

### Biological evaluation

Diversification of the 3D molecular shapes of drug-like core skeletons may lead to various interactions with diverse biopolymers and allow for the identification of specific modulators of challenging targets such as PPIs and protein–nucleic acid interactions. To investigate this possibility, we have attempted to identify novel chemical inhibitors of the LRS–RagD interaction^28^ for the modulation of mTORC1—a dominant effector that regulates cellular growth, proliferation and autophagy^39^. Upon activation of mTORC1 by multiple upstream inputs such as growth factors, energy status and amino acids, particularly the branched-chain amino acid leucine (Leu), mTORC1 plays a crucial role in triggering eukaryotic cell growth and proliferation through stimulating protein biosynthesis and other anabolic processes while suppressing a catabolic process, namely autophagy^40^. Consequently, the dysregulation of mTORC1 activation could lead to malfunction in central biological pathways that could lead to cancer cell growth, survival and proliferation^41^.

**Figure 5:**
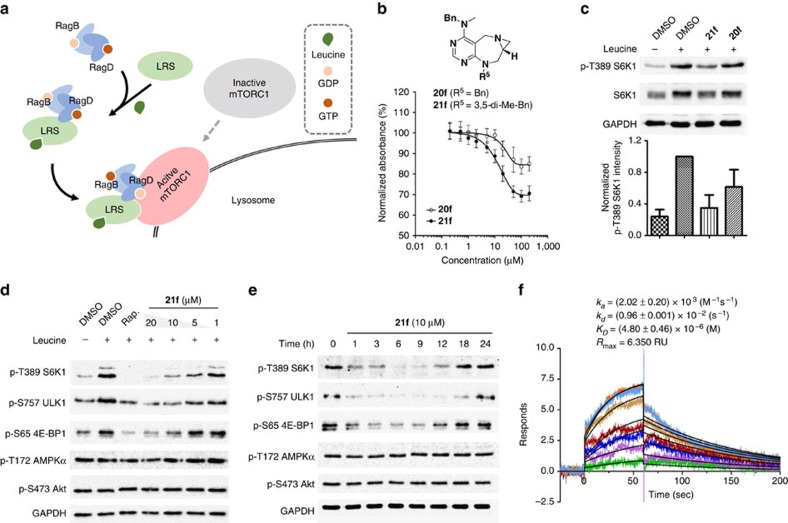
Discovery of chemical modulator for LRS–RagD interaction. (**a**) Noncanonical role of LRS. Leucine-loaded LRS binds to RagD, which promotes the translocation of mTORC1 to lysosomal surface and subsequent activation. (**b**) Dose–response curves in ELISA of **20f** and **21f**. The results represent the mean of three biological replicates; error bars represent the s.e.m. (**c**) Effects of **20f** and **21f** on mTORC1 signalling pathway. HEK293T cells were treated with 20 μM of **20f** and **21f** for 3 h. As a negative control, cells were deprived of leucine for 3 h. Level of phospho-T389 S6K1 were quantified against a DMSO control. The bar graph represents the mean of five biological replicates; error bars represent the s.e.m. (**d**) Dose-dependent effects of **21f** to mTORC1 signalling pathway. HEK293T cells were treated with 20, 10, 5 and 1 μM of **21f** for 3 h. As a negative control, cells were deprived of leucine for 3 h. Rapamycin (Rap) was used as a positive control and cells were treated with 200 nM of Rap for 3 h. (**e**) Time-course study of the inhibitory effect of **21f** on mTORC1 signalling pathway for 0–24 h. HEK293T cells were treated with 10 μM of **21f**. Phospho-T389 S6K1, phospho-S757 ULK1, phospho-S65 4E-BP1, phospho-T172 AMPKα, phospho-S473 Akt, S6K1 and GAPDH were determined by western blot. The western blot results shown are representative of three biological replicates. (**f**) Sensorgrams of SPR spectroscopy of **21f** showed its concentration-dependent binding to purified LRS. The concentration plotted are 1, 2.5, 5, 10, 12.5, 15, 17.5 and 20 μM, in order of increasing **21f**. The curve fittings are shown in black. The sensorgrams represent the mean of two biological replicates. The full gel images of western blotting are in Supplementary Fig. 22.

LRS catalyses the conjugation of Leu to its cognate tRNA to form an aminoacyl-tRNA, which serves as a precursor for protein synthesis^42^. In addition to its canonical role in protein biosynthesis, a recent study suggested a noncanonical role of LRS in amino acid-dependent mTORC1 activation by sensing intracellular Leu concentration^28^. According to this study, LRS can mediate Leu signalling to mTORC1 via direct binding to RagD-GTP protein and form a LRS–RagD protein complex in a Leu-dependent manner, which leads to the translocation of mTORC1 to the lysosome membrane and subsequent activation of mTORC1 (Fig. 5a). Thus, LRS–RagD interactions could serve as a Leu-sensing mechanism in the amino acid-dependent activation of mTORC1 (refs 28, 41). Given that the LRS–RagD interaction and structures of human LRS and RagD have not yet been fully characterized, the discovery of novel small-molecule PPI inhibitors of LRS and RagD could shed light on the molecular mechanism of mTORC1 activation in an amino acid-dependent manner. In addition, they could serve as lead compounds for the development of potential therapeutic agents with new modes of action to treat human diseases linked to the oncogenic activation of mTORC1 (refs 39, 41).

To identify novel small-molecule PPI modulators of the LRS–RagD interaction, our pDOS library was subjected to ELISA-based HTS using purified human LRS and GST (glutathione-*S*-transferase)-tagged RagD (Fig. 5b and Supplementary Fig. 16), where the antibody-based signal was lowered upon treatment with LRS–RagD interaction inhibitors. This screening exercise led to the identification of aziridine-containing **20f** and **21f** as dose-dependent inhibitors of the LRS–RagD interaction. On the basis of this initial data, we hypothesized that **20f** and **21f** might inhibit the LRS-mediated Leu signalling to mTORC1 via direct disruption of the LRS–RagD interaction. As shown in Fig. 5c, the level of phosphorylated p70 ribosomal protein S6 kinase 1 (S6K1) (ref. 43), a typical mTORC1 kinase substrate, was decreased in the absence of Leu. Similarly, **20f** and **21f** suppressed the phosphorylation of S6K1 even in the presence of Leu. On the basis of the reduction of LRS–RagD interaction by ELISA (**20f** 15.5%; **21f** 30.2%) and the suppression of phosphorylated S6K1 by western blotting (**20f** 16.4%; **21f** 48.9%), we selected **21f** as the candidate compound and subjected it to further western blot analysis and biophysical study using surface plasmon resonance (SPR) spectroscopy.

As shown in Fig. 5d, **21f** downregulated not only the phosphorylation of S6K1 but also eukaryotic translation initiation factor 4E-binding protein 1 (4E-BP1) (ref. 43) and mammalian autophagy-initiating kinase (ULK1) (ref. 44), known substrates of mTORC1 kinase, in a dose-dependent manner in HEK293T cell lines and two additional cell lines, Ca Ski cervical cancer cell and DU145 prostate cancer cell lines (Supplementary Fig. 17). Interestingly, **21f** affected neither mTORC2 signalling pathway (the level of phospho-S473 Akt), nor the energy-dependent 5′ AMP-activated protein kinase (AMPK) signalling pathway (the level of phospho-T172 AMPKα) (Fig. 5d,e). This observation confirmed the selective inhibitory activity of **21f** toward mTORC1 signalling pathway over mTORC2 or other energy-mediated signalling pathway. In addition, **21f** inhibited the phosphorylation of mTORC1 substrates over 12 h (Fig. 5e), which is similar to Rap, one of the classic selective mTORC1 inhibitors (Supplementary Fig. 18). However, the general inhibitory pattern of **21f** on mTORC1 pathway was quite different from that of Rap, particularly the phosphorylation level of S757 ULK1, which provides the clue that **21f** has a different mode of action from that of Rap via specific inhibition of LRS–RagD interaction. At the same time, the gradual loss of **21f**'s activity might be caused by the negative feedback loops in the mTORC1 signalling pathway^39^ or metabolic clearance of **21f** in the cellular system. The subsequent biophysical study using SPR revealed the binding affinity patterns of **21f** and other pyrimidodiazepine-containing scaffolds (**14f** and **19f**) towards purified LRS. We clearly observed the 1:1 binding event in the SPR sensorgrams of **21f** with *K*_D_ value of 4.8 (±0.46) μM and the saturation event at the high dosage (Fig. 5f and Supplementary Fig. 19), whereas **14f** and **19f** showed no dose-dependent responses without any specific binding events (Supplementary Fig. 19), which supported that **21f** specifically binds LRS and disrupts the interaction between LRS and RagD.

**Figure 6:**
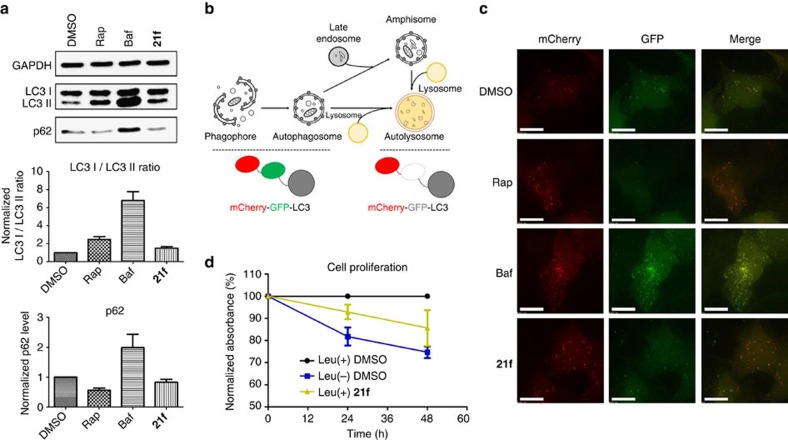
Autophagic activation of **21f** and its reduction on cell proliferation. (**a**) Western blot analysis of LC3 and p62. HeLa cells were treated with 200 nM of rapamycin (Rap), 10 nM of bafilomycin A1 (Baf) or 20 μM of **21f** for 6 h. Quantification of relative band intensity of LC3 I/LC3 II ratio and p62 expression level were normalized with DMSO sample. Data from at least three independent experiments were normalized; error bars represent the s.d. (**b**) Schematic depiction of mCherry-GFP-LC3 system. The pH decreases with lysosome fusion to autophagosome, thereby quenching GFP fluorescence while mCherry fluorescence is maintained. (**c**) mCherry and GFP fluorescence images. HeLa cells were transfected with mCherry-GFP-LC3 plasmid and treated with 200 nM of Rap, 10 nM of Baf or 20 μM of **21f** for 6 h. Scale bar, 15 μm. (**d**) Normalized cellular proliferation level in HEK293T cells was measured by colorimetric BrdU assay under the Leu-deprived media or under the normal media in the absence and presence of **21f** (5 μM). Error bars represent the s.e.m.

As previously mentioned, autophagy is a major catabolic pathway regulated by mTORC1. Therefore, we next examined whether **21f** stimulates cellular autophagy through the inhibition of LRS-mediated activation of mTORC1. Autophagic activity was evaluated using western blot analysis with conversion of LC3 I to LC3 II associated with the formation of autophagosome and the degradation of p62 reflecting autolysosome formation indicating the final progression of the autophagic processes^45^. Bafilomycin A1 (Baf) is a known inhibitor of vacuolar-type H^+^-ATPase, which inhibits the autolysosomal degradation of cellular contents as well as autophagosomal-lysosomal fusion through the inhibition of acidification, thereby blocking the late-stage flux of autophagy^46^. As shown in Fig. 6a, treatment with Baf resulted in an increased ratio of LC3 II/I and accumulation of p62 compared with the DMSO control. In contrast, treatment with Rap or **21f** increased the cellular level of LC3 II and stimulated the degradation of p62, which was consistent with their suppressive effects on mTORC1 activity^47^. To monitor autophagy in living cells, we performed the mCherry-green fluorescent protein (GFP)-LC3 assay^45^,^48^. Because of the difference in acid sensitivity of mCherry (acid-stable) and GFP (acid-sensitive), the entire autophagy process from autophagosome to autolysosome formation could be differentiated by the fluorescence emission pattern (Fig. 6b). For instance, cells expressing mCherry-GFP-LC3 show yellow puncta when autophagosomes are formed. However, the formation of autolysosome via autophagosomal-lysosomal fusion results in red puncta because the acidic environment of autolysosomes quenches GFP fluorescence. As shown in Fig. 6c and Supplementary Fig. 20, yellow puncta was clearly observed upon treatment with Baf, which is consistent with the inhibitory effect of Baf on autophagosomal-lysosomal fusion. When cells were treated with Rap or **21f**, red puncta was clearly observed, which indicates that **21f** could activate autophagy via the suppression of LRS-mediated activation of mTORC1. Finally, we evaluated whether **21f** could inhibit Leu-mediated cell proliferation using 5-bromo-2′-deoxyuridine (BrdU) assay and water-soluble tetrazolium salts assay, because Leu plays a critical role in promoting cellular proliferation through the activation of mTORC1 (ref. 40). As shown in Fig. 6d and Supplementary Fig. 21, **21f** suppressed cell proliferation even in the presence of Leu, similar to in the Leu-deprived condition, which indicated that **21f** could inhibit Leu-mediated signalling to mTORC1 by disrupting the LRS–RagD interaction.

## Discussion

In this study, we developed a new pDOS strategy using pyrimidodiazepine as a privileged substructure. Highly functionalized substrate **1** allowed for the preparation of 16 distinct pyrimidodiazepine- or pyrimidine-containing polyheterocycles through different pairing reactions of five unique reactive sites (A–E) within an average of 2.2 steps. The newly synthesized natural product-like core skeletons with high *sp*^3^ carbon fractions exhibited much higher skeletal diversity than existing pyrimidine-based drugs, as demonstrated by PMI analysis. Considering privileged substructures as ‘chemical navigators' to efficiently access unexploited regions of bioactive chemical space, the pDOS synthetic pathway using pyrimidodiazepine provides unique collections of polyheterocyclic compounds with biological relevancy. Therefore, this pDOS library could play a pivotal role in the perturbation of a wide range of challenging biological targets in a selective and specific manner. To demonstrate this, we performed ELISA-based HTS with this pDOS library and identified **21f**, a new small-molecule PPI inhibitor of the LRS–RagD interaction, which mediates Leu sensing in the mTORC1 signalling pathway. Through a series of biological experiments including western blotting, biophysical study using SPR, live-cell imaging with mCherry-GFP-LC3 and cell proliferation assays, we confirmed that **21f** could selectively inhibit mTORC1 activity, induce cellular autophagy, and reduce cellular proliferation even in the presence of Leu by disrupting the LRS–RagD interaction. Therefore, this PPI inhibitor could be a powerful tool to specifically delineate unrevealed amino acid-dependent biological processes of mTORC1. Furthermore, it could act as a lead structure in the development of novel therapeutic agents to treat diseases linked to the oncogenic activation of mTORC1, thus highlighting the great potential of the pDOS strategy to address unmet therapeutic challenges.

## Methods

### Chemical synthesis

Compounds were synthesized according to Supplementary Methods. X-ray crystallographic image and data of compound **16f** are reported in Supplementary Fig. 5 and Supplementary Tables 1–5, and its cif file was uploaded as Supplementary Data 1 with CCDC number 1500586. Nuclear magnetic resonance spectra (^1^H, ^13^C, one-dimensional or two-dimensional nuclear magnetic resonance) of compounds are shown in Supplementary Figs 23–68.

### PMI Calculation

The outcome of PMI analysis of our pyrimidodiazepine-based pDOS library (Supplementary Fig. 14) was compared with a set of FDA-approved drugs embedded with pyrimidine moieties (Supplementary Fig. 15) as well as with various structurally diverse bioactive natural products (Supplementary Table 6). Energy-minimized 3D structures of the individual compounds were calculated with Materials Studio 4.2 [Accelrys Software Inc.], and visualized by Discovery Studio 3.0. A generalized gradient approximation for the exchange-correlation functional of Becke, Lee, Yang and Parr (BLYP)^49^ was used in conjunction with the double numerical basis set with polarization (DNP) as implemented in DMol3. PMI of the energy-minimized structures were calculated using PreADMET v2.0. After calculating the normalized ratios of the PMI (*I*_*1*_*/I*_*3*_ and *I*_*2*_*/I*_*3*_) of each compounds (Supplementary Table 6), they were plotted as dots in an isosceles triangle defined by vertices (0,1), (0.5,0.5) and (1,1), which correspond to rod, disc, and sphere shapes, respectively, (Fig. 4c and Supplementary Fig. 13).

### Antibodies, plasmids and proteins

Anti-LC3B (ab51520), anti-S6K1 (ab32359), anti-phospho-T389 S6K1 (ab2571) and horseradish peroxidase (HRP) -labelled anti-horse IgG secondary antibodies (ab6802) were purchased from Abcam. Anti-GST (sc-459) was purchased from Santa Cruz Biotechnology. Anti-p62 (CST 5114), anti-glyceraldehyde-3-phosphate dehydrogenase (GAPDH) (CST 2118), anti-phospho-S65 4E-BP1 (CST 9451), anti-phospho-S757 ULK1 (CST 6888), anti-phospho-S473 Akt (CST 4058), anti-phospho-T172 AMPKα (CST 2531) and HRP-labelled anti-rabbit IgG secondary antibodies (CST 7074) were purchased from Cell Signaling Technology. His-tagged LRS and GST-tagged RagD were in laboratory stocks. mCherry-GFP-LC3 plasmid (pBabe vector) was from Dr Heesun Cheong, Division of Chemical Biology, Research Institute, National Cancer Center, Korea.

### Cell culture

Human embryonic kidney (HEK)293T, DU145, Ca Ski and HeLa cells were obtained from American Type Culture Collection (ATCC, VA, USA). HEK293T cell was cultured in DMEM with 10% (v/v) fetal bovine serum and 1% (v/v) antibiotic-antimycotic solution. HeLa, DU145 and Ca Ski cells were cultured in RPMI 1640 medium with 10% (v/v) fetal bovine serum and 1% antibiotic-antimycotic solution. Both cells were maintained in 100 mm cell culture dish in an incubator at 37 °C, in a humidified atmosphere with 5% CO_2_.

### ELISA

His-tagged human LRS were diluted in carbonate buffer (100 mM, pH 9.6) at the concentration of 0.5 ng μl^−1^. Solution was distributed to the half-bottom 96-well clear plate from CORNING 3690. After incubation overnight at 4 °C (sealed), coating solution from each well was removed and washed for three times with PBS with 0.05% Tween 20 (PBST). 5% bovine serum albumin in PBS solution was treated to each well for blocking step, followed by the treatment of each compound and GST-tagged human RagD simultaneously for 3 h. GST protein itself was treated as negative control. Diluted GST antibody (1:1,000) in PBST was added and incubated at room temperature for 1 h. After washing with PBST, the HRP-conjugated anti-horse IgG secondary antibody diluent (1:5,000) was treated and incubated at room temperature for 1 h. TMB was added to each well for colorimetric development. Blue colour should be developed in positive wells. To stop the colour reaction, 1M H_3_PO_4_ stopping solution was added. Finally, absorbance at 450 nm were measured.

### Western blotting

Cells were lysed with radio-immunoprecipitation assay buffer (50 mM Tris, pH 7.8, 150 mM NaCl, 0.5% deoxycholate, 1% IGEPAL CA-630, protease inhibitor cocktail and phosphatase inhibitor). Protein was obtained after centrifugation at 15,000 r.p.m. for 20 min, by transferring supernatant. Protein concentration was normalized with Micro BCA protein assay kit. Overall protein sampling procedure was done at 4 °C. Prepared protein samples were analysed with SDS–polyacrylamide gel electrophoresis and following western blot procedure. Protein was transferred into nitrocellulose membrane after SDS–polyacrylamide gel electrophoresis experiment. Membrane was blocked with 2% bovine serum albumin in TBST over 1 h at room temperature. Primary antibodies were treated overnight at 4 °C (anti-LC3B (ab51520); 1:1,000, anti-S6K1 (ab32359); 1:1,000, anti-phsopho-T389 S6K1 (ab2571); 1:800, anti-p62 (CST 5114); 1:1,000, anti-glyceraldehyde-3-phosphate dehydrogenase (GAPDH) (CST 2118); 1:1,000, anti-phospho-S65 4E-BP1 (CST 9451); 1:1,000, anti-phospho-S757 ULK1 (CST 6888); 1:800, anti-phospho-S473 Akt (CST 4058); 1:1,000 andanti-phospho-T172 AMPKα (CST 2531; 1:1,000), followed by washing with TBST. HRP-labelled anti-rabbit IgG secondary antibodies (1:5,000) were treated at room temperature for 1 h. Antibodies were treated with the concentration indicated in antibody manufacturer's protocol. After washing with TBST, membrane was developed by Amersham ECL prime solution. Chemiluminescent signal was measured by ChemiDoc MP imaging system.

### Surface plasmon resonance (SPR) assay

The dissociation rate constant (*K*_D_) towards His-LRS was determined by SPR technique at the National Center for Inter-university Research Facilities in Seoul National University using a Biacore T100 instrument from GE Healthcare. The carboxyl group on the surface of CM5 sensor chip was replaced with reactive succinimide ester using combination of 1-ethyl-3-(3-dimethylaminopropyl)-carbodiimide and N-hydroxysuccinimide (NHS) in flow cells 1 and 2. Human LRS (1.5 × PBS, pH 7.3) were immobilized on the flow cell 2 (aimed RU; 12,000) through formation of amide bond by reacting with the resulting NHS ester. The remaining NHS ester on flow cells 1 and 2 was quenched by injection of 1 M ethanolamine-HCl (pH 8.0) solution. During the immobilization process, PBS was used as running buffer. After the immobilization of LRS, compounds were injected for 60 s at a flow rate of 20 μl min^−1^ in various concentration from 1 to 15 μM. At the same flow rate, dissociation of compounds from the sensor surface was monitored for 200 s. As a running buffer, 1 × PBS (pH 7.3) containing 3% DMSO and 0.005% P20 solution were used. The binding events were measured at 25 °C. Data analysis were done by using Biacore T100 Evaluation software from GE Healthcare. Final sensorgrams were obtained after the elimination of responses from flow cell 1 and buffer-only control. The dissociation constant (*K*_D_) was calculated by fitting the sensorgrams to the 1:1 binding model.

### Transfection

Cells were seeded in chambered coverglass from Nunc, 24 h before transfection. mCherry-GFP-LC3 plasmid was transfected to HeLa cell using Lipofectamine 2000 reagent. Transfection was preceded according to manufacturer's protocol.

### mCherry-GFP-LC3 puncta imaging

DeltaVision Elite imaging system was used for the imaging of mCherry-GFP-LC3 transfected HeLa cell. For live-cell imaging, chamber was maintained at 37 °C, 5% CO_2_ condition. Image was obtained with × 60 scale, using mCherry/mCherry, GFP/GFP (Excitation/Emission) filter sets. mCherry (excitation: 575/25 nm, emission: 625/45 nm); and GFP (excitation: 475/28 nm, emission: 525/48 nm). Images were analysed and merged with SoftWorks deconvolution software.

### Cell proliferation assay

HEK293T cells were seeded in transparent 96-well plate from CORNING. Twenty-four hours after seeding, leucine-free DMEM and compound-DMEM solution (final compound concentration of 5 μM) were treated in each well after the removal of culture media. Cell proliferation assay was done with water-soluble tetrazolium salts assay and Cell proliferation ELISA, BrdU (colorimetric) kit, following the manufacturer's protocol.

### Data availability

The X-ray crystallographic coordinates for structures reported in this study have been deposited at the Cambridge Crystallographic Data Centre (CCDC), under CCDC numbers 1500586. These data can be obtained free of charge from the Cambridge Crystallographic Data Centre via www.ccdc.cam.ac.uk/data_request/cif. All other data is available from the authors upon reasonable request.

## Additional information

**How to cite this article:** Kim, J. *et al*. Diversity-oriented synthetic strategy for developing a chemical modulator of protein*–*protein interaction. *Nat. Commun.* **7,** 13196 doi: 10.1038/ncomms13196 (2016).

## Supplementary Material

Supplementary Information Supplementary Figures 1-68, Supplementary Tables 1-6, Supplementary Methods and Supplementary References

Supplementary Data 1 X-Ray coordinates

## Figures and Tables

**Figure 1:**
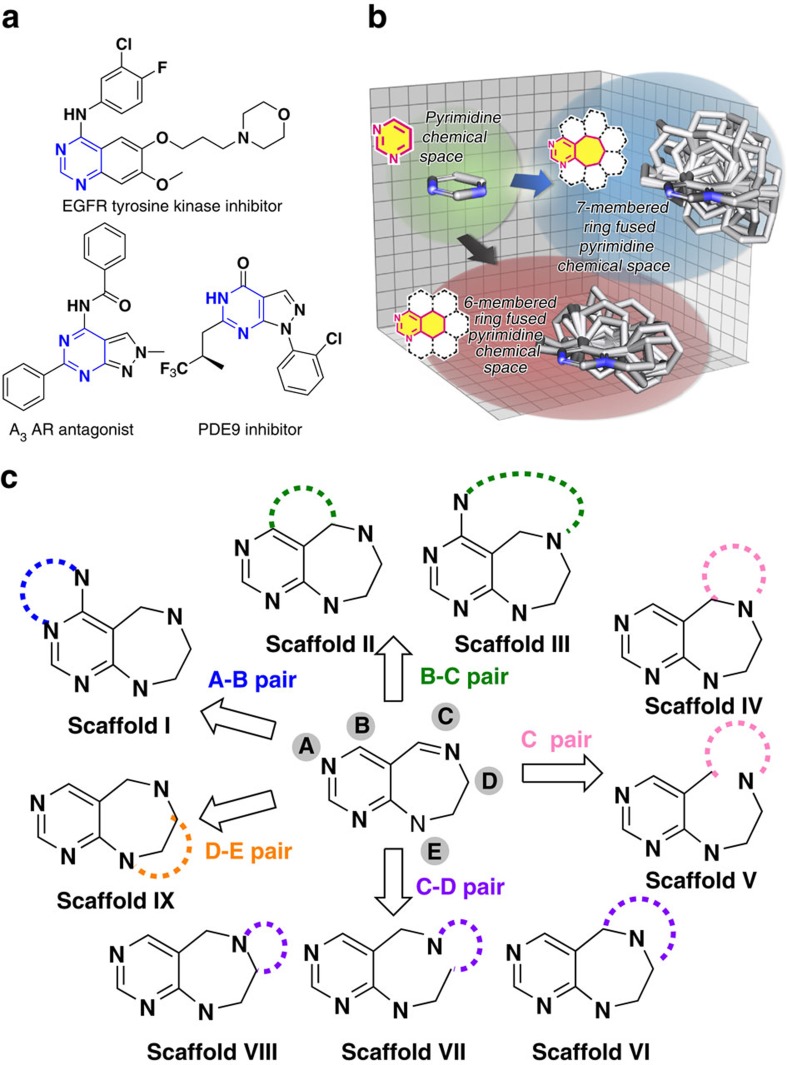
Diversity-oriented synthetic strategy with pyrimidine as a privileged structure. (**a**) Pyrimidine-containing bioactive compounds. (**b**) 3D chemical space of pyrimidine and the comparison between pyrimidine-containing tricyclic 6/6/6 and 6/7/6 systems in terms of 3D diversity and complexity by overlaying energy-minimized conformers aligned along the pyrimidine substructure. (**c**) Synthetic strategy for diversity-oriented synthesis of pyrimidodiazepine- or pyrimidine-containing polyheterocycles through divergent pairing pathways.
