# Supplementary material for: Diversity-oriented synthetic strategy for developing a chemical modulator of protein–protein interaction
Source: Nat Commun. 2016 Oct 24;7:13196. doi: 10.1038/ncomms13196 (PMC5078997; doi:10.1038/ncomms13196)
Supplement: Supplementary Information — Supplementary Figures 1-68, Supplementary Tables 1-6, Supplementary Methods and Supplementary References [file ncomms13196-s1.pdf]

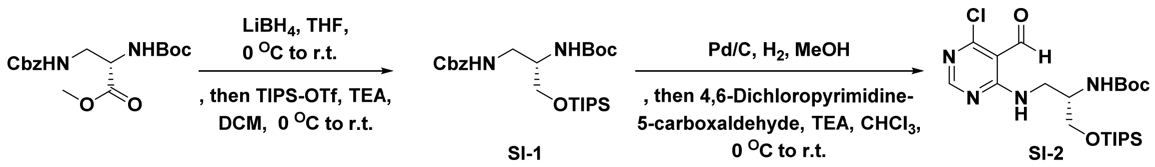

Supplementary Figure 1. Synthetic procedure for the *tert*-butyl (*S*)-(1-((6-chloro-5-formylpyrimidin-4-yl)amino)-3-((triisopropylsilyl)oxy)propan-2-yl)carbamate (**SI-2**)

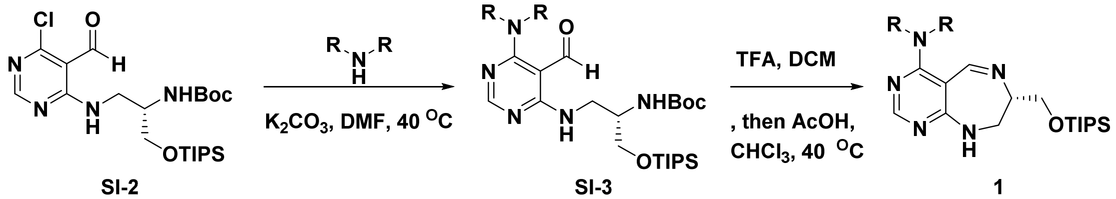

Supplementary Figure 2. General procedure for the preparation of **1a**–**c** and **1e**–**f**.

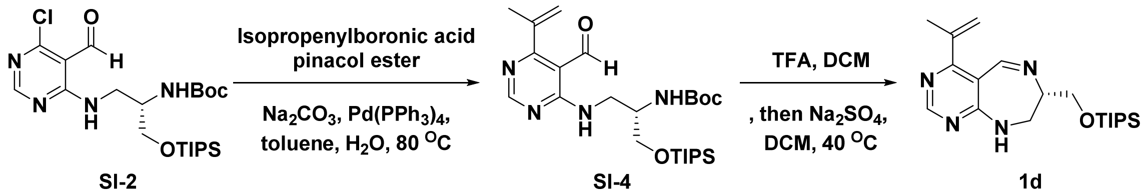

Supplementary Figure 3. Synthetic procedure for the preparation of (*S*)-4-(prop-1-en-2-yl)-7-(((triisopropylsilyl)oxy)methyl)-8,9-dihydro-7H-pyrimido[4,5-e][1,4]diazepine (**1d**).

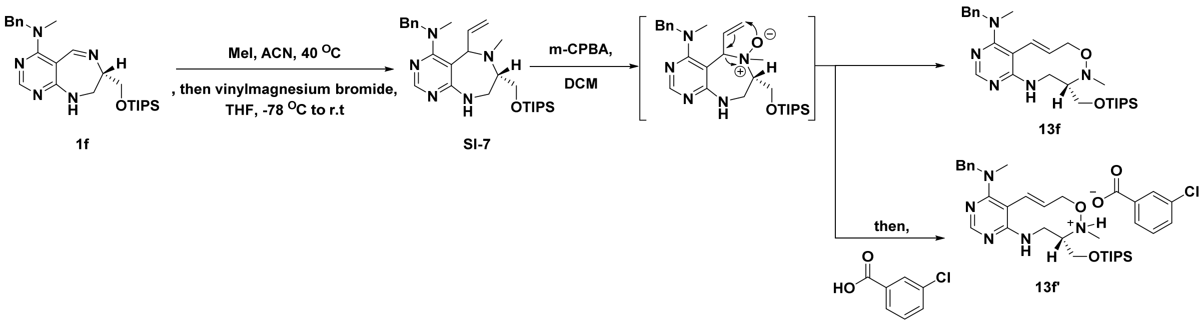

Supplementary Figure 4. Synthetic procedure for the preparation of **13f** and **13f´**.

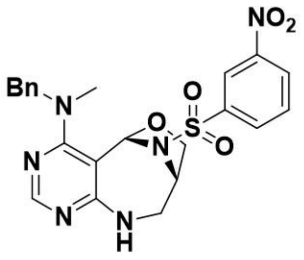

16f

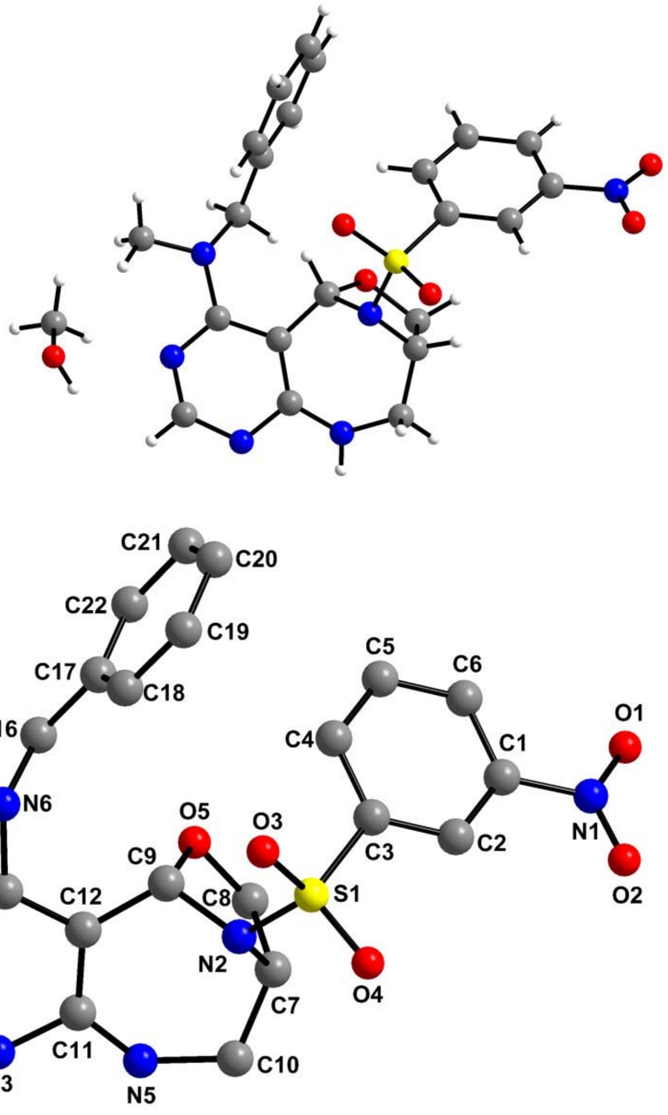

Supplementary Figure 5. X-ray structure of **16f**. Deposition number in Cambridge Structural Database: CCDC1500586.

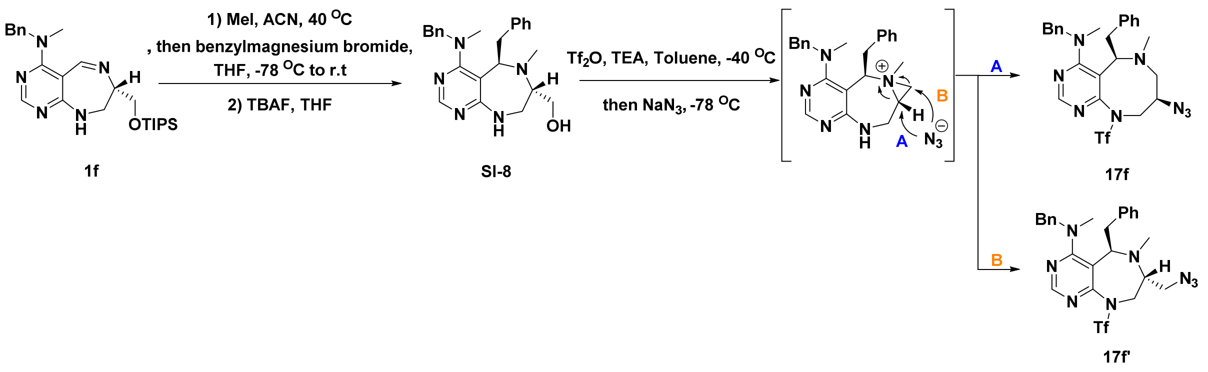

Supplementary Figure 6. Synthetic procedure for the preparation of **17f** and **17f´**.

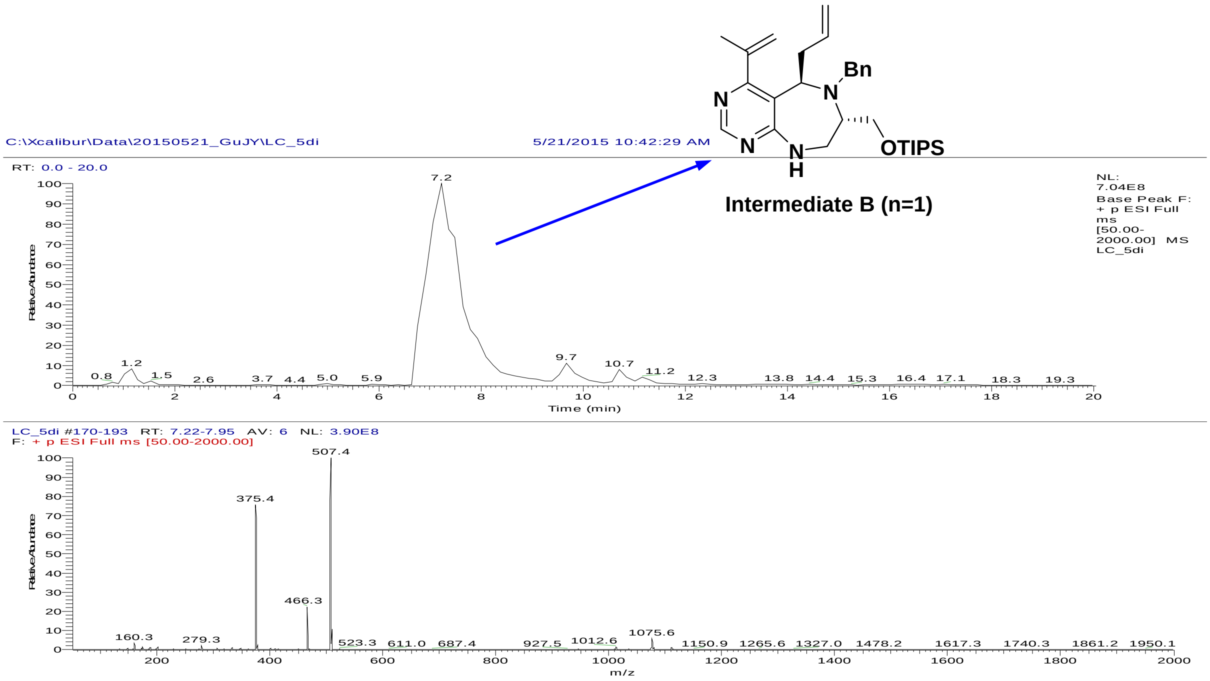

Supplementary Figure 7. LC-MS analysis of the crude reaction mixtures (**Intermediate B** (n=1)) for the determination of diastereomeric ratio (d.r. >99:1).

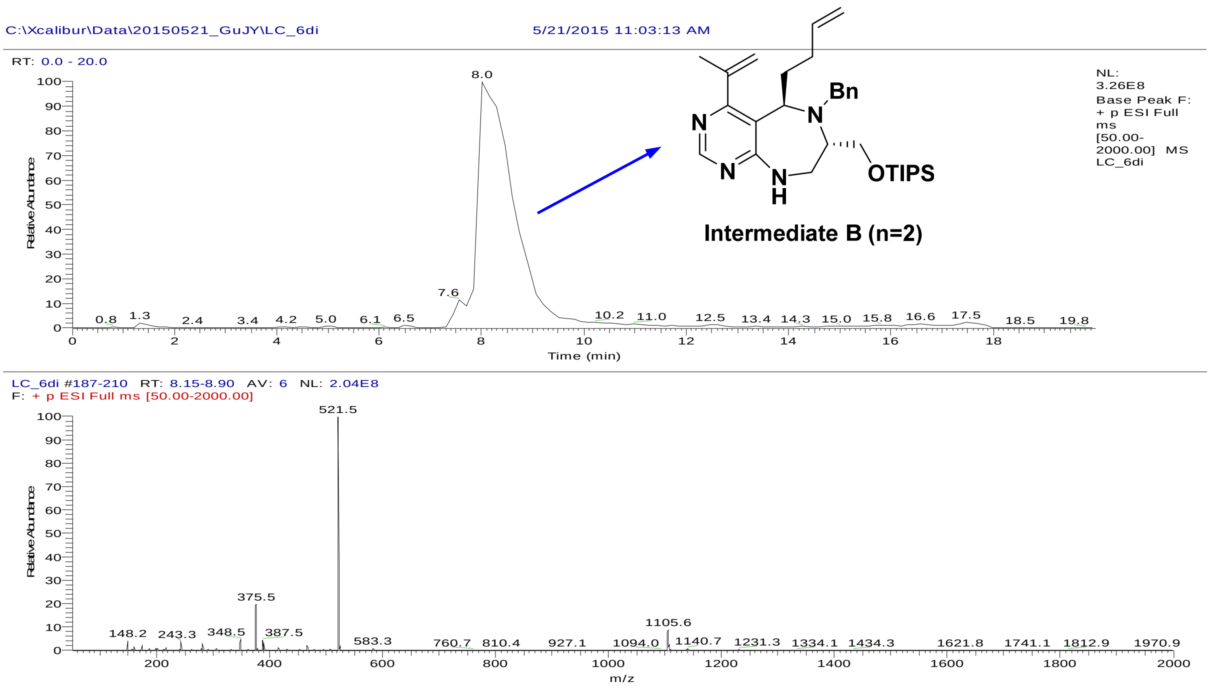

Supplementary Figure 8. LC-MS analysis of the crude reaction mixtures (**Intermediate B** (n=2)) for the determination of diastereomeric ratio (d.r. >99:1).

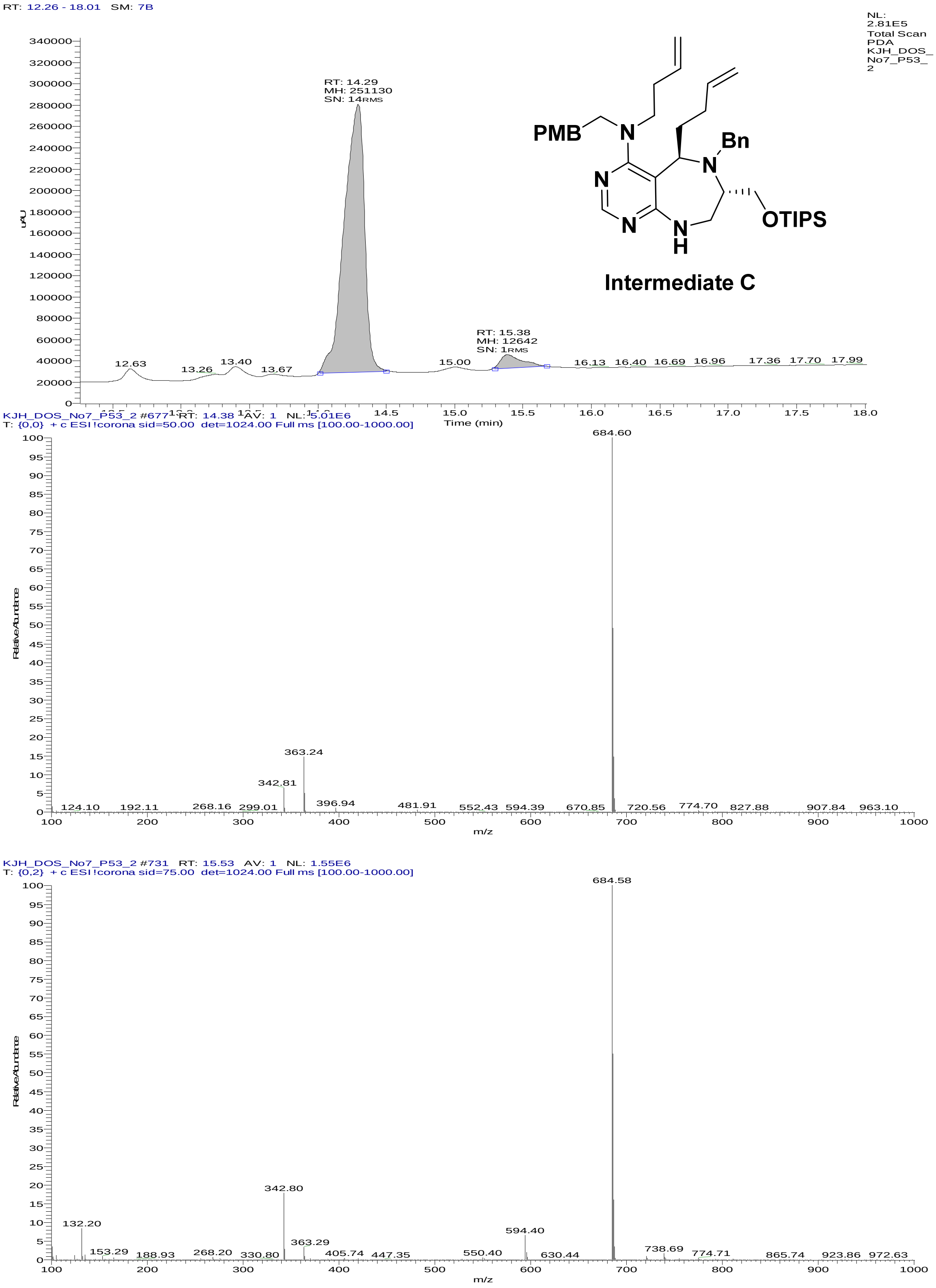

Supplementary Figure 9. LC-MS analysis of the crude reaction mixtures (**Intermediate C**) for the determination of diastereomeric ratio (d.r. 95:5).

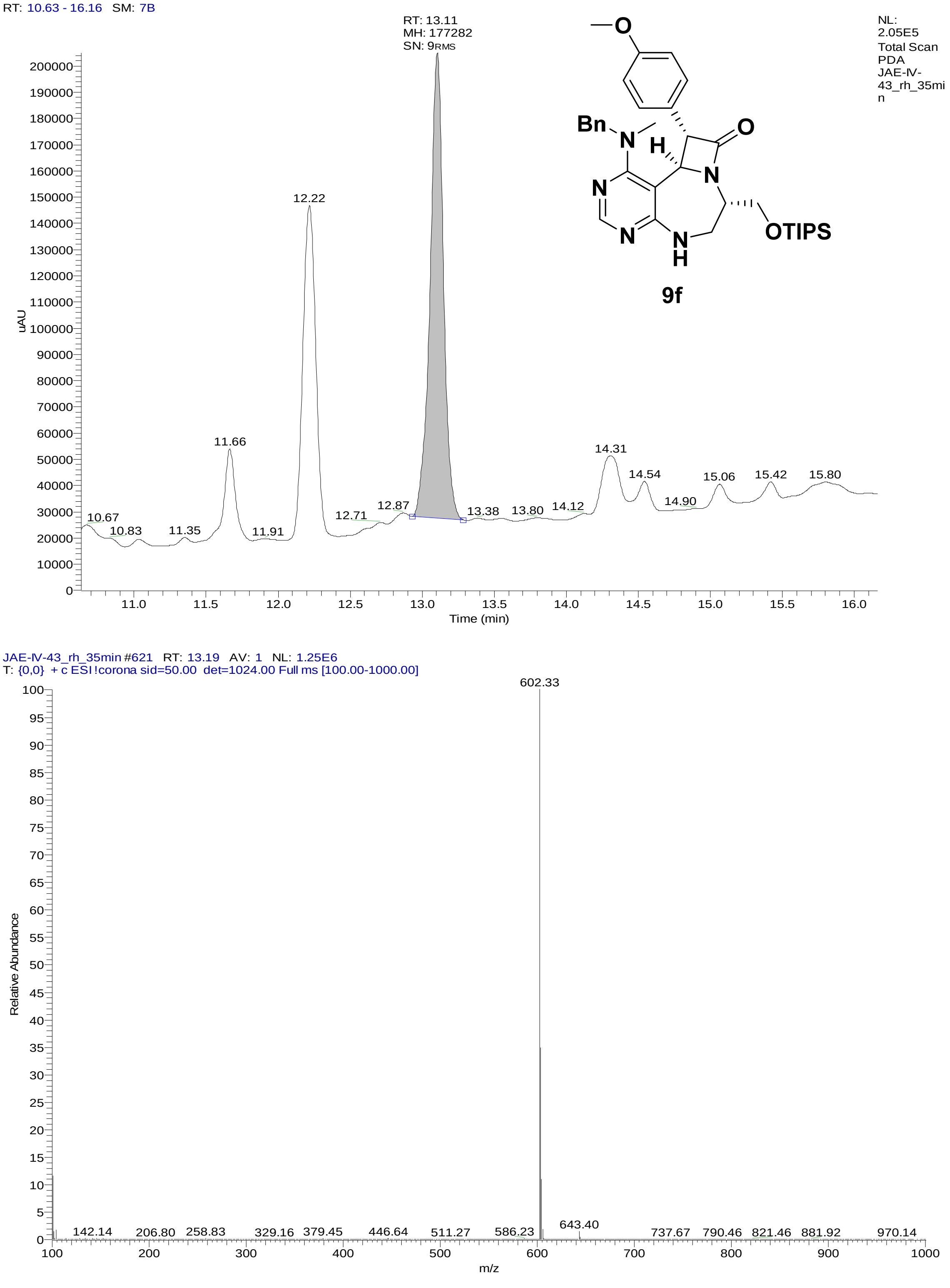

Supplementary Figure 10. LC-MS analysis of the crude reaction mixtures (**9f**) for the determination of diastereomeric ratio (d.r. >99:1).

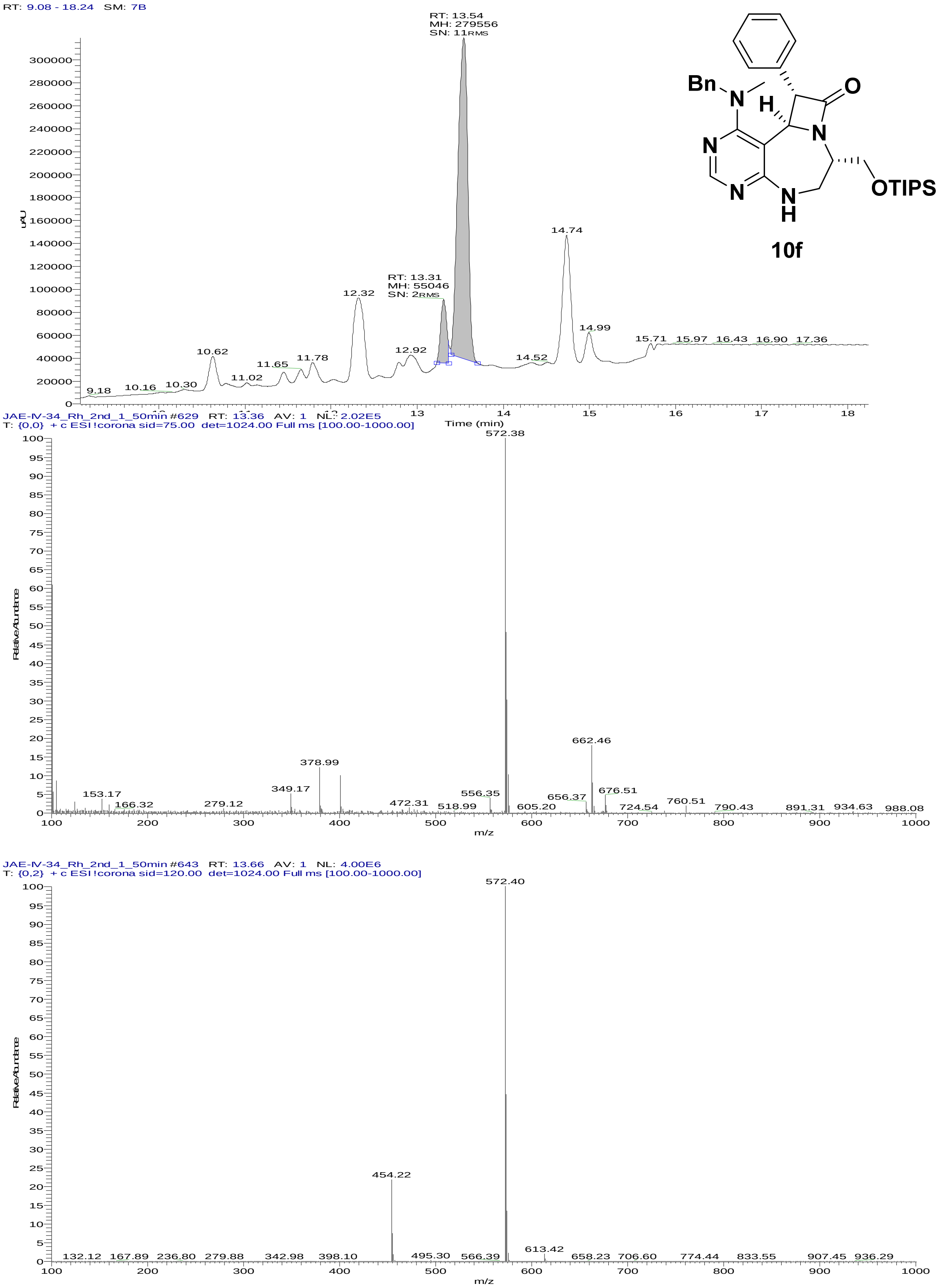

Supplementary Figure 11. LC-MS analysis of the crude reaction mixtures (**10f**) for the determination of diastereomeric ratio (d.r. 90:10).

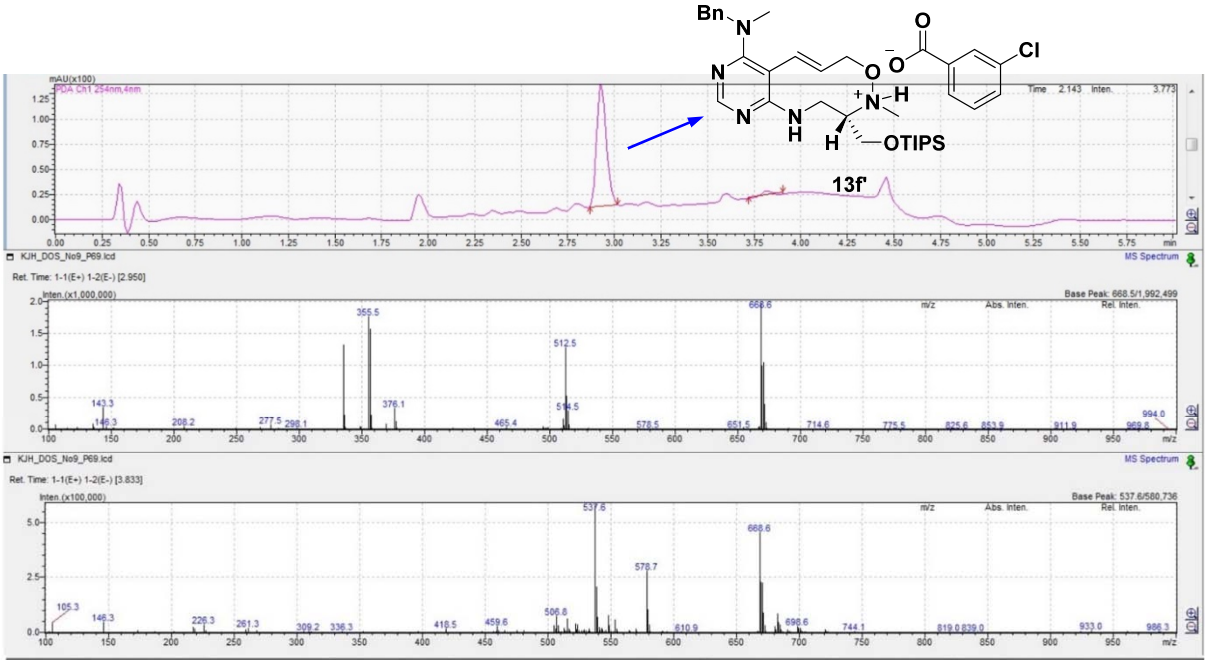

Peak#	Ret. Time	Area	Height	Mark	Conc.	Unit	ID#	Name	Area%
1	2.928	442082	119842	M	0.000				99.033
2	3.818	4317	3373	M	0.000				0.967
Total		446399	123215		0.000				100.000

Supplementary Figure 12. LC-MS analysis of the crude reaction mixtures (**13f´**) for the determination of E/Z ratio (99:1).

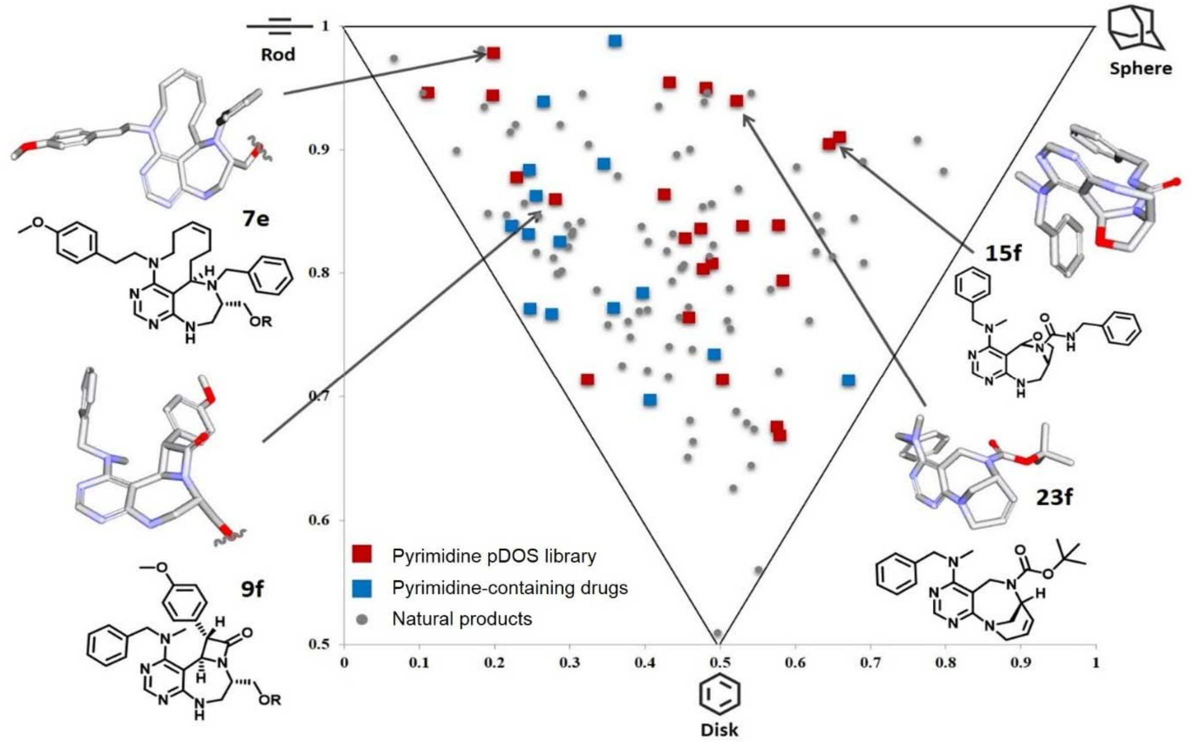

Supplementary Figure 13. Principal moment of inertia (PMI) plot.

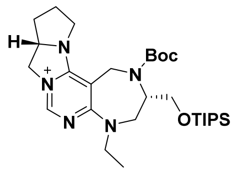
2a

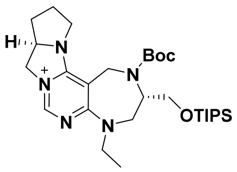
3b

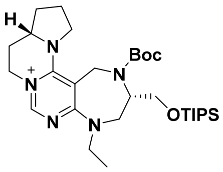
4c

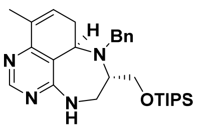
5d

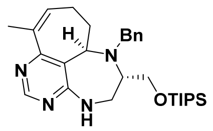
6d

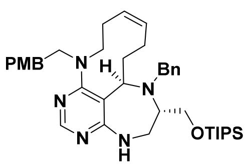
7e

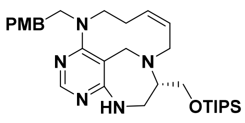
8e

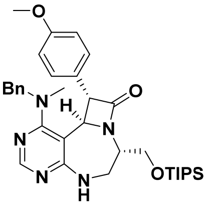
9f

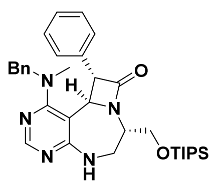
10f

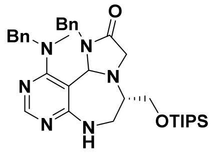
11f

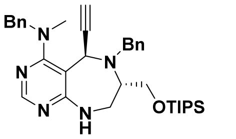
12f

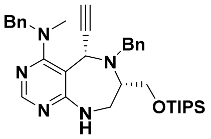
12f'

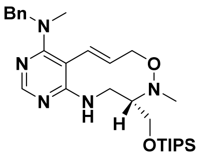
13f

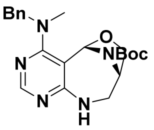
14f

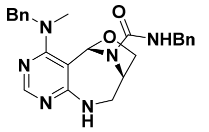
15f

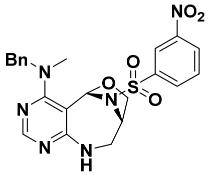
16f

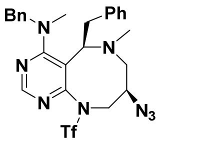
17f

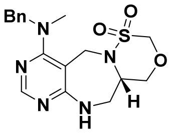
18f

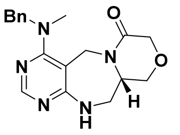
19f

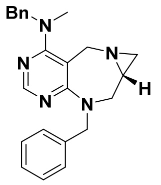
20f

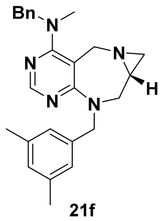
21f

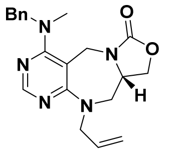
22f

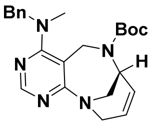
23f

Supplementary Figure 14. Complete pDOS Library used in PMI analysis

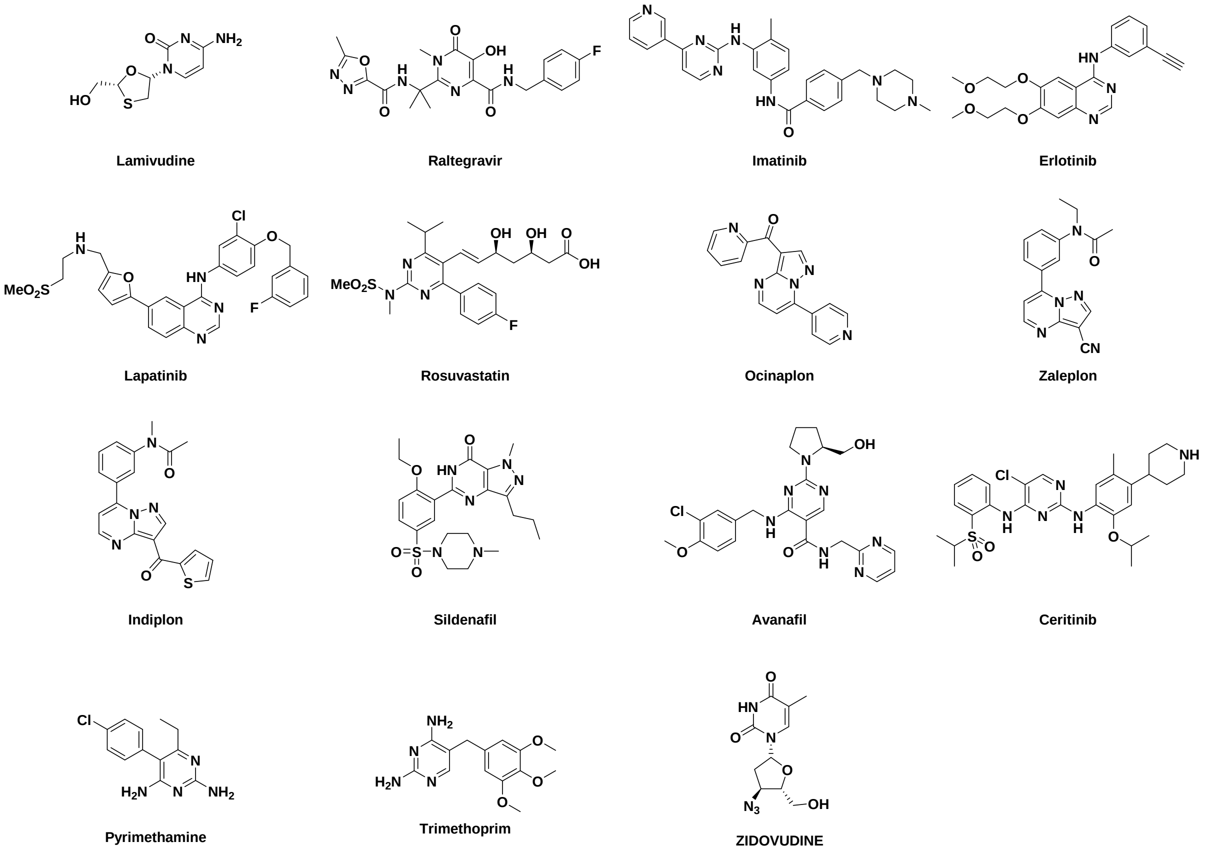

Supplementary Figure 15. FDA-approved drugs embedded with pyrimidine moieties.[1]

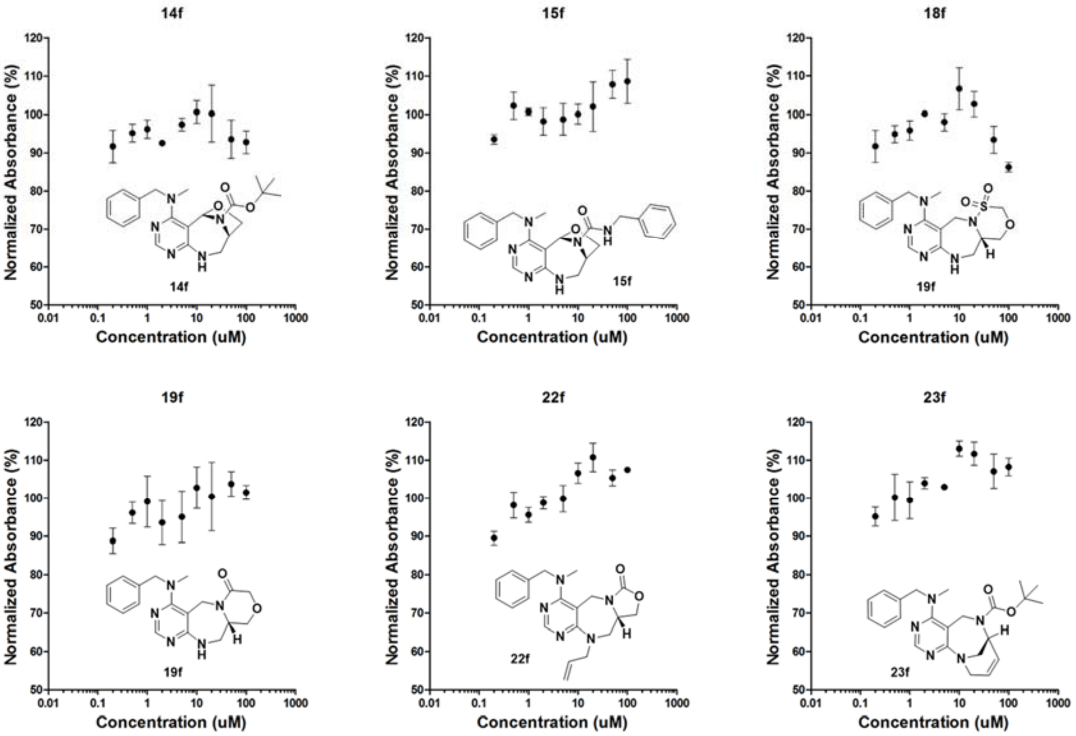

Supplementary Figure 16. Dose-response curves in ELISA with **14f**, **15f**, **18f**, **19f**, **22f** and **23f**.

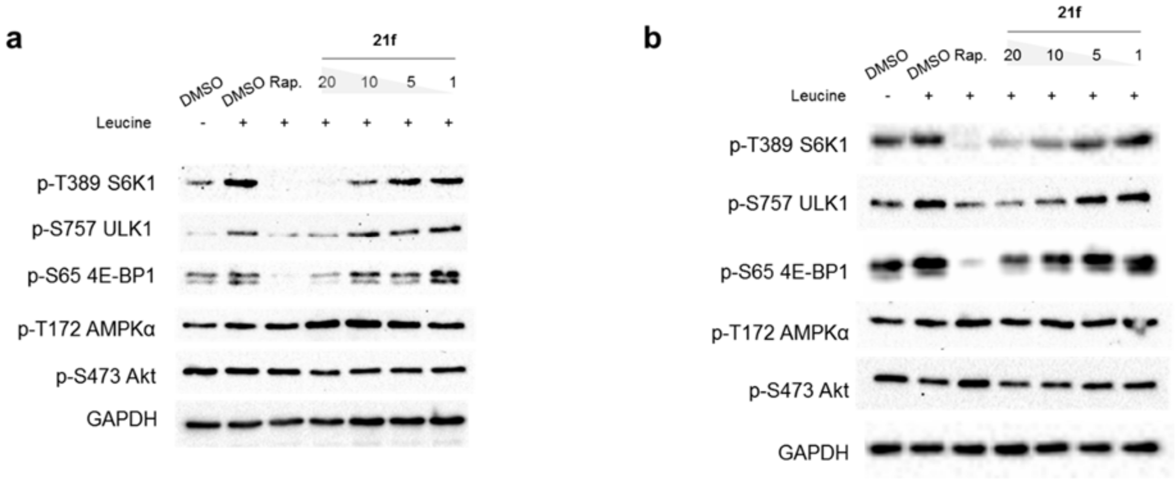

Supplementary Figure 17. Effects of **21f** on mTORC1 signaling pathway. a) Ca Ski or b) DU145 Cells were treated with 20, 10, 5, 1 uM of **21f** or 200 nM of Rapamycin for 3 h in complete media condition. For negative control, 293T cells were starved for leucine for 3 h. Phospho-T389 S6K1, phospho-S757 ULK1, phospho-S65 4E-BP1, phospho-T172 AMPKα, phospho-S473 Akt and GAPDH were determined by western blot.

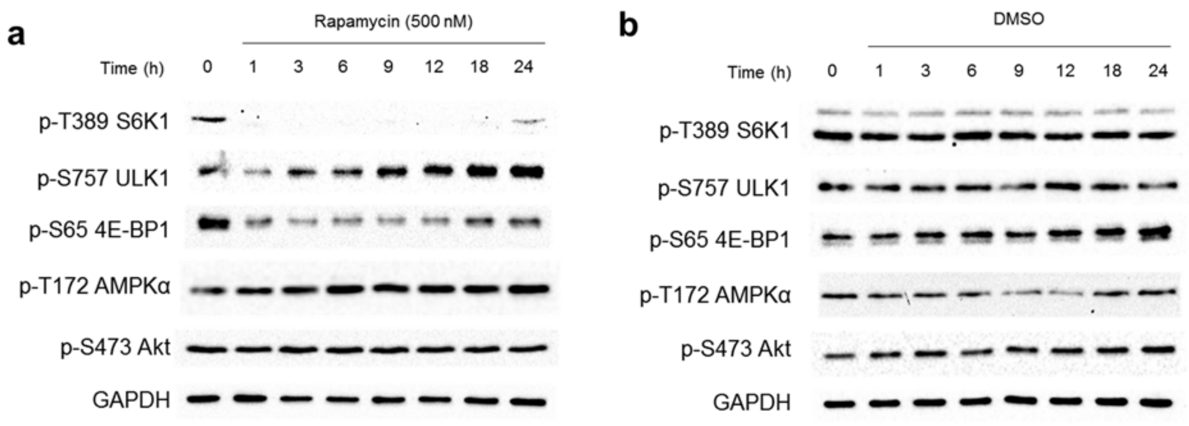

Supplementary Figure 18. Effects of **21f** on mTORC1 signaling pathway. HEK293T cells were treated with a) 500 nM Rapamycin or b) DMSO for specified time in complete media. Phospho-T389 S6K1, phospho-S757 ULK1, phospho-S65 4E-BP1, phospho-T172 AMPKα, phospho-S473 Akt and GAPDH were determined by western blot.

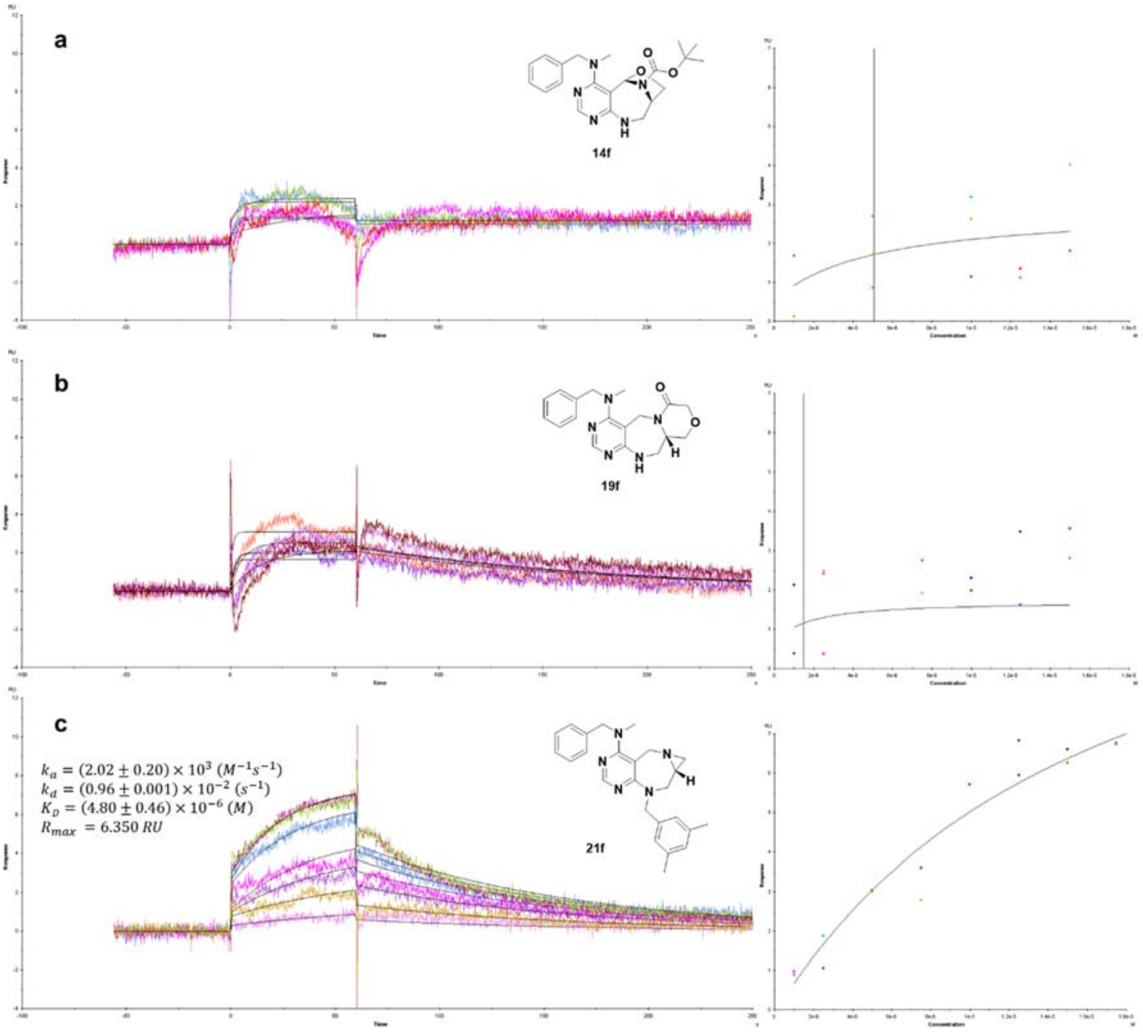

Supplementary Figure 19. SPR curves showing concentration-dependent binding to purified LRS. Normalized RUs are plotted over a time course. a) SPR curves with **14f** with 1, 5, 10, 12.5 and 15 μM concentrations. b) SPR curves with **19f** with 1, 2.5, 7.5, 10, 12.5 and 15 μM concentrations. c) SPR curves with **21f** with 1, 2.5, 5, 10, 12.5, 15, 17.5 and 20 μM concentrations.

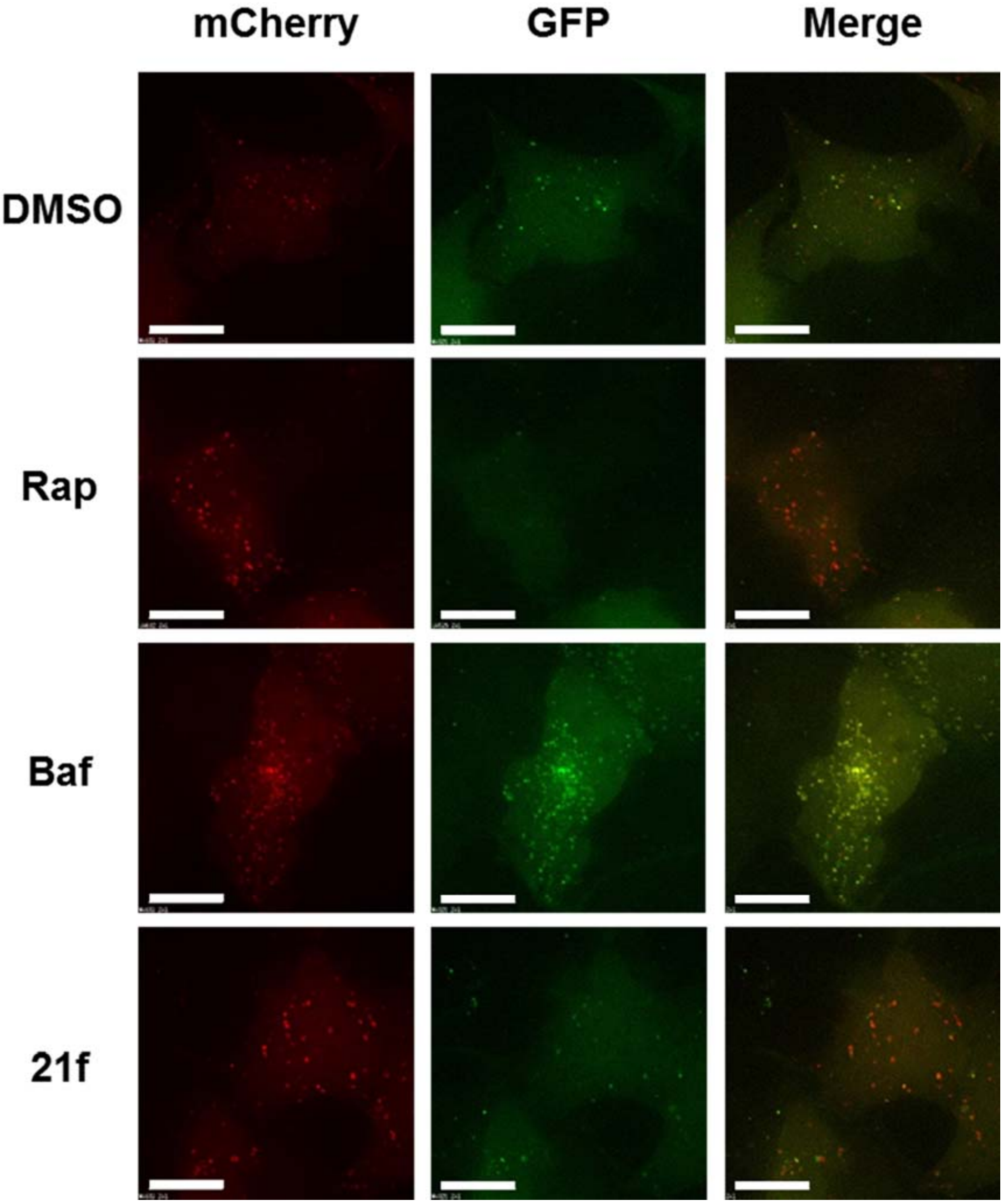

Supplementary Figure 20. mCherry and GFP fluorescence images. HeLa cells were transfected with mCherry-GFP-LC3 plasmid and treated with 200 nM of Rap, 10 nM of Baf or 20 μM of **21f** for 6 h. Scale bar represents 15 μm.

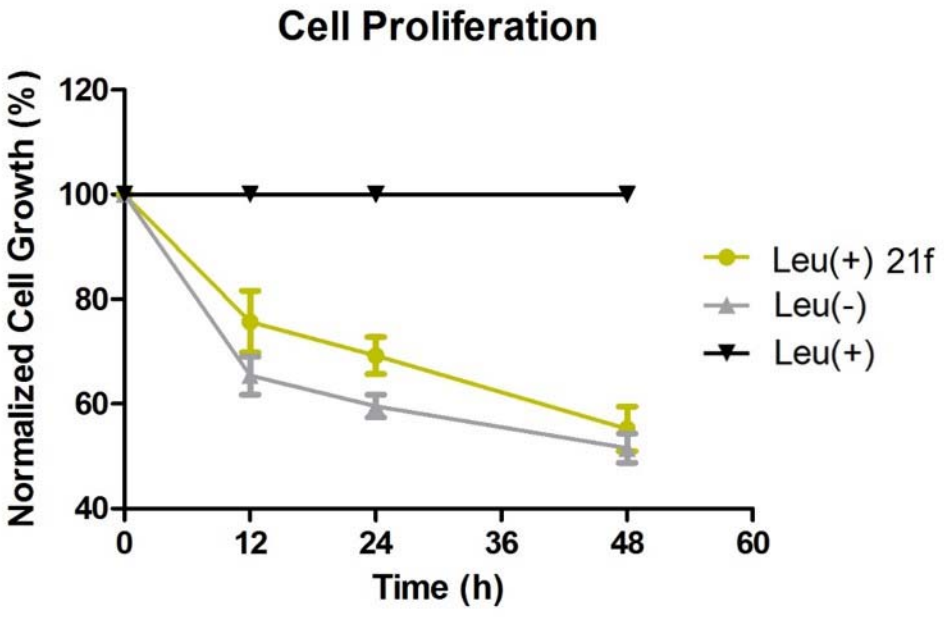

Supplementary Figure 21. Cell proliferation assay. Normalized cellular proliferation level in HEK293T cells was measured by WSTs assay under the Leu-deprived media [Leu(-)] or under the normal media in the absence and presence of 20 μM of **21f**. Error bars represent the s.e.m.

Fig. 5c

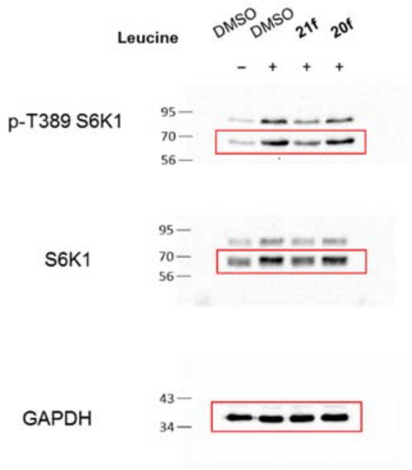

Leucine
DMSO
DMSO
21f
20f
–
+
+
+
p-T389 S6K1
95
70
56
S6K1
95
70
56
GAPDH
43
34

Fig. 5d

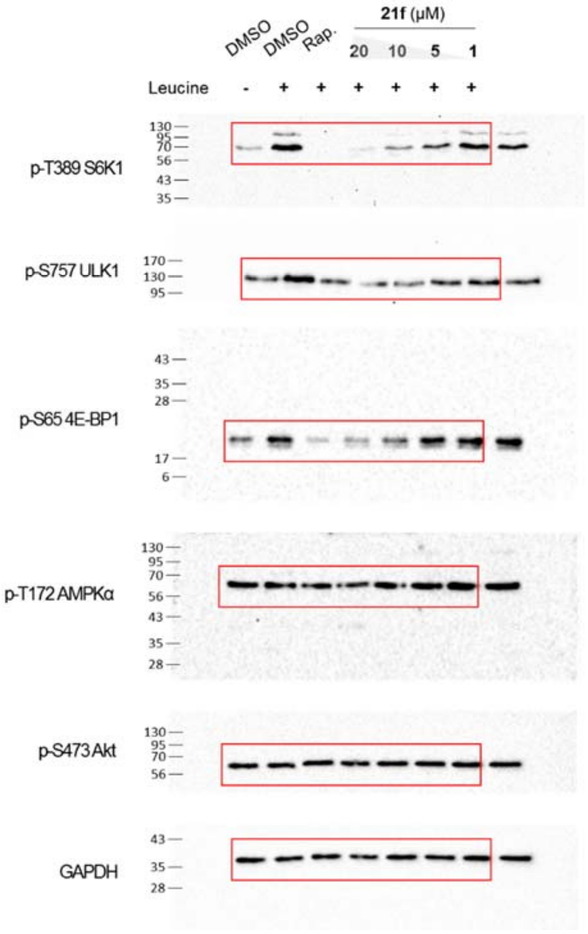

DMSO
DMSO
Rap.
21f (μM)
20
10
5
1
Leucine
-
+
+
+
+
+
+
p-T389 S6K1
130
95
70
56
43
35
p-S757 ULK1
170
130
95
p-S65 4E-BP1
43
35
28
17
6
p-T172 AMPKα
130
95
70
56
43
35
28
p-S473 Akt
130
95
70
56
GAPDH
43
35
28

Fig. 5e

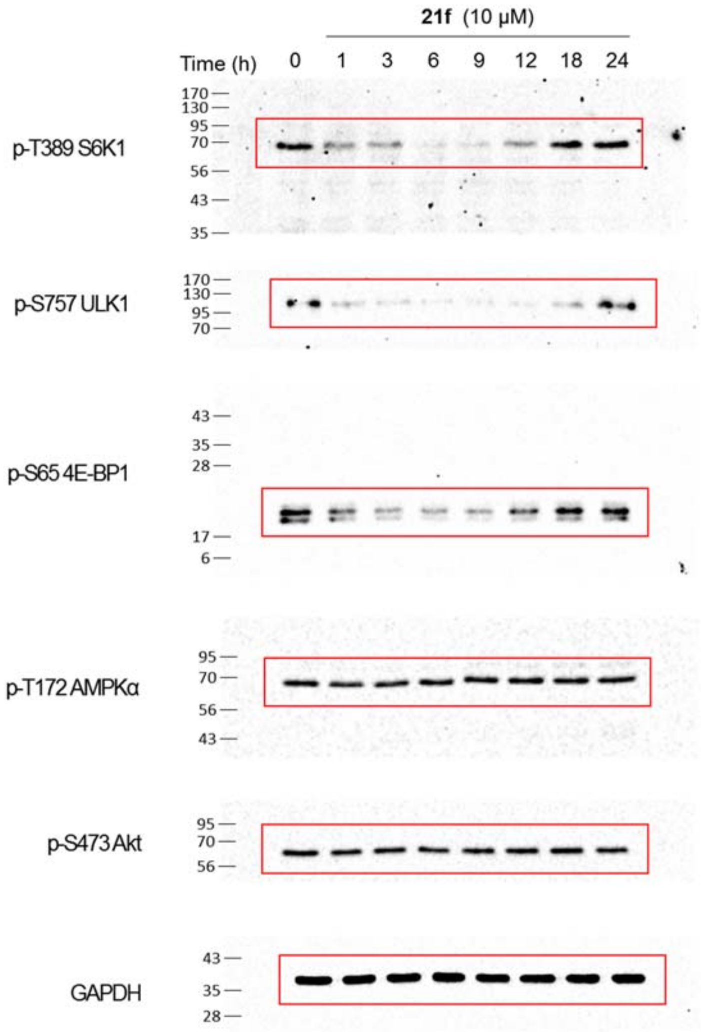

21f (10 μM)
Time (h) 0 1 3 6 9 12 18 24
p-T389 S6K1
p-S757 ULK1
p-S65 4E-BP1
p-T172 AMPKα
p-S473 Akt
GAPDH

Supplementary fig. 17a

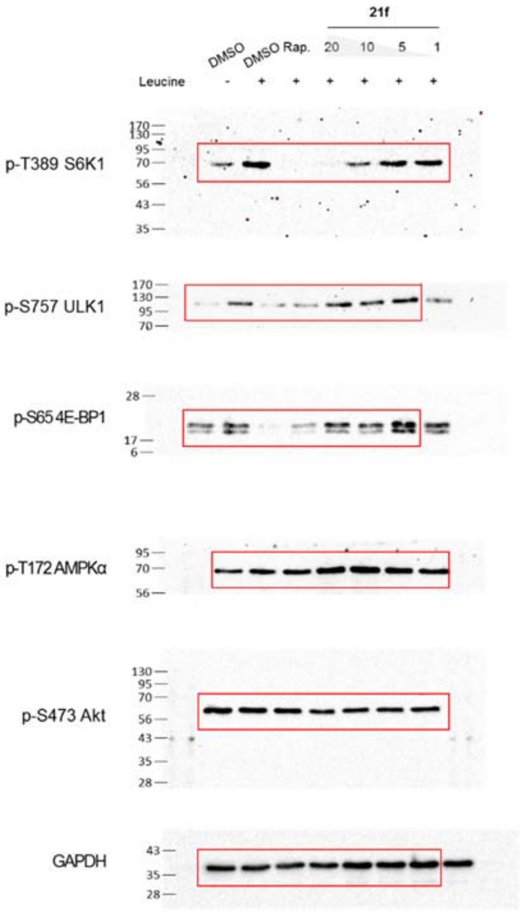

Supplementary fig. 17b

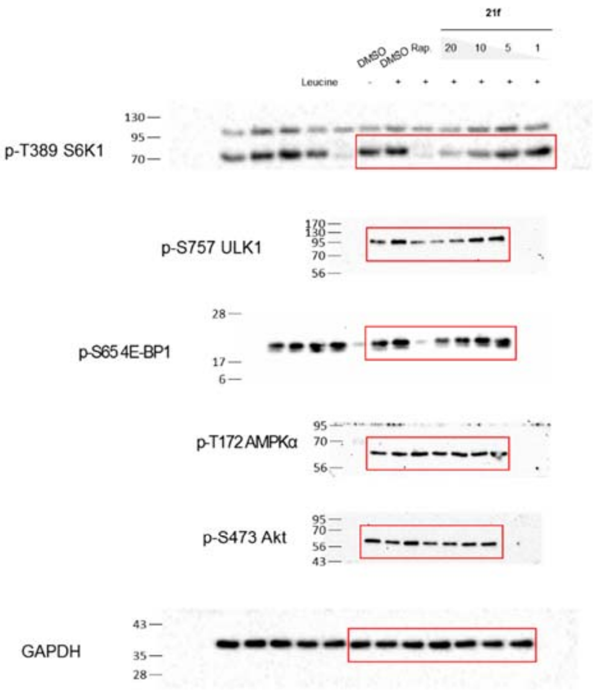

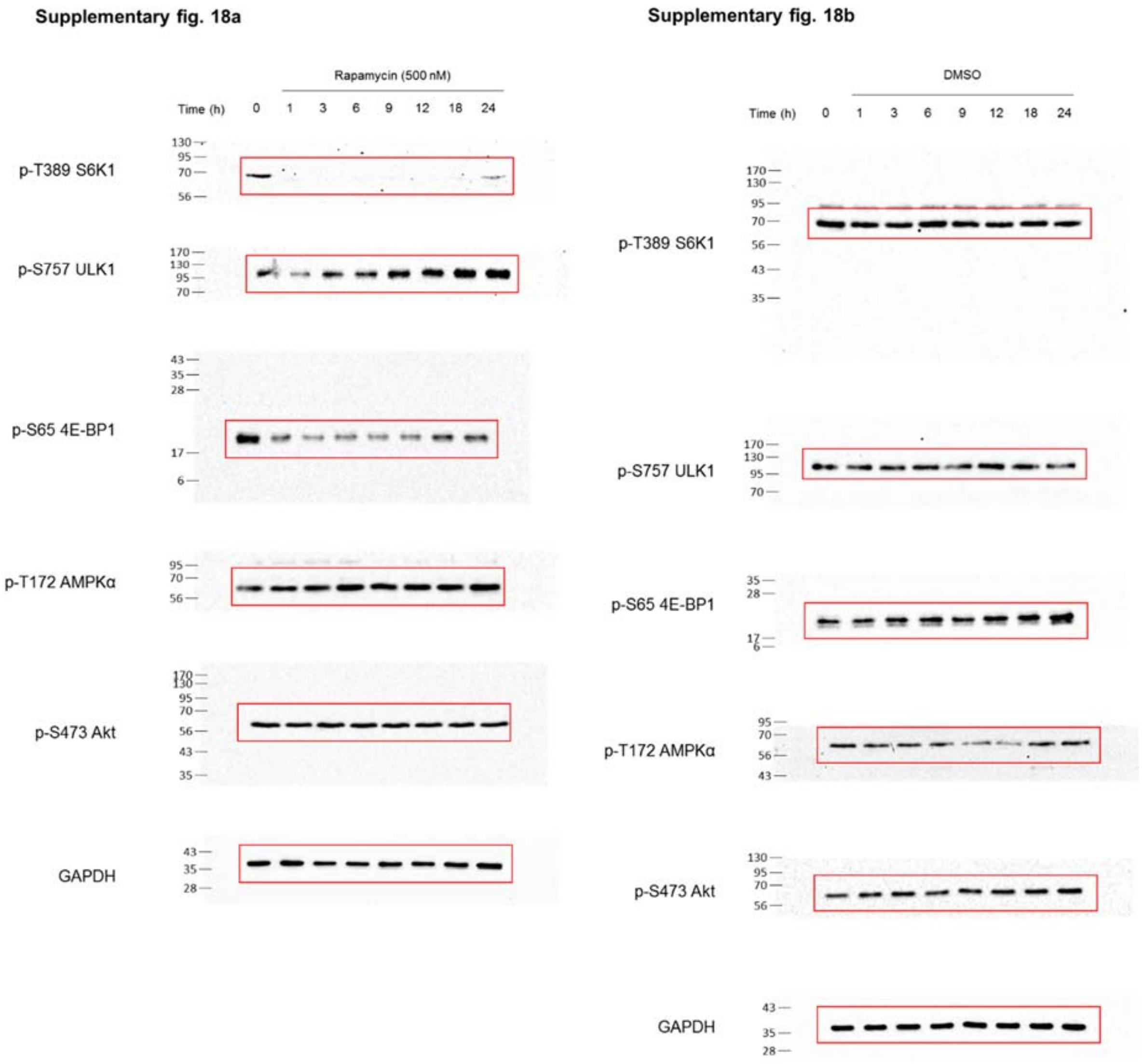

Supplementary Figure 22. Full scans of western blots. The protein of interest being detected is labeled on the left side of each blot.

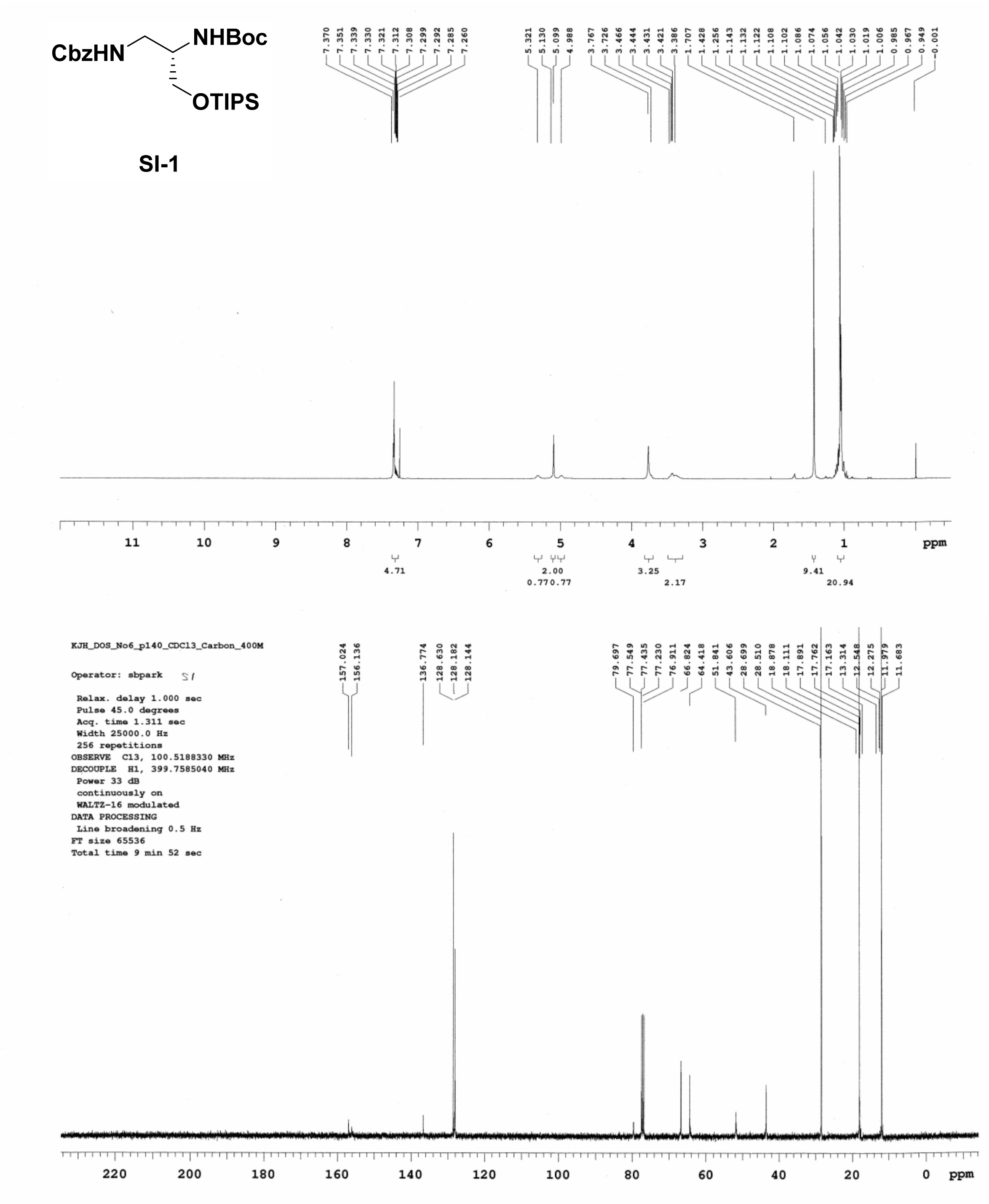

Supplementary Figure 23. ^{1}H and ^{13}C NMR spectra for **SI-1**.

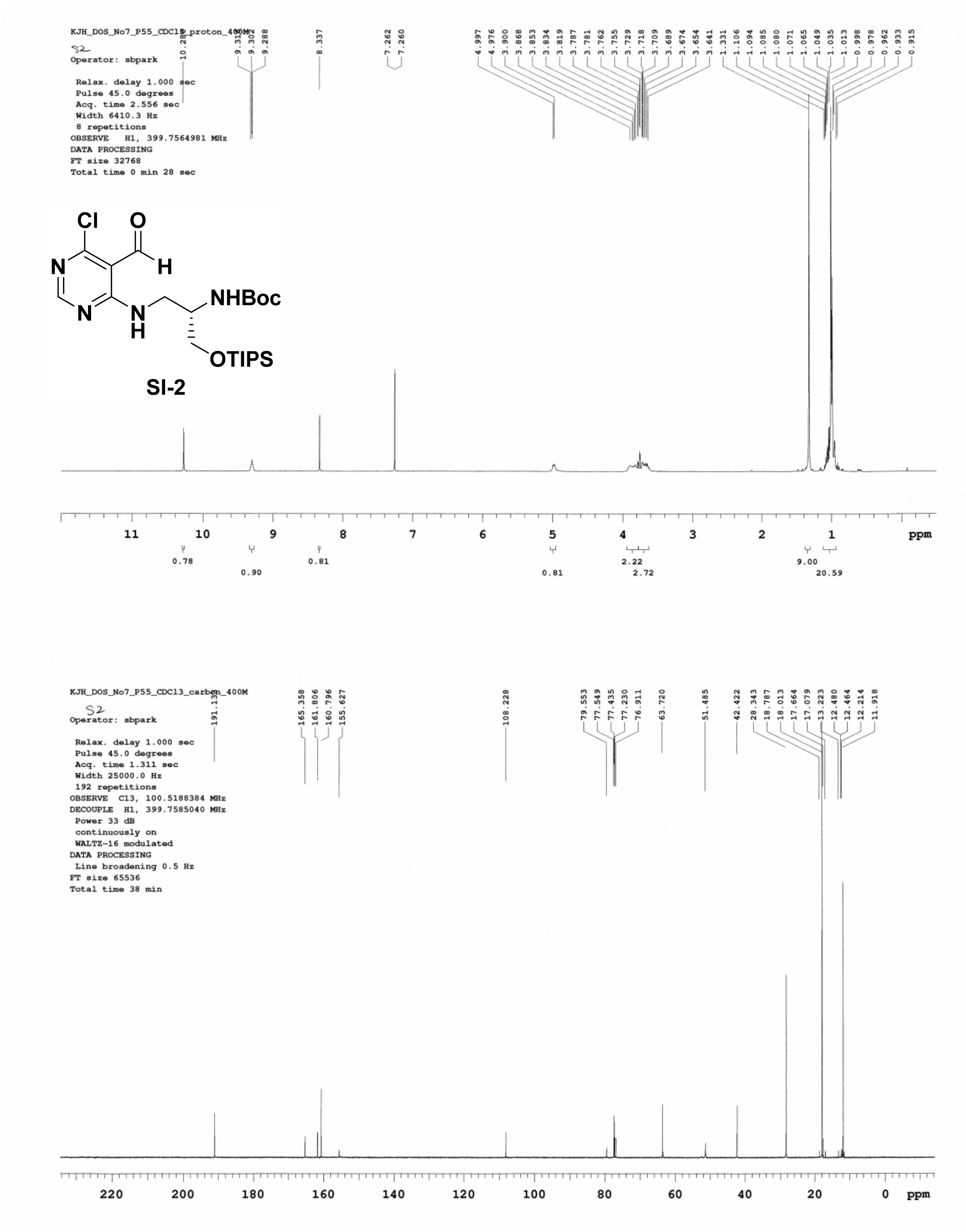

Supplementary Figure 24. ^{1}H and ^{13}C NMR spectra for **SI-2**.

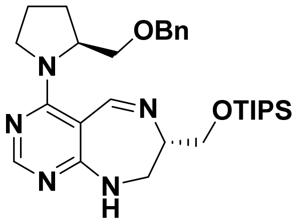

1a

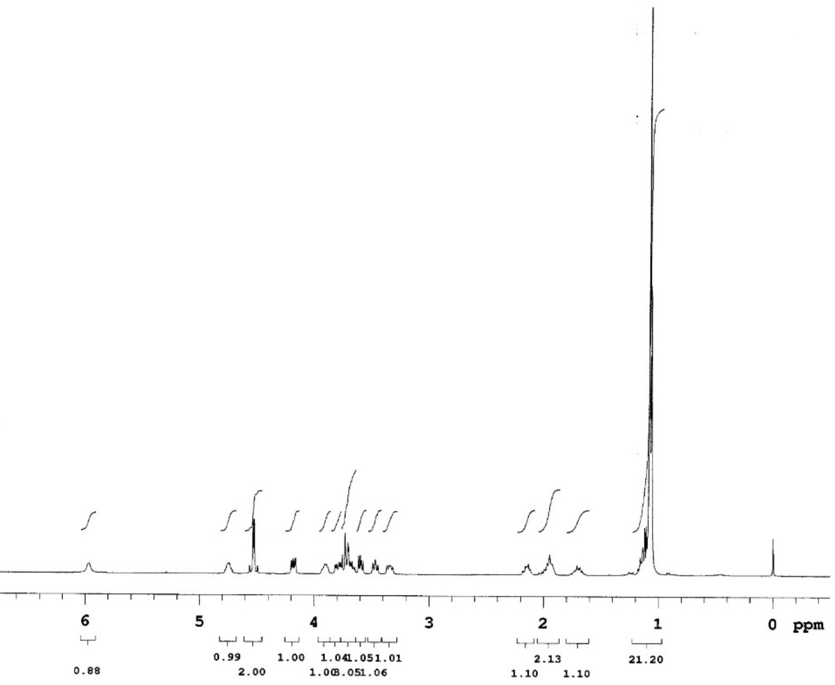

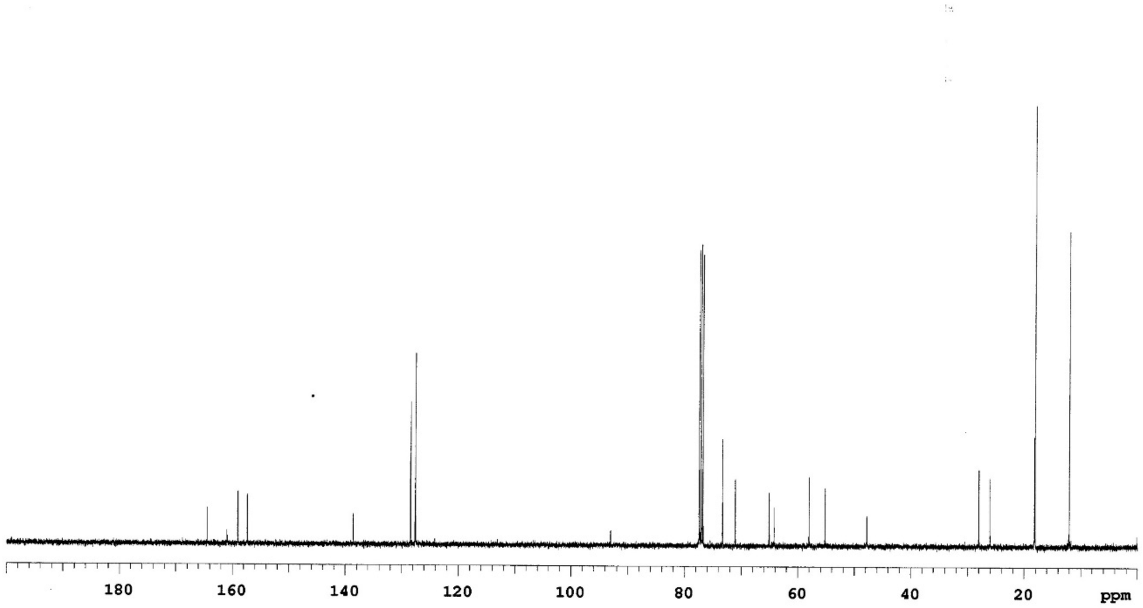

Supplementary Figure 25. ^{1}H and ^{13}C NMR spectra for **1a**.

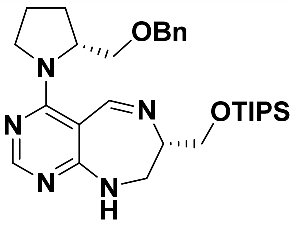

1b

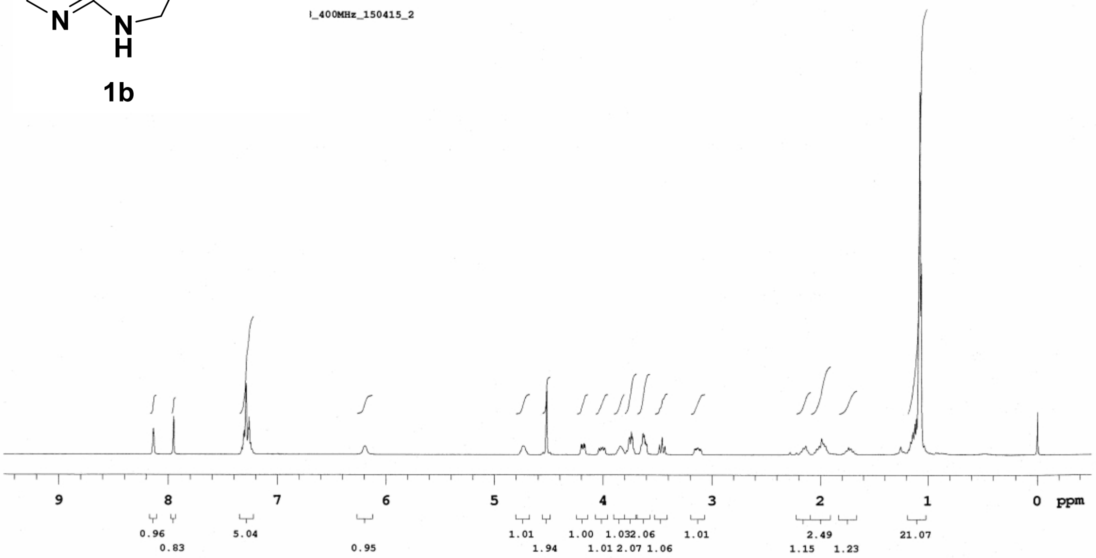

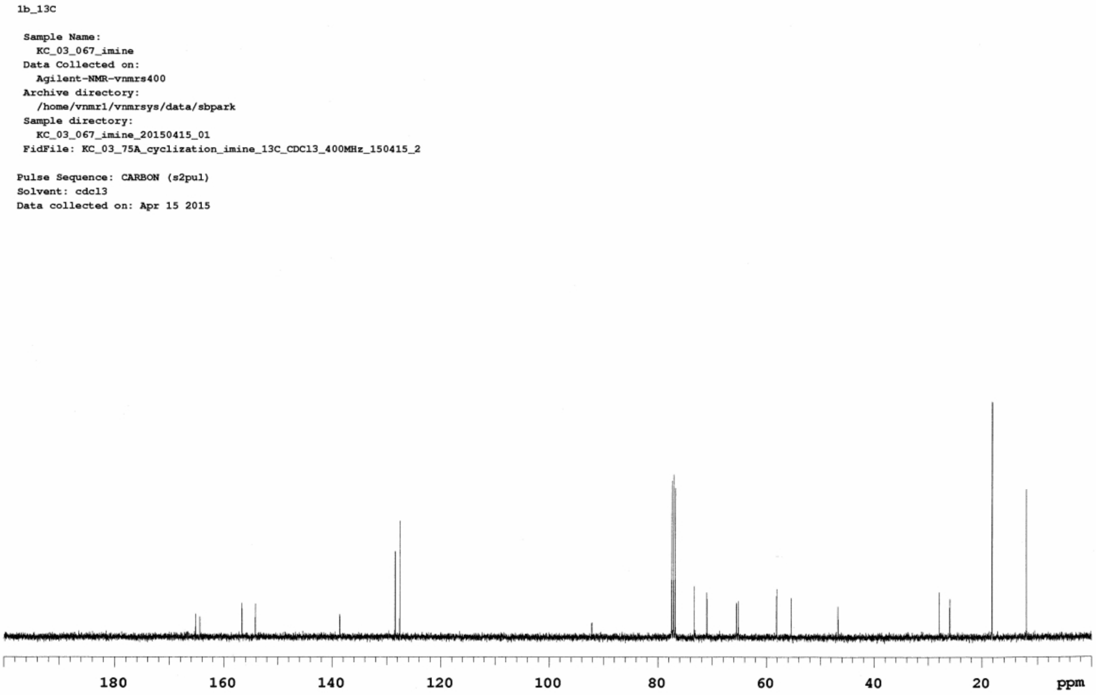

Supplementary Figure 26. ^{1}H and ^{13}C NMR spectra for **1b**.

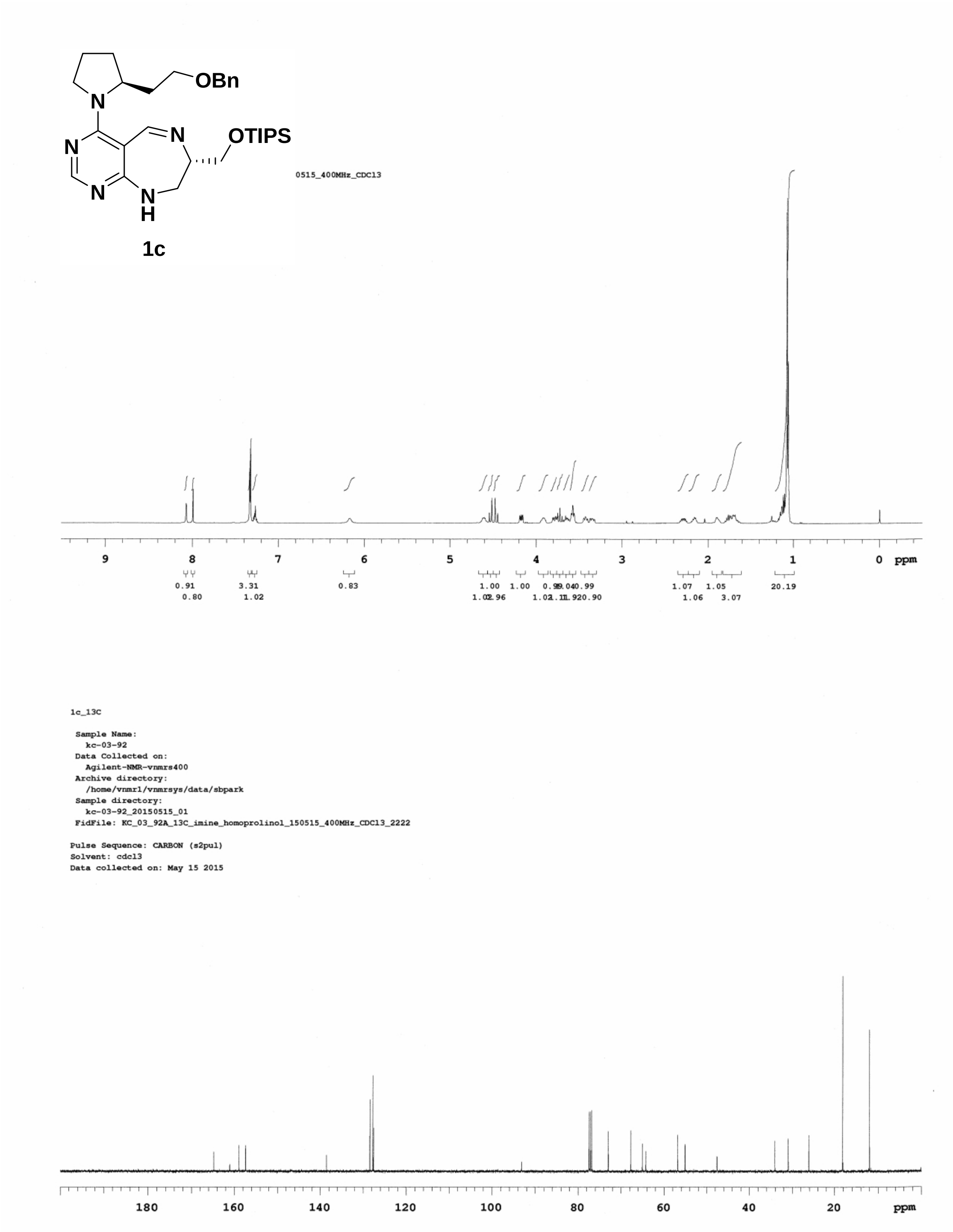

Supplementary Figure 27. ^{1}H and ^{13}C NMR spectra for **1c**.

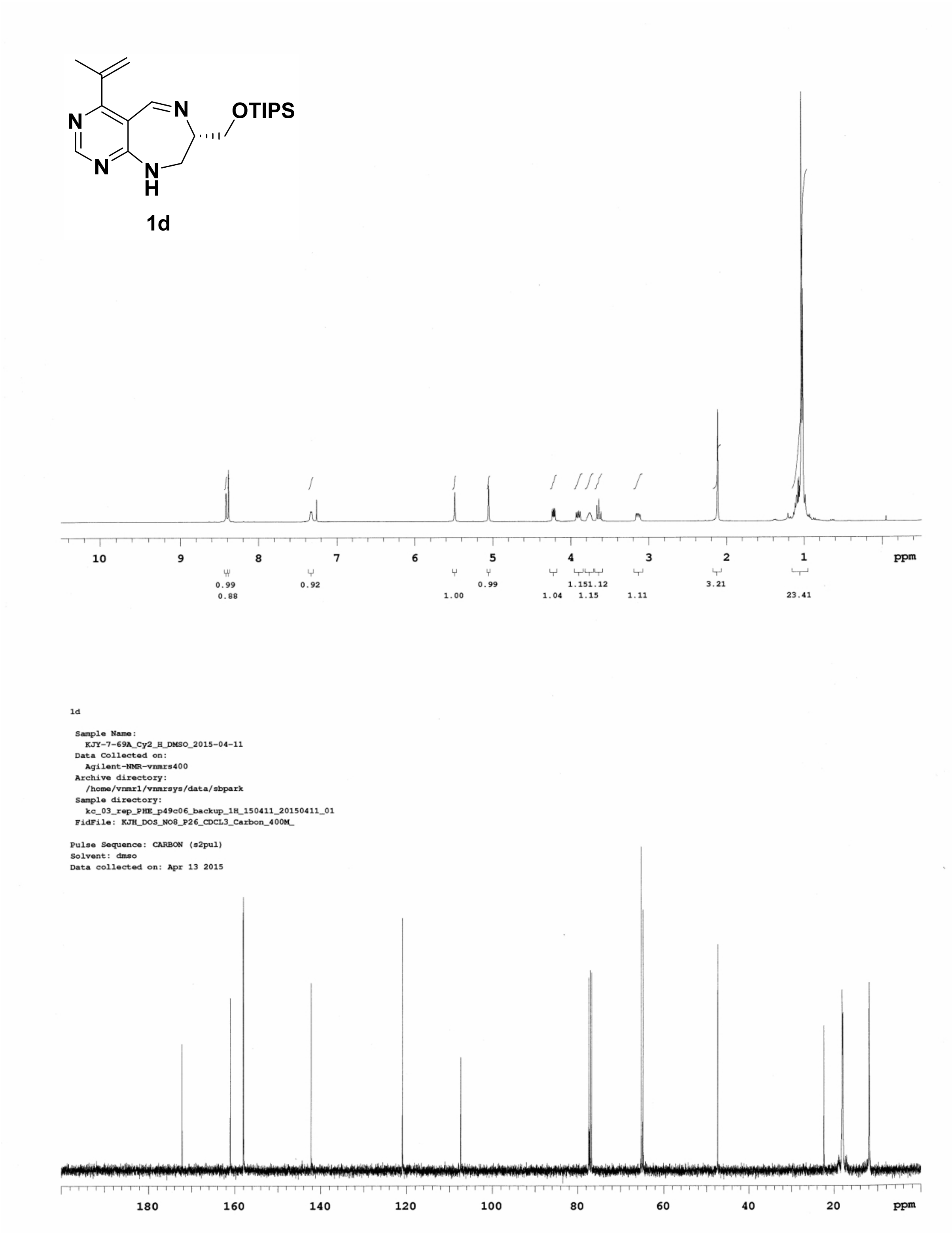

Supplementary Figure 28. ^{1}H and ^{13}C NMR spectra for **1d**.

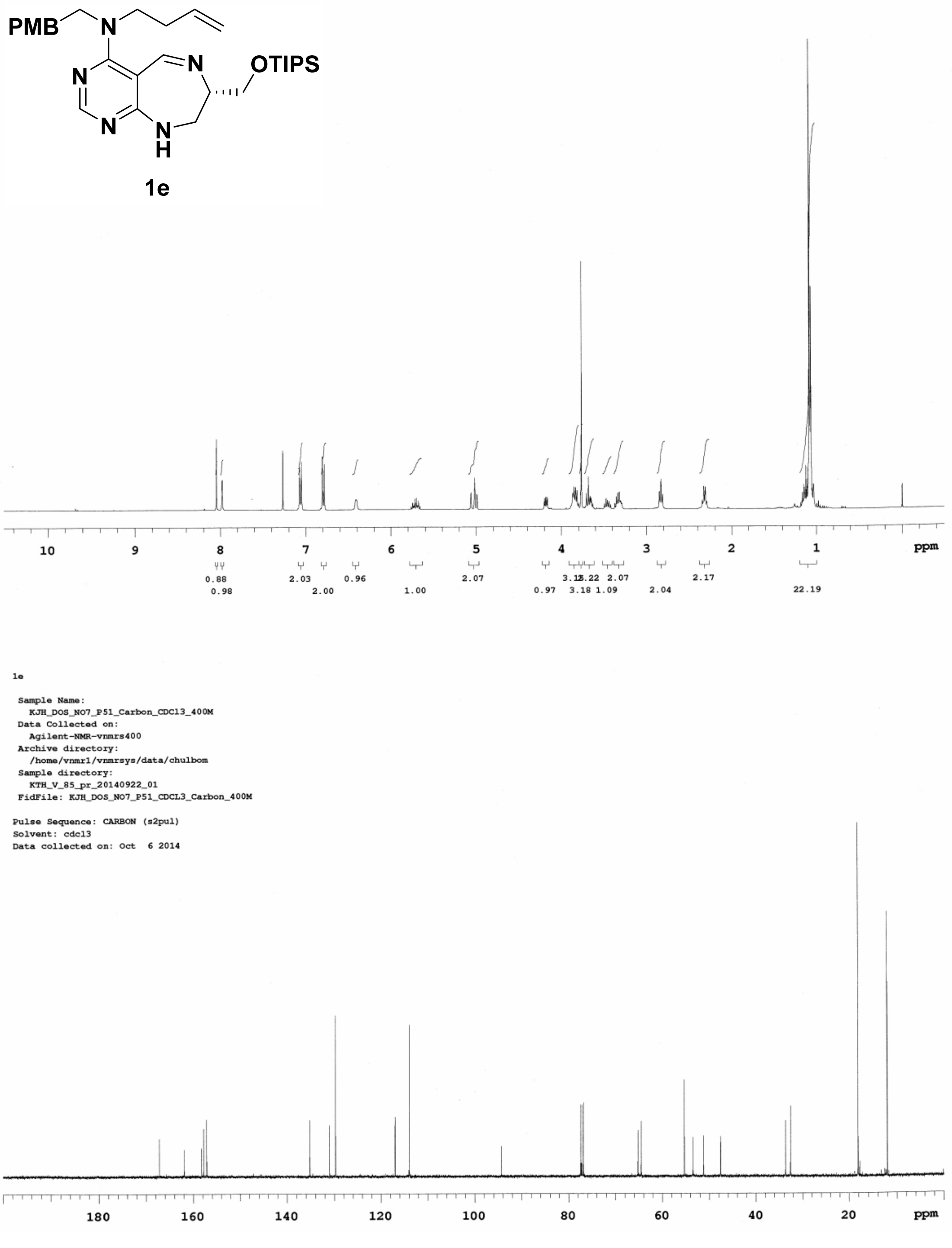

Supplementary Figure 29. ^{1}H and ^{13}C NMR spectra for **1e**.

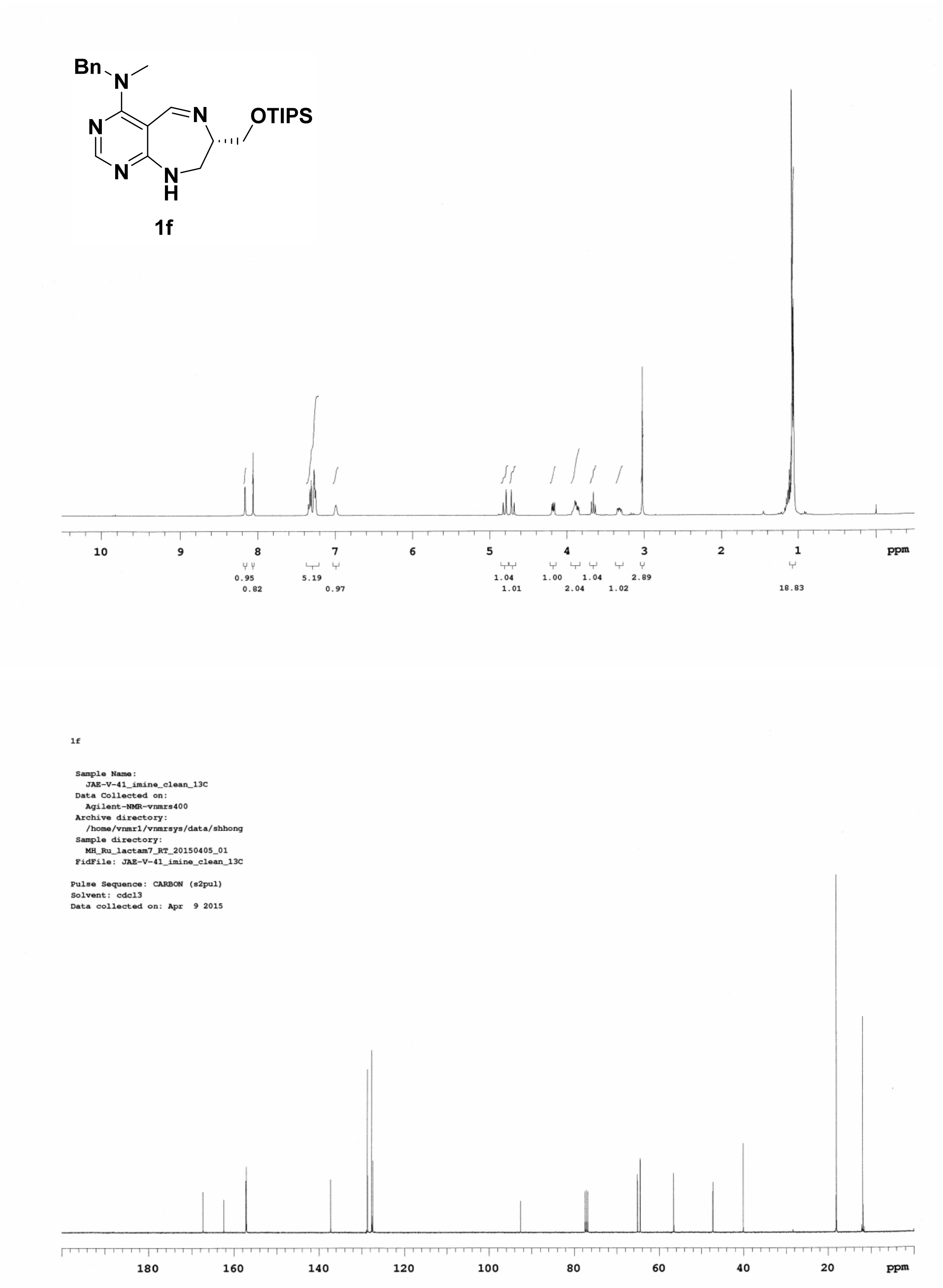

Supplementary Figure 30. ^{1}H and ^{13}C NMR spectra for **1f**.

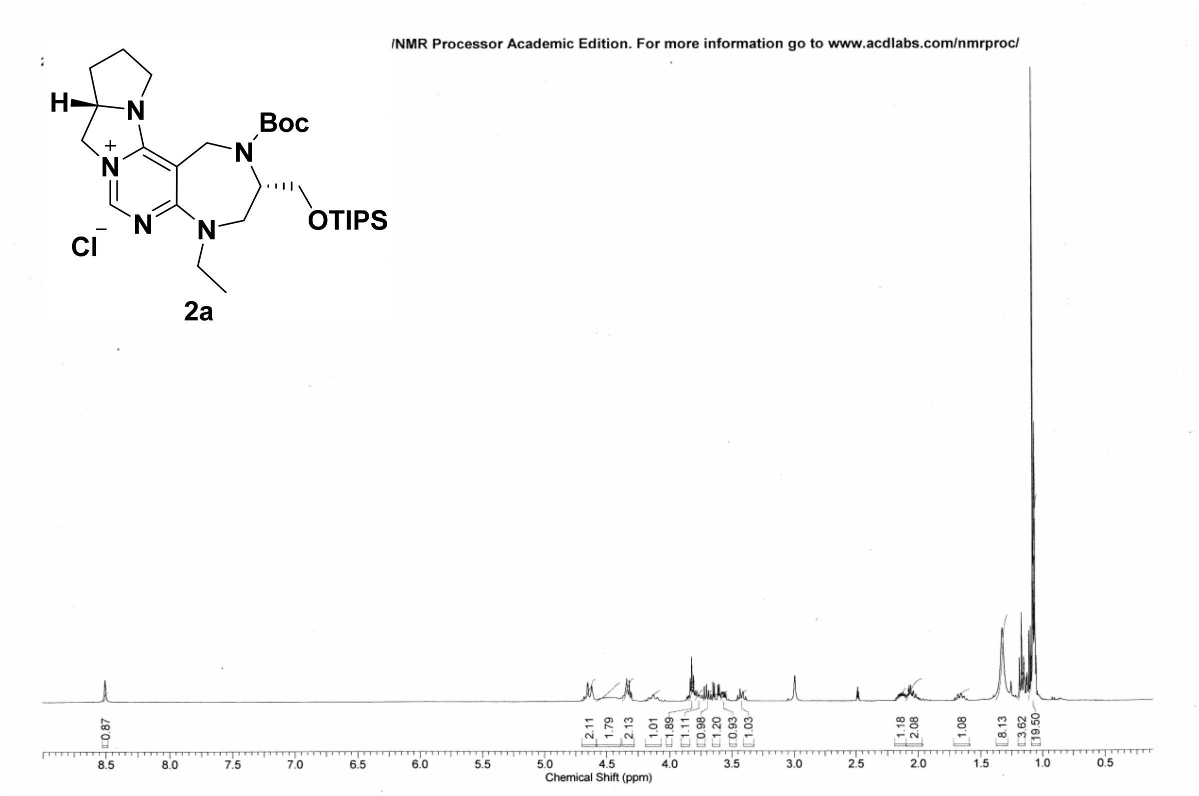

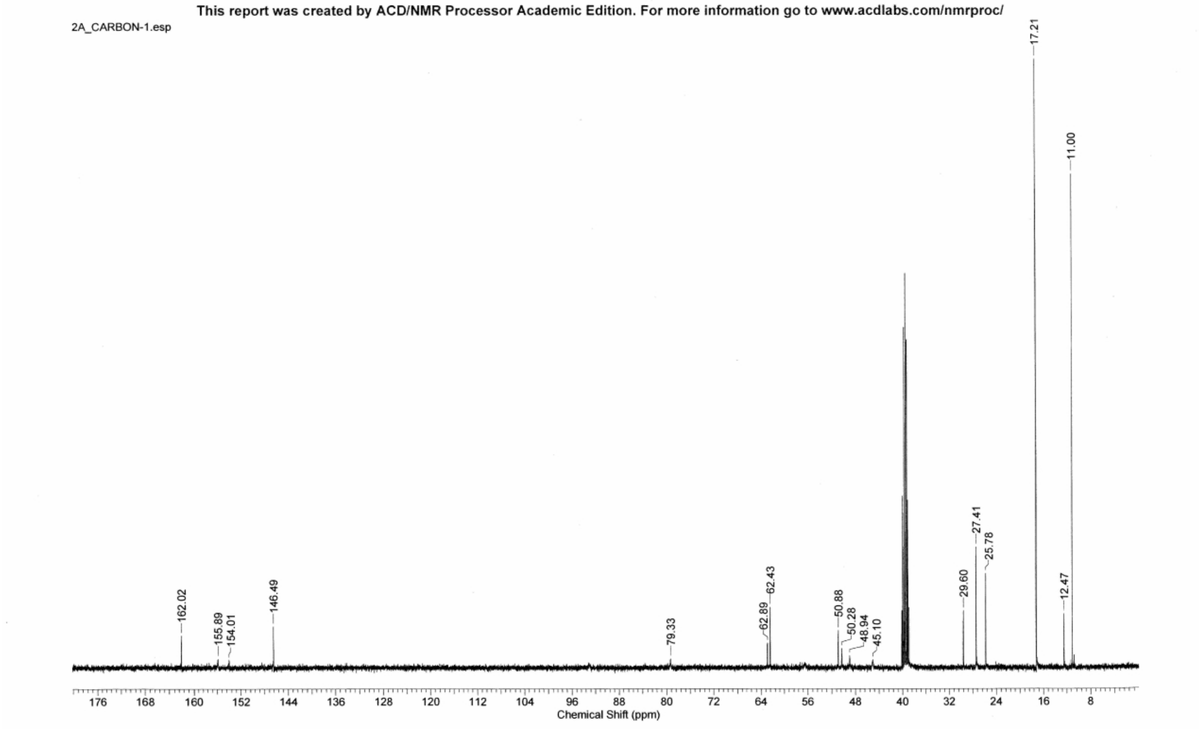

Supplementary Figure 31. ^{1}H and ^{13}C NMR spectra for **2a**.

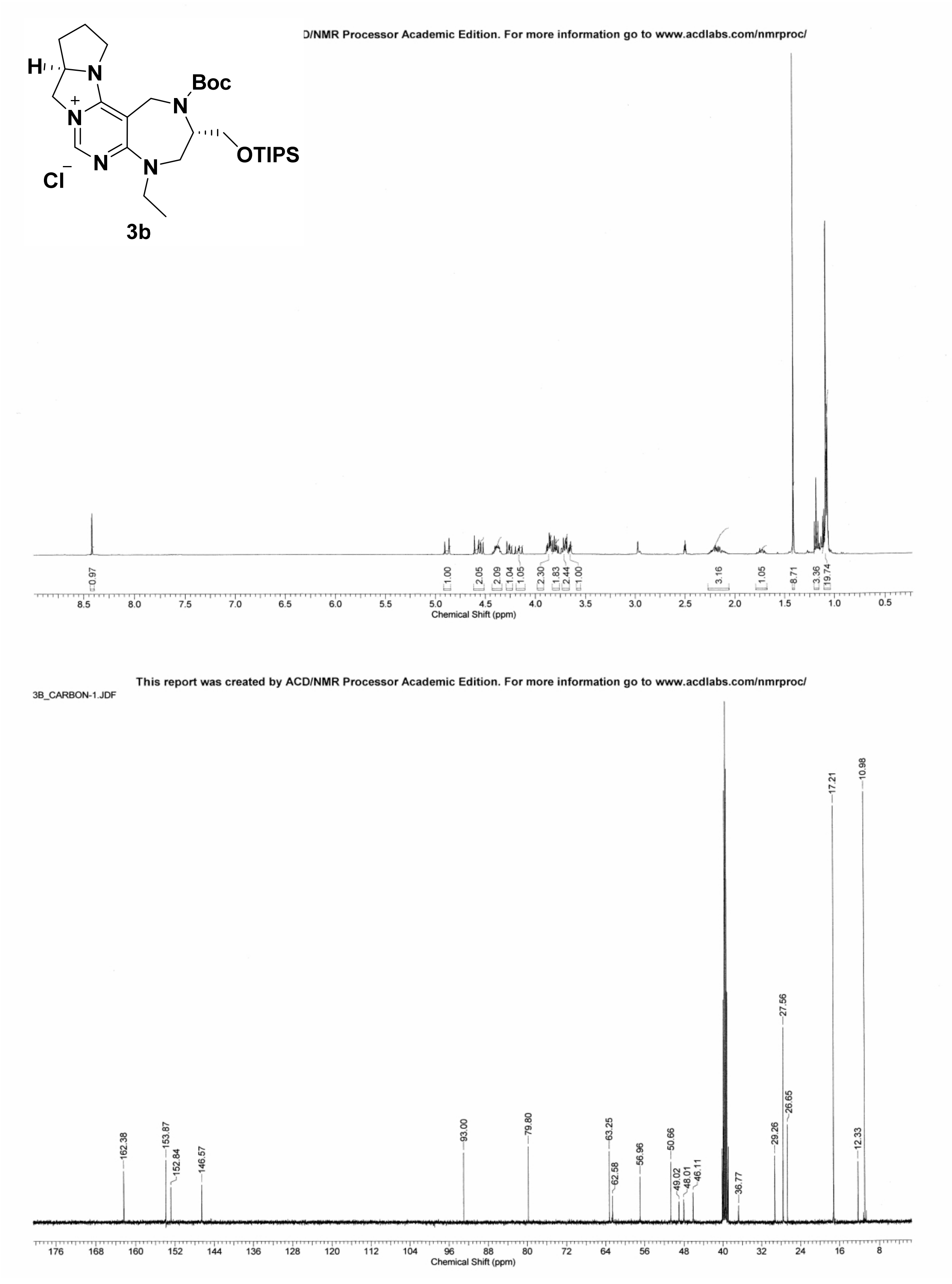

Supplementary Figure 32. ^{1}H and ^{13}C NMR spectra for **3b**.

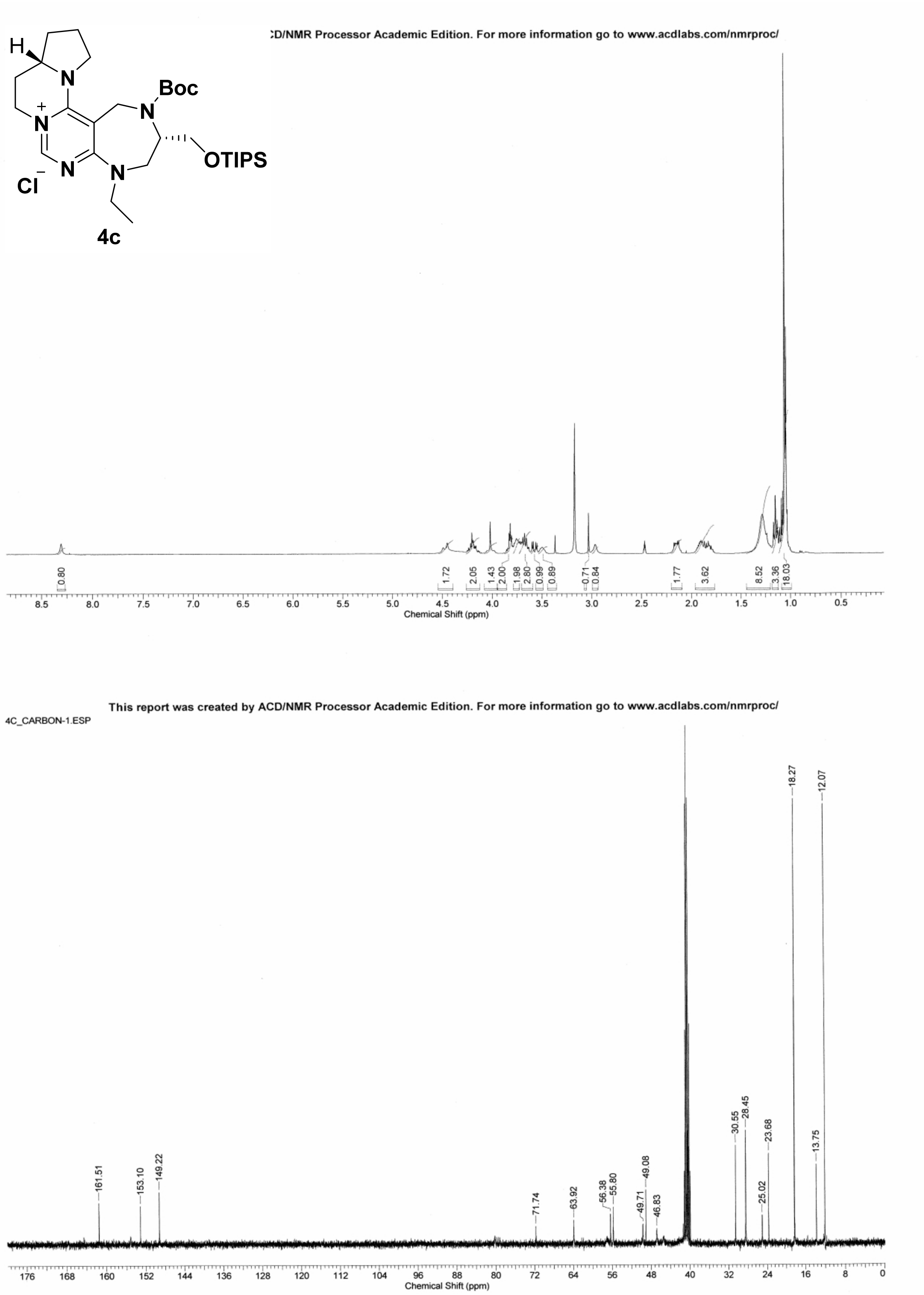

Supplementary Figure 33. ^{1}H and ^{13}C NMR spectra for **4c**.

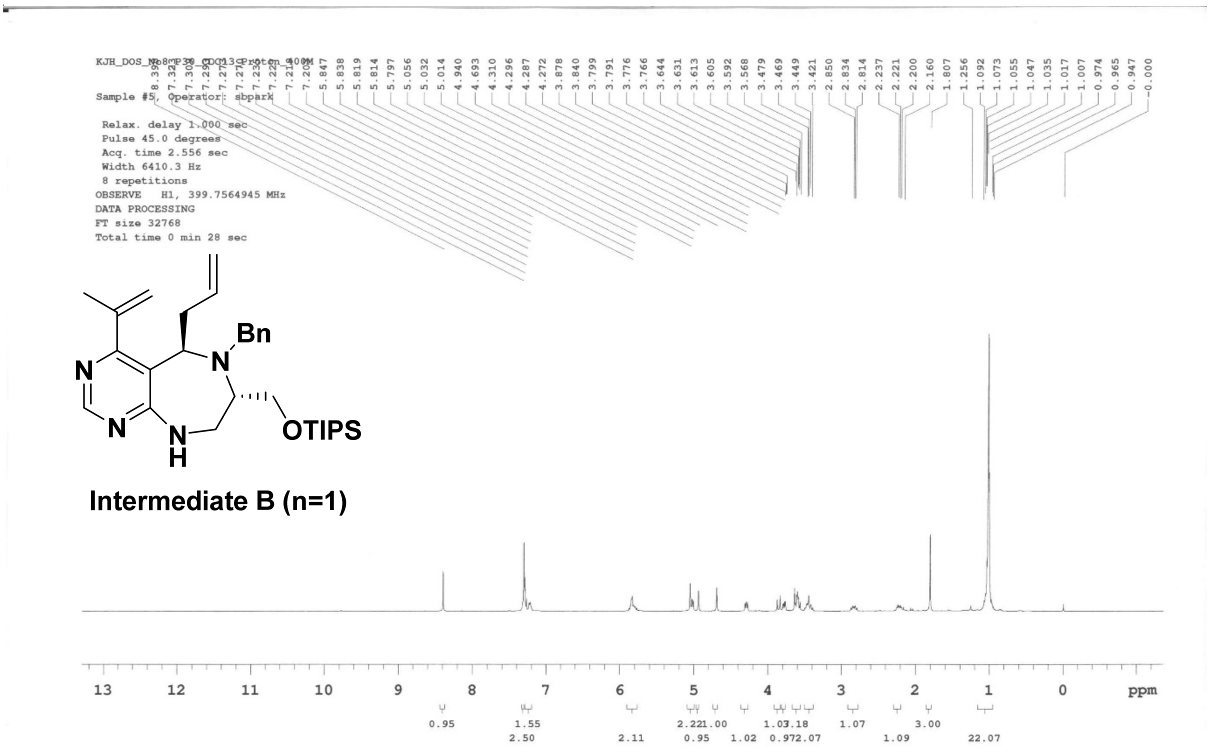

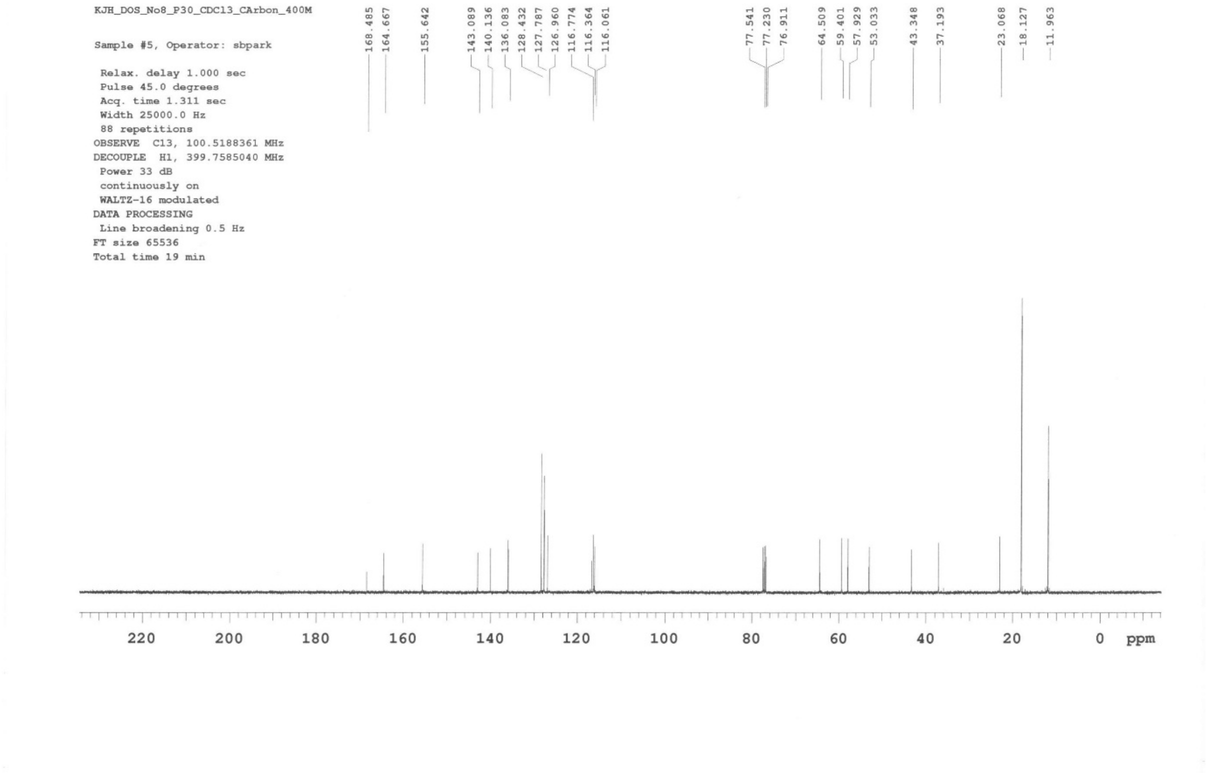

Supplementary Figure 34. ^{1}H and ^{13}C NMR spectra for **Intermediate B (n=1)**.

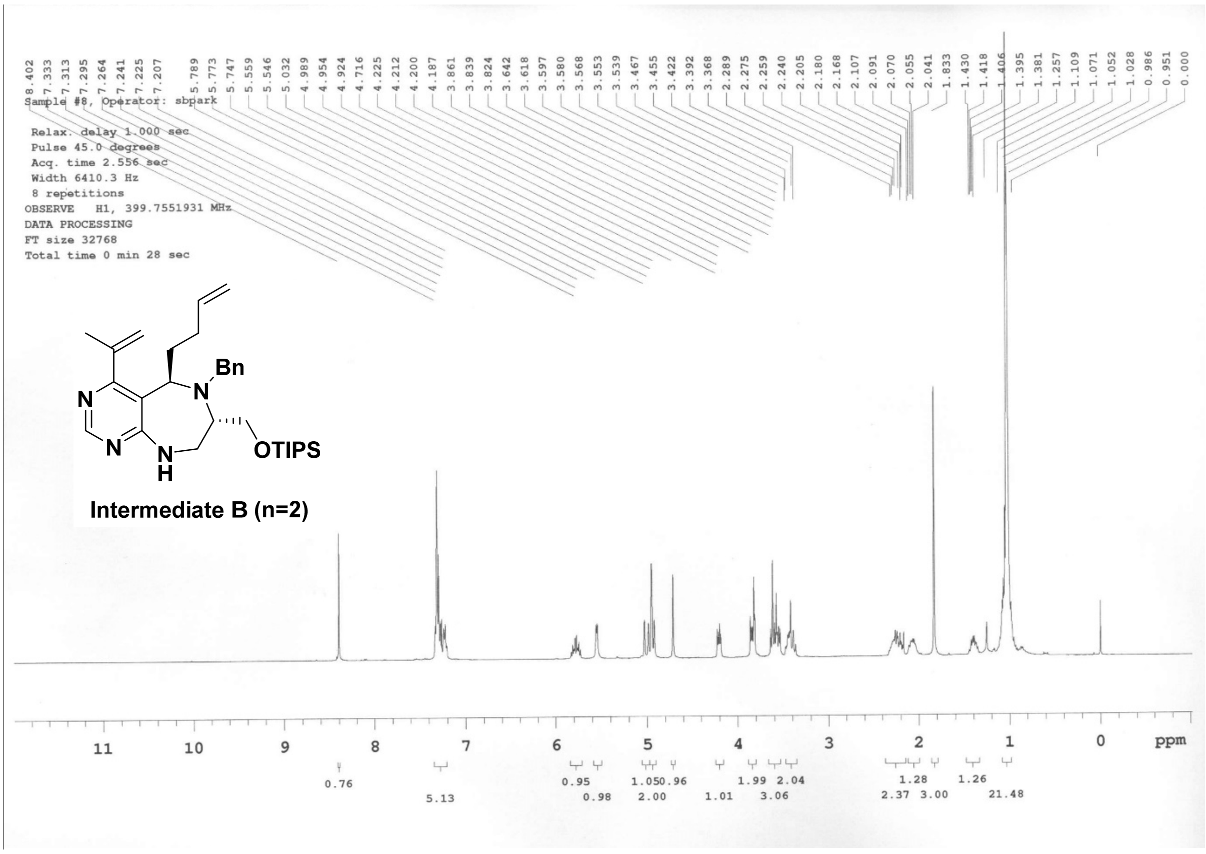

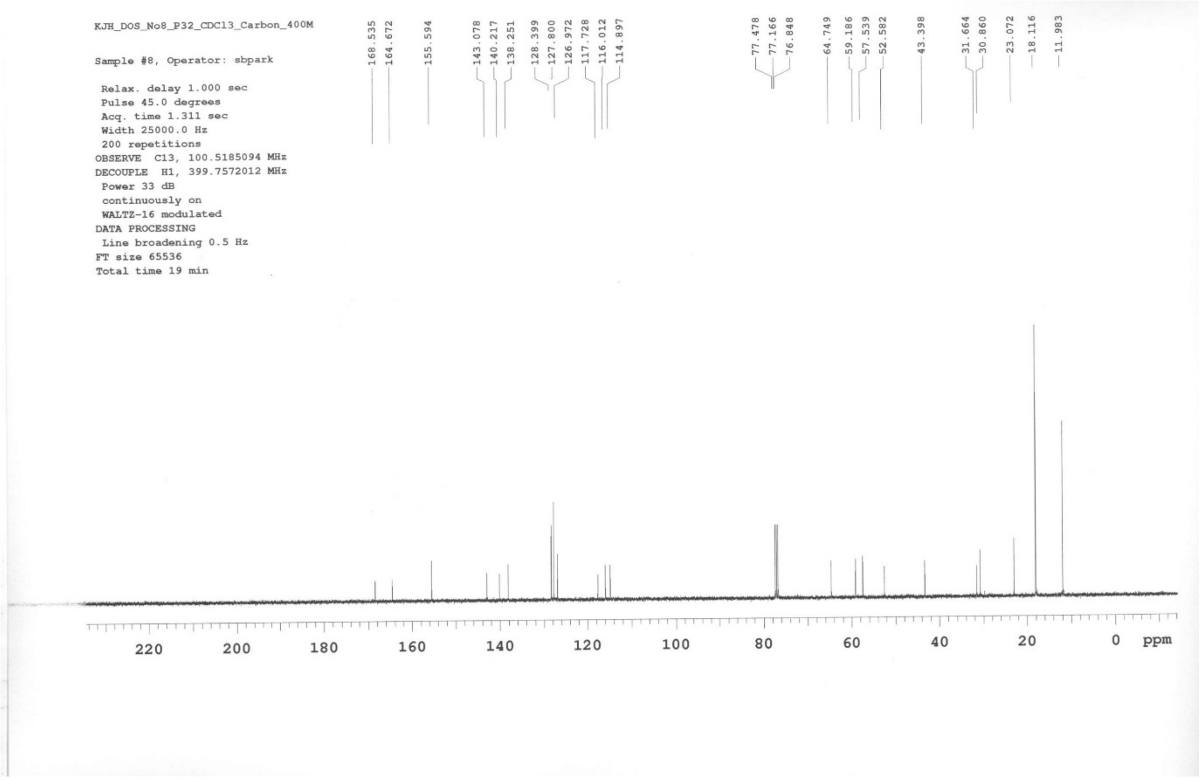

Supplementary Figure 35. ^{1}H and ^{13}C NMR spectra for **Intermediate B (n=2)**.

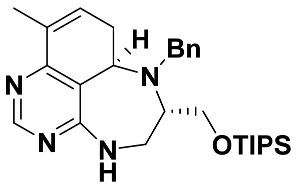

5d

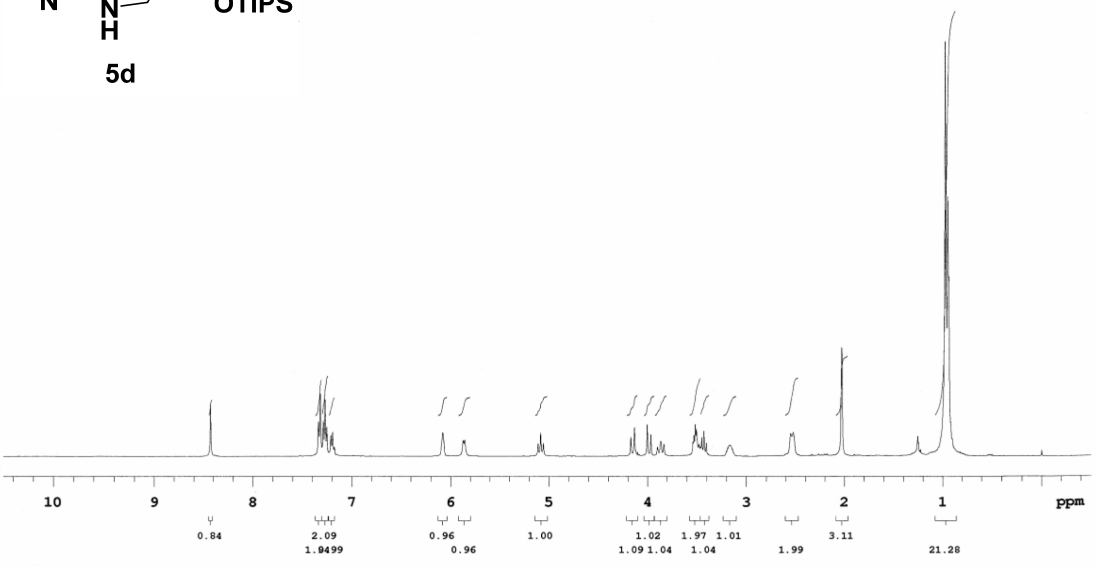

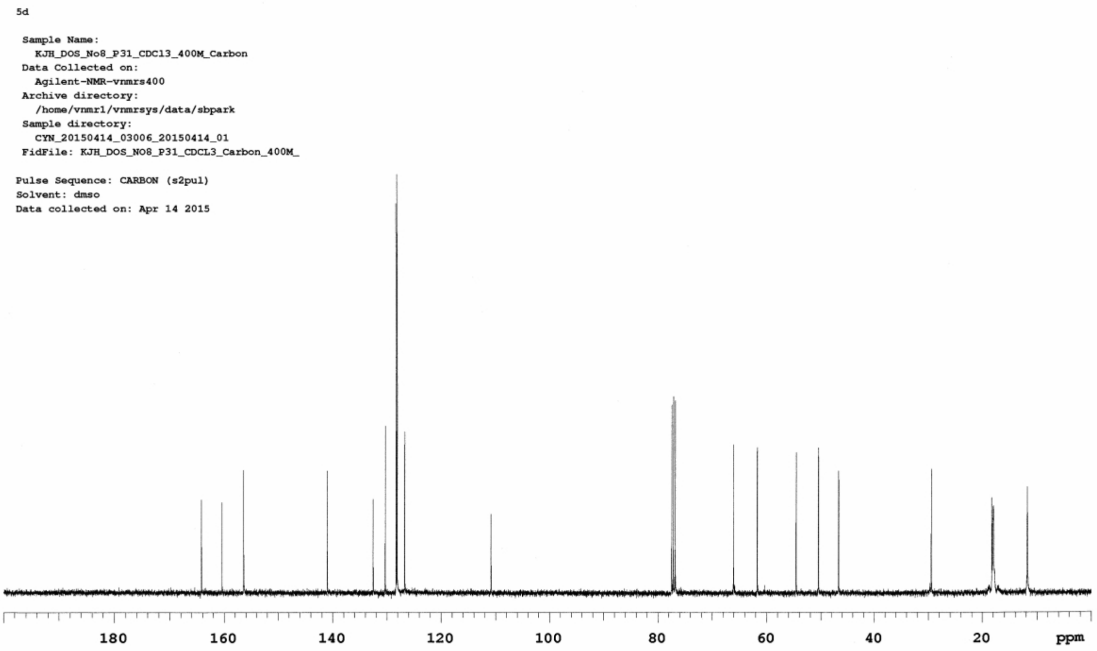

Supplementary Figure 36. ^{1}H and ^{13}C NMR spectra for **5d**.

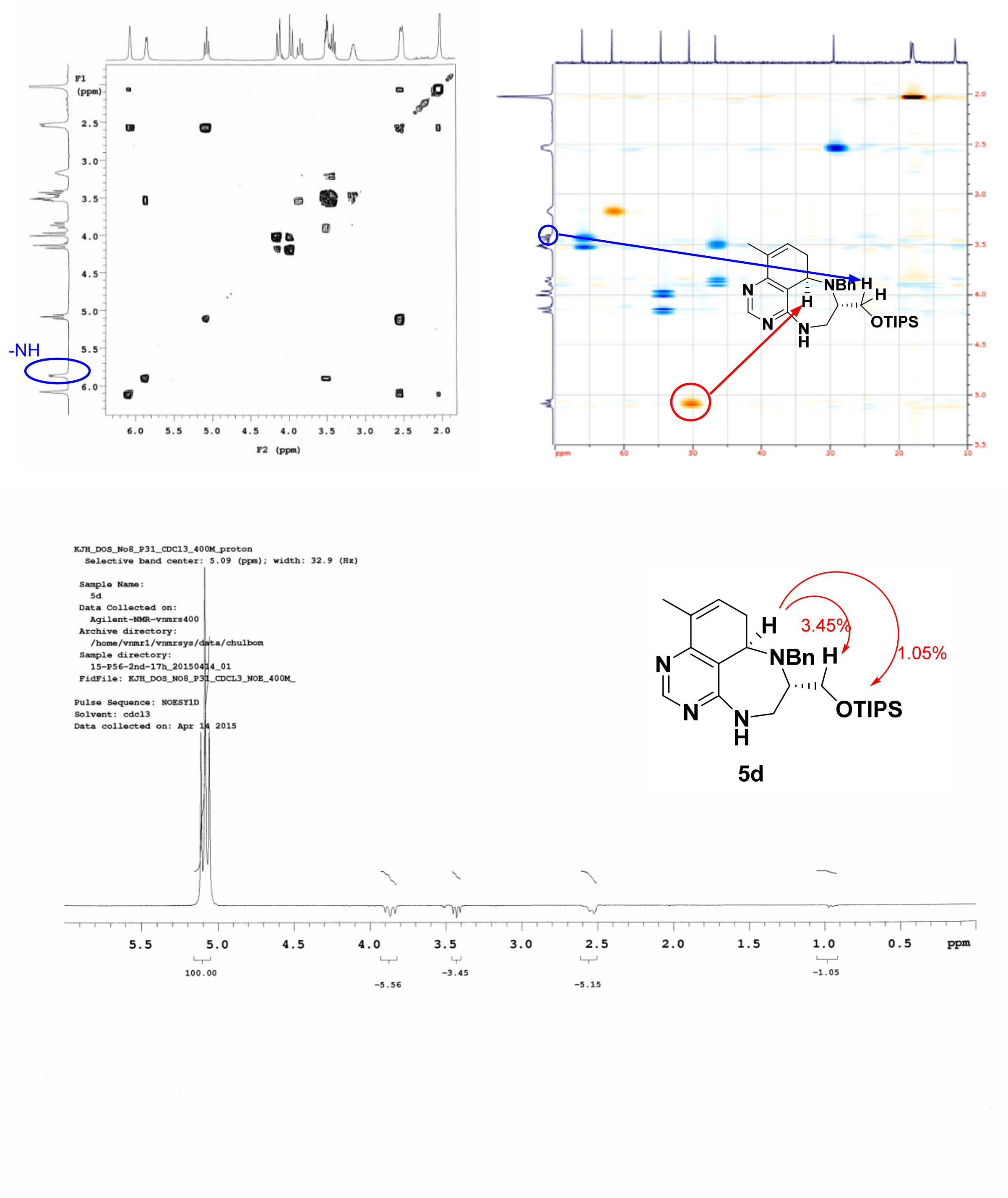

Supplementary Figure 37. HSQC and NOE spectra for **5d**.

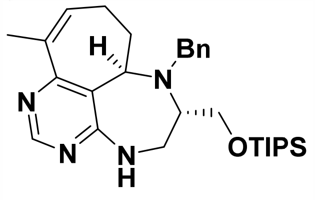

6d

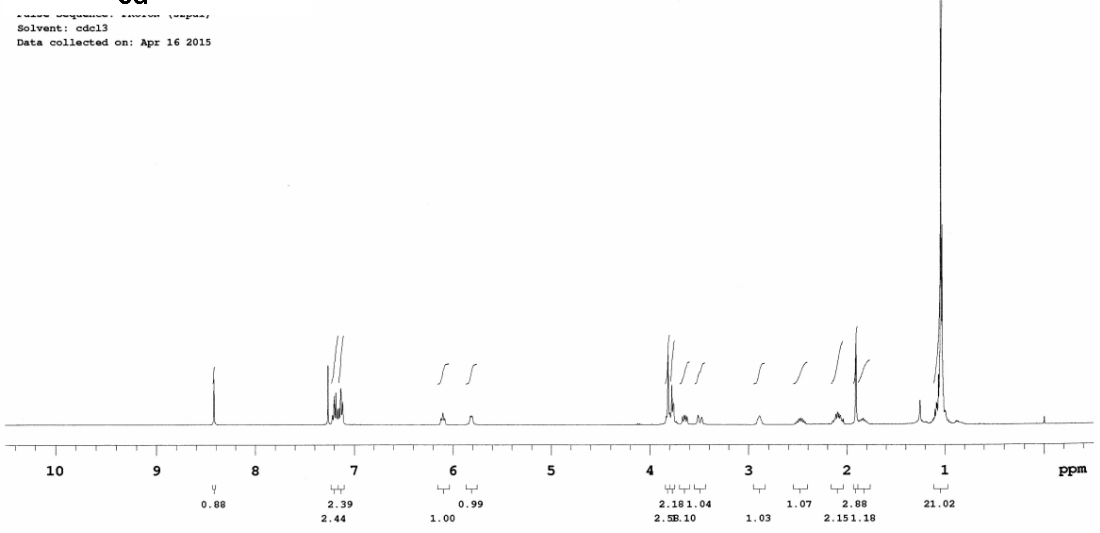

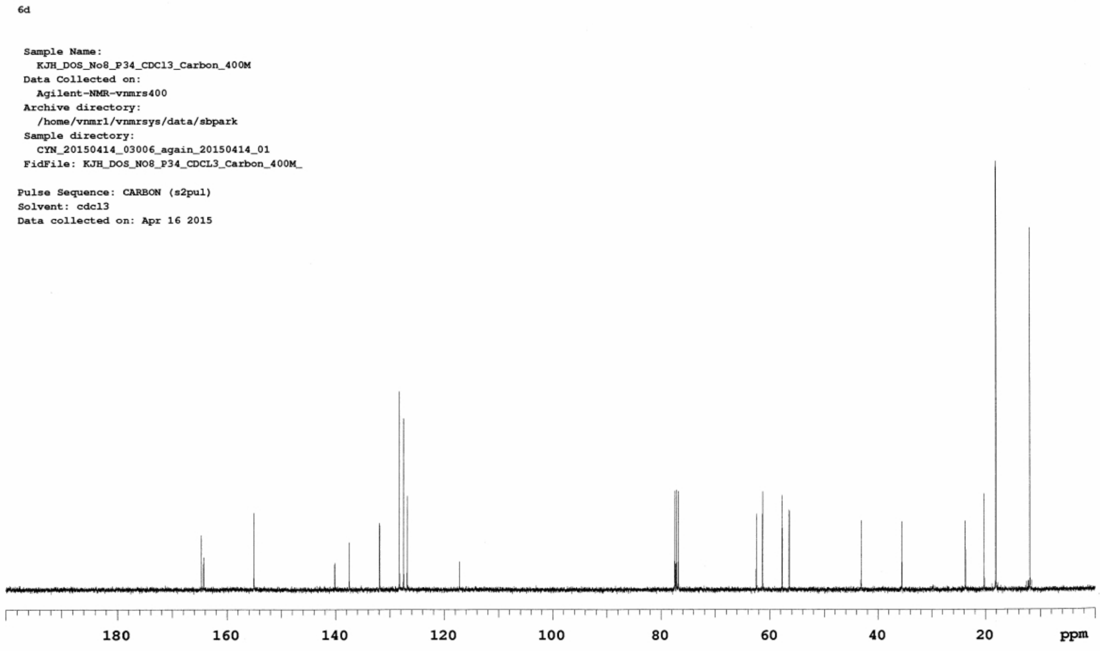

Supplementary Figure 38. ^{1}H and ^{13}C NMR spectra for **6d**.

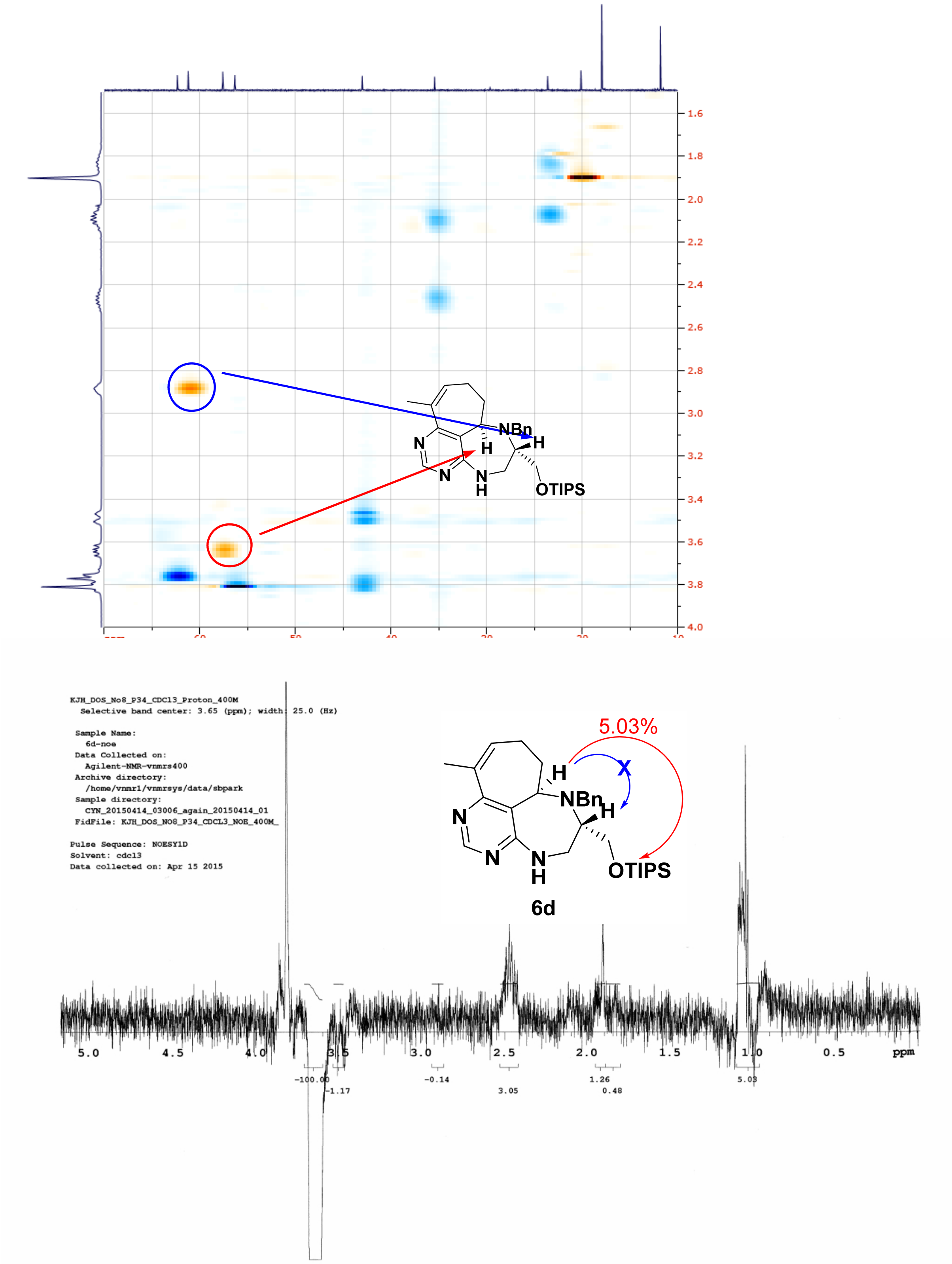

Supplementary Figure 39. HSQC and NOE spectra for **6d**.

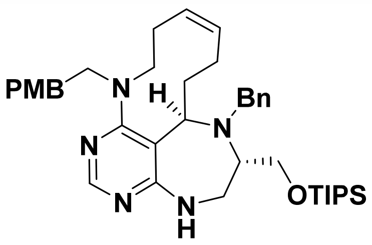

7e

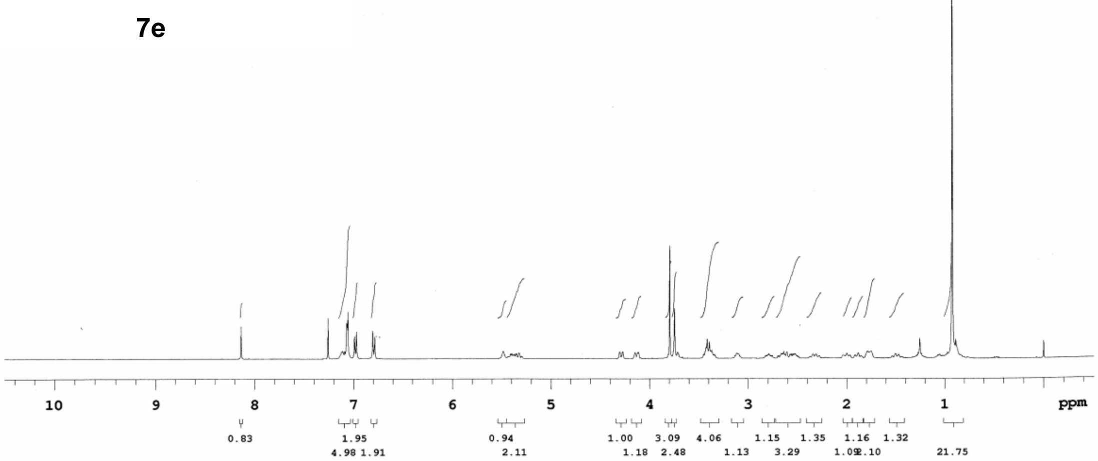

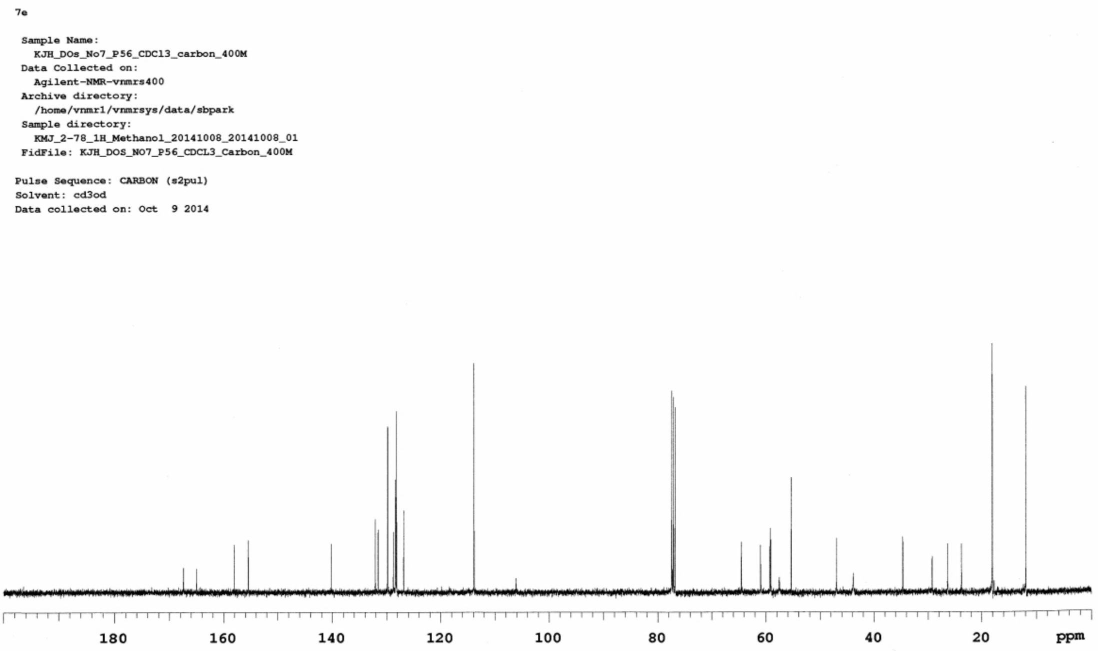

Supplementary Figure 40. ^{1}H and ^{13}C NMR spectra for **7e**.

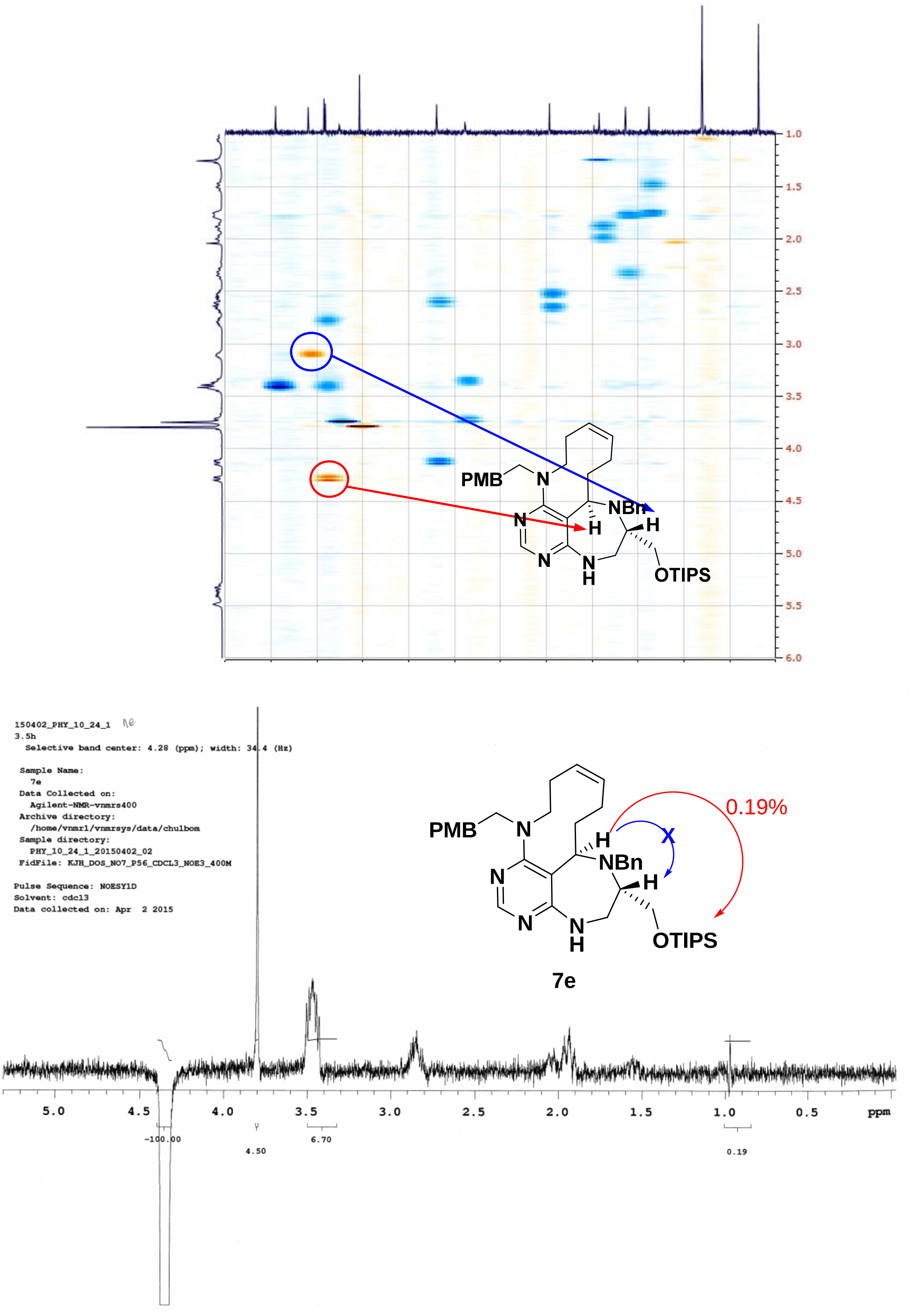

Supplementary Figure 41. HSQC and NOE spectra for **7e**.

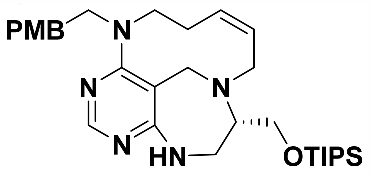

8e

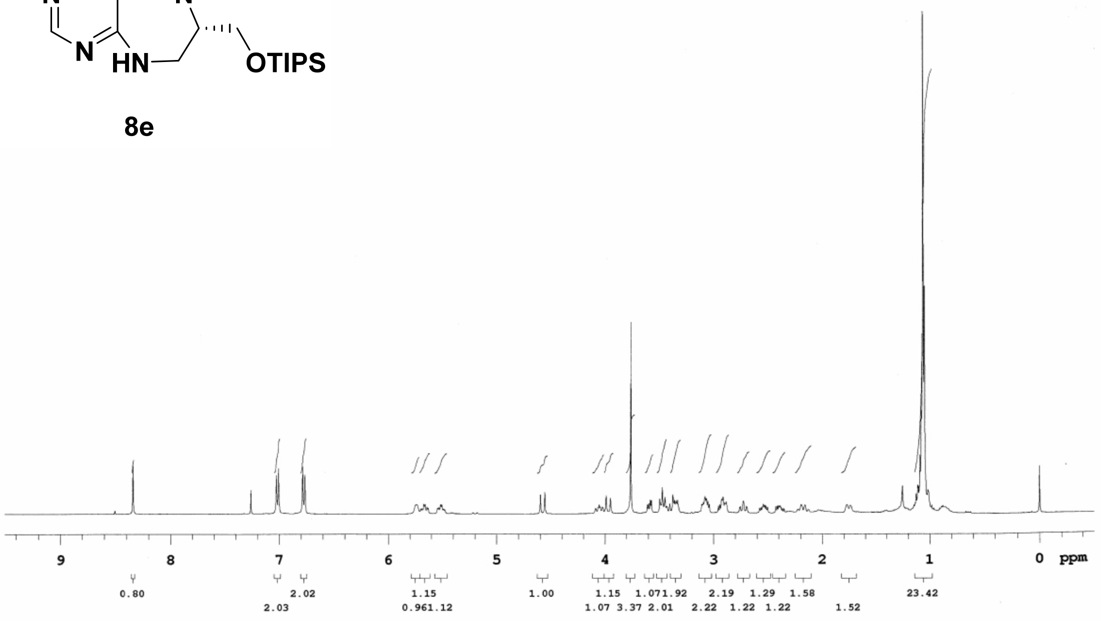

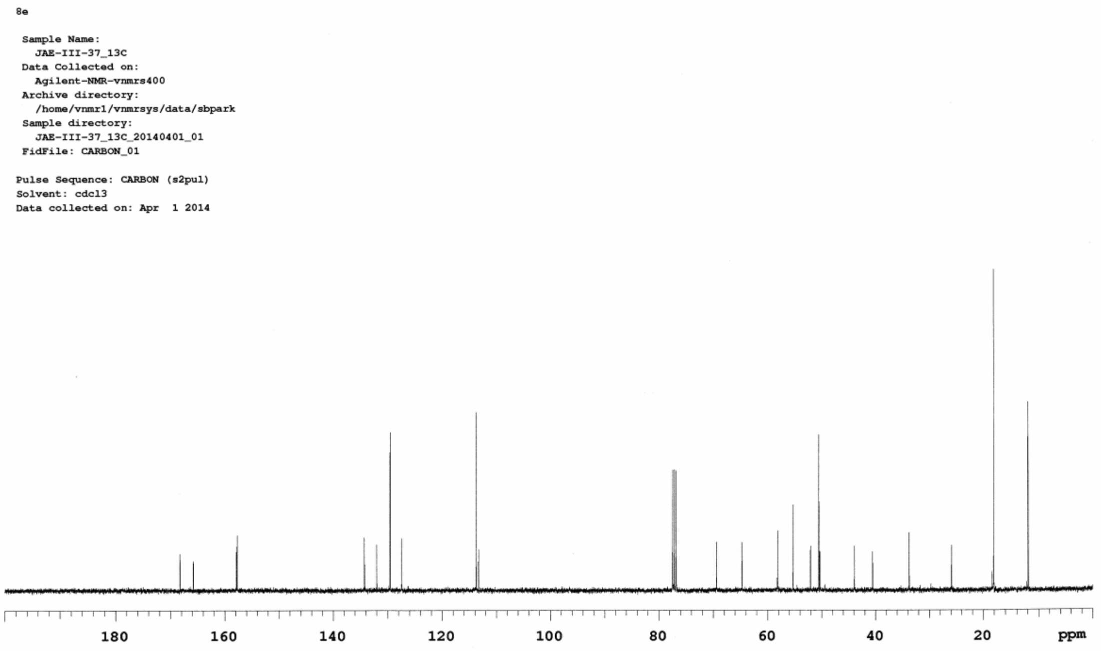

Supplementary Figure 42. ^{1}H and ^{13}C NMR spectra for **8e**.

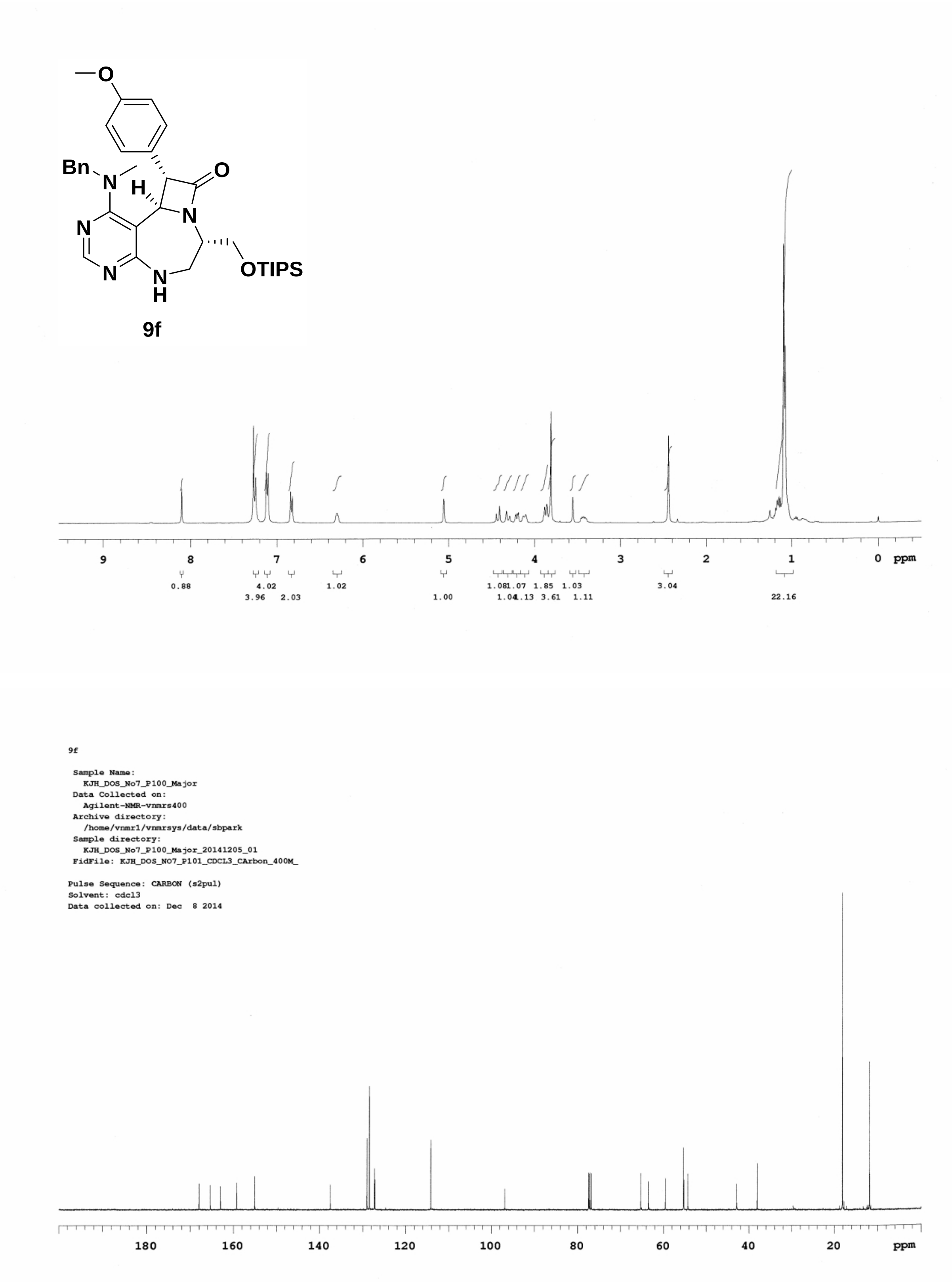

Supplementary Figure 43. ^{1}H and ^{13}C NMR spectra for **9f**.

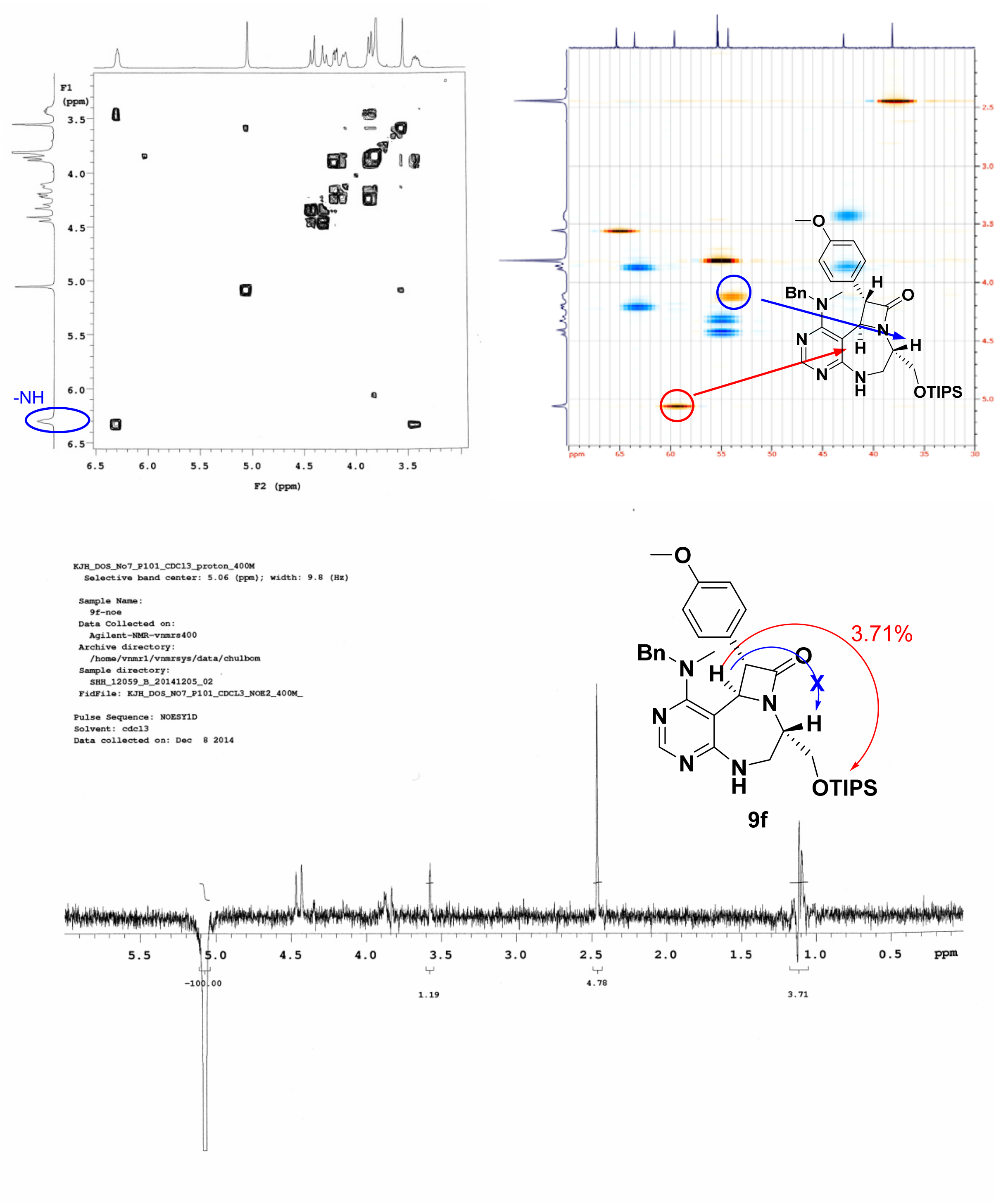

Supplementary Figure 44. COSY, HSQC and NOE spectra for **9f**.

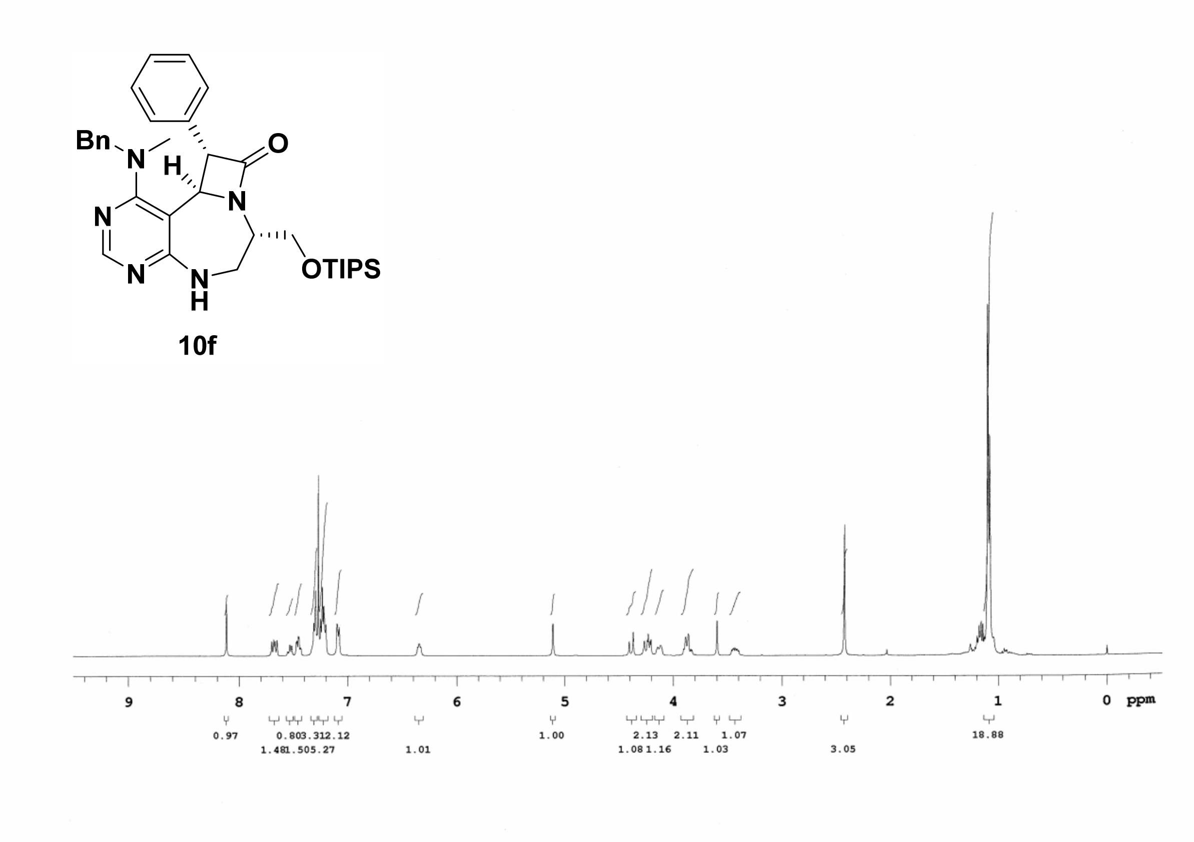

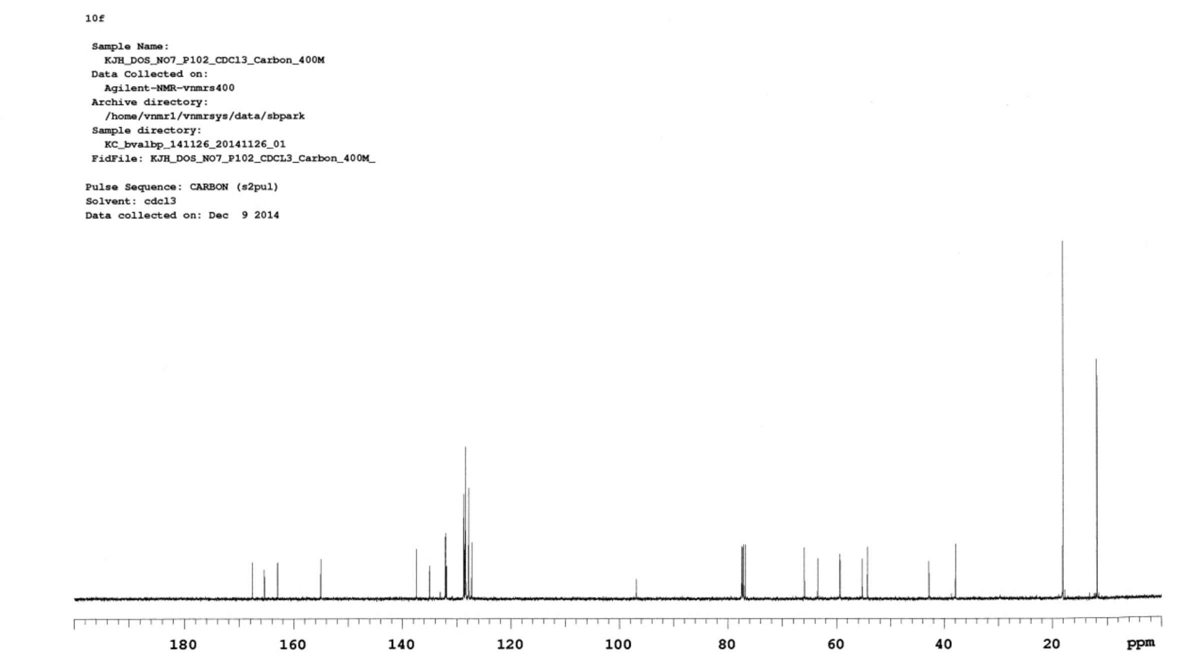

Supplementary Figure 45. ^{1}H and ^{13}C NMR spectra for **10f**.

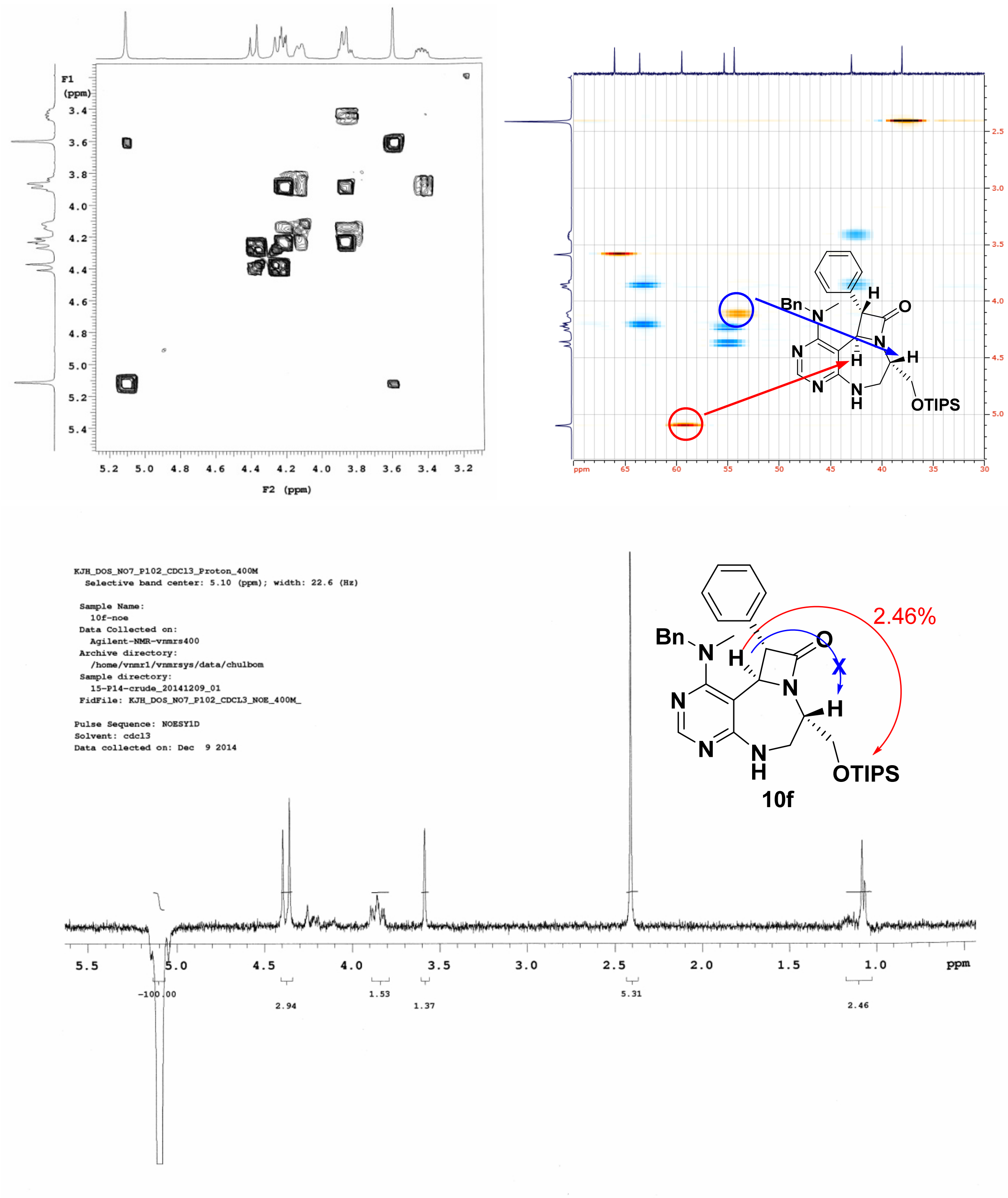

Supplementary Figure 46. COSY, HSQC and NOE spectra for **10f**.

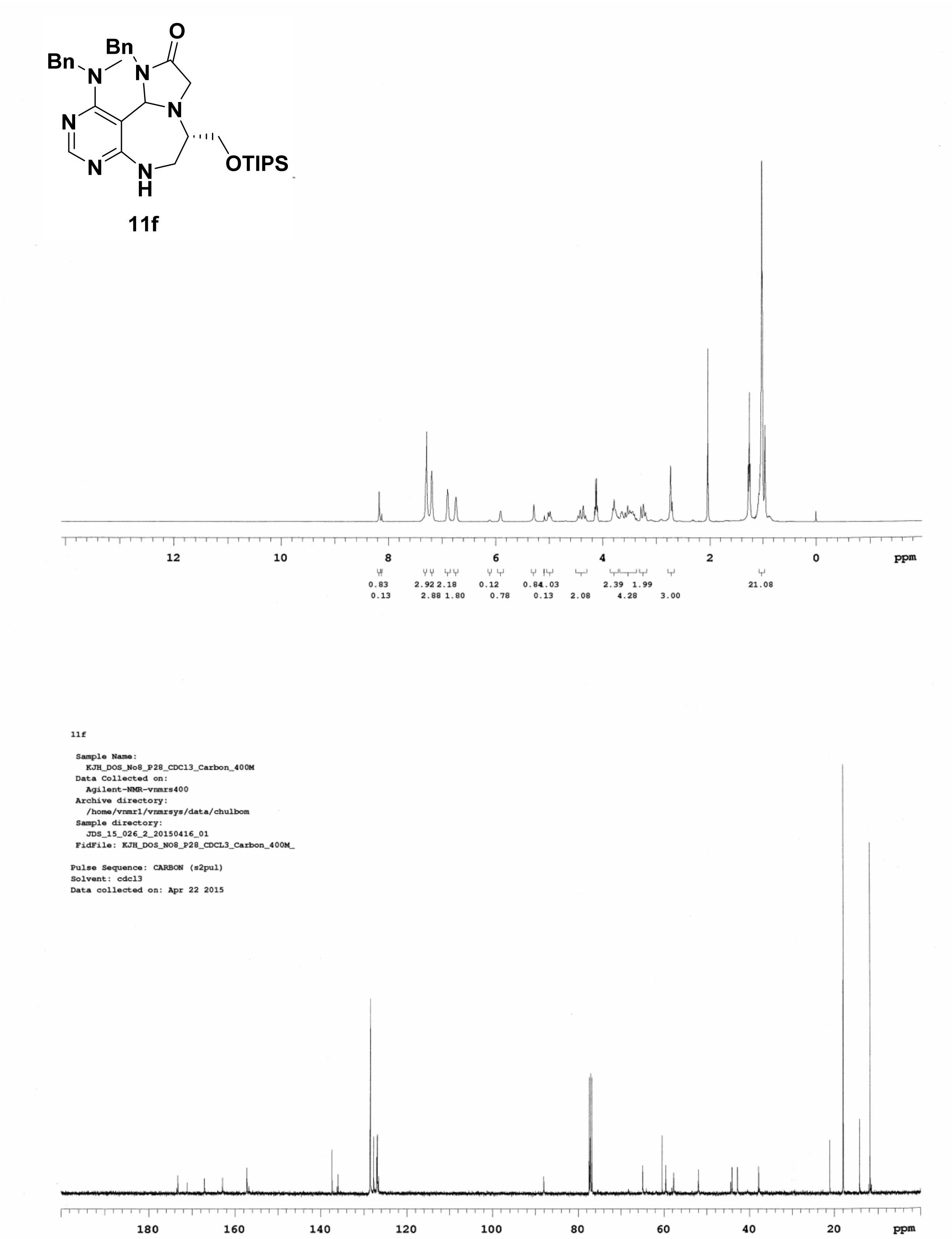

Supplementary Figure 47. ^{1}H and ^{13}C NMR spectra for **11f**.

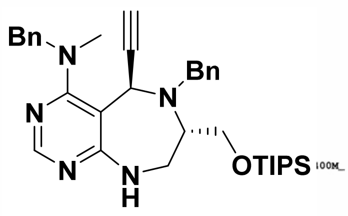

12f

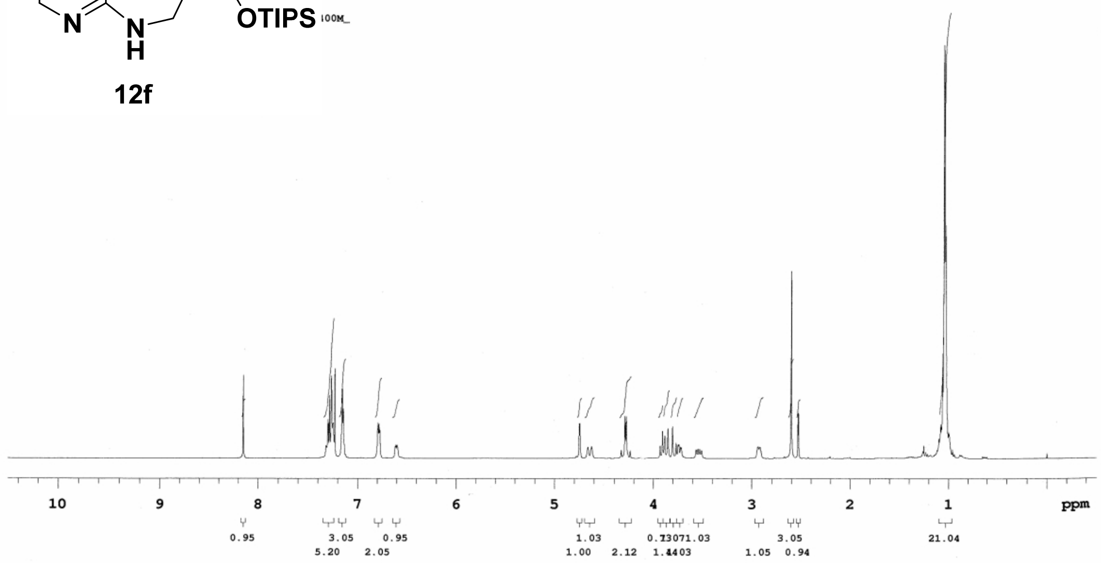

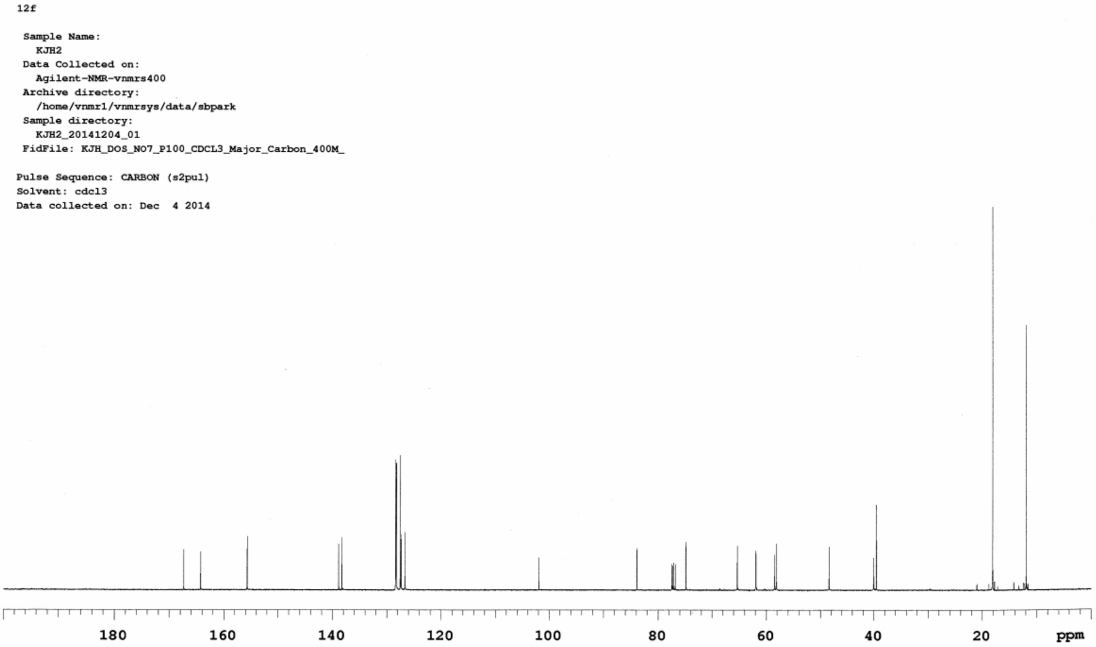

Supplementary Figure 48. ^{1}H and ^{13}C NMR spectra for **12f**.

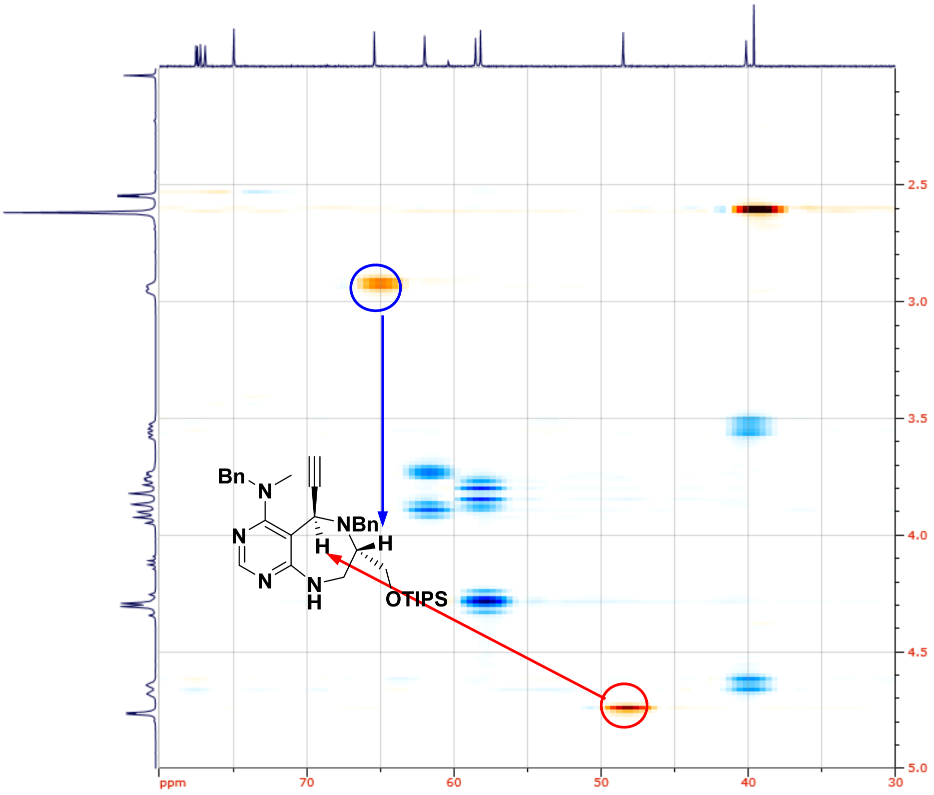

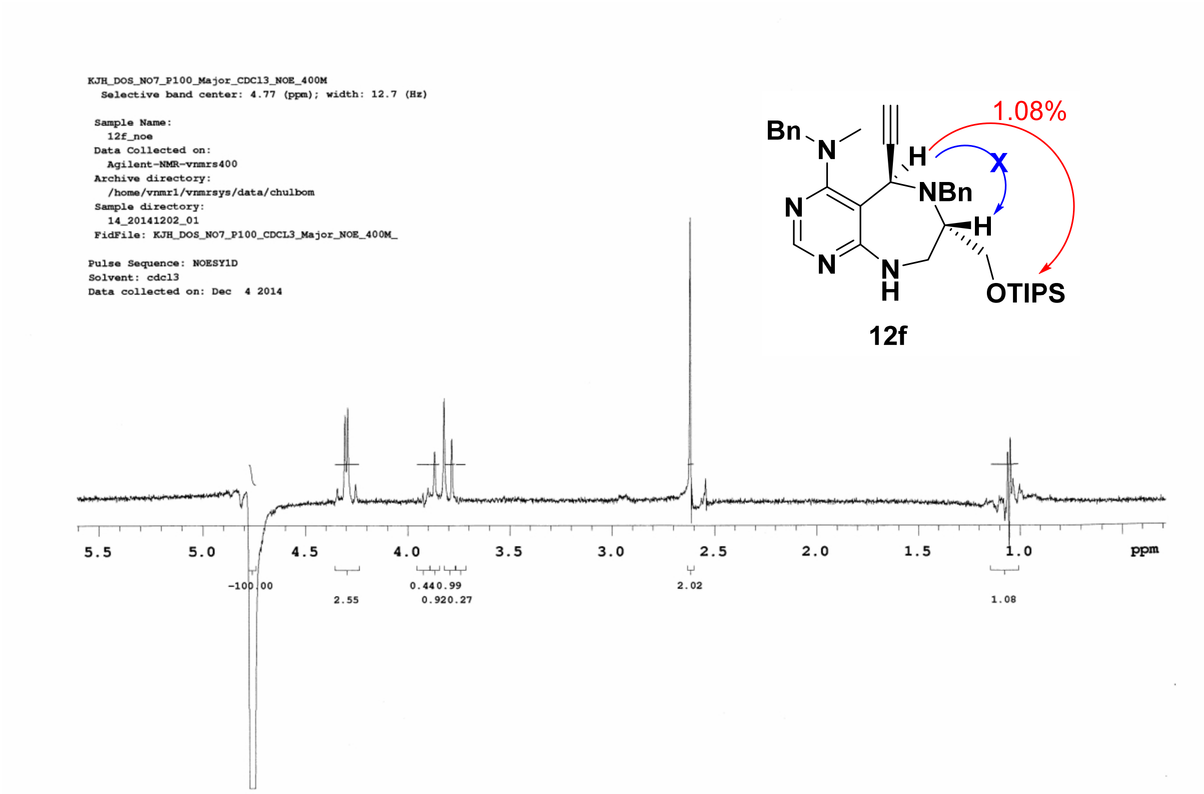

Supplementary Figure 49. HSQC and NOE spectra for **12f**.

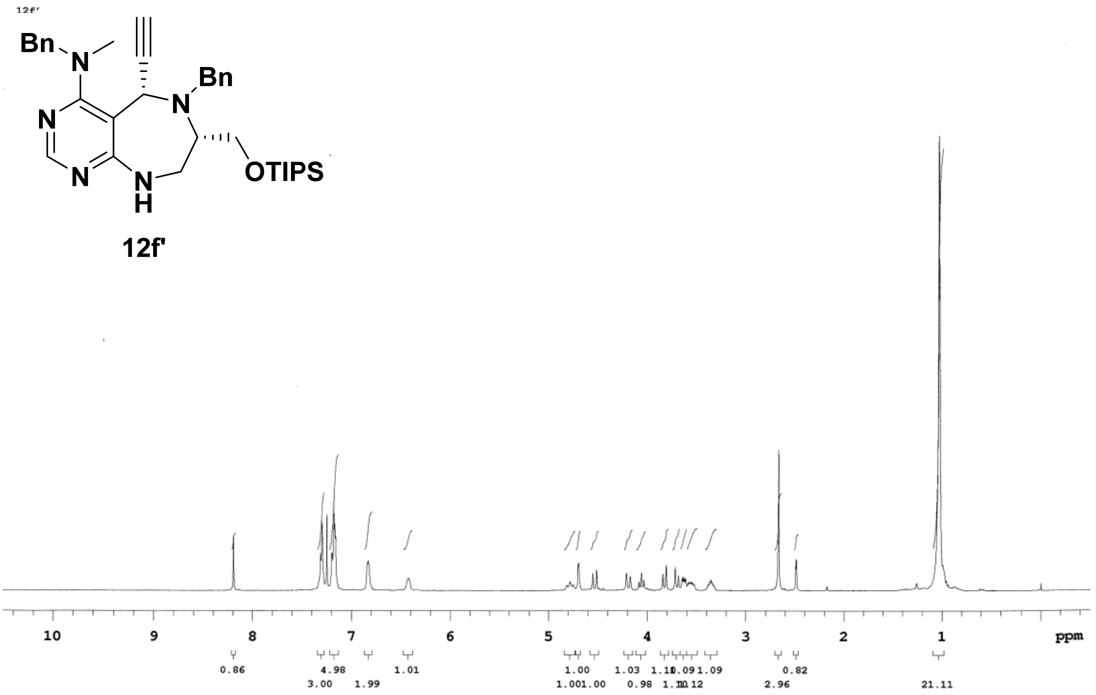

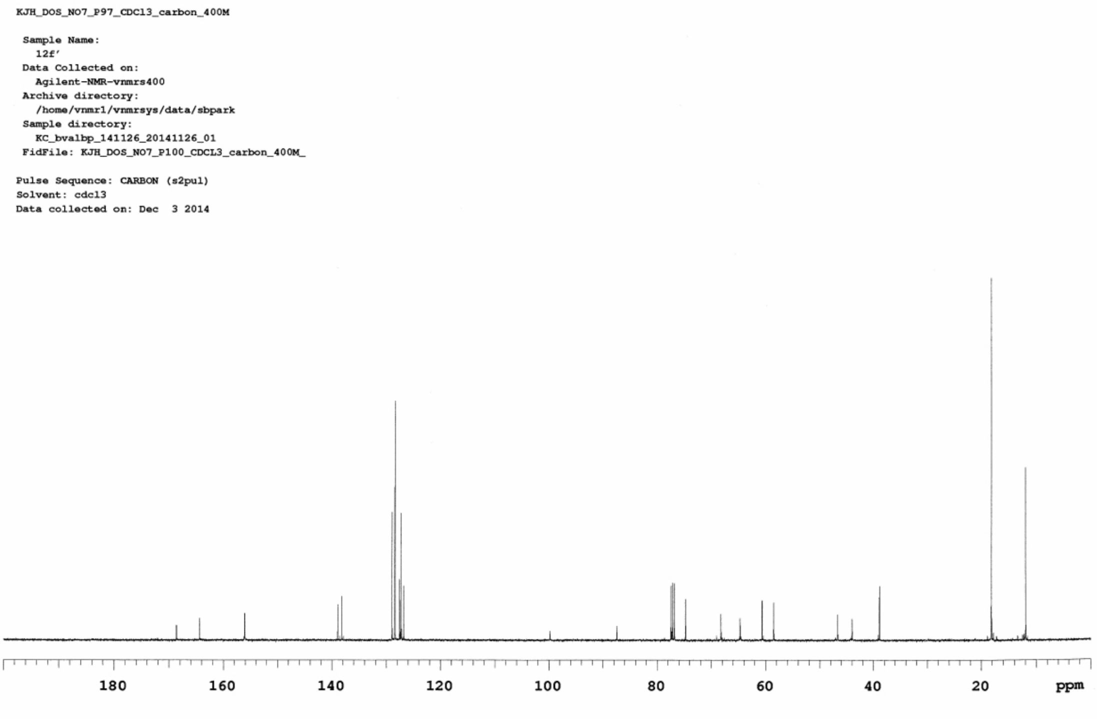

Supplementary Figure 50. ^{1}H and ^{13}C NMR spectra for **12f´**.

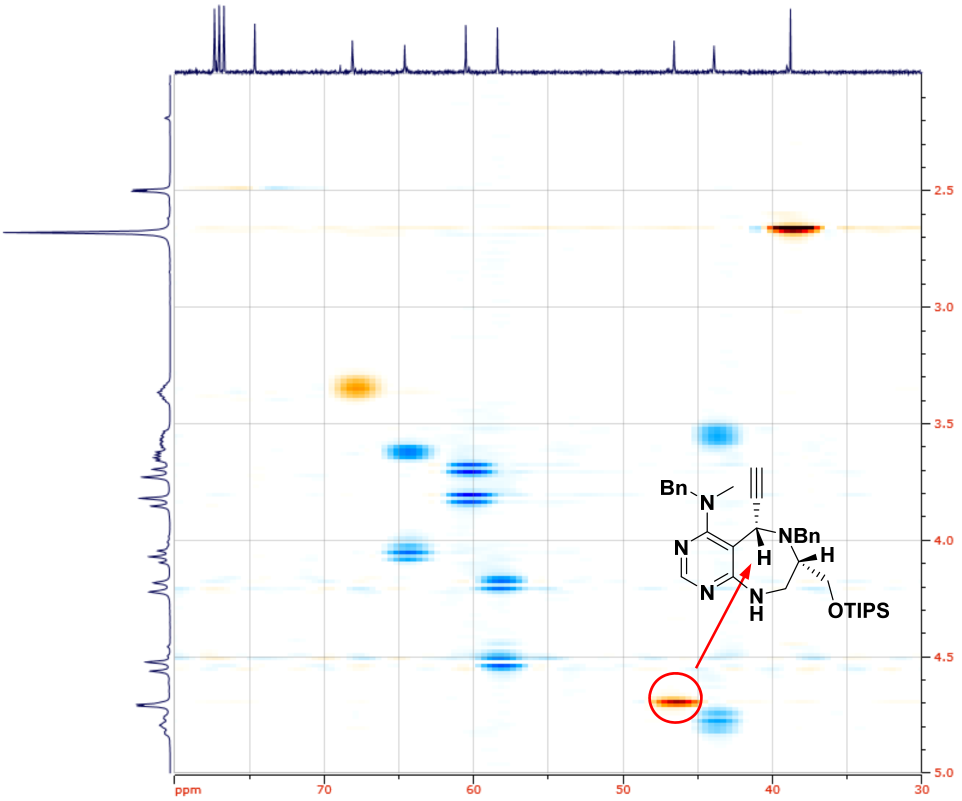

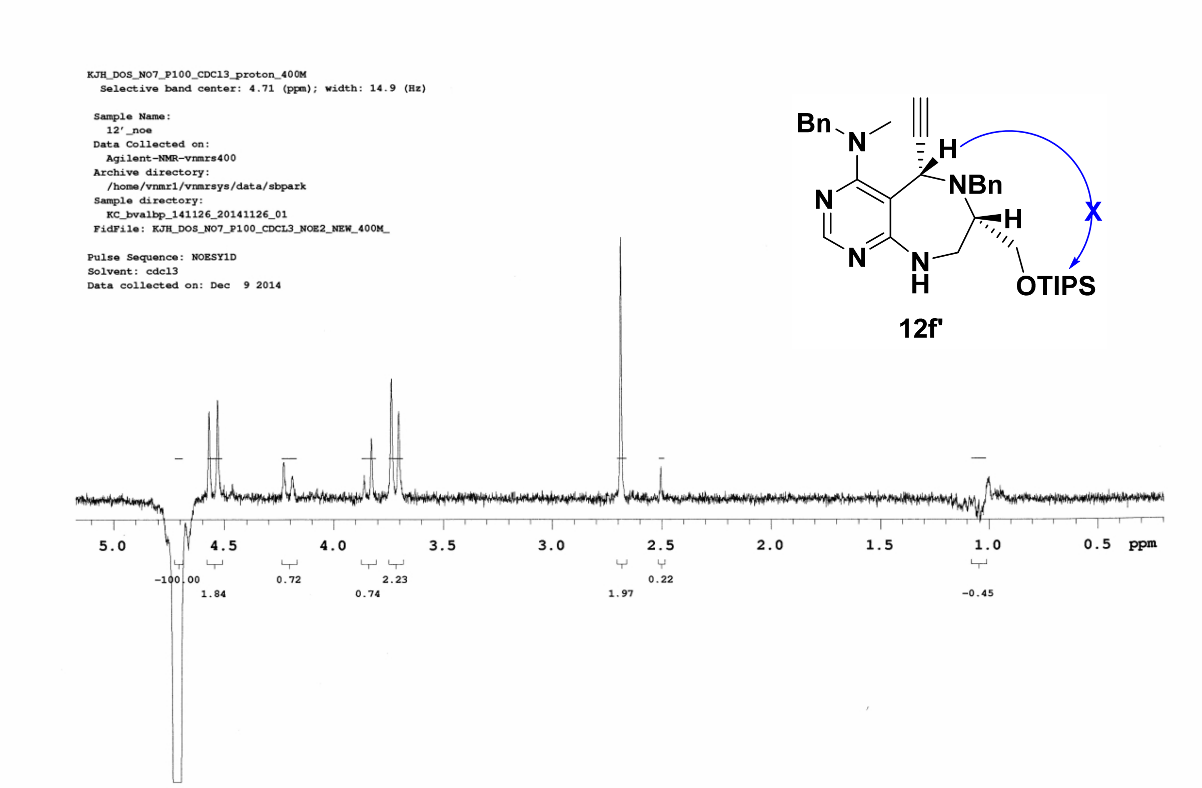

Supplementary Figure 51. HSQC and NOE spectra for **12f**´.

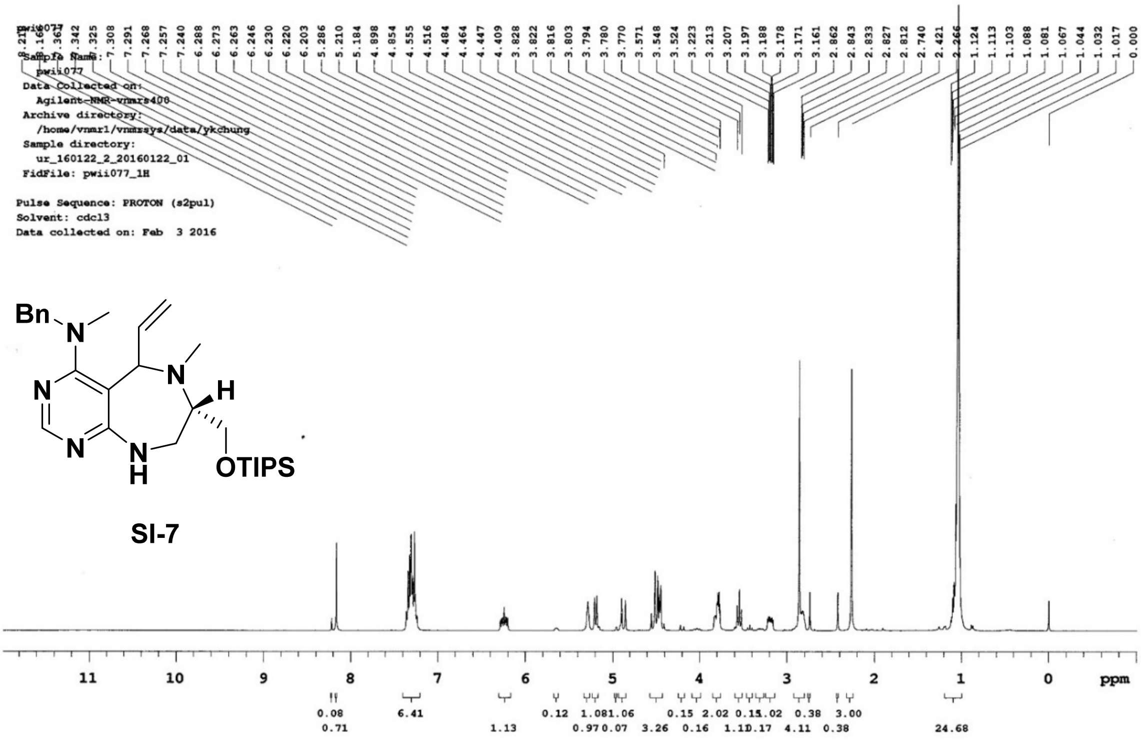

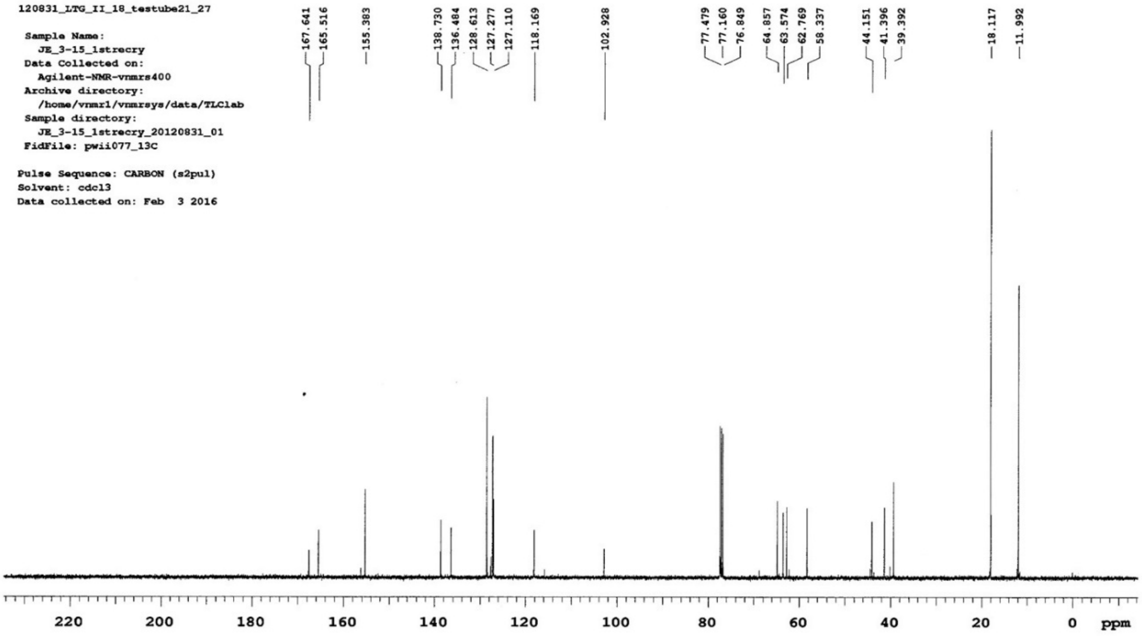

Supplementary Figure 52. ^{1}H and ^{13}C NMR spectra for **SI-7**.

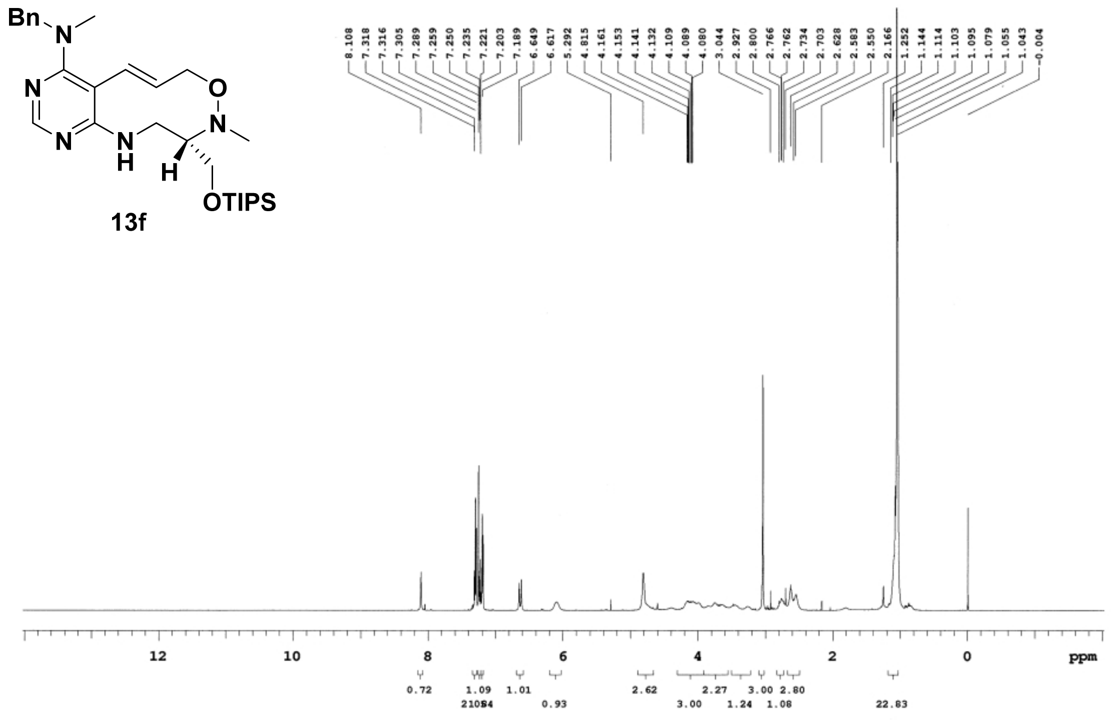

Supplementary Figure 53. ^{1}H NMR spectra for **13f**.

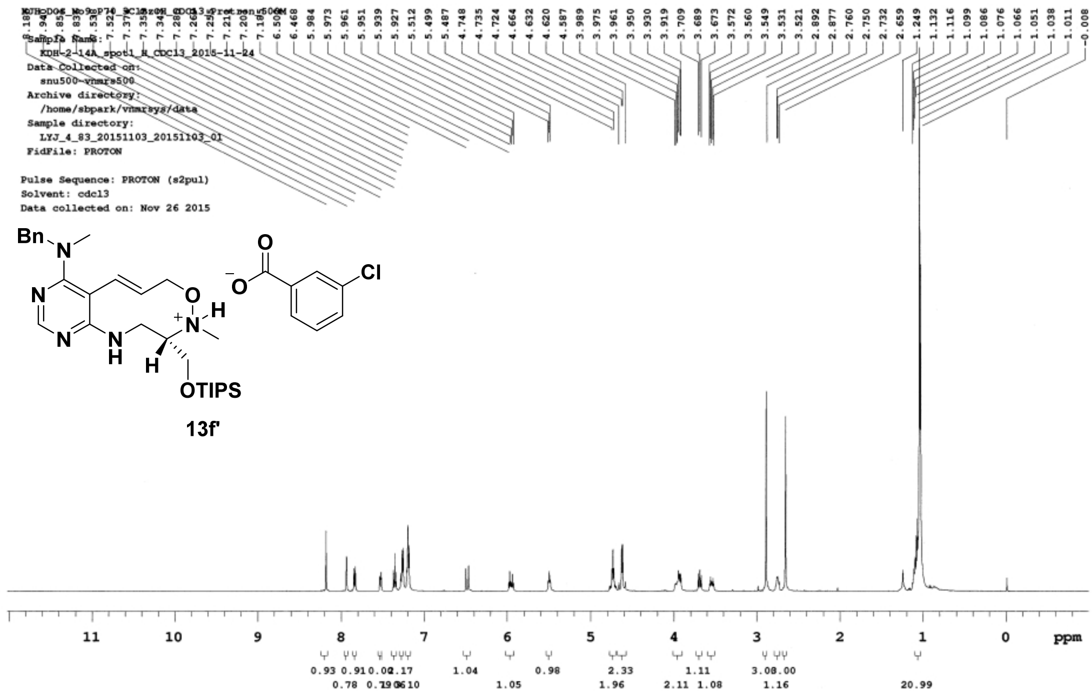

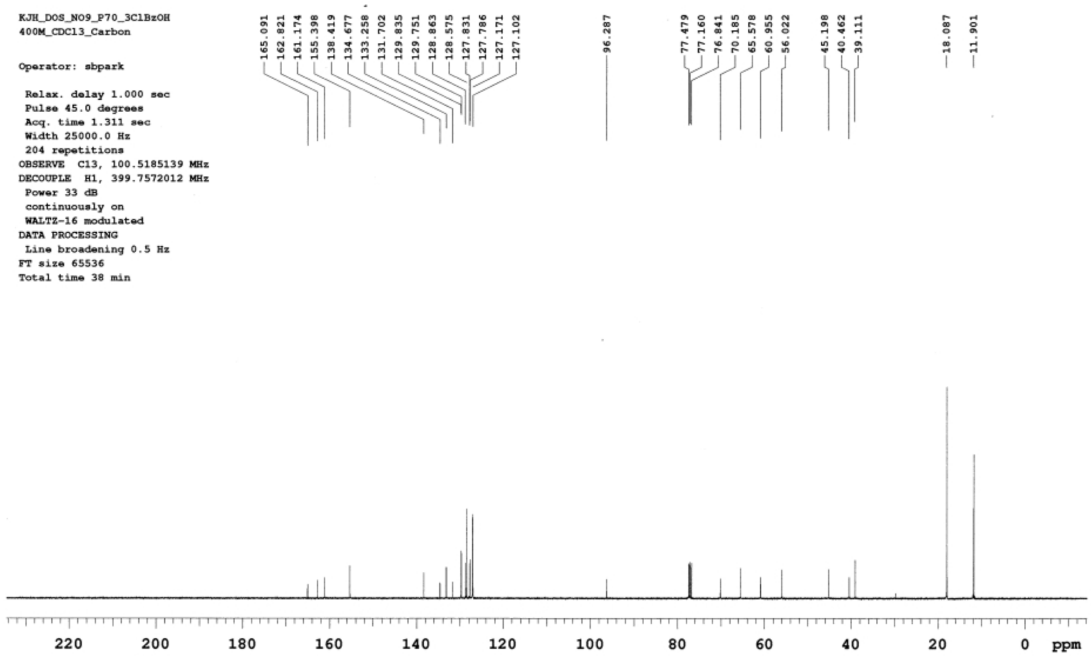

Supplementary Figure 54. ^{1}H and ^{13}C NMR spectra for **13f´**.

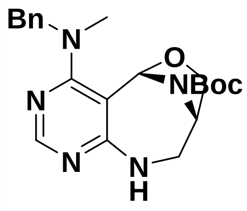

14f

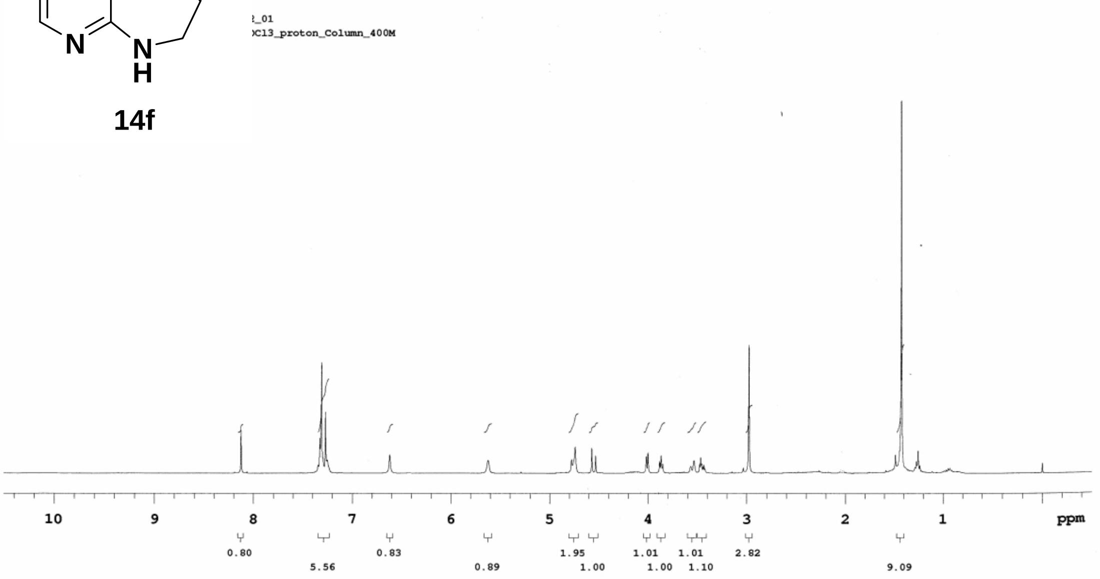

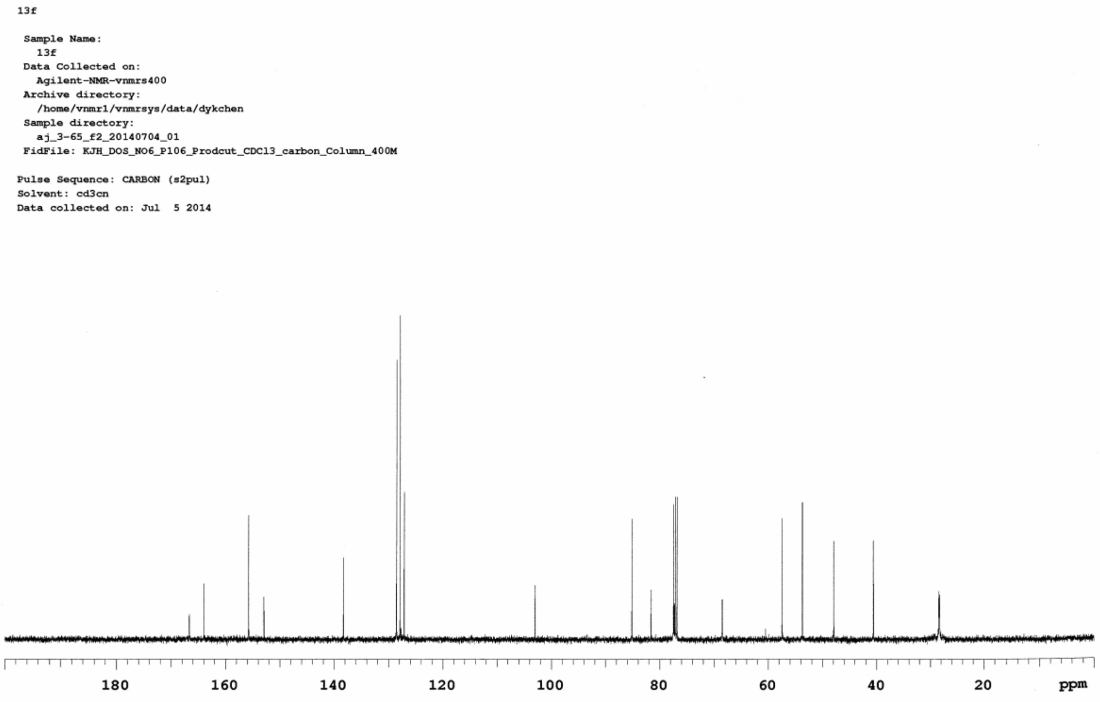

Supplementary Figure 55. ^{1}H and ^{13}C NMR spectra for **14f**.

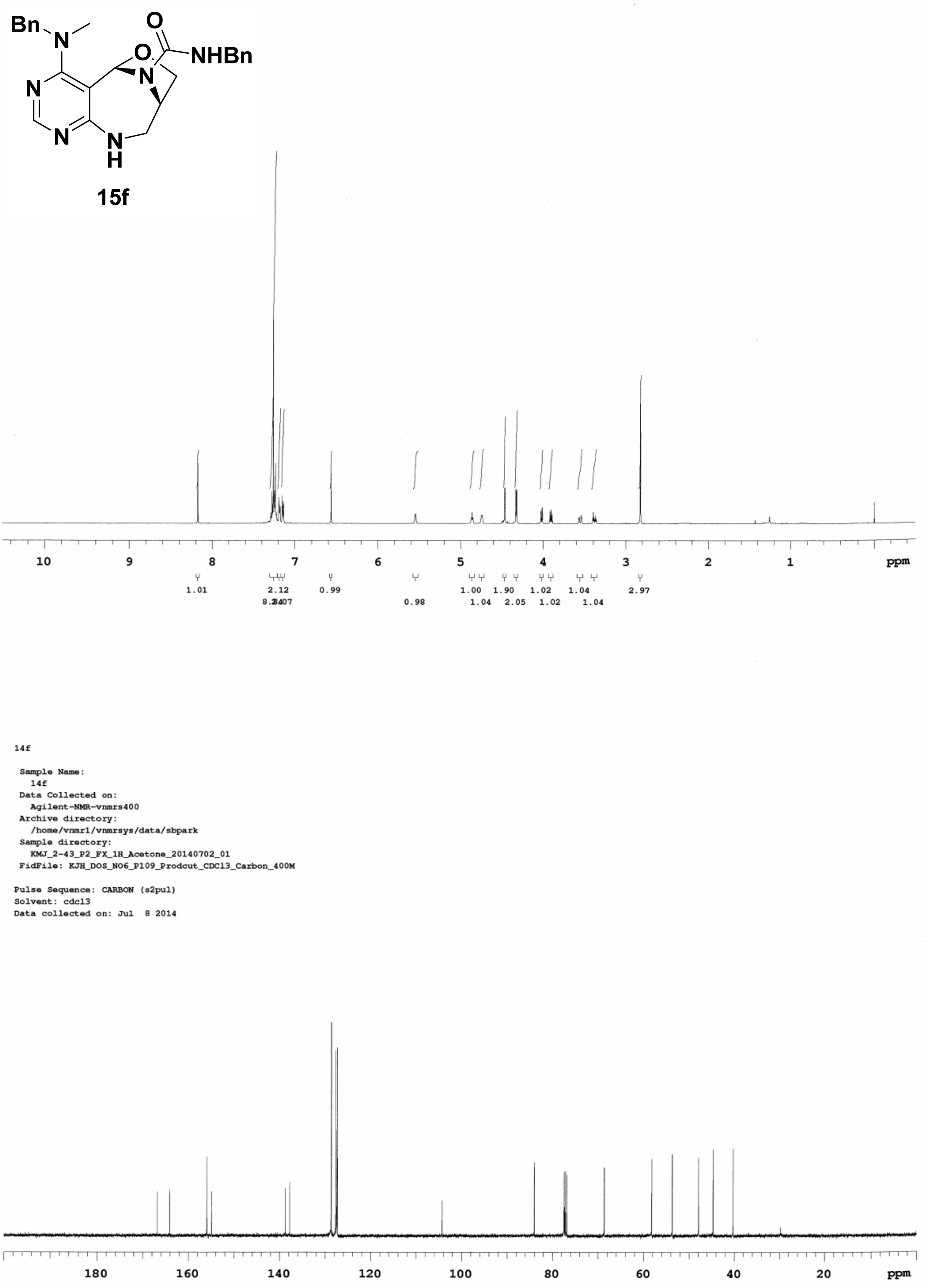

Supplementary Figure 56. ^{1}H and ^{13}C NMR spectra for **15f**.

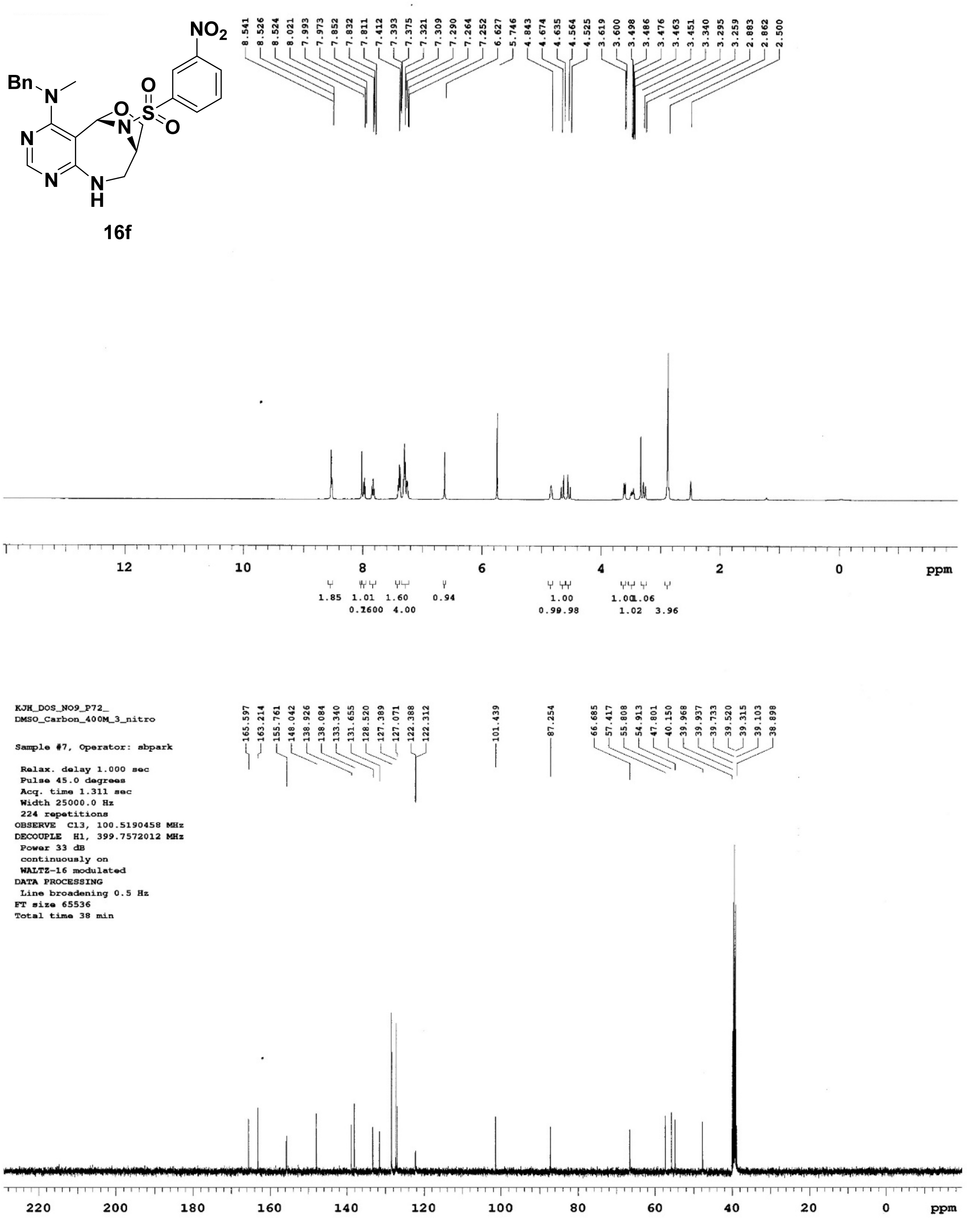

Supplementary Figure 57. ^{1}H and ^{13}C NMR spectra for **16f**.

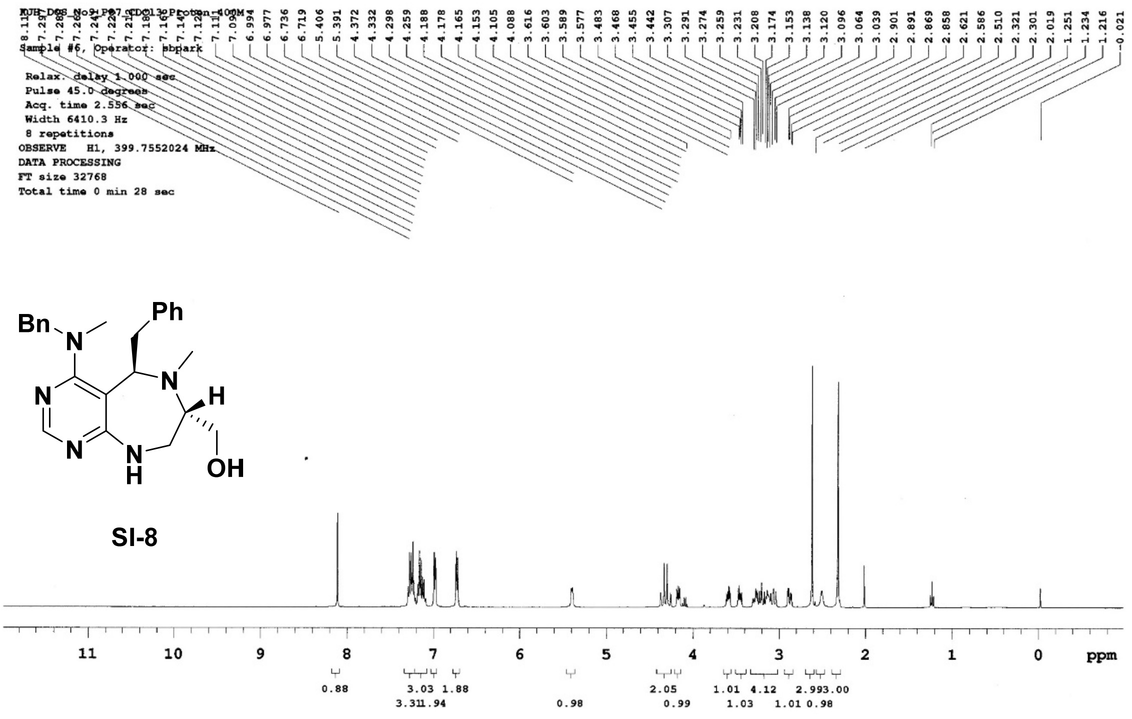

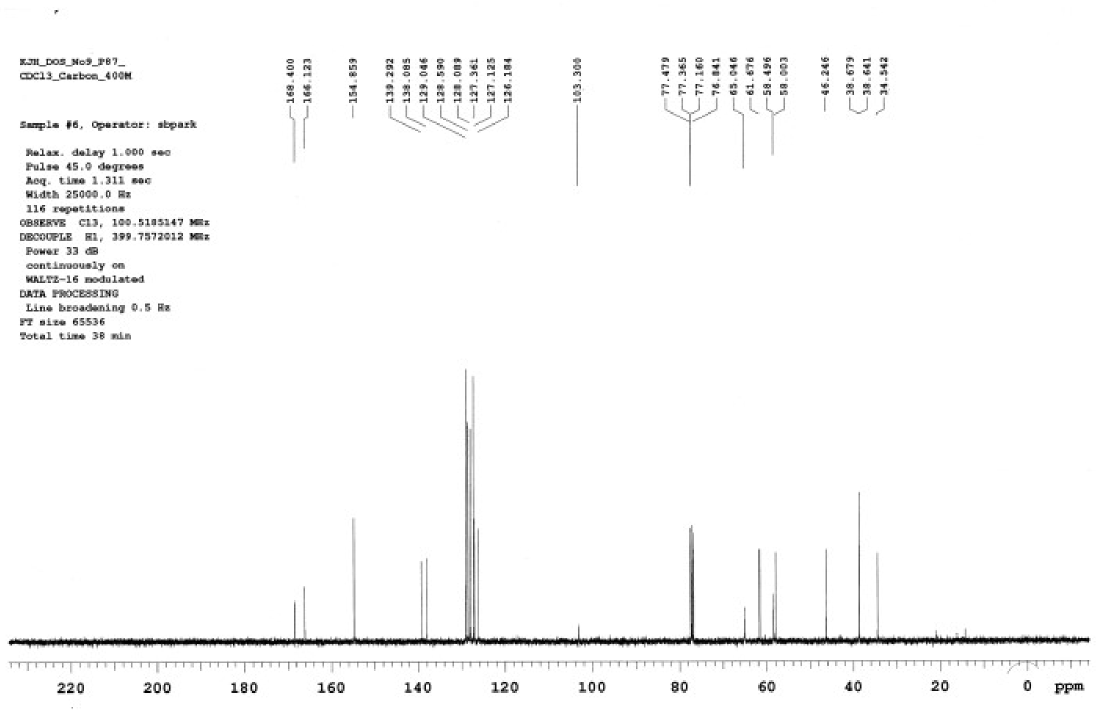

Supplementary Figure 58. ^{1}H and ^{13}C NMR spectra for **SI-8**.

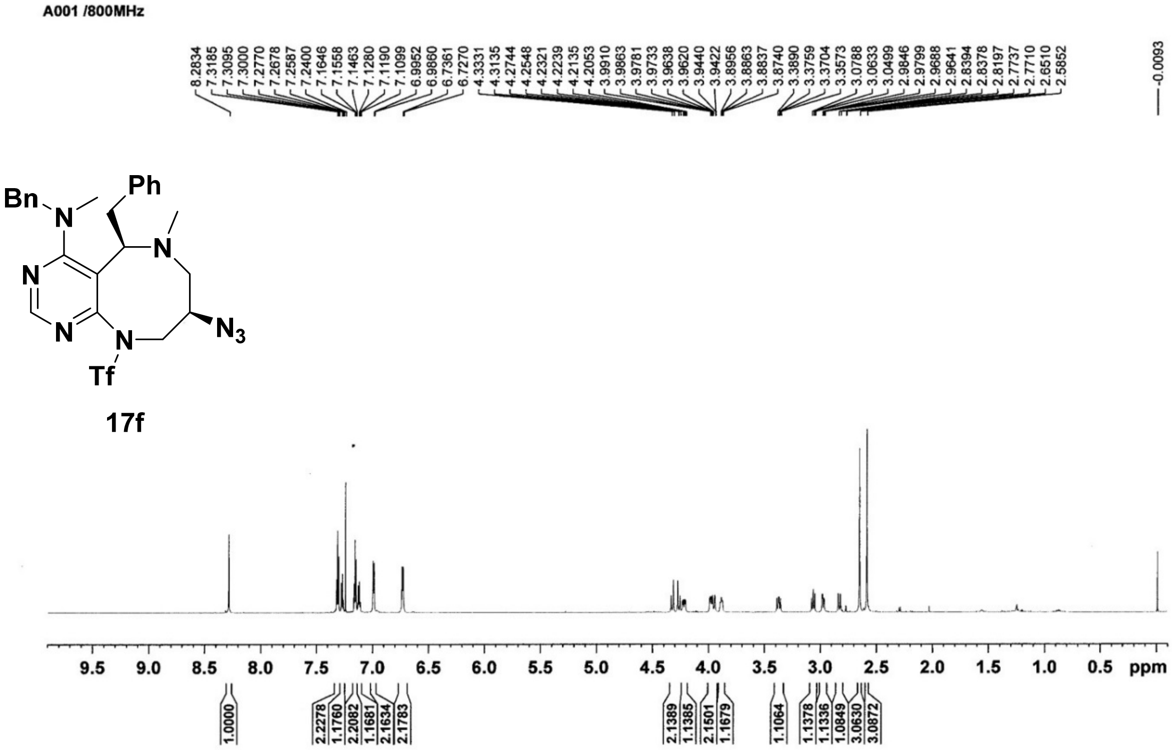

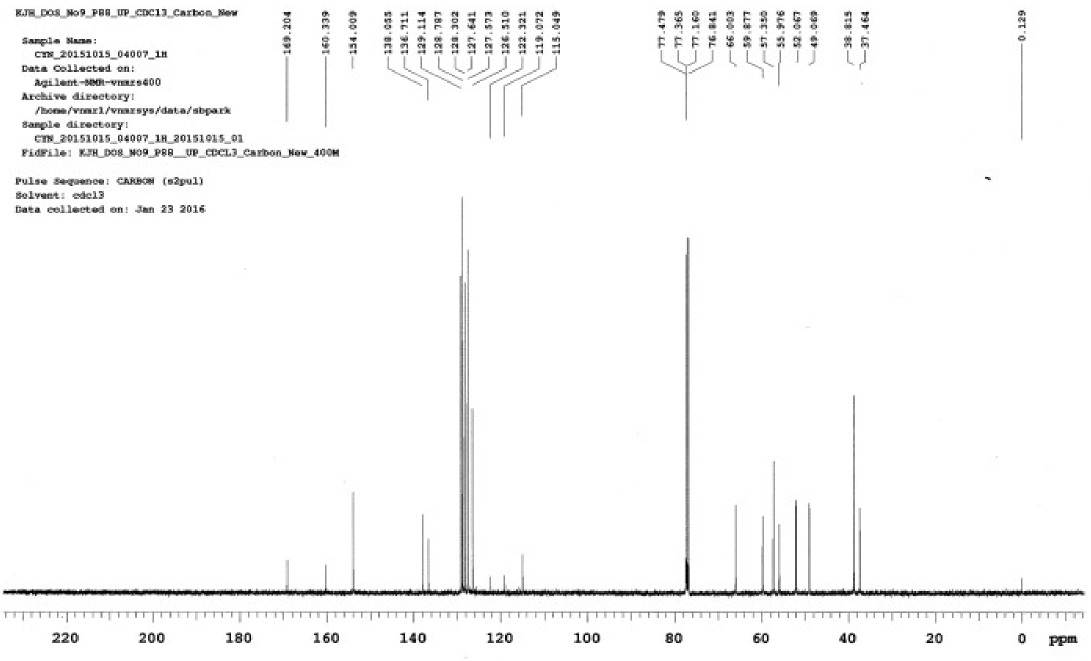

Supplementary Figure 59. ^{1}H and ^{13}C NMR spectra for **17f**.

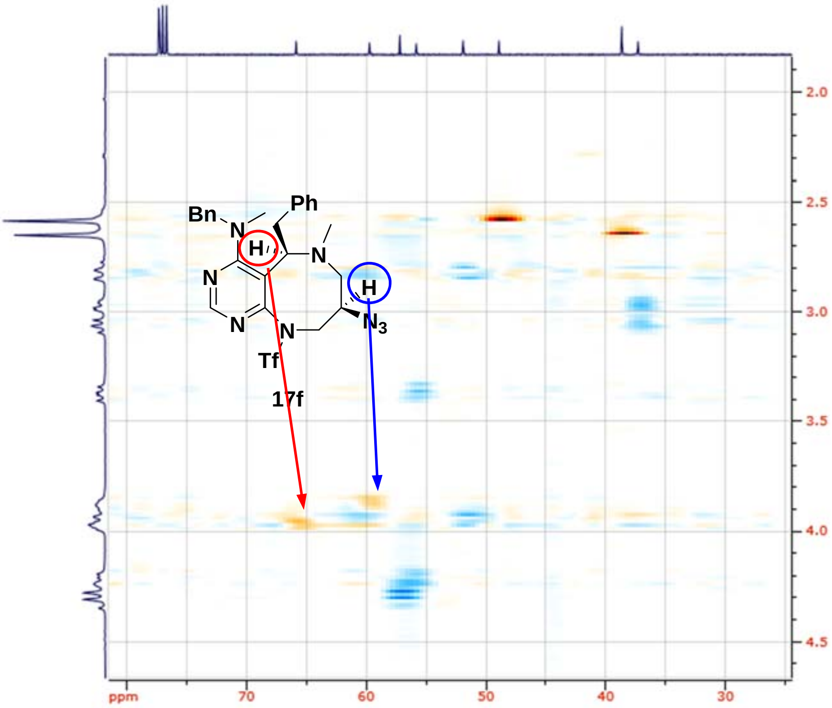
Bn
N
Ph
H
N
N
H
N
N
N3
Tf
17f
2.0
2.5
3.0
3.5
4.0
4.5
ppm
70
60
50
40
30

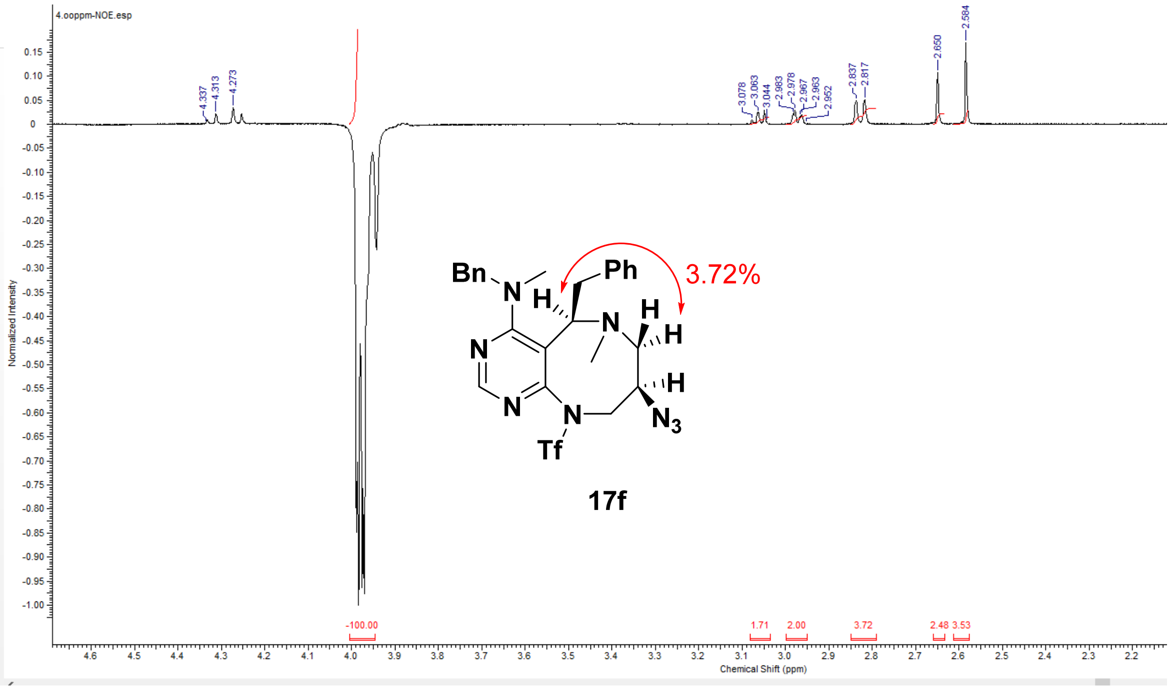
4.0ppm-NOE.esp
Normalized Intensity
Chemical Shift (ppm)
3.72%
Bn
N
H
Ph
N
H
H
H
N
N
N
N3
Tf
17f
-100.00
1.71
2.00
3.72
2.48
3.53

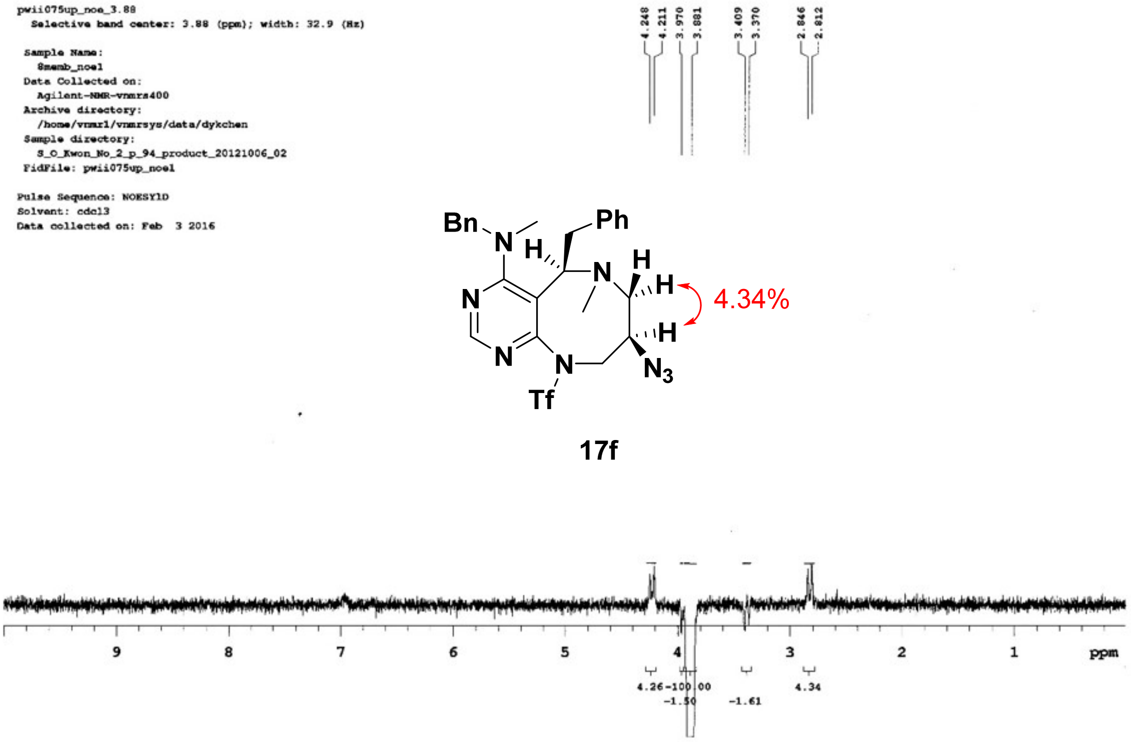

Supplementary Figure 60. HSQC and NOE spectra for **17f**.

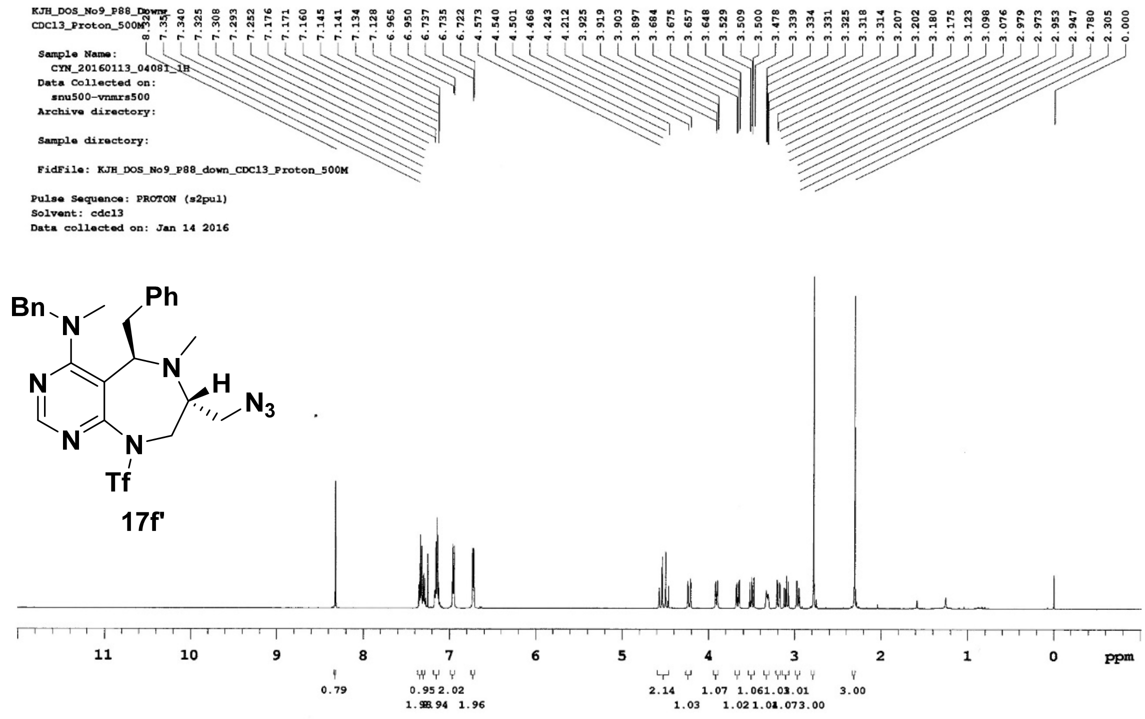

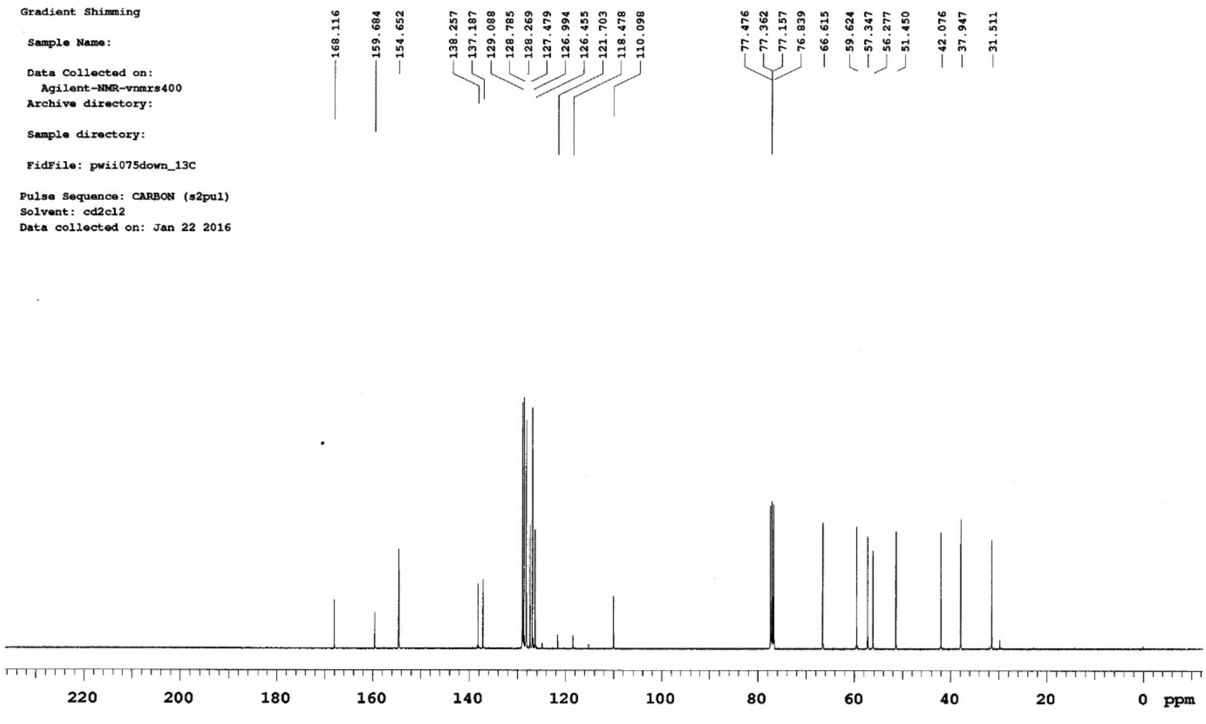

Supplementary Figure 61. ^{1}H and ^{13}C NMR spectra for **17f´**.

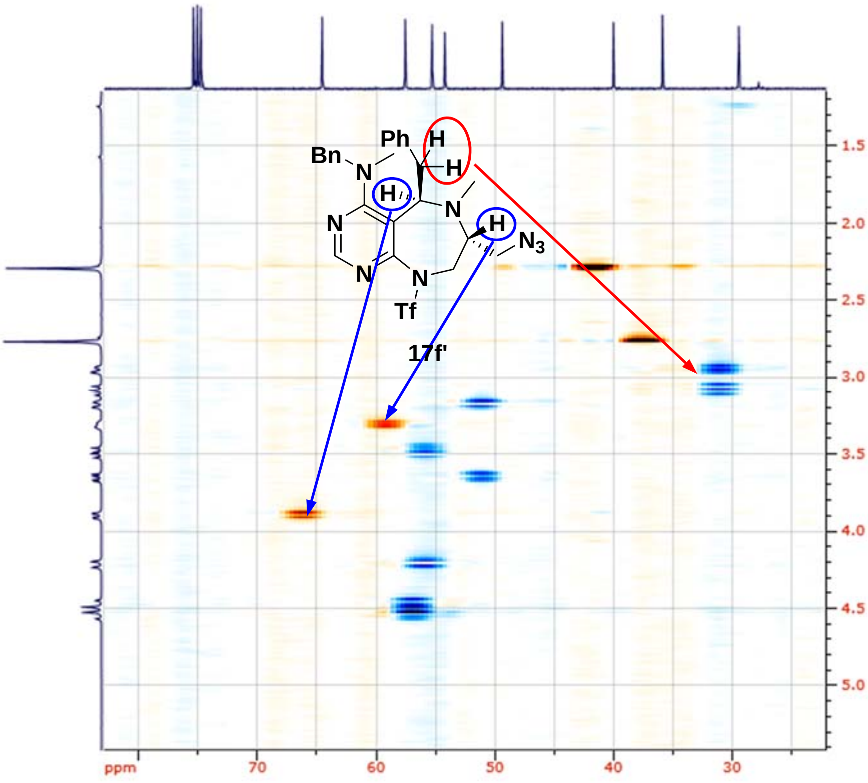

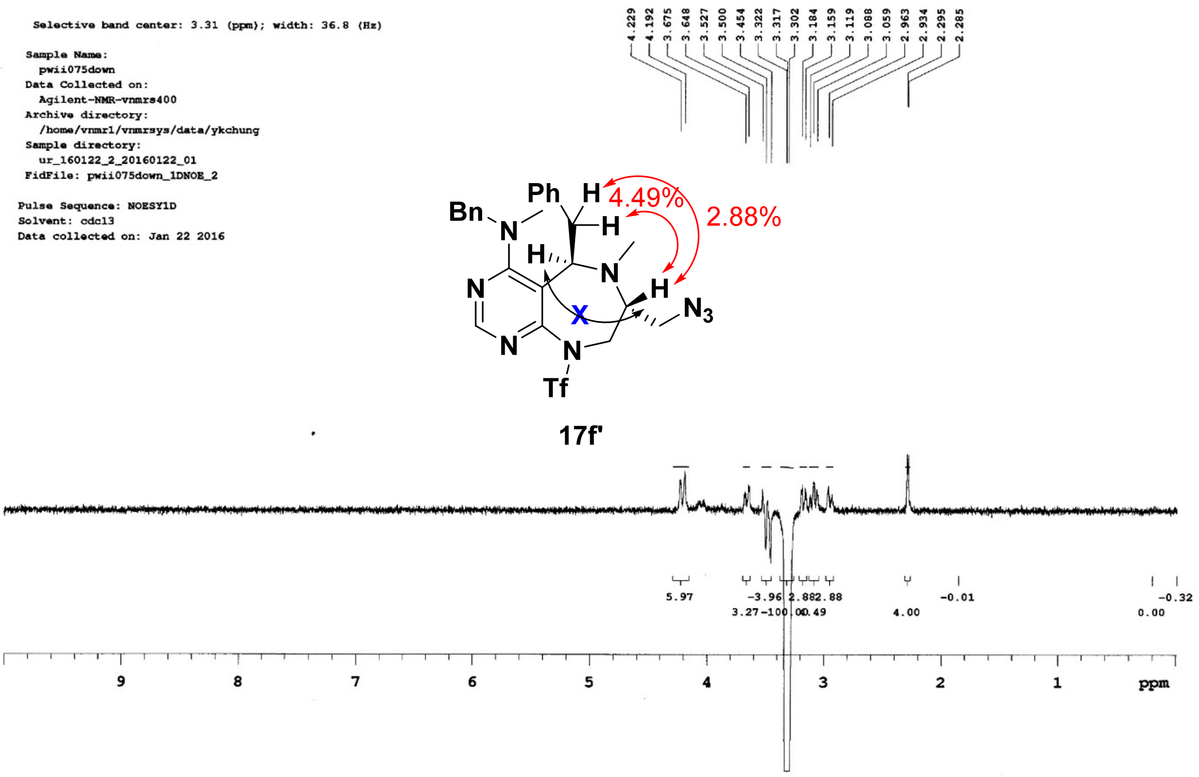

Supplementary Figure 62. HSQC and NOE spectra for **17f´**.

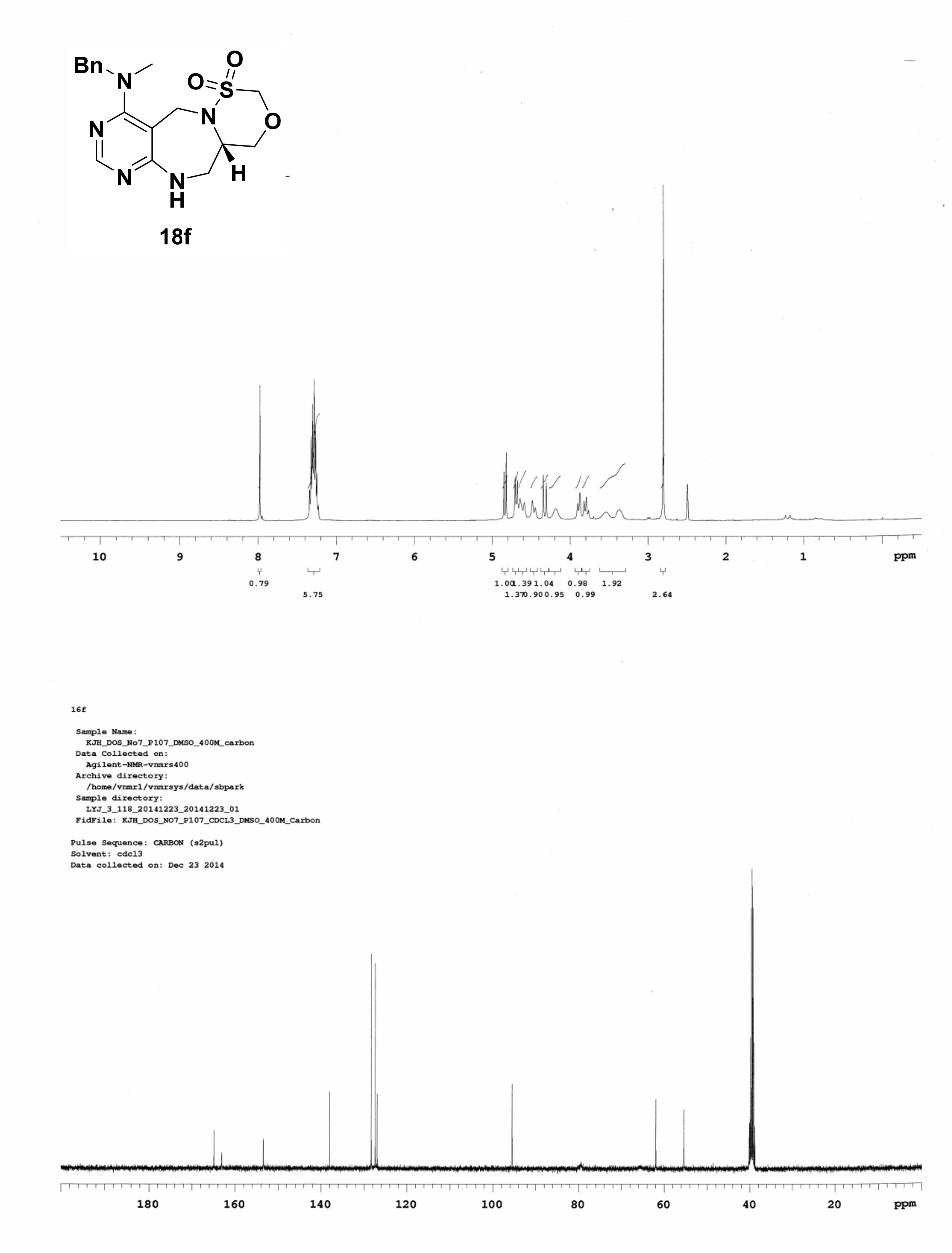

Supplementary Figure 63. ^{1}H and ^{13}C NMR spectra for **18f**.

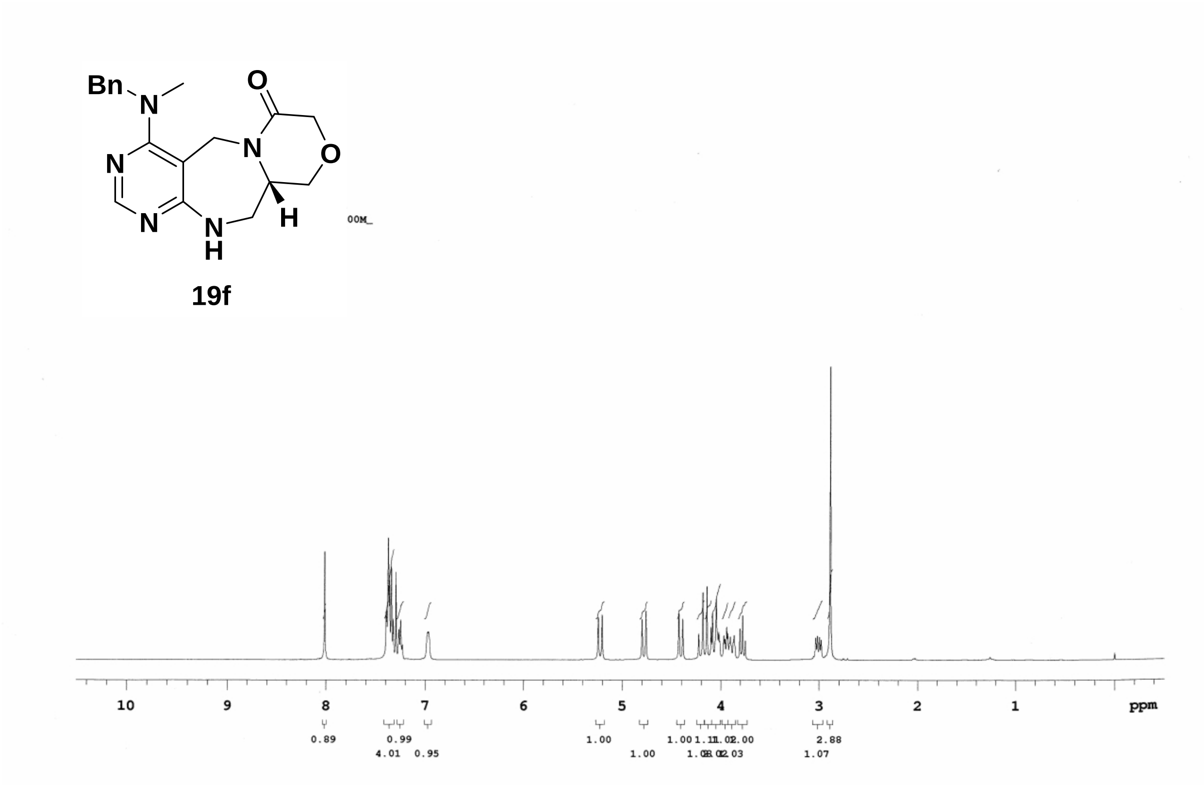

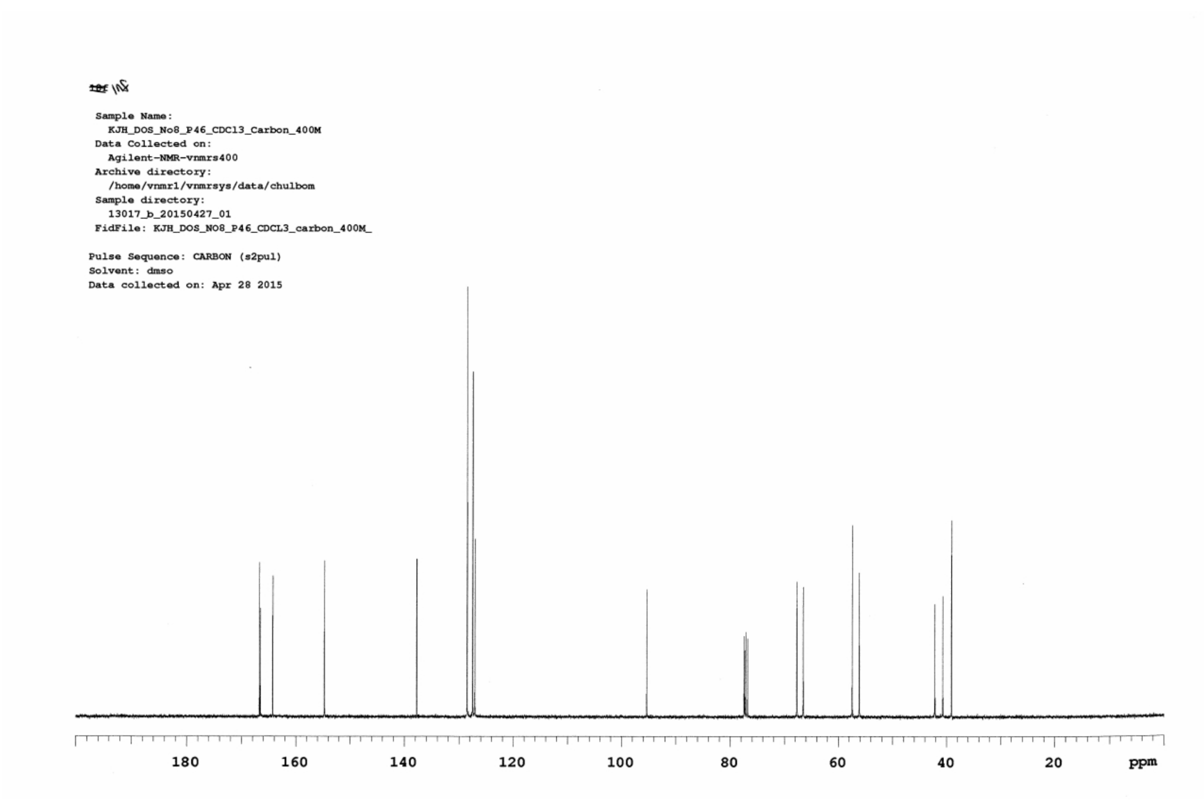

Supplementary Figure 64. ^{1}H and ^{13}C NMR spectra for **19f**.

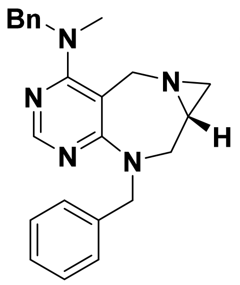

20f

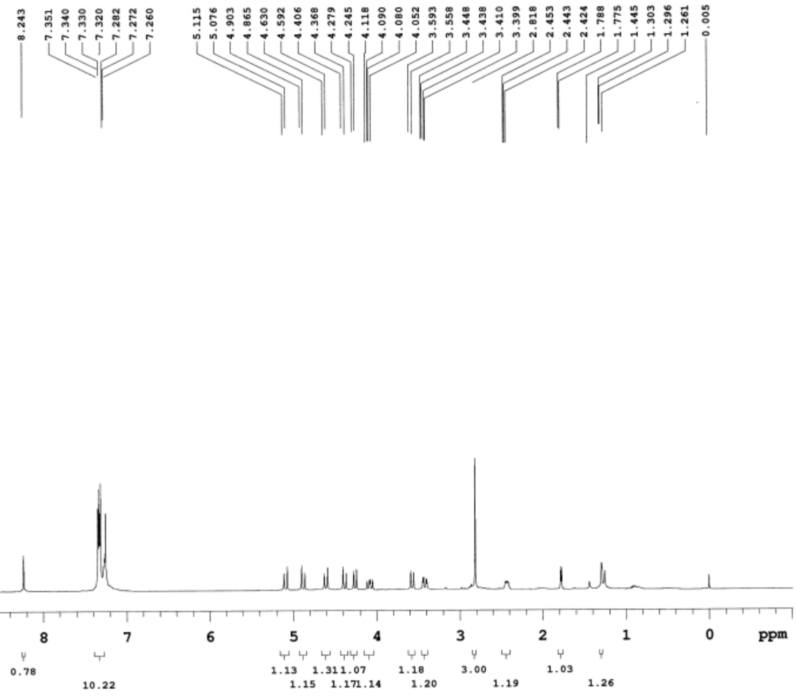

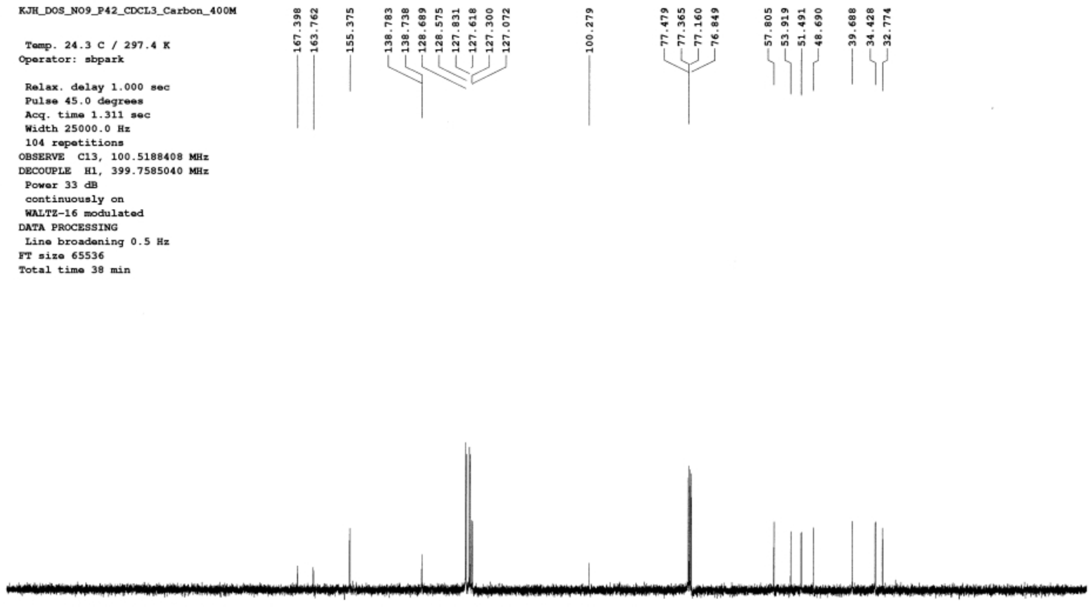

Supplementary Figure 65. ^{1}H and ^{13}C NMR spectra for **20f**.

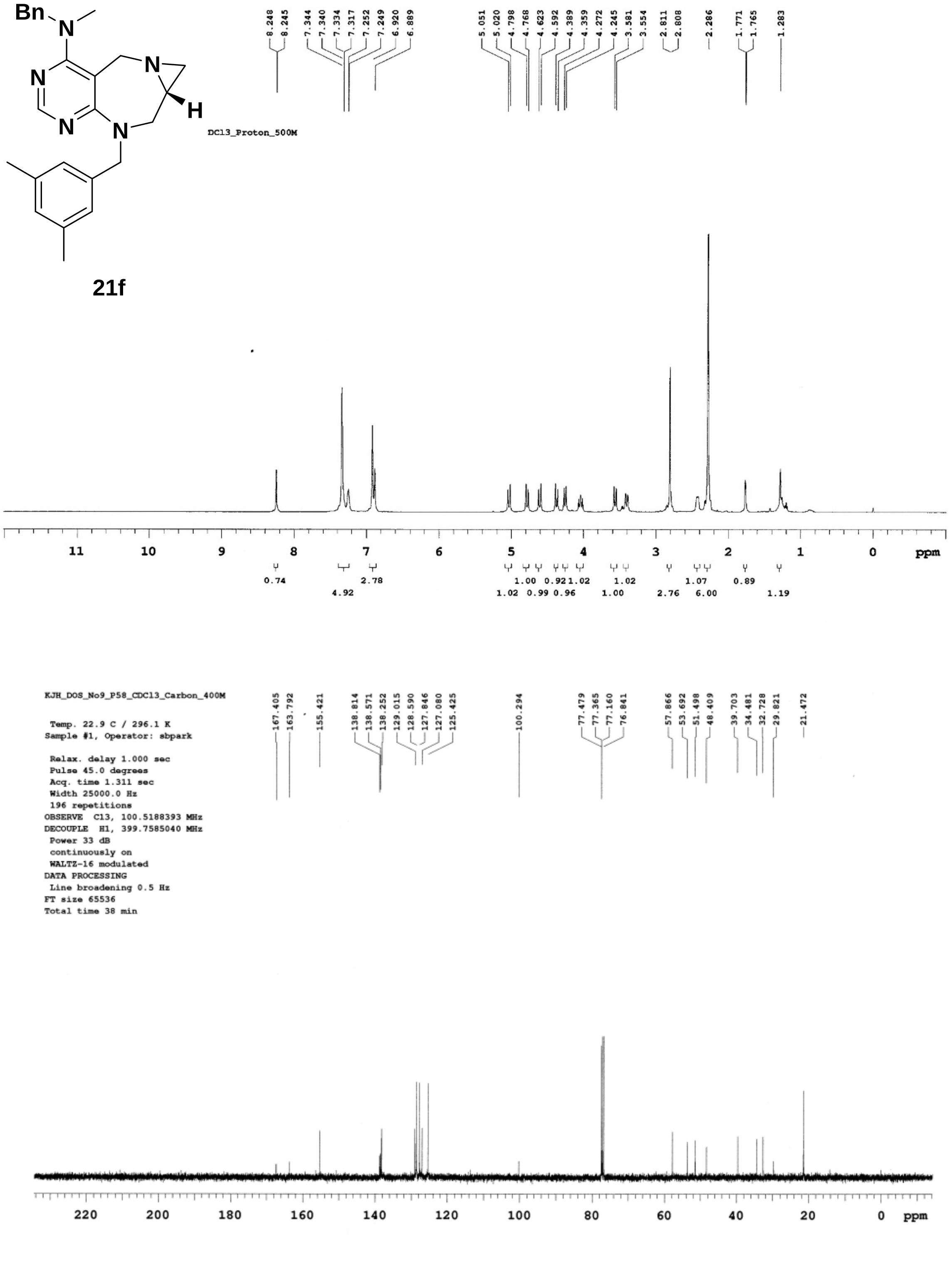

Supplementary Figure 66. ^{1}H and ^{13}C NMR spectra for **21f**.

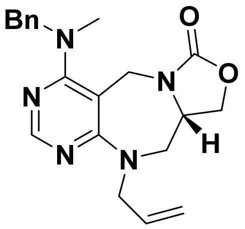

22f

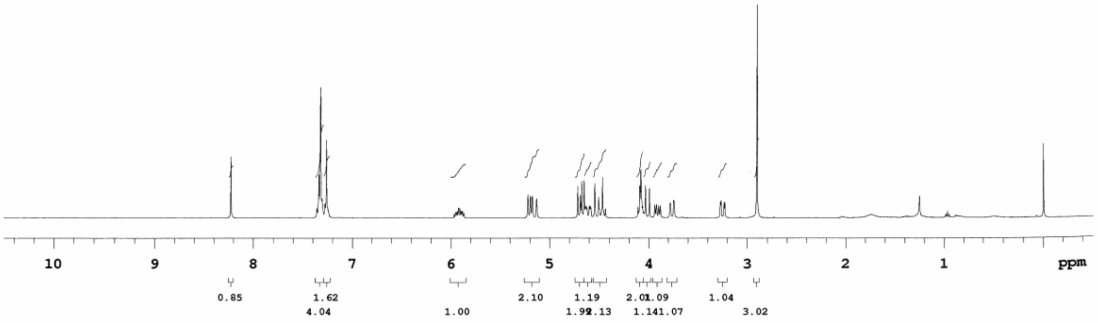

Sample Name:
19f
Data Collected on:
Agilent-NMR-vnmrs400
Archive directory:
/home/vnmr1/vnmrsys/data/sbpark
Sample directory:
JAE-V-9_re_13C_20140402_20150402_01
FidFile: CARBON_01

Pulse Sequence: CARBON (s2pul)
Solvent: cdcl3
Data collected on: Apr 2 2015

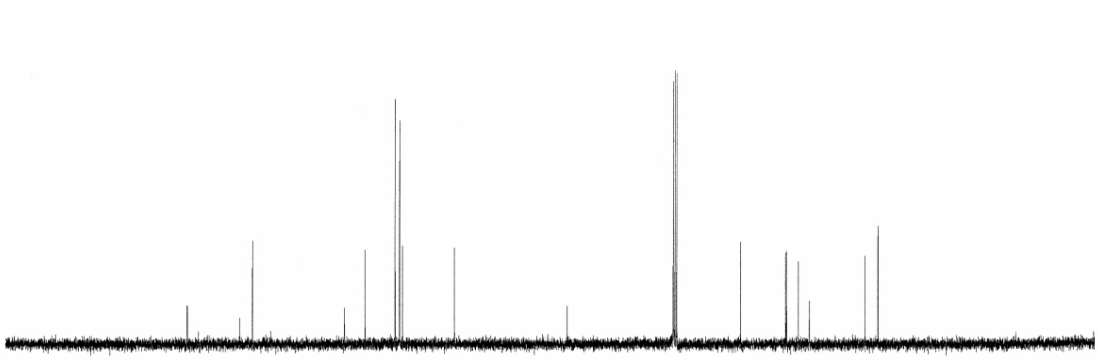

Supplementary Figure 67. ^{1}H and ^{13}C NMR spectra for **22f**.

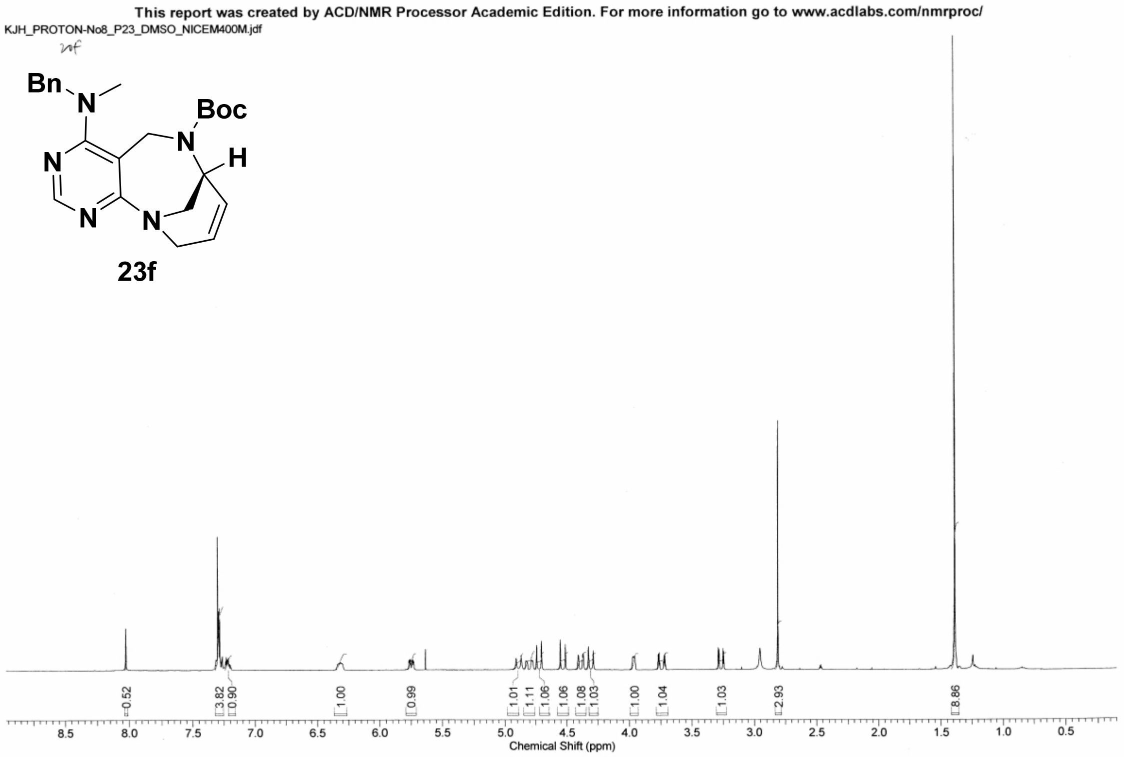

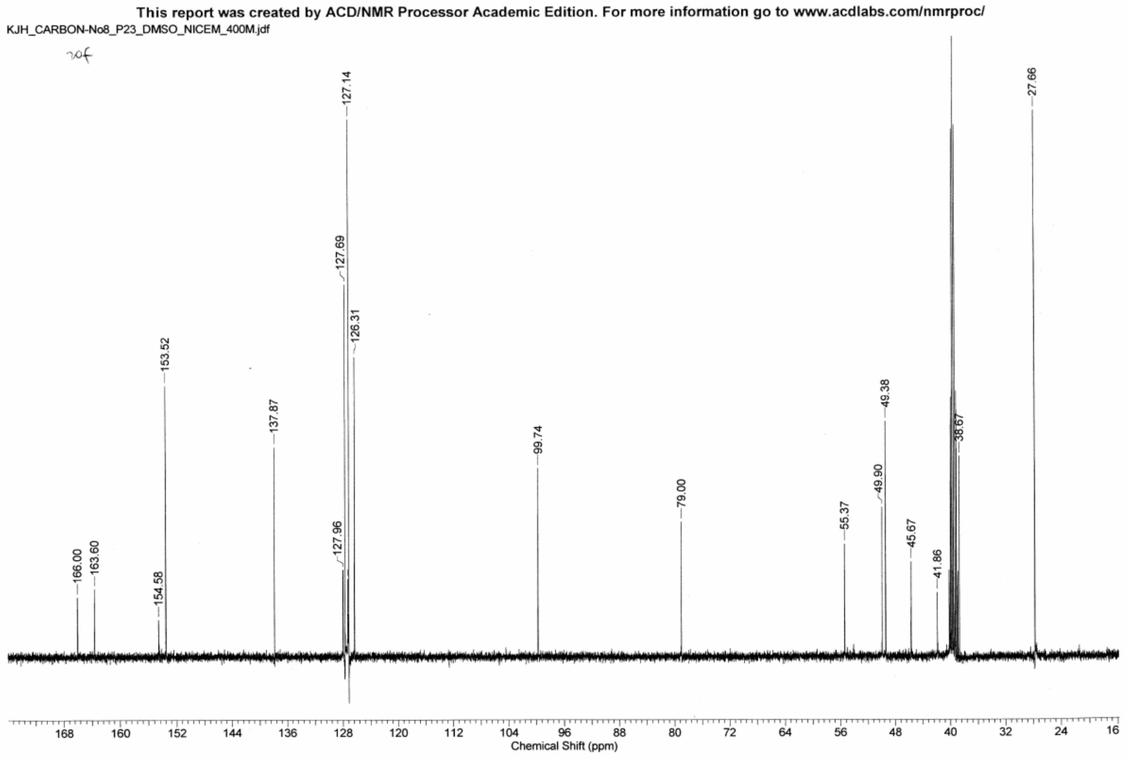

Supplementary Figure 68. ^{1}H and ^{13}C NMR spectra for **23f**.

Supplementary Table 1. Crystal data and structure refinement for **16f**

**Chemical formula**	$C_{23}H_{26}N_6O_6S$	
**Formula weight**	514.56	
**Temperature**	296(2) K	
**Wavelength**	0.71073 Å	
**Crystal size**	0.200 x 0.400 x 0.400 mm	
**Crystal habit**	colorless block	
**Crystal system**	monoclinic	
**Space group**	$P\ 1\ 2_1\ 1$	
**Unit cell dimensions**	a = 10.0193(5) Å	
	b = 11.4723(6) Å	α = 90°
	c = 10.4263(5) Å	β = 92.079(3)°
**Volume**	1197.66(10) Å^3	γ = 90°
**Z**	2	
**Density (calculated)**	1.427 g/cm^3	
**Absorption coefficient**	0.188 mm^{-1}	
**F(000)**	540	
**Theta range for data collection**	1.96 to 28.28°	
**Index ranges**	-13<=h<=13, -15<=k<=15, -13<=l<=13	
**Reflections collected**	20689	
**Independent reflections**	5558 [R(int) = 0.1027]	
**Coverage of independent reflections**	98.4%	
**Absorption correction**	multi-scan	
**Max. and min. transmission**	0.9630 and 0.9290	
**Refinement method**	Full-matrix least-squares on F^2	
**Refinement program**	SHELXL-2013 (Sheldrick, 2013)	
**Function minimized**	$\Sigma\ w(F_o^2 - F_c^2)^2$	

**Data / restraints / parameters**	5558 / 7 / 316
**Goodness-of-fit on F^2**	1.070
**Final R indices**	2086 data; I>2σ(I)<br>R1 = 0.1380, wR2 = 0.2464
	all data<br>R1 = 0.3170, wR2 = 0.3164
**Weighting scheme**	$w=1/[\sigma^2(F_o^2)+(0.1037P)^2+2.0087P]$<br>where $P=(F_o^2+2F_c^2)/3$
**Absolute structure parameter**	0.1(1)
**Largest diff. peak and hole**	0.563 and -0.341 eÅ^{-3}
**R.M.S. deviation from mean**	0.085 eÅ^{-3}

Supplementary Table 2. Atomic coordinates and equivalent isotropic atomic displacement parameters (Å^2) for **16f.** U(eq) is defined as one third of the trace of the orthogonalized U_{ij} tensor.

	**x/a**	**y/b**	**z/c**	**U(eq)**
C1	0.0654(11)	0.2662(8)	0.2825(11)	0.073(5)
C2	0.0756(9)	0.2179(9)	0.1610(11)	0.064(4)
C3	0.9707(10)	0.1529(9)	0.1075(7)	0.061(4)
C4	0.8555(9)	0.1360(9)	0.1755(8)	0.063(4)
C5	0.8452(10)	0.1843(10)	0.2971(8)	0.083(6)
C6	0.9502(12)	0.2494(10)	0.3506(8)	0.087(6)
C7	0.9039(13)	0.3097(11)	0.8665(12)	0.048(3)
C8	0.7896(15)	0.3606(14)	0.9460(14)	0.064(4)
C9	0.7383(13)	0.1742(13)	0.8667(14)	0.062(4)
C10	0.9085(16)	0.3633(13)	0.7335(15)	0.067(4)
C11	0.6910(13)	0.2706(12)	0.6512(12)	0.045(3)
C12	0.6595(13)	0.1888(14)	0.7453(12)	0.053(4)
C13	0.5495(16)	0.1137(14)	0.7190(14)	0.065(4)
C14	0.510(2)	0.1962(16)	0.5265(16)	0.081(6)
C15	0.4248(16)	0.9415(14)	0.7672(15)	0.076(5)
C16	0.4715(13)	0.0786(11)	0.9379(13)	0.051(3)
C17	0.5295(13)	0.0088(13)	0.0482(14)	0.051(4)
C18	0.6162(14)	0.9193(11)	0.0347(13)	0.052(4)
C19	0.6710(15)	0.8579(15)	0.1429(15)	0.069(4)
C20	0.6369(19)	0.8892(17)	0.2628(15)	0.081(6)
C21	0.5471(17)	0.9817(18)	0.2765(18)	0.080(5)
C22	0.4972(17)	0.0382(16)	0.1683(18)	0.077(5)

N1	0.1780(19)	0.3272(16)	0.3397(16)	0.099(6)
N2	0.8809(11)	0.1862(9)	0.8580(10)	0.052(3)
N3	0.6145(14)	0.2749(11)	0.5388(11)	0.067(4)
N4	0.4777(12)	0.1196(11)	0.6096(13)	0.065(3)
N5	0.7864(13)	0.3494(11)	0.6539(13)	0.073(4)
N6	0.5032(10)	0.0339(10)	0.8133(11)	0.052(3)
O1	0.1590(19)	0.3801(18)	0.4392(17)	0.161(7)
O2	0.2791(15)	0.3318(15)	0.2851(12)	0.121(6)
O3	0.9228(11)	0.9869(8)	0.9465(9)	0.068(3)
O4	0.1082(9)	0.1229(9)	0.9126(8)	0.062(3)
O5	0.7025(8)	0.2633(8)	0.9584(8)	0.059(3)
S1	0.9786(4)	0.1030(3)	0.9515(3)	0.0570(10)
O1S	0.2246(13)	0.0545(10)	0.5055(11)	0.087(3)
C1S	0.1250(19)	0.0840(18)	0.5891(17)	0.104(7)

Supplementary Table 3. Bond lengths (Å) and Bond angles (°) for **16f.**

C1-C2	1.39	C1-C6	1.39
C1-N1	1.439(15)	C2-C3	1.39
C2-H2	0.93	C3-C4	1.39
C3-S1	1.728(8)	C4-C5	1.39
C4-H4	0.93	C5-C6	1.39
C5-H5	0.93	C6-H6	0.93
C7-N2	1.438(15)	C7-C10	1.520(19)
C7-C8	1.552(18)	C7-H7	0.98
C8-O5	1.426(16)	C8-H8A	0.97
C8-H8B	0.97	C9-N2	1.442(16)
C9-O5	1.453(15)	C9-C12	1.477(19)
C9-H9	0.98	C10-N5	1.462(18)
C10-H10A	0.97	C10-H10B	0.97
C11-N5	1.315(17)	C11-N3	1.378(16)
C11-C12	1.402(19)	C12-C13	1.42(2)
C13-N4	1.328(17)	C13-N6	1.433(18)
C14-N4	1.28(2)	C14-N3	1.39(2)
C14-H14	0.93	C15-N6	1.395(17)
C15-H15A	0.96	C15-H15B	0.96
C15-H15C	0.96	C16-N6	1.443(16)
C16-C17	1.501(17)	C16-H16A	0.97
C16-H16B	0.97	C17-C22	1.35(2)
C17-C18	1.355(17)	C18-C19	1.423(19)
C18-H18	0.93	C19-C20	1.36(2)
C19-H19	0.93	C20-C21	1.40(2)
C20-H20	0.93	C21-C22	1.38(2)

C21-H21	0.93	C22-H22	0.93
N1-O2	1.181(18)	N1-O1	1.22(2)
N2-S1	1.658(12)	N5-H5A	0.86
O3-S1	1.445(10)	O4-S1	1.393(9)
O1S-C1S	1.391(19)	O1S-H1S	0.82
C1S-H1S1	0.96	C1S-H1S2	0.96
C1S-H1S3	0.96		
C2-C1-C6	120.0	C2-C1-N1	119.2(12)
C6-C1-N1	120.6(11)	C3-C2-C1	120.0
C3-C2-H2	120.0	C1-C2-H2	120.0
C2-C3-C4	120.0	C2-C3-S1	119.7(6)
C4-C3-S1	120.1(6)	C5-C4-C3	120.0
C5-C4-H4	120.0	C3-C4-H4	120.0
C6-C5-C4	120.0	C6-C5-H5	120.0
C4-C5-H5	120.0	C5-C6-C1	120.0
C5-C6-H6	120.0	C1-C6-H6	120.0
N2-C7-C10	110.6(11)	N2-C7-C8	106.5(11)
C10-C7-C8	112.5(12)	N2-C7-H7	109.0
C10-C7-H7	109.0	C8-C7-H7	109.0
O5-C8-C7	102.7(11)	O5-C8-H8A	111.2
C7-C8-H8A	111.2	O5-C8-H8B	111.2
C7-C8-H8B	111.2	H8A-C8-H8B	109.1
N2-C9-O5	104.1(10)	N2-C9-C12	115.8(12)
O5-C9-C12	110.3(12)	N2-C9-H9	108.8
O5-C9-H9	108.8	C12-C9-H9	108.8
N5-C10-C7	114.9(12)	N5-C10-H10A	108.5
C7-C10-H10A	108.5	N5-C10-H10B	108.5
C7-C10-H10B	108.5	H10A-C10-H10B	107.5

N5-C11-N3	112.0(12)	N5-C11-C12	128.9(13)
N3-C11-C12	119.1(13)	C11-C12-C13	117.7(12)
C11-C12-C9	123.3(13)	C13-C12-C9	119.0(14)
N4-C13-C12	121.9(14)	N4-C13-N6	116.3(14)
C12-C13-N6	121.6(12)	N4-C14-N3	126.2(17)
N4-C14-H14	116.9	N3-C14-H14	116.9
N6-C15-H15A	109.5	N6-C15-H15B	109.5
H15A-C15-H15B	109.5	N6-C15-H15C	109.5
H15A-C15-H15C	109.5	H15B-C15-H15C	109.5
N6-C16-C17	114.1(11)	N6-C16-H16A	108.7
C17-C16-H16A	108.7	N6-C16-H16B	108.7
C17-C16-H16B	108.7	H16A-C16-H16B	107.6
C22-C17-C18	117.6(15)	C22-C17-C16	118.6(14)
C18-C17-C16	123.8(12)	C17-C18-C19	121.5(13)
C17-C18-H18	119.2	C19-C18-H18	119.2
C20-C19-C18	119.8(16)	C20-C19-H19	120.1
C18-C19-H19	120.1	C19-C20-C21	118.6(15)
C19-C20-H20	120.7	C21-C20-H20	120.7
C22-C21-C20	119.2(16)	C22-C21-H21	120.4
C20-C21-H21	120.4	C17-C22-C21	123.3(17)
C17-C22-H22	118.3	C21-C22-H22	118.3
O2-N1-O1	123.3(17)	O2-N1-C1	119.7(17)
O1-N1-C1	116.7(18)	C7-N2-C9	104.3(11)
C7-N2-S1	116.2(9)	C9-N2-S1	118.2(9)
C11-N3-C14	116.8(13)	C14-N4-C13	118.3(16)
C11-N5-C10	132.8(12)	C11-N5-H5A	113.6
C10-N5-H5A	113.6	C15-N6-C13	116.2(12)
C15-N6-C16	116.2(12)	C13-N6-C16	118.5(11)

C8-O5-C9	109.0(10)	O4-S1-O3	120.2(6)
O4-S1-N2	105.7(5)	O3-S1-N2	106.9(6)
O4-S1-C3	107.2(5)	O3-S1-C3	107.9(6)
N2-S1-C3	108.5(5)	C1S-O1S-H1S	109.5
O1S-C1S-H1S1	109.5	O1S-C1S-H1S2	109.5
H1S1-C1S-H1S2	109.5	O1S-C1S-H1S3	109.5
H1S1-C1S-H1S3	109.5	H1S2-C1S-H1S3	109.5

Supplementary Table 4. Anisotropic atomic displacement parameters ($Å^2$) for **16f.** The anisotropic atomic displacement factor exponent takes the form: $-2\pi^2[h^2 a^{*2} U_{11} + ... + 2 h k a^* b^* U_{12}]$

	U_{11}	U_{22}	U_{33}	U_{23}	U_{13}	U_{12}
C1	0.057(11)	0.085(12)	0.077(12)	0.020(10)	-0.010(9)	-0.004(10)
C2	0.060(10)	0.077(11)	0.056(10)	0.015(8)	-0.007(8)	0.013(9)
C3	0.036(8)	0.089(11)	0.058(9)	0.030(8)	-0.012(7)	-0.011(7)
C4	0.068(9)	0.083(11)	0.036(7)	0.014(7)	-0.009(7)	-0.002(8)
C5	0.106(13)	0.108(13)	0.036(7)	-0.012(8)	0.020(9)	0.068(11)
C6	0.086(13)	0.120(15)	0.050(9)	-0.020(10)	-0.060(10)	0.002(11)
C7	0.050(8)	0.044(8)	0.049(8)	0.007(7)	0.002(6)	-0.018(6)
C8	0.063(10)	0.062(9)	0.067(10)	-0.012(8)	0.002(8)	0.023(9)
C9	0.053(9)	0.066(10)	0.069(10)	-0.026(8)	0.016(8)	-0.018(8)
C10	0.080(12)	0.041(8)	0.082(12)	-0.009(8)	0.028(10)	0.003(8)
C11	0.051(8)	0.050(8)	0.036(7)	-0.003(7)	0.003(7)	0.013(7)
C12	0.031(7)	0.089(11)	0.035(7)	-0.015(7)	-0.027(6)	0.016(8)
C13	0.080(11)	0.059(10)	0.054(9)	-0.014(9)	-0.012(9)	0.035(10)
C14	0.108(15)	0.070(11)	0.068(11)	-0.015(10)	0.021(11)	0.062(12)
C15	0.076(5)	0.078(5)	0.074(5)	-0.001(3)	0.000(3)	-0.002(3)
C16	0.046(7)	0.050(9)	0.057(8)	0.003(7)	-0.006(7)	0.003(7)
C17	0.030(7)	0.076(11)	0.047(8)	-0.010(8)	-0.012(6)	0.002(7)
C18	0.059(9)	0.047(8)	0.050(8)	-0.013(7)	-0.013(7)	0.004(7)
C19	0.061(10)	0.069(10)	0.076(12)	0.023(9)	-0.022(9)	-0.013(9)
C20	0.085(13)	0.113(16)	0.043(9)	0.018(10)	-0.013(9)	-0.032(12)
C21	0.060(11)	0.109(14)	0.072(12)	-0.018(11)	0.023(10)	0.001(11)
C22	0.078(12)	0.074(11)	0.077(12)	-0.002(11)	-0.021(11)	-0.015(10)
N1	0.102(13)	0.137(15)	0.058(10)	0.009(10)	-0.020(9)	-0.084(11)

	U_{11}	U_{22}	U_{33}	U_{23}	U_{13}	U_{12}
N2	0.056(7)	0.045(7)	0.056(7)	0.001(5)	0.027(6)	-0.031(6)
N3	0.085(9)	0.060(8)	0.057(8)	0.006(7)	-0.009(7)	-0.015(8)
N4	0.067(8)	0.057(7)	0.071(8)	0.001(7)	-0.004(7)	0.011(7)
N5	0.078(10)	0.064(9)	0.076(9)	0.030(7)	-0.013(8)	-0.030(8)
N6	0.043(7)	0.054(7)	0.056(7)	0.001(6)	-0.027(6)	-0.010(6)
O1	0.194(18)	0.175(16)	0.109(13)	-0.028(12)	-0.075(12)	-0.033(14)
O2	0.101(11)	0.178(15)	0.083(9)	0.021(9)	0.006(8)	-0.067(10)
O3	0.082(7)	0.055(6)	0.068(7)	-0.003(5)	0.007(6)	-0.004(6)
O4	0.071(6)	0.071(7)	0.046(5)	0.006(5)	0.023(5)	-0.004(6)
O5	0.050(6)	0.058(6)	0.070(6)	-0.021(5)	0.003(5)	-0.003(5)
S1	0.051(2)	0.062(2)	0.058(2)	0.0058(19)	-0.0042(18)	-0.002(2)
O1S	0.099(9)	0.098(8)	0.063(7)	-0.019(7)	0.018(7)	-0.012(8)
C1S	0.114(15)	0.113(17)	0.086(13)	0.023(12)	0.003(12)	-0.060(14)

Supplementary Table 5. Hydrogen atomic coordinates and isotropic atomic displacement parameters (Å^2) for **16f**.

	**x/a**	**y/b**	**z/c**	**U(eq)**
H2	1.1527	0.2292	1.1155	0.077
H4	0.7852	0.0925	1.1397	0.075
H5	0.7681	0.1731	1.3426	0.099
H6	0.9433	0.2817	1.4319	0.104
H7	0.9894	0.3236	0.9126	0.057
H8A	0.8229	0.3878	1.0293	0.077
H8B	0.7450	0.4245	0.9011	0.077
H9	0.7188	0.0974	0.9027	0.075
H10A	0.9274	0.4459	0.7425	0.081
H10B	0.9820	0.3284	0.6890	0.081
H14	0.4580	0.1991	0.4507	0.098
H15A	0.3333	-0.0339	0.7582	0.114
H15B	0.4316	-0.1225	0.8264	0.114
H15C	0.4554	-0.0828	0.6852	0.114
H16A	0.3752	0.0808	0.9442	0.061
H16B	0.5041	0.1581	0.9453	0.061
H18	0.6403	-0.1026	0.9528	0.063
H19	0.7301	-0.2034	1.1314	0.083
H20	0.6724	-0.1498	1.3344	0.097
H21	0.5215	0.0046	1.3575	0.096
H22	0.4382	0.0998	1.1788	0.092
H5A	0.7745	0.4035	0.5976	0.088
H1S	0.2565	0.1140	0.4756	0.13
H1S1	0.1324	0.0355	0.6640	0.156

	x/a	y/b	z/c	U(eq)
H1S2	0.0391	0.0726	0.5471	0.156
H1S3	0.1348	0.1642	0.6138	0.156

Supplementary Table 6. Normalized PMI ratios of pDOS library, natural products and FDA-approved drugs embedded with pyrimidine moieties.

**pDOS Library**			**Natural products**[2]		
Compound	npr1	npr2	Compound	npr1	npr2
**2a**	0.431687	0.954947	Actinonin	0.314978	0.841802
**3b**	0.457578	0.764383	Adriamycin	0.28322	0.800172
**4c**	0.480318	0.950596	AmphotericinB	0.215551	0.847453
**5d**	0.425604	0.864353	Apoptolidin	0.301694	0.830875
**6d**	0.582658	0.794739	Bleomycin	0.404781	0.826205
**7e**	0.198477	0.978871	BrefeldinA	0.255142	0.817388
**8e**	0.110591	0.946567	BrevetoxinB	0.149647	0.899486
**9f**	0.279873	0.860449	CalyculinA	0.442236	0.896401
**10f**	0.229213	0.87778	Colchicine	0.395296	0.837965
**11f**	0.65839	0.910791	Colchicine	0.258193	0.846521
**12f**	0.488957	0.808504	Colchicine	0.458247	0.773003
**12f`**	0.502805	0.714443	Colchicine	0.513293	0.755147
**13f**	0.323176	0.714363	CytochalasinB	0.431674	0.740608
**14f**	0.579194	0.668807	Discodermolide	0.182128	0.981632
**15f**	0.644331	0.905114	DuocarmycinA	0.104513	0.945885
**16f**	0.529740	0.838876	EpothiloneA	0.44771	0.804309
**17f**	0.574725	0.676289	ErythromycinA	0.485996	0.81381
**18f**	0.452583	0.828674	Fumagillin	0.066479	0.974218
**19f**	0.197015	0.944054	Geldanamycin	0.369201	0.725345
**20f**	0.476582	0.803727	Geldanamycin	0.392818	0.769442
**21f**	0.577074	0.839108	GinkgolideB	0.363537	0.879045
**22f**	0.473966	0.836454	Lactacystin	0.478299	0.938709
**23f**	0.521283	0.939915	Monensin	0.221267	0.91423
			MycobactinS	0.56741	0.787169
			PenicillinG	0.227679	0.84074
			PhorbolMA	0.403734	0.770927
			PhorbolMA	0.512005	0.787906

Natural products			Natural products		
Compound	npr1	npr2	Compound	npr1	npr2
RifamycinB	0.53483	0.679088	SQ26180	0.435835	0.794101
RifamycinB	0.544966	0.674059	Thienamycin	0.47668	0.854387
RifamycinB	0.618686	0.762072	AvermectinB 1a	0.278083	0.812448
RifamycinB	0.524795	0.868426	Calicheamicin	0.227585	0.92033
RifamycinB	0.648821	0.813721	CyclosporinA	0.418415	0.935663
RifamycinB	0.67808	0.844921	Daptomycin	0.762741	0.908347
RifamycinB	0.627544	0.817624	EchinocandinB	0.317238	0.945521
RifamycinB	0.60182	0.886052	FK506	0.377179	0.761235
SalicylihalamideA	0.191225	0.84892	Lipstatin	0.462998	0.73867
Staurosporine	0.464212	0.664055	MidecamycinA1	0.325184	0.904263
Streptomycin	0.335744	0.786998	PseudomonicAcidA	0.380836	0.748719
TalaromycinB	0.186426	0.935125	Rapamycin	0.445581	0.764618
Telomestatin	0.496427	0.509642	Taxol	0.437027	0.832107
TrapoxinB	0.460215	0.680871	Validamycin	0.451444	0.807012
Trichostatin	0.288807	0.802339	MycoleptodisicnA	0.35082	0.758279
Vancomycin	0.516891	0.626096	SteenkrotinB	0.634869	0.834254
Vincristine	0.482176	0.946784	(+)-MuironolideA	0.627984	0.847099
Quinine	0.303785	0.834042	(-)-Morphine	0.50907	0.762043
Spongistatin1	0.428667	0.817984	(-)-Lycoramine	0.551205	0.560635
ZaragozicAcidA	0.287707	0.92077	(-)-Rocaglamide	0.689462	0.890844
Arglabin	0.402854	0.721308	SchilancitrilactonesB	0.242841	0.882821
Artemisinin	0.541282	0.644874	DaphniglaucinC	0.797208	0.883075
Bestatin	0.29757	0.838998	DaphnicyclidinA	0.45685	0.651231
CephamycinC	0.488698	0.85665	(-)-DaphmanidinA	0.577477	0.720988
Coformycin	0.298204	0.821609	Anibamine	0.690921	0.808847
Compactin	0.430983	0.716564	Radicicol	0.490589	0.823386
Forskolin	0.520873	0.688349			
Mizoribine	0.239424	0.857007			
Plaunotol	0.460051	0.90039			
Spergualin	0.541169	0.94605			

FDA-approved drugs embedded with pyrimidine moieties[6]

Compound	npr1	npr2
Lamivudine	0.245638	0.883881
Raltegravir	0.357692	0.772534
Imatinib	0.670512	0.713467
Erlotinib	0.344318	0.888863
Lapatinib	0.245018	0.832311
Rosuvastatin	0.221782	0.838978
Ocinaplon	0.246698	0.771762
Zaleplon	0.285975	0.826159
Indiplon	0.275805	0.767635
Sildenafil	0.491575	0.734667
Avanafil	0.396445	0.784796
Ceritinib	0.359299	0.988453
Pyrimethamine	0.406723	0.697858
Trimethoprim	0.254044	0.862894
Zidovudine	0.264752	0.939218

Supplementary Methods

1. General Information of Synthetic Protocols

NMR spectra were obtained on an Agilent 400-MR DD2 Magnetic Resonance System (400 MHz, Agilent, USA), JEOL JNM-LA400 with LFG [400 MHz, Jeol, Japan], Varian/Oxford As-500 [500 MHz, Varian Assoc., Palo Alto, USA] or Bruker Avance III HD 800 MHz NMR Spectrometer [800 MHz, Bruker, Germany]. Chemical shifts values were recorded as parts per million (δ), referenced to tetramethylsilane (TMS) as the internal standard or to the residual solvent peak ($CDCl_3$, ^{1}H: 7.26, ^{13}C: 77.16, CD_3OD, ^{1}H: 3.31, ^{13}C: 49.00, DMSO-d_6, ^{1}H: 2.50, ^{13}C: 39.52). Multiplicities were indicated as follows: s (singlet), d (doublet), t (triplet), q (quartet); m (multiplet); dd (doublet of doublet); dt (doublet of triplet); td (triplet of doublets); br s (broad singlet) and so on. Coupling constants were reported in hertz (Hz). IR spectra were measured on a Thermo Scientific Nicolet™ 6700 FT-IR spectrometer. Low resolution mass spectra were obtained on a Finnigan Surveyor MSQ Plus LC/MS [Thermo], LCQ LC/MS [Thermo] or Shimadzu LCMS-2020 using the electrospray ionization (ESI) method. High resolution mass spectra were analyzed at the Mass Spectrometry Laboratory of National Instrumentation Center for Environmental Management (NICEM) in Seoul on a LCQ LC/MS [Thermo] using the electrospray ionization (ESI) method or by Ultra High Resolution ESI Q-TOF mass spectrometer (Bruker). All commercially available reagents were used without further purification unless noted otherwise. Commercially available reagents were obtained from Sigma-Aldrich, TCI, Acros, or Alfa Aesar. All solvents were obtained by passing them through activated alumina columns of solvent purification systems from Glass Contour. Analytical thin-layer chromatography (TLC) was performed using Merck Kiselgel 60 F254 plates, and the components were visualized by observation under UV light (254 and 365 nm) or by treating the plates with ninhydrin followed by thermal visualization. Flash column chromatography was performed on Merck Kieselgel 60 (230–400 mesh). Microwave reactions were performed using the CEM Discover Benchmate and microwave reaction conditions were as indicated in the Experimental Section. HPLC purification was performed on an Agilent 1260 Infinity system with an YMC-Pack silica column (SL12S05-2520WTX, 250 mm×20 mm, 5 μm).

2. **Synthesis and Characterization of Substrates**

Synthetic procedure for the preparation of Benzyl *tert*-butyl (3-((triisopropyl-silyl)oxy)propane-1,2-diyl)(*S*)-dicarbamate (SI-1).

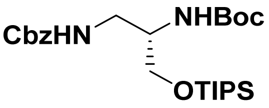

SI-1

Lithium borohydride ($LiBH_4$) (2.0 M solution in THF, 56.8 ml, 113.5 mmol) was slowly added to a stirring solution of methyl (*S*)-3-(((benzyloxy)carbonyl)amino)-2-((*tert*-butoxycarbonyl) amino)propanoate[3] (20.00 g, 56.76 mmol) in tetrahydrofuran (THF) (570.0 ml) at 0 °C. Then, the resulting mixture was left to stir and allowed to warm to r.t. After completion of the reaction as indicated by TLC, the reaction mixture was quenched with saturated NH_4Cl(aq) and extracted twice with dichloromethane (DCM). The combined organic layer was dried over anhydrous Na_2SO_4(s) and filtered. The organic layer was condensed under reduced pressure, and the crude resultant was dissolved in dry DCM (570.0 ml) under argon atmosphere. To this solution were sequentially added triethylamine (Et_3N) (15.83 ml, 113.5 mmol) and triisopropylsilyl trifluoromethanesulfonate (TIPS-OTf) (22.88 ml, 85.14 mmol) at 0 °C. The resulting mixture was left to stir and allowed to warm to r.t. After completion of the reaction as indicated by TLC, the resultant was quenched with saturated NH_4Cl(aq) and extracted twice with DCM. The combined organic layer was dried over anhydrous Na_2SO_4(s) and filtered. The filtrate was condensed under reduced pressure, followed by silica-gel flash column chromatography to afford the desired product **SI-1** (22.37 g, 82% yield) as a colorless oil (Supplementary Fig. 1).

R_f = 0.4 (hexane/EtOAc 5:1); ^{1}H NMR (400 MHz, $CDCl_3$) δ 7.35–7.29 (m, 5H), 5.32 (br s, 1H), 5.10 (s, 2H), 5.00 (br s, 1H), 3.77–3.73 (m, 3H), 3.47–3.39 (m, 2H), 1.43 (s, 9H), 1.06 (m, 21H); ^{13}C NMR (100 MHz, $CDCl_3$) δ 157.0, 156.1, 136.7, 128.6, 128.11, 128.07, 79.6, 66.8, 64.3, 51.8, 43.5, 28.4, 18.0, 11.9; LRMS(ESI+): Calcd for $C_{25}H_{45}N_2O_5Si^+$ $[M+H]^+$ 481.31, found 481.11.

Synthetic procedure for the preparation of *tert*-butyl (*S*)-(1-((6-chloro-5-formylpyrimidin-4-yl)amino)-3-((triisopropylsilyl)oxy)pro-pan-2-yl)carbamate (SI-2).

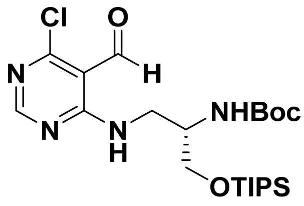

SI-2

To a solution of **SI-1** (22.37 g, 46.54 mmol) in methanol (MeOH) (470.0 ml) was carefully added 10 wt. % Pd/C (11.19 g) and the mixture was vigorously stirred under H_2 atmosphere (1 atm) at r.t. After completion of the reaction as indicated by TLC, the reaction mixture was filtered through Celite® while washing with ethyl acetate. The filtrate was condensed under reduced pressure and the crude resultant was dissolved in chloroform ($CHCl_3$, 470.0 ml). To this solution were sequentially added Et_3N (9.737 ml, 69.81 mmol) and 4,6-dichloropyrimidine-5-carboxaldehyde (8.237 g, 46.54 mmol) at 0 °C and left to stir. After 30 min, the reaction mixture was quenched with saturated NH_4Cl(aq). The resultant was extracted twice with $CHCl_3$, dried over anhydrous Na_2SO_4(s), and filtered. The filtrate was condensed under reduced pressure and purified by silica-gel flash column chromatography to afford the desired product **SI-2** (15.19 g, 67% yield) as a colorless oil (Supplementary Fig. 1).

R_f = 0.5 (hexane/EtOAc 3:1); ^{1}H NMR (400 MHz, $CDCl_3$) δ 10.28 (s, 1H), 9.30 (t, J = 5.7 Hz, 1H), 8.34 (s, 1H), 4.99 (d, J = 8.6 Hz, 1H), 3.90–3.62 (m, 5H), 1.33 (s, 9H), 1.06 (m, 21H); ^{13}C NMR (100 MHz, $CDCl_3$) δ 191.1, 165.3, 161.7, 160.7, 155.6, 108.2, 79.5, 63.7, 51.4, 42.4, 28.3, 17.9, 11.9; LRMS (ESI+): Calcd for $C_{22}H_{40}ClN_4O_4Si^+$ $[M+H]^+$ 487.25, found 487.03.

General procedure for the preparation of 1a–c and 1e–f.

To a stirring solution of **SI-2** (500 mg, 1.026 mmol) and K_2CO_3 (283.6 mg, 2.052 mmol) in *N*,*N*-dimethylformamide (DMF) (10.0 ml) was added amine[4,5] (1.539 mmol) and the resulting mixture was stirred at 40 °C. After completion of the reaction as indicated by TLC, the resultant

was quenched with saturated NH_4Cl(aq) and extracted twice with ethyl acetate. The combined organic layer was dried over anhydrous Na_2SO_4(s) and filtered. The organic solvent was evaporated under vacuum to afford a crude oil, which was purified by silica-gel flash column chromatography to afford **SI-3**. To obtain the cyclic imine product **1**, **SI-3** was first treated with 10% trifluoroacetic acid (TFA) in DCM (20.0 ml) at r.t. After the starting material was consumed as indicated by TLC, any excess TFA was removed by azeotropic evaporation with toluene under reduced pressure, and the crude resultant was dissolved in 1% acetic acid (AcOH) in $CHCl_3$ (100.0 ml). The resulting mixture was stirred at 40 °C. After completion of the reaction as indicated by TLC, the resultant was quenched with saturated $NaHCO_3$(aq) and extracted twice with $CHCl_3$. The combined organic layer was dried over anhydrous Na_2SO_4(s) and filtered. The filtrate was condensed under reduced pressure, followed by silica-gel flash column chromatography to afford the desired product **1** (Supplementary Fig. 2).

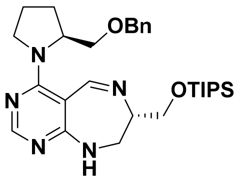

1a

(*S*)-4-((*S*)-2-((benzyloxy)methyl)pyrrolidin-1-yl)-7-(((triisopropylsilyl)oxy)methyl)-8,9-dihydro-7H-pyrimido[4,5-e][1,4]diazepine (1a). A pale yellow oil; R_f = 0.2 (DCM/MeOH = 20:1); 403.1 mg, 75% overall yield; ^{1}H NMR (400 MHz, $CDCl_3$) δ 8.11 (s,1H), 7.98 (s, 1H), 7.29 (m, 5H), 5.97 (br s, 1H), 4.74 (m, 1H), 4.54, 4.53 (ABq, J_{AB} = 12.1 Hz, 2H), 4.18 (dd, *J* = 9.8, 4.7 Hz, 1H), 3.90 (m, 1H), 3.80 (dd, *J* = 12.5, 5.5 Hz, 1H), 3.73 (m, 2H), 3.67 (m, 1H), 3.60 (dd, *J* = 9.4, 5.9 Hz, 1H), 3.47 (m, 1H), 3.34 (dd, *J* = 11.3, 7.0 Hz, 1H), 2.15 (m, 1H), 1.96 (m, 2H), 1.71 (m, 1H), 1.08 (m, 21H); ^{13}C NMR (100 MHz, $CDCl_3$) δ 164.5, 160.9, 159.1, 157.4, 138.6, 128.4, 127.6, 127.5, 93.1, 73.3, 71.1, 65.1, 64.2, 58.0, 55.2, 47.8, 28.0, 26.1, 18.2, 12.0; HRMS(ESI+): Calcd for $C_{29}H_{46}N_5O_2Si^+$ $[M+H]^+$ 524.3415, found 524.3414, Δppm –0.19.

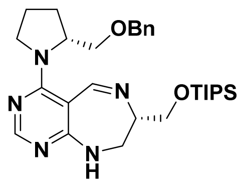

1b

(*S*)-4-((*R*)-2-((benzyloxy)methyl)pyrrolidin-1-yl)-7-(((triisopropylsilyl)oxy)methyl)-8,9-dihydro-7H-pyrimido[4,5-e][1,4]diazepine (1b). A pale yellow oil; $R_f = 0.2$ (DCM/MeOH = 20:1); 408.4 mg, 76% overall yield; ^{1}H NMR (400 MHz, $CDCl_3$) δ 8.14 (s, 1H), 7.96 (s, 1H), 7.30 (m, 5H), 6.20 (br s, 1H), 4.74 (m, 1H), 4.53 (s, 2H), 4.19 (dd, J = 9.7, 3.1 Hz, 1H), 4.02 (m, 1H), 3.84 (m, 1H), 3.74 (m, 2H), 3.63 (m, 2H), 3.46 (t, J = 9.7 Hz, 1H), 3.14 (m, 1H), 2.14 (m, 1H), 1.99 (m, 2H), 1.74 (m, 1H), 1.09 (m, 21H); ^{13}C NMR (100 MHz, $CDCl_3$) δ 165.0, 164.2, 156.4, 154.0, 138.4, 128.2, 127.4, 127.4, 92.0, 73.1, 70.8, 65.3, 65.0, 57.9, 55.2, 46.6, 27.8, 25.8, 18.0, 11.8; HRMS(ESI+): Calcd for $C_{29}H_{46}N_5O_2Si^+$ $[M+H]^+$ 524.3415, found 524.3415.

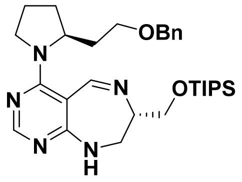

1c

(*S*)-4-((*S*)-2-(2-(benzyloxy)ethyl)pyrrolidin-1-yl)-7-(((triisopropylsilyl)oxy)methyl)-8,9-dihydro-7H-pyrimido[4,5-e][1,4]diazepine (1c). A colorless oil; $R_f = 0.35$ (DCM/MeOH = 20:1); 419.4 mg, 76% overall yield; ^{1}H NMR (400 MHz, $CDCl_3$) δ 8.07 (s, 1H), 7.99 (s, 1H), 7.33 (m, 4H), 7.29–7.26 (m, 1H), 6.17 (br s, 1H), 4.61 (m, 1H), 4.53, 4.26 (ABq, J_{AB} = 11.94 Hz, 2H), 4.18 (dd, J = 9.8, 4.3 Hz, 1H), 3.92 (m, 1H), 3.81–3.62 (m, 3H), 3.60–3.56 (m, 2H), 3.45 (m, 1H), 3.33 (m, 1H), 2.33–2.25 (m, 1H), 2.19–2.13 (m, 1H), 1.91–1.87 (m, 1H), 1.80–1.66 (m, 3H), 1.08 (m, 21H); ^{13}C NMR (100 MHz, $CDCl_3$) δ 164.8, 161.1, 159.0, 157.4, 138.6, 128.4, 127.8, 127.6, 93.2, 73.0, 67.7, 65.1, 64.3, 56.9, 55.1, 47.6, 34.1, 31.0, 26.2, 18.1, 12.0; HRMS(ESI+): Calcd for $C_{30}H_{48}N_5O_2Si^+$ $[M+H]^+$ 538.3572, found 538.3571, Δppm –0.19.

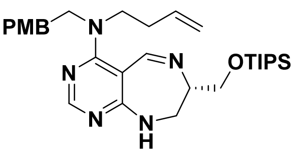

1e

(*S*)-N-(but-3-en-1-yl)-N-(4-methoxyphenethyl)-7-(((triisopropylsilyl)oxy)methyl)-8,9-dihydro-7H-pyrimido[4,5-e][1,4]diazepin-4-amine (1e). A pale yellow oil; R_f = 0.4 (DCM/MeOH = 20:1); 364.2 mg, 66% overall yield; ^{1}H NMR (400 MHz, $CDCl_3$) δ 8.05 (s, 1H), 7.98 (d, *J* = 1.9 Hz, 1H), 7.07 (d, *J* = 8.6 Hz, 2H), 6.80 (d, *J* = 8.6 Hz, 2H), 6.42 (m, 1H), 5.72 (dddd, *J* = 16.8, 10.1, 6.6, 6.6 Hz, 1H), 5.02 (m, 2H), 4.18 (dd, *J* = 9.7, 4.3 Hz, 1H), 3.84 (m, 3H), 3.77 (s, 3H), 3.69 (m, 2H), 3.46 (m, 1H), 3.34 (m, 2H), 2.83 (t, *J* = 7.4 Hz, 2H), 2.33 (q, *J* = 7.0 Hz, 2H), 1.08 (m, 21H); ^{13}C NMR (100 MHz, $CDCl_3$) δ 167.2, 161.9, 158.2, 157.8, 157.1, 135.2, 131.0, 129.7, 117.0, 114.0, 94.3, 65.2, 64.5, 55.3, 53.5, 51.2, 47.6, 33.7, 32.6, 18.1, 12.0; HRMS(ESI+): Calcd for $C_{30}H_{48}N_5O_2Si^+$ $[M+H]^+$ 538.3572, found 538.3572.

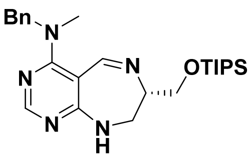

1f

(S)-N-benzyl-N-methyl-7-(((triisopropylsilyl)oxy)methyl)-8,9-dihydro-7H-pyrimido[4,5-e][1,4]diazepin-4-amine (1f). A colorless oil; R_f = 0.35 (DCM/MeOH = 20:1); 349.1 mg, 75% overall yield; ^{1}H NMR (400 MHz, $CDCl_3$) δ 8.17 (d, *J* = 1.9 Hz, 1H), 8.06 (s, 1H), 7.35–7.25 (m, 5H), 6.99 (br s, 1H), 4.81, 4.71 (ABq, J_{AB} = 15.2 Hz, 2H), 4.18 (dd, *J* = 9.8, 4.3 Hz, 1H), 3.89 (m, 2H), 3.66 (t, *J* = 10.0 Hz, 1H), 3.32 (ddd, *J* = 13.0, 7.1, 2.5 Hz, 1H), 3.03 (s, 3H), 1.08 (m, 21H); ^{13}C NMR (100 MHz, $CDCl_3$) δ 167.4, 162.4, 157.2, 157.1, 137.3, 128.7, 127.7, 127.4, 92.7, 65.1, 64.5, 56.6, 47.3, 40.1, 18.0, 11.9; HRMS(ESI+): Calcd for $C_{25}H_{40}N_5OSi^+$ $[M+H]^+$ 454.2997, found 454.2996, Δppm –0.22.

(*S*)-4-(prop-1-en-2-yl)-7-(((triisopropylsilyl)oxy)methyl)-8,9-dihydro-7H-pyrimido[4,5-e][1,4]diazepine (1d).

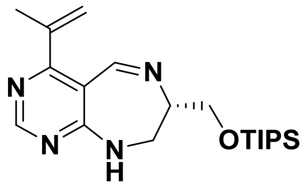

1d

Palladium tetrakis(triphenylphosphine) $Pd(PPh_3)_4$ (119.0 mg, 0.103 mmol, 10 mol%) was added to a vigorously stirring solution of **SI-2** (500 mg, 1.026 mmol), isopropenylboronic acid pinacol ester (0.290 ml, 1.539 mmol), and Na_2CO_3 (380.6 mg, 3.591 mmol.) in toluene–water solvent mixture (25.0 ml/25.0 ml) at r.t. The resulting mixture was stirred at 80 °C. After completion of the reaction as indicated by TLC, the reaction mixture was diluted with ethyl acetate, washed with brine, dried over anhydrous Na_2SO_4(s), and filtered. The filtrate was condensed under reduced pressure and the residue was purified with silica-gel flash column chromatography to provide compound **SI-4** (379.2 mg, 75% yield). To obtain the cyclic imine products **1d**, **SI-4** (379.2 mg, 0.770 mmol) was first treated with 10% TFA in DCM (100.0 ml). After the starting material was consumed as indicated by TLC, the resultant was quenched with saturated $NaHCO_3$(aq) and extracted twice with DCM. The combined organic layer was dried over anhydrous Na_2SO_4(s) and filtered. The filtrate was condensed under reduced pressure and the crude resultant was dissolved in DCM (100.0 ml). To this solution was added Na_2SO_4(s) (2.915 g, 20.52 mmol) and the resulting mixture was stirred at 40 °C. After completion of the reaction as indicated by TLC, the reaction mixture was filtered and concentrated *in vacuo*. The residue was purified with silica-gel flash column chromatography to afford the desired cyclic imine product **1d** (234.4 mg, 81% yield, 61% overall yield) as a colorless oil (Supplementary Fig. 3).

R_f = 0.2 (DCM/MeOH 20:1); ^{1}H NMR (400 MHz, $CDCl_3$) δ 8.47 (d, *J* = 2.3 Hz, 1H), 8.43 (s, 1H), 7.37 (br d, *J* = 5.0 Hz, 1H), 5.54 (s, 1H), 5.10 (s, 1H), 4.27 (dd, *J* = 9.7, 4.7 Hz, 1H), 3.95 (dd, *J* = 13.0, 7.0 Hz, 1H), 3.81 (m, 1H), 3.69 (t, *J* = 10.1 Hz, 1H), 3.19 (m, 1H), 2.17 (s, 3H), 1.08 (m, 21H); ^{13}C NMR (100 MHz, $CDCl_3$) δ 172.2, 161.0, 158.0, 157.9, 142.2, 120.9, 107.3, 65.2, 64.8, 47.4, 22.3, 18.1, 11.9; HRMS(ESI+): Calcd for $C_{20}H_{35}N_4OSi^+$ $[M+H]^+$ 375.2575, found 375.2574, Δppm –0.27.

Synthetic procedure for the preparation of 2a

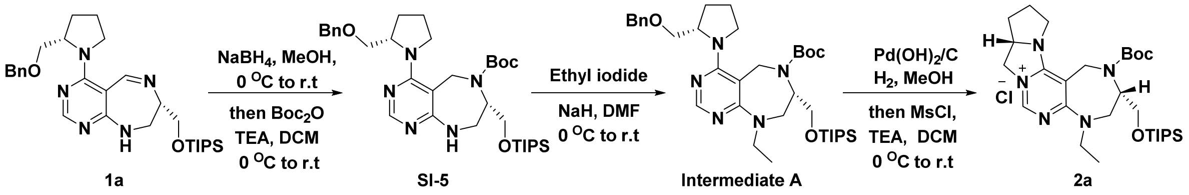

Compound 2a. To a stirring solution of **1a** (403.1 mg, 0.770 mmol) in MeOH (8.0 ml) was added sodium borohydride ($NaBH_4$) (145.6 mg, 3.850 mmol) at 0 °C. The resulting mixture was left to stir and allowed to warm to r.t. After completion of the reaction as indicated by TLC, the resultant was quenched with saturated $NaHCO_3$(aq) and extracted twice with DCM. The combined organic layer was dried over anhydrous Na_2SO_4(s) and filtered. The organic solvent was evaporated under reduced pressure, and the crude resultant was dissolved in DCM (8.0 ml). To this solution were sequentially added Et_3N (0.215 ml, 1.540 mmol) and di-*tert*-butyl dicarbonate (Boc_2O) (218.5 mg, 1.001 mmol) at 0 °C. The resulting mixture was left to stir and allowed to warm to r.t. After completion of the reaction as indicated by TLC, the resultant was quenched with saturated NH_4Cl(aq) and extracted twice with DCM. The combined organic layer was dried over anhydrous Na_2SO_4(s) and the filtrate was condensed under reduced pressure, followed by silica-gel flash column chromatography to afford **SI-5** (260.3 mg, 54% yield). To a solution of **SI-5** (260.3 mg, 0.416 mmol) in DMF (4.0 ml) under argon atmosphere was added NaH (60% dispersion in mineral oil, 33.28 mg, 0.832 mmol) at 0 °C and left to stir. After 30 min, ethyl iodide (0. 067 ml, 0.832 mmol) was slowly added and the mixture was allowed to slowly warm to r.t. After the starting material was consumed as indicated by TLC, the reaction mixture was quenched with saturated NH_4Cl(aq). The resultant was extracted twice with ethyl acetate, dried with anhydrous Na_2SO_4(s), filtered, and concentrated *in vacuo*. The residue was purified by silica-gel flash column chromatography to obtain intermediate **A** (258.5 mg, 95% yield). To a solution of intermediate **A** (258.3 mg, 0.395 mmol) in MeOH (4.0 ml) was added 20 wt. % $Pd(OH)_2/C$ (25.9 mg) and then, the mixture was vigorously stirred under H_2 atmosphere (1 atm) at r.t. After completion of the reaction as indicated by TLC, the reaction mixture was filtered through Celite® while washing with ethyl acetate. The filtrate was condensed under reduced pressure and the crude resultant was dissolved in DCM (4.0 ml). To this solution were sequentially added Et_3N (0.165 ml, 1.185 mmol) and methanesulfonyl chloride (MsCl) (0.061 ml, 0.790 mmol) at 0 °C. The resulting mixture was allowed to slowly

warm up to r.t. After completion of the reaction indicated by TLC, the reaction mixture was diluted with DCM and washed twice with 1N HCl. The resultant was dried with anhydrous Na_2SO_4(s), filtered, condensed under reduced pressure, and purified by silica-gel flash column chromatography to afford the desired product **2a** (280.6 mg, 61 % yield, 31% overall yield) as a white solid;

R_f = 0.1 (DCM/MeOH = 10:1); ^{1}H NMR (400 MHz, DMSO-d_6, 100 °C) δ 8.50 (s, 1H), 4.64 (m, 2H), 4.48 (m, 2H), 4.33 (m, 2H), 4.13 (m, 1H), 3.82 (m, 2H), 3.76 (m, 1H), 3.69 (m, 1H), 3.62 (dd, *J* = 15.1, 4.6 Hz, 1H), 3.57 (m, 1H), 3.42 (m, 1H), 2.14 (m, 1H), 2.04 (m, 2H), 1.66 (m, 1H), 1.32 (br s, 9H), 1.17 (t, *J* = 7.1 Hz, 3H), 1.08 (m, 21H); ^{13}C NMR (100 MHz, DMSO-d_6, 100 °C) δ 162.0, 155.8, 154.0, 146.5, 79.3, 62.8, 62.4., 50.8, 50.2, 48.9, 45.1, 29.6, 27.4, 25.7, 17.2, 12.4, 11.0; IR (neat) ν_{max}: 2940, 2866, 1698, 1638, 1523, 1174 cm^{-1}; HRMS(ESI+): Calcd for $C_{29}H_{52}N_5O_3Si^+$ [M]$^+$ 546.3834, found 546.3831, Δppm –0.55; mp: 136–138 °C.

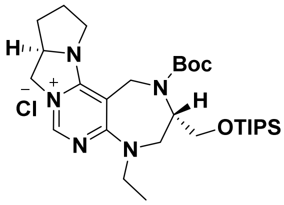

3b

Compound 3b. A pale yellow solid; R_f = 0.1 (DCM/MeOH = 10:1); 38% overall yield; ^{1}H NMR (400 MHz, DMSO-d_6, 100 °C) δ 8.42 (s, 1H), 4.88 (d, *J* = 16.9 Hz, 1H), 4.58 (d, *J* = 16.9 Hz, 1H), 4.54 (dd, *J* = 10.8, 9.4 Hz, 1H), 4.38 (m, 2H), 4.27 (dd, *J* = 10.9, 8.2 Hz, 1H), 4.16 (dd, *J* = 15.1, 11.9 Hz, 1H), 3.86 (m, 2H), 3.80 (m, 2H), 3.70 (m, 2H), 3.65 (m, 1H), 2.17 (m, 3H), 1.73 (m, 1H), 1.41 (s, 9H), 1.18 (t, *J* = 6.9 Hz, 3H), 1.09 (m, 21H); ^{13}C NMR (100 MHz, DMSO-d_6, 100 °C) δ 162.3, 153.8, 152.8, 146.5, 93.0, 79.8, 63.2, 62.5, 56.9, 50.6, 49.0, 48.0, 46.1, 36.7, 29.2, 27.5, 26.6, 17.2, 12.3, 10.9; IR (neat) ν_{max}: 2944, 2863, 1698, 1636, 1517, 1166 cm^{-1}; HRMS(ESI+): Calcd for $C_{29}H_{52}N_5O_3Si^+$ [M]$^+$ 546.3834, found 546.3833, Δppm –0.18; mp: 77–79 °C. The product was synthesized according to the synthetic procedure for the preparation of **2a** from **1b**.

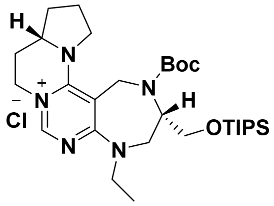

4c

Compound 4c. A pale yellow solid; R_f = 0.2 (DCM/MeOH = 10:1); 40 % overall yield; ^{1}H NMR (400 MHz, DMSO-d_6, 100 °C) δ 8.34 (s, 1H), 4.48 (m, 2H), 4.23 (m, 2H), 4.05 (m, 1H), 3.85 (m, 2H), 3.79 (m, 2H), 3.71 (m, 3H), 3.61 (dd, J = 15.0, 4.6 Hz, 1H), 3.53 (m, 1H), 3.06 (br s, 1H), 3.00 (m, 1H), 2.18 (m, 1H), 1.91 (m, 3H), 1.29 (br s, 9H), 1.18 (t, J = 7.0 Hz, 3H), 1.09 (m, 21H); ^{13}C NMR (100 MHz, DMSO-d_6, 100 °C) δ 161.5, 153.1, 149.2, 149.2, 71.7, 63.9, 56.3, 55.8, 49.7, 49.0, 46.8, 30.5, 28.4, 25.0, 23.6, 18.2, 13.7, 12.0; IR (neat) ν_{max}: 2946, 2867, 1694, 1625, 1529, 1457, 1116 cm^{-1}; HRMS(ESI+): Calcd for $C_{30}H_{54}N_5O_3Si^+$ [M]$^+$ 560.3990, found 560.3993, Δppm –0.54.; mp: 71–73 °C. The product was synthesized according to the synthetic procedure for the preparation of **2a** from **1c**.

Synthetic procedure for the preparation of 5d

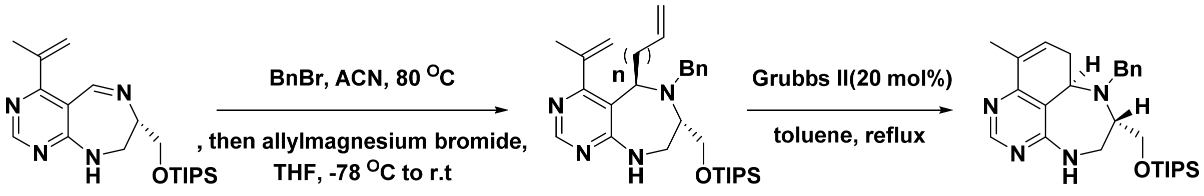

Intermediate B (n=1). To a stirring solution of **1d** (100 mg, 0.267 mmol) in acetonitrile (ACN) (13.5 ml), benzyl bromide (0.048 ml, 0.401 mmol) was added and the resulting mixture was stirred at 80 °C. After completion of the reaction indicated by TLC, the organic solvent was removed under reduced pressure and the crude resultant was washed with hexane to remove any excess benzyl bromide. To a cooled solution of resultant iminium ion in dry THF (27.0 ml) was added allylmagnesium bromide (1.0 M solution in diethyl ether, 1.335 ml, 1.335 mmol) dropwise over 30 min at –78 °C. The reaction mixture was allowed to slowly warm to r.t over an 18 h period. The resultant was quenched with saturated NH_4Cl(aq) and extracted twice with

DCM. The combined organic layer was dried over anhydrous Na_2SO_4(s) and filtered. The filtrate was condensed under reduced pressure, followed by flash column chromatography to afford intermediate **B** (n= 1, 115.0 mg, 85% yield, d.r. >99:1) as a pale yellow oil.

R_f = 0.5 (hexane/EtOAc = 1:1); ^{1}H NMR (400 MHz, $CDCl_3$) δ 8.40 (s, 1H), 7.32–7.29 (m, 3H), 7.24–7.20 (m, 2H), 5.88–5.78 (m, 2H), 5.03 (m, 2H), 4.94 (s, 1H), 4.69 (s, 1H), 4.29 (dd, *J* = 9.4, 5.9 Hz, 1H), 3.86 (d, *J* = 15.3 Hz, 1H), 3.78 (dd, *J* = 9.6, 3.7 Hz, 1H), 3.64–3.57 (m, 3H), 3.50–3.40 (m, 2H), 2.84 (m, 1H), 2.22 (m, 1H), 1.81 (s, 3H), 1.01 (s, 21H); ^{13}C NMR (100 MHz, $CDCl_3$) δ 168.4, 164.6, 155.6, 143.0, 140.1, 136.0, 128.4, 127.7, 126.9, 116.7, 116.3, 116.0, 64.4, 59.3, 57.9, 53.0, 43.3, 37.1, 23.0, 18.1, 11.9; HRMS(ESI+): Calcd for $C_{30}H_{47}N_4OSi^+$ [M+H]$^+$ 507.3514, found 507.3516, Δppm +0.39. The diastereomeric ratio was determined by LC-MS analysis of crude reaction mixture and by 1H NMR analysis of samples purified by short silica-gel column (Supplementary Fig. 7 and 34).

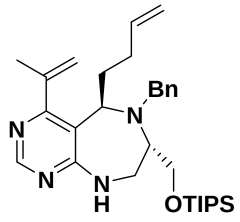

Intermediate B (n= 2)

Intermediate B (n=2). A yellow oil; R_f = 0.5 (hexane/EtOAc = 1:1); 120.8 mg, 87% yield; d.r. >99:1; ^{1}H NMR (400 MHz, $CDCl_3$) δ 8.40 (s, 1H), 7.33–7.21 (m, 5H), 5.78 (m, 1H), 5.55 (d, *J* = 5.1 Hz, 1H), 5.01 (dd, *J* = 17.2, 1.2 Hz, 1H), 4.94 (m, 2H), 4.72 (s, 1H), 4.21 (dd, *J* = 10.2, 5.1 Hz, 1H), 3.84 (m, 2H), 3.64–3.54 (m, 3H), 3.48–3.37 (m, 2H), 2.33–2.17 (m, 2H), 2.11–2.04 (m, 1H), 1.83 (s, 3H), 1.45–1.36 (m, 1H), 1.06 (m, 21H); ^{13}C NMR (100 MHz, $CDCl_3$) δ 168.5, 164.7, 155.6, 143.1, 140.2, 138.2, 128.4, 127.8, 127.0, 117.7, 116.0, 114.9, 64.7, 59.2, 57.5, 52.6, 43.4, 31.7, 30.9, 23.1, 18.1, 12.0; HRMS(ESI+): Calcd for $C_{31}H_{49}N_4OSi^+$ [M+H]$^+$ 521.3670, found 521.3670. The product was synthesized according to the synthetic procedure for the preparation of **Intermediate B (n=1)** from **1d** and 3-butenylmagnesium bromide (0.2 M solution in THF). The diastereomeric ratio was determined by LC-MS analysis of crude reaction mixture and by ^{1}H NMR analysis of samples purified by short silica-gel column (Supplementary Fig. 8 and 35).

Compound 5d. To a solution of intermediate **B** (115.0 mg, 0.227 mmol) in toluene (11.5 ml) was added 2nd generation Grubbs' catalyst (38.20 mg, 0.045 mmol, 20 mol%) and the mixture was left to stir at reflux. After the starting material was indicated as by TLC, the organic solvent was removed under reduced pressure, and the residue was purified by silica-gel flash column chromatography to obtain the desired product **5d** (79.34 mg, 73 % yield, 62% overall yield) as a pale yellow oil.

R_f = 0.4 (hexane/EtOAc = 2:1); ^{1}H NMR (400 MHz, $CDCl_3$) δ 8.43 (s, 1H), 7.34–7.18 (m, 5H), 6.08 (br s, 1H), 5.87 (d, 6.3 Hz, 1H), 5.08 (t, J = 11.0 Hz, 1H), 4.16, 3.99 (ABq, J_{AB} = 14.9 Hz, 2H), 3.87 (t, J = 13.3 Hz, 1H), 3.54–3.41 (m, 3H), 3.17 (m, 1H), 2.54 (d, J = 10.5 Hz, 2H), 2.03 (s, 3H), 0.97 (m, 21 H); ^{13}C NMR (100 MHz, $CDCl_3$) δ 164.2, 160.4, 156.4, 141.1, 132.6, 130.4, 128.3, 128.1, 126.7, 110.9, 66.1, 61.8, 54.6, 50.5, 46.7, 29.5, 18.3, 17.9, 11.8; IR (neat) ν_{max}: 3241, 3028, 2940, 2865, 1565, 1460, 1116, 1066 cm^{-1}; HRMS(ESI+): Calcd for $C_{28}H_{43}N_4OSi^+$ $[M+H]^+$ 479.3201, found 479.3206, Δppm +1.04. The stereochemistry of this product was confirmed by Nuclear Overhauser Effect (NOE) spectroscopy. 1D-NOE result supported the stereochemistry as shown in Supplementary Fig. 37.

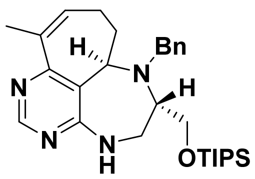

6d

Compound 6d. A yellow oil; R_f = 0.4 (hexane/EtOAc = 2:1); 87.95 mg, 72% yield, 63% overall yield; ^{1}H NMR (400 MHz, $CDCl_3$) δ 8.42 (s, 1H), 7.22–7.12 (m, 5H), 6.10 (t, J = 6.7 Hz, 1H), 5.82 (d, J = 5.5 Hz, 1H), 3.84–3.76 (m, 5H), 3.65 (dd, J = 11.7, 7.0 Hz, 1H), 3.49 (m, 1H), 2.89 (m, 1H), 2.48 (m, 1H), 2.10 (m, 2H), 1.91 (s, 3H), 1.84 (m, 1H), 1.26 (m, 21H); ^{13}C NMR (100 MHz, $CDCl_3$) δ 164.5, 164.1, 154.9, 140.0, 137.4, 131.8, 128.1, 127.4, 126.7, 117.1, 62.3, 61.2, 57.6, 56.3, 43.0, 35.4, 23.6, 20.1, 17.9, 11.8; IR (neat) ν_{max}: 3241, 2944, 2865, 1570, 1462, 1102, 883 cm^{-1}; HRMS(ESI+): Calcd for $C_{29}H_{45}N_4OSi^+$ $[M+H]^+$ 493.3357, found 493.3349, Δppm +0.41. The product was synthesized according to the synthetic procedure for the preparation of **5d** from **Intermediate B (n=2)**. The stereochemistry of this product was confirmed by NOE spectroscopy. 1D-NOE result supported the stereochemistry as shown in Supplementary Fig. 39.

Synthetic procedure for the preparation of 7e

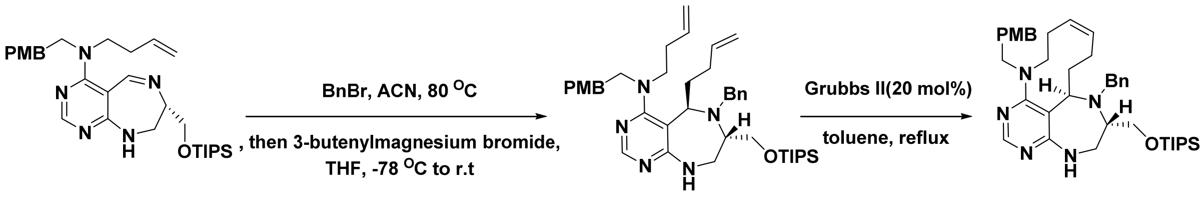

1e Intermediate C 7e

Compound 7e. To a stirring solution of **1e** (120 mg, 0.223 mmol) in acetonitrile (ACN) (11.0 ml), benzyl bromide (0.040 ml, 0.335 mmol) was added and the resulting mixture was stirred at 80 °C. After completion of the reaction indicated by TLC, the organic solvent was removed under reduced pressure and the crude resultant was washed with hexane to remove any excess benzyl bromide. To a cooled solution of resultant iminium ion in dry THF (22.0 ml) was added 3-butenylmagnesium bromide (0.2 M solution in THF, 5.575 ml, 1.115 mmol) dropwise over 30 min at –78 °C. The reaction mixture was allowed to slowly warm to r.t over an 18 h period. The resultant was quenched with saturated NH_4Cl(aq) and extracted twice with DCM. The combined organic layer was dried over anhydrous Na_2SO_4(s) and filtered. The filtrate was condensed under reduced pressure, followed by flash column chromatography to afford intermediate **C** (122.0 mg, 80% yield, d.r. 95:5). The diastereomeric ratio was determined by LC-MS analysis of crude reaction mixture (Supplementary Fig. 9). To a solution of intermediate **C** (122.0 mg, 0.178 mmol) in toluene (36.0 ml) was added 2nd generation Grubbs' catalyst (30.22 mg, 0.036 mmol, 20 mol%) and the mixture was left to stir at reflux. After the starting material was indicated as by TLC, the organic solvent was removed under reduced pressure, and the residue was purified by silica-gel flash column chromatography to obtain the desired product **7e** (85.24 mg, 73 % yield, 58% overall yield) as a pale yellow oil.

R_f = 0.2 (hexane/EtOAc = 3:1); ^{1}H NMR (400 MHz, $CDCl_3$) δ 8.15 (s, 1H), 7.15–7.05 (m, 5H), 6.99 (d, J = 8.6 Hz, 2H), 6.80 (d, J = 8.2 Hz, 2H), 5.47 (m, 1H), 5.37 (m, 2H), 4.30 (dd, J = 11.9, 2.2 Hz, 1H), 4.13 (m, 1H), 3.80 (s, 3H), 3.75 (m, 2H), 3.72 (m, 1H), 3.39 (m, 4H), 3.11 (m, 1H), 2.80 (m, 1H), 2.59 (m, 3H), 2.33 (m, 1H), 2.01 (m, 1H), 1.88 (m, 1H), 1.77 (m, 2H), 1.50 (m, 1H), 0.92 (m, 21H); ^{13}C NMR (100 MHz, $CDCl_3$) δ 167.4, 165.0, 158.0, 155.4, 140.2, 132.1, 131.6, 129.8, 128.8, 128.3, 128.2, 126.8, 113.9, 106.1, 64.5, 60.9, 59.2, 59.1, 57.6, 55.3, 46.9, 43.8, 29.2, 26.3, 23.8, 18.0, 11.9; IR (neat) ν_{max}: 3245, 3000, 2942, 2865, 1572, 1512, 1247, 1113 cm^{-1}; HRMS(ESI+): Calcd for $C_{39}H_{58}N_5O_2Si^+$ [M+H]$^+$ 656.4354, found 656.4345,

Δppm –1.37. The stereochemistry of **7e** was confirmed by NOE spectroscopy. 1D-NOE result supported the stereochemistry as shown in Supplementary Fig. 41.

Synthetic procedure for the preparation of 8e

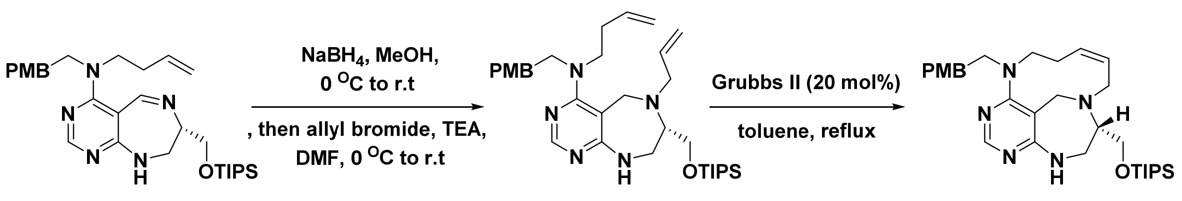

Compound 8e. To a stirring solution of **1e** (120 mg, 0.223 mmol) in MeOH (2.2 ml) was added $NaBH_4$ (42.18 mg, 1.115 mmol) at 0 °C and the resulting mixture was warmed to r.t. After the starting material was consumed as indicated by TLC, the resultant was quenched with saturated $NaHCO_3$(aq) and extracted twice with DCM. The combined organic layer was dried over anhydrous Na_2SO_4(s) and filtered. The solvent was evaporated under vacuum, and then the crude resultant was dissolved in DMF (2.2 ml). To this solution were sequentially added Et_3N (0.062 ml, 0.446 mmol) and allyl bromide (0.025 ml, 0.290 mmol) at 0 °C. The reaction was allowed to slowly warm to r.t. After completion of the reaction indicated by TLC, the resultant was quenched with saturated NH_4Cl(aq) and extracted twice with ethyl acetate. The combined organic layer was dried over anhydrous Na_2SO_4(s) and filtered. The filtrate was condensed under reduced pressure, followed by silica-gel flash column chromatography to afford **SI-6** (81.47 mg, 63% yield). To a solution of **SI-6** (81.47 mg, 0.140 mmol) in toluene (28.0 ml) was added second generation Grubbs' catalyst (23.77 mg, 0.028 mmol, 20 mol%) and the mixture was left to stir at reflux. After completion of the reaction as indicated by TLC, solvent was removed under reduced pressure and the residue was purified by silica-gel flash column chromatography to obtain the desired product **8e** (54.08 mg, 70% yield, 44% overall yield) as a yellow oil.

R_f = 0.3 (DCM/MeOH = 20:1); ^{1}H NMR (400 MHz, $CDCl_3$) δ 8.35 (s, 1H), 7.02 (d, J = 8.6 Hz, 2H), 6.79 (d, J = 8.2 Hz, 2H), 5.74 (br d, J = 5.1 Hz, 1H), 5.67 (td, J = 11.2, 4.5 Hz, 1H), 5.51 (td, J = 10.9, 4.5 Hz, 1H), 4.58 (d, J = 16.0 Hz, 1H), 4.06 (ddd, J = 14.6, 10.9, 3.1 Hz,

1H), 3.97 (d, J = 16.0 Hz, 1H), 3.76 (s, 3H), 3.59 (dd, J =10.0, 4.1 Hz, 1H), 3.46 (m, 2H), 3.37 (m, 2H), 3.08 (m, 2H), 2.92 (m, 2H), 2.73 (m, 1H), 2.54 (m, 1H), 2.39 (ddd, J = 13.4, 10.7, 5.7, 1H), 2.18 (m, 1H), 1.76 (br d, J = 12.1 Hz, 1H), 1.07 (m, 21H); ^{13}C NMR (100 MHz, $CDCl_3$) δ 168.2, 165.8, 157.9, 157.7, 134.3, 132.0, 129.5, 127.4, 113.7, 113.3, 69.4, 64.7, 58.1, 55.3, 52.0, 50.5, 43.9, 40.5, 33.8, 25.9, 18.0, 11.9; IR (neat) ν_{max}: 3239, 3006, 2945, 2866, 1574, 1512, 1265 cm^{-1}; HRMS(ESI+): Calcd for $C_{31}H_{50}N_5O_2Si^+$ $[M+H]^+$ 552.3728, found 552.3722, Δppm –1.09.

General procedure for the preparation of 9f and 10f

To a microwave vessel tightly sealed with a cap, were added **1f** (90.00 mg, 0.198 mmol), alkyne (0.5 mmol), 4-picoline *N*-oxide (54.57mg, 0.5 mmol) and $Rh(PPh_3)_3Cl$ (18.50 mg, 0.020 mmol, 10 mol%) in ACN (3.0 ml). The resulting mixture was heated under microwave irradiation (100 watt) at 90 °C for 35 min. The reaction mixture was diluted with DCM, washed with brine, dried over anhydrous Na_2SO_4(s), filtered, and concentrated *in vacuo*. The residue was purified by silica-gel flash column chromatography to obtain the desired β-lactam product.

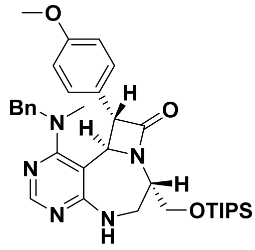

9f

Compound 9f. A pale yellow oil; R_f = 0.3 (DCM/MeOH = 20:1); 72.69 mg, 61% yield; d.r. >99:1; ^{1}H NMR (400 MHz, $CDCl_3$) δ 8.10 (s, 1H), 7.25 (m, 3H), 7.11 (d, J = 8.2 Hz, 4H), 6.83 (d, J = 8.2 Hz, 2H), 6.30 (dd, J = 6.5, 4.5 Hz, 1H), 5.06 (s, 1H), 4.43, 4.31 (ABq, J_{AB} = 14.9 Hz, 2H), 4.21 (dd, J =10.2, 3.5 Hz, 1H), 4.12 (m, 1H), 3.87 (m, 2H). 3.81 (s, 3H), 3.56 (s, 1H), 3.43 (ddd, J = 14.2, 7.3, 4.3 Hz, 1H), 2.44 (s, 3H), 1.09 (m, 21H); ^{13}C NMR (100 MHz, $CDCl_3$) δ 168.0, 165.3, 163.0, 159.2, 155.0, 137.5, 129.0, 128.5, 128.4, 127.3, 127.1, 114.1, 96.9, 65.2,

63.5, 59.5, 55.3, 55.2, 54.2, 42.9, 38.1, 18.0, 11.8; IR (neat) ν_{max}: 3250, 3056, 2942, 2865, 1751, 1567, 1119 cm^{-1}; HRMS(ESI+): Calcd for $C_{34}H_{48}N_5O_3Si^+$ $[M+H]^+$ 602.3521, found 602.3528, Δppm +1.16. The diastereomeric ratio was determined by LC-MS analysis of crude reaction mixture (Supplementary Fig. 10). The stereochemistry of this product was confirmed by NOE spectroscopy. 1D-NOE result supported the stereochemistry as shown in Supplementary Fig. 44.

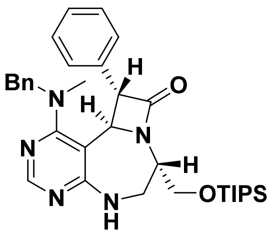

10f

Compound 10f. A yellow solid; R_f = 0.3 (DCM/MeOH = 20:1); 54.35 mg, 48% yield; d.r. 90:10; ^{1}H NMR (400 MHz, $CDCl_3$) δ 8.12 (s, 1H), 7.70–7.65 (m, 1H), 7.53 (m, 1H), 7.46 (m, 1H), 7.30 (m, 2H), 7.23 (m, 3H), 7.09 (m, 2H), 6.35 (dd, *J* = 6.7, 4.7 Hz, 1H), 5.11 (d, *J* = 1.2 Hz, 1H), 4.39 (m, 1H), 4.23 (m, 2H), 4.13 (m, 1H), 3.87 (m, 2H), 3.60 (d, *J* = 1.5 Hz, 1H), 3.43 (ddd, *J* = 14.4, 7.3, 4.5 Hz, 1H), 2.42 (s, 3H), 1.10 (m, 21H); ^{13}C NMR (100 MHz, $CDCl_3$) δ 167.6, 165.4, 163.0, 155.0, 137.4, 135.0, 132.1, 132.0, 128.7, 128.4, 127.8, 127.2, 96.9, 65.9, 63.5, 59.4, 55.2, 54.3, 42.8, 37.9, 18.0, 11.8; IR (neat) ν_{max}: 3239, 3059, 3030, 2942, 2866, 1755, 1567, 1108 cm^{-1}; HRMS(ESI+): Calcd for $C_{33}H_{46}N_5O_2Si^+$ $[M+H]^+$ 572.3415, found 572.3413, Δppm –0.35; mp: 86–88 ºC. The diastereomeric ratio was determined by LC-MS analysis of crude reaction mixture (Supplementary Fig. 11). The stereochemistry of this product was confirmed by NOE spectroscopy. 1D-NOE result supported the stereochemistry as shown Supplementary Fig. 46.

Synthetic procedure for the preparation of 11f

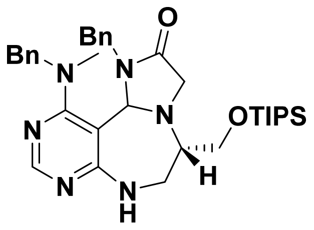

11f

Compound 11f. To a microwave vessel tightly sealed with a cap, were added **1f** (90mg, 0.198 mmol), *N*-benzyl-2-chloroacetamide (54.54 mg, 0.297 mmol) and NaBr (30.56 mg, 0.297 mmol) in DMF (3.0 ml). The resulting mixture was heated under microwave irradiation (150 watt) at 110 °C for 30 min. The reaction mixture was cooled to r.t. 1,8-Diazabicyclo[5.4.0]undec-7-ene (DBU, 0.059 ml, 0.396 mmol) was added and left to stir at r.t. After completion of the reaction as indicated by TLC, the reaction mixture was diluted with ethyl acetate, washed with saturated $NaHCO_3$(aq), dried over anhydrous Na_2SO_4(s), filtered, and concentrated *in vacuo*. The residue was purified by HPLC to obtain desired product **11f** (72.57 mg, 61%, d.r. 6:1) as a pale yellow oil. The diastereomeric ratio was determined by ^{1}H NMR spectroscopy (Supplementary Fig. 47).

R_f = 0.3 (hexane/EtOAc = 1:1); ^{1}H NMR (400 MHz, $CDCl_3$) δ 8.17 (s, 0.83H, *major diastereomer*), 8.12 (s, 0.13H, *minor diastereomer*), 7.28 (br s, 3H), 7.19 (br s, 3H), 6.89 (br s, 2H), 6.73 (br s, 2H), 6.11 (br s, 0.12H, *minor diastereomer*), 5.90 (s, 0.78H, *major diastereomer*), 5.28 (s, 0.84H, *major diastereomer*), 5.08 (s, 0.13H, *minor diastereomer*), 4.98 (m, 1H), 4.44, 4.34 (ABq, J_{AB} = 16.2 Hz, 2H), 3.78 (m, 2H), 3.66–3.37 (m, 4H), 3.24 (m, 2H), 2.73 (m, 3H), 1.05–0.95 (m, 21H); ^{13}C NMR (100 MHz, $CDCl_3$) δ 173.3, 167.1, 162.9, 157.3, 137.4, 136.0, 128.6, 128.5, 127.8, 127.1, 126.9, 126.7, 88.1, 77.3, 65.0, 59.6, 57.8, 52.0, 44.1, 42.9, 37.9, 18.03, 18.02, 11.9; IR (neat) ν_{max}: 3290, 3030, 2943, 2865, 1694, 1629, 1571, 1118 cm^{-1}; HRMS(ESI+): Calcd for $C_{34}H_{49}N_6O_2Si^+$ [M+H]$^+$ 601.3681, found 601.3693, Δppm +2.00.

Synthetic procedure for the preparation of 12f and 12f´

To a stirring solution of **1f** (180.0 mg, 0.397 mmol) in ACN (20 ml), benzyl bromide (0.071 ml, 0.596 mmol) was added and the resulting mixture was stirred at 80 °C. After the starting material was consumed as indicated by TLC, the organic solvent was removed under reduced pressure to afford the crude iminium ion. After washing with hexane to remove any excess benzyl bromide, ethynylmagnesium bromide (0.5 M solution in THF, 3.970 ml, 1.985 mmol) was added dropwise to a cooled solution of resultant iminium ion in dry THF (40 ml) over 30 min at –78 °C. The reaction was allowed to slowly warm to r.t over an 18 h period. The mixture was quenched with saturated NH_4Cl(aq) and extracted twice with DCM. The combined organic layer was dried over anhydrous Na_2SO_4(s) and the filtrate was condensed under reduced pressure, followed by silica-gel flash column chromatography to afford **12f** (major) and **12f´** (minor).

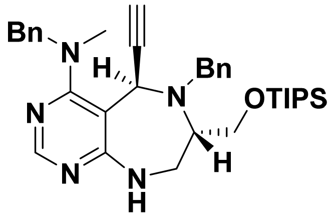

12f

Compound 12f (major). A pale yellow oil; R_f = 0.4 (hexane/EtOAc = 3:1); 158.4 mg, 70% yield; ^{1}H NMR (400 MHz, $CDCl_3$) δ 8.16 (s, 1H), 7.33–7.24 (m, 5H), 7.17–7.14 (m, 3H), 6.79–6.78 (m, 2H), 6.27 (m, 1H), 4.74 (d, *J* = 2.3 Hz, 1H), 4.64 (d, *J* = 14.9 Hz, 1H), 4.31, 4.26 (ABq, J_{AB} = 15.1 Hz, 2H), 3.92–3.85 (m, 2H), 3.78 (d, *J* = 14.5 Hz, 1H), 3.73 (dd, *J* = 10.0, 3.3 Hz, 1H), 3.54 (ddd, *J* = 14.4, 7.6, 1.4 Hz 1H), 2.93 (m, 1H), 2.60 (s, 3H), 2.53 (d, *J* = 2.3 Hz, 1H), 1.10–1.00 (m, 21H); ^{13}C NMR (100 MHz, $CDCl_3$) δ 167.4, 164.2, 155.8, 138.9, 138.3, 128.5, 128.4, 128.3, 127.6, 127.4, 126.7, 102.0, 83.9, 74.9, 65.3, 62.0, 58.5, 58.2, 48.5, 40.2, 39.6, 18.1, 12.0; IR (neat) ν_{max}: 3241, 2943, 2865, 1573, 1404, 1101, 669 cm^{-1}; HRMS(ESI+) Calcd for $C_{34}H_{48}N_5OSi^+$ $[M+H]^+$ 570.3623, found 570.3614, Δppm –1.58. The stereochemistry of this product was confirmed by NOE spectroscopy. 1D-NOE result supported the stereochemistry as shown in Supplementary Fig. 49.

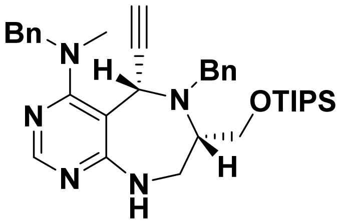

12f'

Compound 12f´ (minor). A pale yellow oil; R_f = 0.25 (hexane/EtOAc = 3:1); 18.78 mg, 8.3% yield; ^{1}H NMR (400 MHz, $CDCl_3$) δ 8.21 (s, 1H), 7.33–7.31 (m, 3H), 7.21–7.17 (m, 5H), 6.84 (m, 2H), 6.34 (m, 1H), 4.79 (m, 1H), 4.71 (d, *J* = 2.3 Hz, 1H), 4.54, 4.20 (ABq, J_{AB} = 15.5 Hz, 2H), 4.07 (t, *J* = 10.2 Hz, 1 H), 3.84, 3.71 (ABq, J_{AB} = 13.7 Hz, 2H), 3.64 (dd, *J* = 9.8, 5.1 Hz, 1H), 3.57 (ddd, *J* = 15.0, 7.7, 4.7 Hz 1H), 3.37 (m, 1H), 2.68 (s, 3H), 2.50 (d, *J* = 2.7 Hz, 1H), 1.04 (br s, 21H); ^{13}C NMR (100 MHz, $CDCl_3$) δ 168.7, 164.4, 156.1, 139.0, 138.2, 129.0, 128.5, 128.4, 127.6, 127.3, 126.8, 99.9, 87.5, 74.8, 68.3, 64.7, 60.7, 58.5, 46.7, 44.0, 38.9, 18.1, 12.0; IR (neat) $ν_{max}$: 3239, 2940, 2865, 1571, 1404, 1102, 698 cm^{-1}; HRMS(ESI+): Calcd for $C_{34}H_{48}N_5OSi^+$ [M+H]$^+$ 570.3623, found 570.3625, Δppm +0.35. The stereochemistry of this product was confirmed by NOE spectroscopy. 1D-NOE result supported the stereochemistry as shown in Supplementary Fig. 51.

Synthetic procedure for the preparation of 13f and 13f´.

Compound SI-7

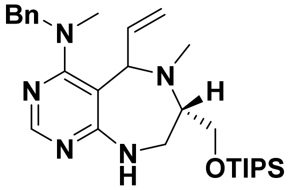

SI-7

To a stirring solution of **1f** (90 mg, 0.198 mmol) in ACN (10.0 ml), iodomethane (0.018 ml, 0.297 mmol) was added and the resulting mixture was stirred at 40 °C. After the starting

material was consumed as indicated by TLC, the organic solvent and any excess iodomethane were removed under reduced pressure to afford the crude iminium ion. To a cooled solution of resultant iminium ion in dry THF (20.0 ml) was added vinylmagnesium bromide (1.0 M solution in THF, 0.990 ml, 0.990 mmol) dropwise over 30 min at –78 °C. The reaction was allowed to slowly warm to r.t over an 18 h period. The mixture was quenched with saturated NH_4Cl(aq) and extracted twice with DCM. The combined organic layer was dried over anhydrous Na_2SO_4(s) and the filtrate was condensed under reduced pressure, followed by silica-gel flash column chromatography to afford **SI-7** (70.68 mg, 72% yield, d.r. 9:1) as a yellow oil (Supplementary Fig. 4). The diastereomeric ratio was determined by ^{1}H NMR spectroscopy (Supplementary Fig. 52).

R_f = 0.27 (DCM/MeOH = 20:1); ^{1}H NMR (400 MHz, $CDCl_3$) δ 8.22 (s, 0.08 H, *minor diastereomer*), 8.17 (s, 0.71H, *major diastereomer*), 7.36–7.24 (m, 6H, *major diastereomer+minor diastereomer*), 6.25 (m, 1H), 5.64 (br s, 0.12H, *minor diastereomer*), 5.29 (br s, 0.97H, *major diastereomer*), 5.20 (d, *J* = 10.2 Hz, 1H), 4.88 (d, *J* = 17.2 Hz, 1H), 4.56–4.45 (m, 3H), 5.20 (d, *J* = 10.2 Hz, 1H), 3.84–3.77 (m, 2H), 3.55 (t, *J* = 9.4 Hz, 1.11H, *major diastereomer*), 3.43 (t, *J* = 9.8 Hz, 0.15 H, *minor diastereomer*), 3.19 (m, 1H), 2.86–2.81 (m, 4H), 2.74 (s, 0.38H, *minor diastereomer*), 2.42 (s, 0.38H, *minor diastereomer*), 2.27 (s, 3H, *major diastereomer*), 1.10–1.02 (m, 25H, *major diastereomer+minor diastereomer*); ^{13}C NMR (100 MHz, $CDCl_3$) δ 167.6, 165.5, 155.4, 138.7, 136.5, 128.6, 127.3, 127.1, 118.2, 102.9, 64.9, 63.6, 62.8, 58.3, 44.2, 41.4, 39.4, 18.1, 12.0; HRMS(ESI+): Calcd for $C_{28}H_{46}N_5OSi^+$ [M+H]$^+$ 496.3466, found 496.3465, Δppm –0.20.

Compound 13f

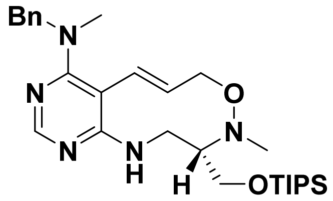

13f

To a solution of **SI-7** (70.68 mg, 0.143 mmol) in DCM (14.3 ml) was added 3-chloroperbenzoic acid (*m*-CPBA, 32.08 mg, 0.186 mmol) and the mixture was left to stir at reflux. After 1h,

solvent was removed under reduced pressure and the residue was purified by silica-gel flash column chromatography to obtain the desired product **13f** (42.45 mg, 58% yield, 42% overall yield) as a colorless oil (Supplementary Fig. 4).

R_f = 0.3 (hexane/EtOAc = 3:1); ^{1}H NMR (500 MHz, $CDCl_3$) δ 8.11 (s, 1H), 7.32 (m, 2H), 7.24 (m, 1H), 7.20 (m, 2H), 6.63 (d, *J* = 16.1 Hz, 1H), 6.09 (m, 1H), 4.82 (br s, 3H), 4.09 (m, 3H), 3.71 (m, 2H), 3.45–3.27 (m, 1H), 3.04 (s, 3H), 2.77 (m , 1H), 2.63–2.55 (m, 3H), 1.08 (m, 21H);); IR (neat) ν_{max}: 3395, 3254, 3060, 3028, 2926, 1946, 1713, 1567, 1493, 1452, 1266, 1105 cm^{-1}; HRMS(ESI+): Calcd for $C_{28}H_{46}N_5O_2Si^+$ $[M+H]^+$ 512.3415, found 512.3417, Δ ppm +0.39. The ^{1}H NMR spectrum of **13f** was too broad to analyze. As shown below, the structure of **13f** was fully confirmed only after treatment of 3-chlorobenzoic acid as a salt product, **13f**´, when broad peak of **13f** converted to sharp and well-resolved NMR signals.

Compound 13f´

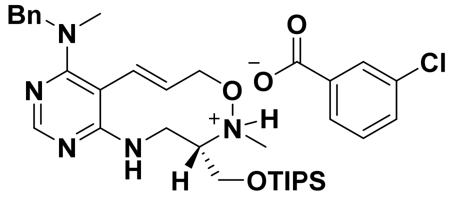

13f'

To a solution of **SI-7** (70.68 mg, 0.143 mmol) in DCM (14.3 ml) was added 3-chloroperbenzoic acid (*m*-CPBA, 32.08 mg, 0.186 mmol) and the mixture was left to stir at reflux. After 1 h, 3-chlorobenzoic acid (22.39mg, 0.143 mmol) was added and left to stir at r.t. After completion of the reaction as indicated by TLC, solvent was removed under reduced pressure and the residue was purified by silica-gel flash column chromatography to obtain the desired product **13f**´(70.72mg, 74% yield, 53% overall yield) as a pale yellow oil (Supplementary Fig. 4). The *E/Z* ratio (99:1) was determined by LC-MS analysis of the crude reaction mixtures (Supplementary Fig. 12).

R_f = 0.3 (hexane/EtOAc 1:1); ^{1}H NMR (500 MHz, $CDCl_3$) δ 8.19 (s, 1H), 7.95 (s, 1H, 3-chlorobenzoic acid-H), 7.85 (d, *J* = 7.8 Hz, 1H, 3-chlorobenzoic acid-H), 7.54 (d, *J* = 8.3 Hz,

1H, 3-chlorobenzoic acid-H), 7.37 (t, J = 7.8 Hz, 1H, 3-chlorobenzoic acid-H), 7.29–7.26 (m, 2H), 7.22–7.19 (m, 3H), 6.49 (d, J = 16.6 Hz, 1H), 5.96 (dt, J = 16.6, 5.9 Hz, 1H), 5.51 (t, J = 6.4 Hz, 1H), 4.74 (m, 2H), 4.66, 4.62 (ABq, J_{AB} = 15.0 Hz, 2H), 3.96 (m, 2H), 3.70 (dd, J = 9.8, 8.3 Hz, 1H), 3.55 (dt, J = 14.3, 5.6 Hz, 1H), 2.90 (s, 3H), 2.76 (m, 1H), 2.67 (s, 3H), 1.06 (m, 21H), ^{13}C NMR (100 MHz, $CDCl_3$) δ 165.1, 162.8, 161.2, 155.4, 138.4, 134.7, 133.3, 131.7, 129.84, 129.75, 128.9, 128.6, 127.8, 127.79, 127.2, 127.1, 96.3, 70.2, 65.6, 61.0, 56.0, 45.2, 40.5, 39.1, 18.1, 11.9; IR (neat) ν_{max}: 3396, 3062, 3026, 2945, 2863, 1724, 1572, 1253, 1117 cm^{-1}; HRMS(ESI+): Calcd for $C_{35}H_{51}ClN_5O_4Si^+$ [M+H]$^+$ 668.3393, found 668.3387, Δppm – 0.90.

Synthetic procedure for the preparation of 14f

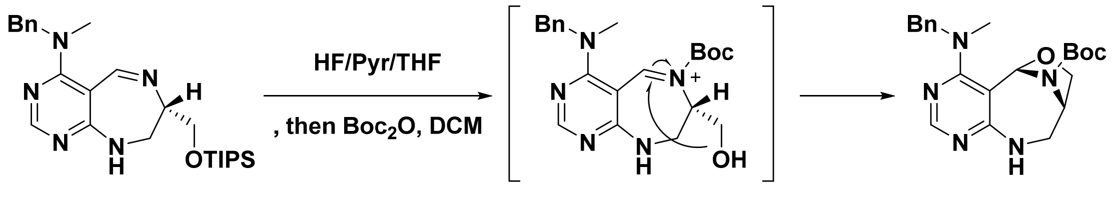

Compound 14f. 1f (90.00 mg, 0.198 mmol) was treated with HF/pyridine/THF (5/5/90) solution (2 ml) and then, the mixture was stirred at r.t. After the starting material was consumed as indicated by TLC, ethoxytrimethylsilane (2 ml) was added and allowed to react for 1 h to quench any excess HF (HF/pyridine protocol). The reaction mixture was condensed *in vacuo* to afford the crude product. To a solution of crude product in DCM (4 ml) was added Boc_2O (56.09 mg, 0.257 mmol) and then, the mixture was stirred at r.t. After completion of the reaction as indicated by TLC, solvent was removed under reduced pressure and the residue was purified through the recrystallization in hexane to obtain desired product **14f** (68.47 mg, 87% overall yield) as a white solid.

R_f = 0.3 (EA); ^{1}H NMR (400 MHz, $CDCl_3$) δ 8.13 (s, 1H), 7.35–7.24 (m, 5H), 6.62 (s, 1H), 5.63 (br s, 1H), 4.76 (m, 2H), 4.55 (d, J = 15.3 Hz, 1H), 4.01 (d, J = 7.0 Hz, 1H), 3.87 (t, J = 6.7 Hz, 1 H), 3.55 (br d, J = 13.6 Hz, 1H), 3.48–3.43 (m, 1H), 2.98 (s, 3H), 1.43 (s, 9H); ^{13}C NMR (100 MHz, $CDCl_3$) δ 166.7, 164.0, 155.8, 153.0, 138.3, 128.6, 127.9, 127.2, 103.0, 85.2,

81.6, 68.5, 57.5, 53.7, 48.0, 40.7, 28.5, 28.4, 28.3; IR (neat) ν_{max}: 3248, 2975, 2931, 1700, 1571, 1403, 1162, 1048, 699 cm^{-1}; HRMS(ESI+): Calcd for $C_{21}H_{28}N_5O_3^+$ $[M+H]^+$ 398.2187, found 398.2174, Δppm –3.26; mp: 50–52 ºC.

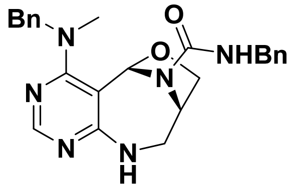

15f

Compound 15f. A white solid; R_f = 0.4 (EA); 77.57 mg, 91% yield; ^{1}H NMR (500 MHz, $CDCl_3$) δ 8.18 (s, 1H), 7.29–7.28 (m, 2H), 7.25–7.24 (m, 4H), 7.19–7.18 (m, 2H), 7.14 (d, *J* = 6.4 Hz, 2H), 6.57 (s, 1H), 5.55 (br s, 1H), 4.86 (t, *J* = 5.4 Hz, 1 H), 4.74 (m, 1H), 4.46 (br d, *J* = 1.5 Hz, 2H), 4.32 (d, *J* = 5.4 Hz, 2H), 4.02 (dd, *J* = 7.3, 1.0 Hz, 1H), 3.91 (m, 1H), 3.55 (m, 1H), 3.38 (dt, *J* = 13.2, 4.2 Hz, 1H), 2.83 (s, 3H); ^{13}C NMR (100 MHz, $CDCl_3$) δ 166.8, 164.1, 156.0, 154.9, 138.8, 137.8, 128.7, 128.6, 127.7, 127.5, 127.4, 127.3, 104.3, 84.0, 68.7, 58.2, 53.7, 48.0, 44.7, 40.3; IR (neat) ν_{max}: 3258, 2971, 1633, 1542, 1405, 1049, 698 cm^{-1}; HRMS(ESI+): Calcd for $C_{24}H_{27}N_6O_2^+$ $[M+H]^+$ 431.2190, found 431.2184, Δppm –1.39; mp: 67–69 ºC. The product was synthesized according to the synthetic procedure for the preparation of **14f** from **1f** and benzyl isocyanate (1.3 equiv.).

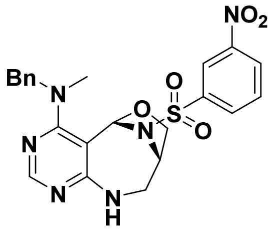

16f

Compound 16f. A white solid; R_f = 0.3 (EA); 59.2 mg, 62% yield; ^{1}H NMR (400 MHz, DMSO-d_6) δ 8.54–8.53 (m, 2H), 8.02 (s, 1H), 7.98 (d, *J* = 7.8 Hz, 1H), 7.83 (t, *J* = 8.2 Hz, 1H),

7.39 (m, 2H), 7.26 (m, 4H), 6.63 (s, 1H), 4.84 (br s, 1H), 4.65, 4.54 (ABq, J_{AB} = 15.6 Hz, 2H), 3.61 (d, J = 7.4 Hz, 1H), 3.51–3.45 (m, 1H), 3.28 (d, J = 14.1 Hz, 1H), 2.88 (m, 4H); ^{13}C NMR (400 MHz, DMSO-d_6) δ 165.6, 163.2, 155.8, 148.0, 138.9, 138.1, 133.3, 131.7, 128.5, 127.4, 127.1, 122.4, 122.3, 101.4, 87.3, 66.7, 57.4, 55.8, 54.9, 47.8, 40.0; IR (neat) ν_{max}: 2920, 1573, 1535, 1354, 1176, 734 cm^{-1}; HRMS(ESI+): Calcd for $C_{22}H_{23}N_6O_5S^+$ [M+H]$^+$ 483.1445, found 483.1450, Δppm +1.03; mp: 101–103 °C. The product was synthesized according to the synthetic procedure for the preparation of **14f** from **1f** and 3-nitrobenzene sulfonyl chloride (*m*-NsCl) (1.3 equiv.). Purified by silica-gel flash column chromatography to afford **16f**. The structure of **16f** was confirmed by X-ray crystallographic analysis (Supplementary Fig. 5, Supplementary Table 1–5, CCDC number 1500586).

Synthetic procedure for the preparation of 17f´.

Compound SI-8.

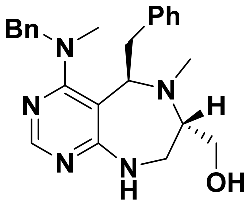

SI-8

To a stirring solution of **1f** (180 mg, 0.397 mmol) in ACN (20.0 ml), iodomethane (0.036 ml, 0.594 mmol) was added and the resulting mixture was stirred at 40 °C. After the starting material was consumed as indicated by TLC, the organic solvent and any excess iodomethane were removed under reduced pressure to afford the crude iminium ion. To a cooled solution of resultant iminium ion in dry THF (40.0 ml) was added benzylmagnesium bromide (2.0 M solution in THF, 0.99 ml, 1.98 mmol) dropwise over 30 min at –78 °C. The reaction was allowed to slowly warm to r.t over an 18 h period. The mixture was quenched with saturated NH_4Cl(aq) and extracted twice with DCM. The combined organic layer was dried over anhydrous Na_2SO_4(s) and the filtrate was condensed under reduced pressure, followed by

filtration through a short pad of silica gel with hexane/EtOAc (1:1) to afford crude product. To a solution of crude product in THF (4 ml) was added TBAF (1.0 M solution in THF, 0.516 ml, 0.516 mmol). The reaction mixture was then left to stir at r.t. After completion of the reaction as indicated by TLC, the solvent was removed under reduced pressure and the residue was purified through the silica-gel flash column chromatography to obtain product **SI-8** (121.8 mg, 76% yield) as a white solid (Supplementary Fig. 6).

R_f = 0.4 (DCM/MeOH = 10:1); ^{1}H NMR (400 MHz, $CDCl_3$) δ 8.13 (s, 1H), 7.32–7.25 (m, 3H), 7.20–7.13 (m, 3H), 6.99 (d, *J* = 7.2 Hz, 2H), 6.73 (d, *J* = 6.7 Hz, 2H), 5.40 (d, *J* = 5.9 Hz, 1H), 4.35, 4.28 (ABq, J_{AB} = 16.0, 15.6 Hz, 2H), 4.19 (dd, *J* = 9.6, 4.5 Hz, 1H), 3.60 (dd, *J* = 10.6, 5.1 Hz, 1H), 3.46 (dd, *J* = 10.8, 5.7 Hz, 1H), 3.31–3.04 (m, 4H), 2.88 (dd, *J* = 12.9, 4.3 Hz, 1H), 2.62 (s, 3H), 2.51 (br s, 1H), 2.32 (s, 3H); ^{13}C NMR (100 MHz, $CDCl_3$) δ 168.4, 166.1, 154.9, 139.3, 138.1, 129.0, 128.6, 128.1, 127.4, 127.1, 126.2, 103.3, 65.0, 61.7, 58.5, 58.0, 46.2, 38.7, 38.6, 34.5; HRMS (ESI+): Calcd for $C_{24}H_{30}N_5O^+$ $[M+H]^+$ 404.2445, found 404.2446, Δppm +0.25; mp: 70–72 °C.

Compound 17f and 17f´. To a stirring solution of **SI-8** (121.8 mg, 0.302 mmol) in toluene (3.0 ml) under argon atmosphere were sequentially added Et_3N (0.126 ml, 0.906 mmol) and trifluoromethanesulfonic anhydride (Tf_2O) (0.112 ml, 0.664 mmol) at –40 °C and left to stir. After 1 h, the temperature of the reaction mixture was brought down to –78 °C and NaN_3 (58.9 mg, 0.906 mmol) was added. The reaction was allowed to slowly warm to r.t over an 18 h period. The resultant was quenched with saturated $NaHCO_3$(aq) and extracted twice with DCM. The combined organic layer was dried over anhydrous Na_2SO_4(s) and the filtrate was condensed under reduced pressure, followed by silica-gel flash column chromatography to afford the **17f** (Path A, eight-membered ring) and **17f´** (Path B, seven-membered ring) as shown in Supplementary Fig. 6.

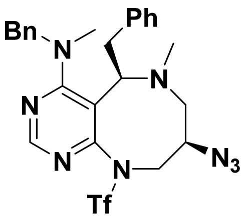

17f

Compound 17f (Path A, eight-membered ring, Supplementary Fig. 6). A white solid; R_f = 0.6 (hexane/EtOAc = 3:1); 37.2 mg, 22% yield, 17% overall yield; ^{1}H NMR (800 MHz, $CDCl_3$) δ 8.29 (s, 1H), 7.32 (m, 2H), 7.28 (m, 1H), 7.17 (m, 2H), 7.13 (m, 1H), 7.00 (d, *J* = 7.3 Hz, 2H), 6.74 (d, *J* = 7.3 Hz, 2H), 4.33, 4.27 (ABq, J_{AB} = 15.7 Hz, 2H), 4.23 (dd, *J* = 14.9, 6.6 Hz, 1H), 3.99 (dd, *J* = 10.3, 3.9 Hz, 1H), 3.96 (dd, *J* = 15.7, 1.5 Hz, 1H), 3.89 (m, 1H), 3.38 (dd, *J* = 14.9, 10.5 Hz, 1H), 3.07 (m, 1H), 2.98 (dd, *J* = 12.5, 3.7 Hz, 1H), 2.84 (br dd, *J* = 16.0, 1.6 Hz, 1H), 2.66 (s, 3H), 2.59 (s, 3H); ^{13}C NMR (100 MHz, $CDCl_3$) δ 169.2, 160.3, 154.0, 138.1, 136.7, 129.1, 128.8, 128.3, 127.64, 127.57, 126.5, 120.7 (q, $^1J_{C,F}$ = 324.4 Hz), 115.0, 66.0, 59.9, 57.4, 56.0, 52.1, 49.1, 38.8, 37.5; IR (neat) ν_{max}: 3028, 2929, 2868, 2108, 1566, 1393 cm^{-1}; HRMS(ESI+) Calcd for $C_{25}H_{28}F_3N_8O_2S^+$ $[M+H]^+$ 561.2003, found 561.2009, Δppm +1.07; mp: 103–105 °C. The stereochemistry of this product was confirmed by NOE spectroscopy. 1D-NOE result supported the stereochemistry as shown in Supplementary Fig. 60.

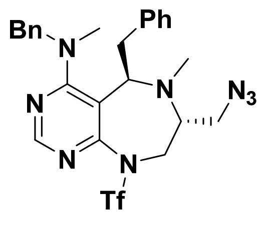

17f'

Compound 17f´ (Path B, seven-membered ring, Supplementary Fig. 6). A white solid; R_f = 0.5 (hexane/EtOAc = 3:1); 55.9 mg, 33% yield, 25% overall yield; ^{1}H NMR (500 MHz, $CDCl_3$) δ 8.32 (s, 1H), 7.34 (m, 2H), 7.29 (m, 1H), 7.14 (m, 3H), 6.96 (d, *J* = 7.3 Hz, 2H), 6.73 (br d, *J* = 7.3 Hz, 2H), 4.56, 4.49 (ABq, J_{AB} = 16.5 Hz, 2H), 4.23 (d, *J* = 15.2 Hz, 1 H), 3.91 (dd, *J* =

11.2, 2.9 Hz, 1H), 3.67 (dd, J = 13.7, 4.4 Hz, 1H), 3.50 (dd, J = 14.9, 10.5 Hz, 1H), 3.32 (m, 1H), 3.19 (dd, J = 13.7, 2.4 Hz, 1H), 3.10 (t, J = 11.8 Hz, 1H), 2.96 (dd, J = 13.0, 3.2 Hz, 1H), 2.78 (s, 3H), 2.31 (s, 3H); ^{13}C NMR (100 MHz, $CDCl_3$) δ 168.1, 159.7, 154.7, 138.3, 137.2, 129.1, 128.8, 128.3, 127.5, 127.0, 126.5, 120.1 (q, $^{1}J_{C,F}$ = 322.6 Hz), 110.1, 66.6, 59.6, 57.4, 56.3, 51.5, 42.1, 38.0, 31.5; IR (neat) ν_{max}: 3030, 2930, 2869, 2105, 1578, 1529, 1391, 1212, 1043 cm^{-1}; HRMS(ESI+) Calcd for $C_{25}H_{28}F_3N_8O_2S^+$ [M+H]$^+$ 561.2003, found 561.2011, Δppm +1.43; mp: 140–142 °C. The stereochemistry of this product was confirmed by NOE spectroscopy. 1D-NOE result supported the stereochemistry as shown in Supplementary Fig. 62.

Synthetic procedure for the preparation of 18f

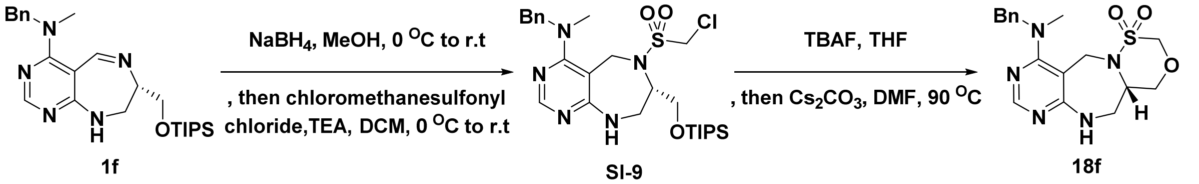

Compound 18f. To a stirring solution of **1f** (90mg, 0.198 mmol) in MeOH (2 ml) was added $NaBH_4$ (36.88 mg, 0.975 mmol) at 0 °C. The resulting mixture was left to stir and allowed to warm to r.t. After the starting material was consumed as indicated by TLC, the resultant was quenched with saturated $NaHCO_3$(aq) and extracted twice with DCM. The combined organic layer was dried over anhydrous Na_2SO_4(s) and filtered. The solvent was evaporated under reduced pressure, and the resulting crude secondary amine was dissolved in DMF (2 ml). To this solution were sequentially added Et_3N (0.055 ml, 0.396 mmol) and chloromethane sulfonyl chloride (0.023 ml, 0.257 mmol) at 0 °C. The resulting mixture was left to stir and allowed to warm to r.t. After completion of the reaction as indicated by TLC, the resultant was quenched with saturated NH_4Cl(aq) and extracted twice with DCM. The combined organic layer was dried over anhydrous Na_2SO_4(s) and the filtrate was condensed under reduced pressure, followed by silica-gel flash column chromatography to afford **SI-9** (97.89 mg, 87% yield). To a solution of **SI-9** (97.89 mg, 0.172 mmol) in THF (2 ml) was added TBAF (1.0 M solution in THF, 0.224 ml, 0.224 mmol). The reaction mixture was then left to stir at r.t. After completion of the reaction as indicated by TLC, the solvent was removed under reduced pressure. To a solution of the crude resultant in DMF (2 ml) was added Cs_2CO_3 (168.1 mg, 0.516 mmol). Then the resulting mixture was stirred at 90 °C. After completion of the reaction as indicated

by TLC, the reaction mixture was diluted with ethyl acetate, washed with brine, dried over anhydrous Na_2SO_4(s), filtered, and concentrated *in vacuo*. The residue was purified with silica-gel flash column chromatography to obtain desired product **18f** (51.02 mg, 79% yield, 69% overall yield) as a white solid.

R_f = 0.4 (DCM/MeOH = 20:1); ^{1}H NMR (400 MHz, DMSO-d_6) δ 7.98 (s, 1H), 7.34–7.23 (m, 6H), 4.84 (d, *J* = 11.7 Hz, 1H), 4.71–4.59 (m, 3H), 4.47 (d, *J* = 15.3 Hz, 1H), 4.33 (d, *J* = 15.7 Hz, 1H), 4.19 (br s, 1H), 3.89 (d, *J* = 10.4 Hz, 1H), 3.80 (t, *J* = 11.2 Hz, 1H), 3.54 (br s, 1H), 3.38 (br s, 1H), 2.81 (s, 3H); ^{13}C NMR (100 MHz, DMSO-d_6) δ 165.0, 163.2, 153.5, 138.1, 128.4, 127.5, 127.0, 95.6, 62.0, 55.4, 40.2, 39.4; IR (neat) ν_{max}: 3230, 2926, 1574, 1409, 1155, 736 cm^{-1}; HRMS(ESI+): Calcd for $C_{17}H_{22}N_5O_3S^+$ [M+H]$^+$ 376.1438, found 376.1437, Δppm –0.27; mp: 196–198 °C.

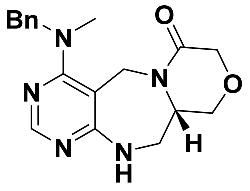

19f

Compound 19f. A colorless oil; R_f = 0.5 (DCM/MeOH = 20:1); 50.40 mg, 75% overall yield; ^{1}H NMR (400 MHz, $CDCl_3$) δ 8.02 (s, 1H), 7.39–7.33 (m, 4H), 7.27–7.23 (m, 1H), 6.97 (m, 1H), 5.23 (d, *J* = 15.7 Hz, 1H), 4.78, 4.41 (ABq, J_{AB} = 15.7 Hz, 2H), 4.21, 4.12 (ABq, J_{AB} = 16.4 Hz, 2H), 4.09–4.01 (m, 2H), 3.97–3.87 (m, 2H), 3.78 (t, *J* = 11.0 Hz, 1H), 3.00 (dd, *J* = 15.3, 7.4 Hz, 1H), 2.89 (s, 3H); ^{13}C NMR (100 MHz, $CDCl_3$) δ 166.7, 166.5, 164.3, 154.7, 137.8, 128.5, 127.5, 127.1, 95.4, 67.7, 66.5, 57.5, 56.2, 42.2, 40.7, 39.1; IR (neat) ν_{max}: 3237, 2908, 1653, 1573, 1408, 1119, 737 cm^{-1}; HRMS(ESI+): Calcd for $C_{18}H_{22}N_5O_2^+$ [M+H]$^+$ 340.1768, found 340.1765, Δppm –0.88. The product was synthesized according to the synthetic procedure for the preparation of **18f** from **1e** and chloroacetic anhydride.

Synthetic procedure for the preparation of 20f

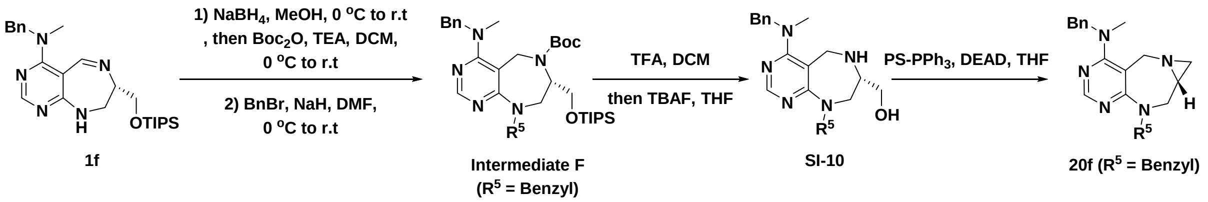

Compound 20f. To a stirring solution of **1f** (135.0 mg, 0.298 mmol) in MeOH (3.0 ml) was added $NaBH_4$ (56.37 mg, 1.490 mmol) at 0 °C. Then the resulting mixture was left to stir and allowed to warm to r.t. After the starting material was consumed as indicated by TLC, the resultant was quenched with saturated $NaHCO_3$(aq) and extracted twice with DCM. The combined organic layer was dried over anhydrous Na_2SO_4(s) and filtered. The solvent was evaporated under reduced pressure, and the resulting crude secondary amine was dissolved in DCM (3.0 ml). To this solution were sequentially added Et_3N (0.083 ml, 0.596 mmol) and Boc_2O (84.55 mg, 0.387 mmol) at 0 °C. The resulting mixture was left to stir and allowed to warm to r.t. After completion of the reaction as indicated by TLC, the resultant was quenched with saturated NH_4Cl(aq) and extracted twice with DCM. The combined organic layer was dried over anhydrous Na_2SO_4(s) and the filtrate was condensed under reduced pressure, followed by silica-gel flash column chromatography to afford the Boc-protected product (162.3 mg, 98% yield). To a solution of Boc-protected product (162.3 mg, 0.292 mmol) in DMF (3.0 ml) under argon atmosphere was added NaH (60% dispersion in mineral oil, 23.36 mg, 0.584 mmol) at 0 °C and left to stir. After 30 min, benzylbromide (0.052 ml, 0.438 mmol) was slowly added. The resulting mixture was left to stir and allowed to warm to r.t. After completion of the reaction as indicated by TLC, the reaction mixture was quenched with saturated NH_4Cl(aq). The resultant was extracted twice with ethyl acetate, dried with anhydrous Na_2SO_4(s), filtered, and concentrated *in vacuo*. The residue was purified by silica-gel flash column chromatography to obtain the intermediate **F** (R^5 = benzyl, 164.1 mg, 87% yield). Intermediate **F (**164.1 mg, 0.254 mmol) was first treated with 10% trifluoroacetic acid (TFA) in DCM (12.5 ml) at r.t. After the starting material was consumed as indicated by TLC, any excess TFA was removed by azeotropic evaporation with toluene under reduced pressure. To a solution of the resulting Boc-deprotected product in THF (2.5 ml) was added TBAF (1.0 M solution in THF, 0.330 ml, 0.330 mmol) and the mixture was stirred at r.t. After completion of the reaction as indicated by

TLC, the reaction mixture was quenched with saturated $NaHCO_3$(aq). The resultant was extracted twice with DCM, dried with anhydrous Na_2SO_4(s), filtered, and concentrated *in vacuo*, followed by silica-gel flash column chromatography to afford **SI-10** (73.21 mg, 74% yield). To a **SI-10 (**73.21 mg, 0.188 mmol) in THF (20.0 ml) under argon atmosphere was added polymer-bound triphenylphosphine (587.5 mg, 0.94 mmol), purchased from Sigma-Aldrich (100-200 mesh, 1.6 mmol/g), and the mixture was stirred at r.t. After 30 min, diehtyl azodicarboxylate (DEAD, 0.058 ml, 0.376 mmol) was slowly added. The mixture was stirred at r.t for 18h. The reaction mixture was filtered through Celite® while washing with DCM. The filtrate was condensed under reduced pressure and purified by silica-gel flash column chromatography to afford the desired product **20f** (R^5 = benzyl) (56.57 mg, 81% yield, 52% overall yield) as a pale yellow oil.

R_f = 0.4 (DCM/MeOH 20:1); ^{1}H NMR (400 MHz, $CDCl_3$) δ 8.24 (s, 1H), 7.35–7.27 (m, 10H), 5.10, 4.88 (ABq, J_{AB} = 15.3 Hz, 2H), 4.61, 4.39 (ABq, J_{AB} = 15.3 Hz, 2H), 4.26 (d, J = 13.7, 1H), 4.09 (dd, J = 15.3, 11.3 Hz, 1H), 3.58 (d, J = 13.7, 1H), 3.42 (dd, J = 15.5, 4.1 Hz, 1H), 2.82 (s, 3H), 2.43 (m, 1H), 1.78 (d, J = 5.1, 1H), 1.30 (d, J = 3.1, 1H); ^{13}C NMR (100 MHz, $CDCl_3$) δ 167.4, 163.8, 155.4, 138.8, 138.7, 128.7, 128.6, 127.8, 127.6, 127.3, 127.1, 100.3, 57.8, 53.9, 51.5, 48.7, 39.7, 34.4, 32.8; IR (neat) ν_{max}: 3027, 2920, 2853, 1559, 1356, 735, 700 cm^{-1}; HRMS(ESI+): Calcd for $C_{23}H_{26}N_5^+$ $[M+H]^+$ 372.2183, found 372.2189, Δppm +1.61.

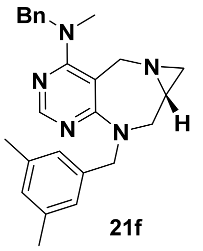

21f

Compound 21f (R^5 = 3,5-dimehtylbenzyl). A pale yellow oil; R_f = 0.5 (DCM/MeOH = 20:1); 49.82 mg, 81% yield, 53% overall yield; ^{1}H NMR (500 MHz, $CDCl_3$) δ 8.25 (s, 1H), 7.34–7.25 (m, 5H), 6.91 (m, 3H), 5.04, 4.78 (ABq, J_{AB} = 15.2 Hz, 2H), 4.61, 4.38 (ABq, J_{AB} = 15.2 Hz, 2H), 4.26 (d, J = 13.7, 1H), 4.04 (dd, J = 14.2, 11.7 Hz, 1H), 3.57 (d, J = 13.7, 1H), 3.41 (dd, J = 15.4, 2.2 Hz, 1H), 2.81 (s, 3H), 2.43(m, 1H), 2.29 (s, 6H), 1.77 (d, J = 2.9, 1H), 1.28 (s, 1H); ^{13}C NMR (100 MHz, $CDCl_3$) δ 167.4, 163.8, 155.4, 138.8, 138.6, 138.3, 129.0, 128.6, 127.8, 127.1, 125.4, 100.3, 57.9, 53.7, 51.5, 48.4, 39.7, 34.5, 32.7, 21.5; IR (neat) ν_{max}: 3026,

2922, 2863, 1564, 1355, 735, 700 cm^{-1}; HRMS(ESI+): Calcd for $C_{25}H_{30}N_5^+$ $[M+H]^+$ 400.2496, found 400.2495, Δppm –0.25. The product was synthesized according to the synthetic procedure for the preparation of **20f** from **1f** and 3,5-dimehtylbenzyl bromide.

Synthetic procedure for the preparation of 22f

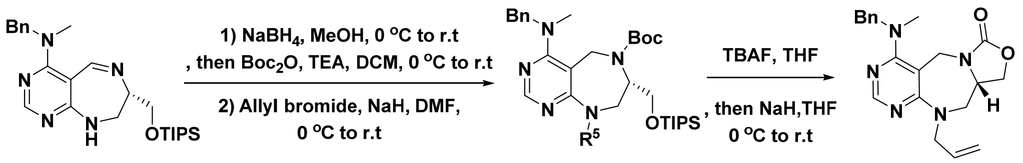

1f Intermediate F (R^5 = allyl) 22f

Compound 22f. To a stirring solution of **1f** (135.0 mg, 0.298 mmol) in MeOH (3.0 ml) was added $NaBH_4$ (56.37 mg, 1.490 mmol) at 0 °C. Then the resulting mixture was left to stir and allowed to warm to r.t. After the starting material was consumed as indicated by TLC, the resultant was quenched with saturated $NaHCO_3$(aq) and extracted twice with DCM. The combined organic layer was dried over anhydrous Na_2SO_4(s) and filtered. The solvent was evaporated under reduced pressure, and the resulting crude secondary amine was dissolved in DCM (3.0 ml). To this solution were sequentially added Et_3N (0.083 ml, 0.596 mmol) and Boc_2O (84.55 mg, 0.387 mmol) at 0 °C. The resulting mixture was left to stir and allowed to warm to r.t. After completion of the reaction as indicated by TLC, the resultant was quenched with saturated NH_4Cl(aq) and extracted twice with DCM. The combined organic layer was dried over anhydrous Na_2SO_4(s) and the filtrate was condensed under reduced pressure, followed by silica-gel flash column chromatography to afford the Boc-protected product (162.3 mg, 98% yield). To a solution of Boc-protected product (162.3 mg, 0.292 mmol) in DMF (3.0 ml) under argon atmosphere was added NaH (60% dispersion in mineral oil, 23.36 mg, 0.584 mmol) at 0 °C and left to stir. After 30 min, allyl bromide (0.038 ml, 0.438 mmol) was slowly added. The resulting mixture was left to stir and allowed to warm to r.t. After completion of the reaction as indicated by TLC, the reaction mixture was quenched with saturated NH_4Cl(aq). The resultant was extracted twice with ethyl acetate, dried with anhydrous Na_2SO_4(s), filtered, and concentrated *in vacuo*. The residue was purified by silica-gel flash column chromatography

to obtain the intermediate **F** (R^5 = allyl, 141.2 mg, 81% yield). To a solution of intermediate **F** (141.2 mg, 0.237 mnol) in THF (2.5 ml) was added TBAF (1.0 M solution in THF, 0.308 ml, 0.308 mmol) and the mixture was stirred at r.t. After completion of the reaction as indicated by TLC, the reaction mixture was quenched with saturated $NaHCO_3$(aq). The resultant was extracted twice with DCM, dried with anhydrous Na_2SO_4(s), filtered, and concentrated *in vacuo*. To a solution of the crude resultant in THF (2.5 ml) under argon atmosphere was added NaH (60% dispersion in mineral oil, 14.22 mg, 0.356 mmol) at 0 °C. Then the resulting mixture was left to stir and allowed to warm to r.t. After completion of the reaction as indicated by TLC, the reaction mixture was diluted with DCM, washed with brine, dried over anhydrous Na_2SO_4(s), filtered, and concentrated *in vacuo*. The residue was purified with silica-gel flash column chromatography to obtain desired product **22f** (56.30 mg, 65% yield, 53% overall yield) as a white solid.

R_f = 0.2 (EA); ^{1}H NMR (400 MHz, $CDCl_3$) δ 8.23 (s, 1H), 7.36–7.31 (m, 4H), 7.28 (m, 1H), 5.92 (m, 1H), 5.21 (d, *J* = 10.2 Hz, 1H), 5.15 (dd, *J* = 17.2, 1.2 Hz, 1H), 4.72–4.65 (m, 2H), 4.61 (dd, *J* = 15.5, 4.9 Hz, 1H), 4.55–4.44 (m, 2H), 4.11–4.07 (m, 2H), 4.02 (d, *J* = 16.0 Hz, 1H), 3.91 (dd, *J* = 15.5, 6.5 Hz, 1H), 3.76 (dd, *J* = 14.9, 2.0 Hz, 1H), 3.25 (dd, *J* = 15.1, 3.3 Hz, 1H), 2.91 (s, 3H); ^{13}C NMR (100 MHz, $CDCl_3$) δ 166.9, 164.9, 157.3, 155.0, 138.1, 134.2, 128.7, 127.9, 127.3, 117.8, 97.0, 65.1, 56.9, 56.7, 54.5, 52.4, 42.1, 39.7; IR (neat) ν_{max}: 3049, 2976, 2917, 1758, 1561, 1410, 1266, 746 cm^{-1}; HRMS(ESI+): Calcd for $C_{20}H_{24}N_5O_2^+$ [M+H]$^+$ 366.1925, found 366.1924, Δppm –0.27; mp: 89–91 °C.

Synthetic procedure for the preparation of 23f

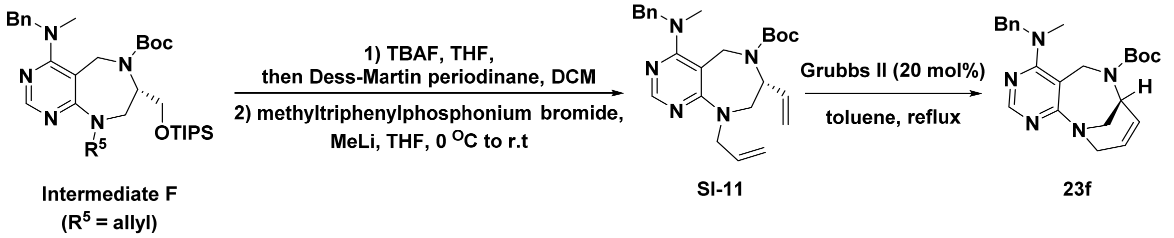

Compound 23f. To a solution of intermediate **F** (R^5 = allyl, 243.1 mg, 0.408 mmol) in THF (4.1 ml) was added TBAF (1.0 M solution in THF, 0.530 ml, 0.530 mmol) and the mixture was stirred at r.t. After completion of the reaction as indicated by TLC, the reaction mixture was quenched with saturated $NaHCO_3$(aq). The resultant was extracted twice with DCM, dried with anhydrous Na_2SO_4(s), filtered, and concentrated *in vacuo*. To a solution of the crude resultant in DCM (8.2 ml) was added Dess-Martin periodinane (259.6 mg, 0.612 mmol) and the mixture was stirred at r.t. After completion of the reaction as indicated by TLC, the reaction mixture was quenched with saturated $NaHCO_3$(aq). The resultant was extracted twice with DCM, dried with anhydrous Na_2SO_4(s), filtered, and concentrated *in vacuo*. The residue was purified by silica-gel flash column chromatography to obtain the aldehyde product (148.3 mg, 83% yield). To a stirring suspension of methyltriphenylphosphonium bromide (363.3 mg, 1.017 mmol) in THF under argon atmosphere was added MeLi (1.6 M in diethyl ether, 0.530 ml, 0.848 mmol) at 0 °C. After 30min, the resulting aldehyde product (148.3 mg, 0.339 mmol) was added. Then the reaction mixture was allowed to slowly warm to r.t over a 12 h period. The reaction mixture was quenched with saturated NH_4Cl(aq). The resultant was extracted twice with ethyl acetate, dried with anhydrous Na_2SO_4(s), filtered, and concentrated *in vacuo*, followed by silica-gel flash column chromatography to afford **SI-11** (130.0 mg, 88% yield). To a solution of **SI-11** (130.0 mg, 0.298 mmol) in toluene was added second generation Grubbs' catalyst (50.94 mg, 0.060 mmol, 20 mol%) and the mixture was left to stir at reflux. After 4h, solvent was removed under reduced pressure and the residue was purified by silica-gel flash column chromatography to obtain desired product **23f** (44.83 mg, 52% recovery yield, 31% overall yield) as a yellow oil.

R_f = 0.2 (hexane/EA = 1:1); ^{1}H NMR (400 MHz, DMSO-d_6, 100 °C) δ 8.06 (s, 1H), 7.35–7.29 (m, 4H), 7.27–7.23 (m, 1H), 6.35 (ddd, J = 9.9, 5.3, 1.8 Hz, 1H), 5.78 (ddd, J = 10.1, 3.9, 1.8 Hz, 1H), 4.92 (d, J = 16.5 Hz, 1H), 4.86–4.81 (m, 1H), 4.76 (d, J = 15.3 Hz, 1H), 4.56 (d, J =

15.9 Hz, 1H), 4.42 (dd, *J* = 15.3, 1.2 Hz, 1H), 4.34 (d, *J* = 15.3 Hz, 1H), 4.00 (dd, *J* = 4.9, 1.8 Hz, 1H), 3.77 (dq, *J* = 18.3, 2.2 Hz, 1H), 3.30 (dd, *J* = 15.3, 2.4 Hz, 1H), 2.84 (s, 3H), 1.42 (s, 9H); ^{13}C NMR (100 MHz, DMSO-d_6, 100 °C) δ 166.0, 163.6, 154.6, 153.5, 137.9, 128.0, 127.7, 127.3, 127.1, 126.3, 99.7, 79.0, 55.4, 49.9, 49.4, 45.7, 41.9, 38.7, 27.7; IR (neat) ν_{max}: 3031, 2978, 2928, 1690, 1588, 1408, 1164 cm^{-1}; HRMS(ESI+): Calcd for $C_{23}H_{30}N_5O_2^+$ [M+H]$^+$ 408.2394, found 408.2382, Δppm –2.94.

3. General Information of Bioassay

Kits, reagents and materials

Micro BCA™ protein assay kit was purchased from PIERCE and used for the measurement of protein concentration of cell lysate. Cell proliferation enzyme-linked immunosorbent assay (ELISA) and 5-bromo-2'-deoxyuridine (BrdU) colorimetric kit were purchased from Roche, Sigma-Aldrich. Ez-cytox WST-based cell viability, proliferation & cytotoxicity assay kit was purchased from Daeil Lab. Compounds used for bioassays were prepared in dimethyl sulfoxide (DMSO) solution. DMSO was purchased from Acros Organics. Tween® 20 was purchased from Sigma-Aldrich. Dulbecco modified eagle medium (DMEM), RPMI 1640, fetal bovine serum (FBS), and antibiotic-antimycotic solution were purchased from Gibco, Invitrogen. Phosphate-buffered saline (PBS) buffer and leucine-free DMEM were purchased from WELGENE. 3,3', 5,5''-tetramethylbenzidine (TMB), a substrate of horseradish peroxidase (HRP) conjugated in secondary antibody, was purchased from Invitrogen. Protein gel casters and western blot equipments including polyvinylidene difluoride (PVDF) membrane were purchased from Bio-Rad. Amersham ECL prime western blotting detection system (Amersham ECL prime solution) was purchased from GE Healthcare Life Science. 100-mm cell culture dish, transparent 96-well plate [#3598], and half-bottom 96 well clear plate [#3690] were purchased from CORNING. Lipofectamine™ 2000 reagent was purchased from Invitrogen. Human embryonic kidney (HEK)293T, DU145, Ca Ski, and HeLa cells were obtained from American Type Culture Collection [ATCC, VA, USA]. Nunc™ Lab-Tek™ II chambered cover glass was purchased from Thermo Scientific.

Antibodies, plasmids and proteins

Anti-LC3B (ab51520), anti-S6K1 (ab32359), anti-phsopho-T389 S6K1 (ab2571), and HRP-labeled anti-horse IgG secondary antibodies (ab6802) were purchased from Abcam. Anti-glutathione-S-transferase (GST) antibody (sc-459) was purchased from Santa Cruz Biotechnology. Anti-p62 (CST 5114), anti-glyceraldehyde-3-phosphate dehydrogenase (GAPDH) (CST 2118), anti-phospho-S65 4E-BP1 (CST 9451), anti-phospho-S757 ULK1

(CST 6888), anti-phospho-S473 Akt (CST 4058), anti-phospho-T172 AMPKα (CST 2531), and HRP-labeled anti-rabbit IgG secondary antibodies (CST 7074) were purchased from Cell Signaling Technology. His-tagged LRS and GST-tagged RagD were in our laboratory stocks. mCherry-GFP-LC3 plasmid (pBabe vector) was from Dr. Heesun Cheong, Division of Chemical Biology, Research Institute, National Cancer Center, Korea.

Instruments and programs

For developing of ELISA, the absorbance of 96-well plate was measured by BioTek Synergy HT Microplate reader. ChemiDoc^{TM} MP imaging system from Bio-Rad was used for analyzing chemiluminescent signal in western blotting assay. Following signal quantification was done by ImageLab 4.0 program provided by Bio-Rad.

DeltaVision Elite imaging system from GE Healthcare was used for imaging experiment. Objective lenses were equipped with Olympus IX-71 inverted microscope with PLAN APO 60×/Oil (PLAPON60×O), 1.42 NA, WD 0.15 mm. sCMOS camera and InSightSSI fluorescence illumination module were equipped with the system. Four-color fluorescent protein (Live Cell) filter set [GE Healthcare, 52-852113-013] was used for imaging. For live cell imaging, CO_2 supporting chamber with an objective air heater were installed with the system. Images were analyzed with SoftWorks program supported by GE Healthcare. Graphs and figures provided were analyzed with GraphPad Prism 5 program.

4. Experimental Procedures of Biological Assays

Cell Culture

HEK293T cell was cultured in Dulbecco modified eagle medium (DMEM) with 10% (v/v) fetal bovine serum (FBS) and 1% (v/v) antibiotic-antimycotic solution. HeLa, DU145, and Ca Ski cell were cultured in RPMI 1640 medium with 10% (v/v) FBS and 1% antibiotic-antimycotic solution. Both cells were maintained in 100-mm cell culture dish in an incubator at 37 ºC, in a humidified atmosphere with 5% CO_2.

ELISA

His-tagged human LRS were diluted in carbonate buffer (100 mM, pH 9.6) at the concentration of 0.5 ng μl^{-1}. Solution were distributed to the half-bottom 96 well clear plate from CORNING 3690. After incubation overnight at 4 °C (sealed), coating solution from each well was removed and washed for three times with phosphate buffered saline with 0.05% Tween® 20 (PBST). 5% bovine serum albumin (BSA) in PBS solution was treated to each well for blocking step, followed by the treatment of each compound and GST-tagged human RagD simultaneously for 3 h. GST protein itself was treated as negative control. Diluted GST antibody in PBST was added and incubated at room temperature for 1 h. After washing with PBST, the HRP-conjugated anti-horse IgG secondary antibody diluent was treated and incubated at room temperature for 1 h. TMB was added to each well for colorimetric development. Blue color should be developed in positive wells. To stop the color reaction, 1 M H_3PO_4 stopping solution was added. Finally, absorbance at 450 nm were measured.

Western Blotting

Cells were lysed with radio-immunoprecipitation assay (RIPA) buffer (50 mM Tris, pH 7.8, 150 mM NaCl, 0.5% deoxycholate, 1% IGEPAL CA-630, protease inhibitor cocktail, and phosphatase inhibitors). Protein was obtained after centrifugation at 15000 rpm for 20 min, by

transferring supernatant. Protein concentration was normalized with Micro BCA™ protein assay kit. Overall protein sampling procedure was done at 4 ºC. Prepared protein samples were analyzed with SDS-PAGE and following western blot procedure. Protein was transferred into nitrocellulose membrane after SDS-PAGE experiment. Membrane was blocked with 2% BSA in TBST over 1 h on r.t. Primary antibodies were treated overnight at 4 ºC [Anti-LC3B (ab51520); 1:1000, anti-S6K1 (ab32359); 1:1000, anti-phsopho-T389 S6K1 (ab2571); 1:800, anti-p62 (CST 5114); 1:1000, anti-glyceraldehyde-3-phosphate dehydrogenase (GAPDH) (CST 2118); 1:1000, anti-phospho-S65 4E-BP1 (CST 9451); 1:1000, anti-phospho-S757 ULK1 (CST 6888); 1:800, anti-phospho-S473 Akt (CST 4058); 1: 1000, anti-phospho-T172 AMPKα (CST 2531)], followed by washing with TBST. HRP-labeled anti-rabbit IgG secondary antibody (1:5000) was treated at room temperature for 1 h. Antibodies were treated with the concentration indicated in antibody manufacturer's protocol. After washing with TBST, membrane was developed by Amersham ECL prime solution. Chemiluminescent signal was measured by ChemiDoc™ MP imaging system.

Surface Plasmon Resonance (SPR) Assay

The dissociation rate constant (K_D) toward His-LRS was determined by surface plasmon resonance (SPR) technique at the national center for inter-university research facilities (NCRF) in Seoul National University using a Biacore T100 instrument from GE Healthcare. The carboxyl group on the surface of CM5 sensor chip was replaced with reactive succinimide ester using combination of 1-ethyl-3-(3-dimethylaminopropyl)-carbodiimide (EDC) and *N*-hydroxysuccinimide (NHS) in flow cells 1 and 2. Human LRS (1.5× PBS, pH 7.3) were immobilized on the flow cell 2 (aimed RU; 12000) through formation of amide bond by reacting with the resulting NHS ester. The remaining NHS ester on flow cells 1 and 2 was quenched by injection of 1 M ethanolamine-HCl (pH 8.0) solution. During the immobilization process, PBS was used as running buffer. After the immobilization of hLRS, compounds were injected for 60 s at a flow rate of 20 μl min^{-1} in various concentration from 1 μM to 15 μM. At the same flow rate, dissociation of compounds from the sensor surface was monitored for 200s. As a running buffer, 1× PBS (pH 7.3) containing 3% DMSO and 0.005% P20 solution were used. The binding events were measured at 25 °C. Data analysis were done by using Biacore

T100 Evaluation software from GE Healthcare. Final sensorgrams were obtained after the elimination of responses from flow cell 1 and buffer-only control. The dissociation constant (K_D) was calculated by fitting the sensorgrams to the 1:1 binding model.

Transfection

Cells were seeded in chambered coverglass from Nunc, 24 h prior to transfection. mCherry-GFP-LC3 plasmid was transfected to HeLa cell using Lipofectamine™ 2000 reagent. Transfection was preceded according to manufacturer's protocol.

mCherry-GFP-LC3 puncta imaging

DeltaVision Elite imaging system was used for the imaging of mCherry-GFP-LC3 transfected HeLa cell. For live-cell imaging, chamber was maintained with 37 ºC, 5% CO_2 condition. Image was obtained with 60× scale, using mCherry/mCherry, GFP/GFP (Excitation/Emission) filter sets. mCherry (Excitation: 575/25 nm, Emission: 625/45 nm); GFP (Excitation: 475/28 nm, Emission: 525/48 nm). Images were analyzed and merged with SoftWorks deconvolution software.

Cell proliferation assay

HEK293T cells were seeded in transparent 96-well plate from CORNING. 24 h after seeding, leucine-free DMEM and compound-DMEM solution (final compound concentration of 5 μM) were treated in each well after the removal of culture media. Cell proliferation assay was done with WSTs assay and cell proliferation ELISA, BrdU (colorimetric) kit, following the manufacturer's protocol.

Supplementary References

(1) Baumann, M., Baxendale, I. R. An overview of the synthetic routes to the best selling drugs containing 6-membered heterocycles. *Beilstein J. Org. Chem.* **7,** 442–495 (2011).

(2) Bauer, R. A., Wenderski, T. A. & Tan, D. S. Biomimetic diversity-oriented synthesis of benzannulated medium rings via ring expansion. *Nat. Chem. Biol.* **9,** 21–29 (2013).

(3) Stojković, M. R., Piotrowski, P., Schmuck, C. & Piantanida, I. A short, rigid linker between pyrene and guanidiniocarbonyl-pyrrole induced a new set of spectroscopic responses to the ds-DNA secondary structure. *Org. Biomol. Chem.* **13,** 1629–1633 (2015).

(4) Robichaud, A. J. *et al.* 3,4-Dihydropyrimido(1,2-*a*)indol-10(2H)-ones as potent non-peptidic inhibitors of caspase-3. *Bioorg. Med. Chem.* **17,** 7755–7768 (2009).

(5) Han, G. *et al.* Modification of cap group in δ-lactam-based histone deacetylase (HDAC) inhibitors. *Bioorg. Med. Chem. Lett.* **17,** 6234–6238 (2007).
